# The development of activity-based mannanase probes

**DOI:** 10.1039/d6sc04720c

**Published:** 2026-07-13

**Authors:** Massimo Tedeschi, Vincent A. J. Lit, Nicholas G. S. McGregor, Tessa Gote, Prajeesh Kooloth Valappil, Mark Arentshorst, Bogdan I. Florea, Berend Gagestein, Zachary Armstrong, Jeroen D. C. Codée, Alba Nin-Hill, Carme Rovira, Arthur F. J. Ram, Gideon J. Davies, Herman S. Overkleeft

**Affiliations:** a Leiden Institute of Chemistry, Leiden University Einsteinweg 55 2300 RA Leiden The Netherlands h.s.overkleeft@lic.leidenuniv.nl; b Institute of Biology Leiden, Leiden University Sylviusweg 72 2333 BE Leiden The Netherlands; c York Structural Biology Laboratory, Department of Chemistry, The University of York Heslington York YO10 5DD UK; d Department de Química Inorgànica i Orgànica & IQTCUB, Universitat de Barcelona Barcelona 08028 Spain; e Institució Catalana de Recerca i Estudis Avançats (ICREA) Barcelona 08020 Spain

## Abstract

β-Mannanases are endo-acting glycoside hydrolases (GHs) that cleave β-1,4 glycosidic linkages in mannan-rich plant cell wall polysaccharides. They find application in the food and paper industries. Activity-based probes (ABPs) are powerful tools for GH profiling in complex biological samples, yet to date, bespoke ABPs reporting on mannanases have not been reported. Here, we describe the synthesis of cyclophellitol-inspired ABPs based on mannobiose, mannotriose, and glucomannose, and their use in reporting mannanase activities in secretomes from saprophytic bacteria and fungi grown on mannan-containing biomass polysaccharides. In addition to mannanases, our ABPs also labelled cellulases in secretomes from both *Aspergillus niger* and *Cellvibrio japonicus*, which may indicate broader (“negative-subsite”) substrate specificity in these enzymes. Mechanistic proof of active-site nucleophile labelling by our ABPs was obtained for both *An*ManA and *Cj*Man26C by X-ray crystallography and for both *An*ManA and *An*Man26A by mass spectrometry. Together, our results establish mannanase-targeted ABPs that may find use alongside existing reagents that report on retaining GHs that process other bulk polysaccharides.

## Abstract 2

This paper describes the development of mannobiose, mannotriose, and glucomannose-based probes and their use in reporting on mannanase activities in secretomes from saprophytic bacteria and fungi grown on mannan-containing biomass polysaccharides.

## Graphical abstract

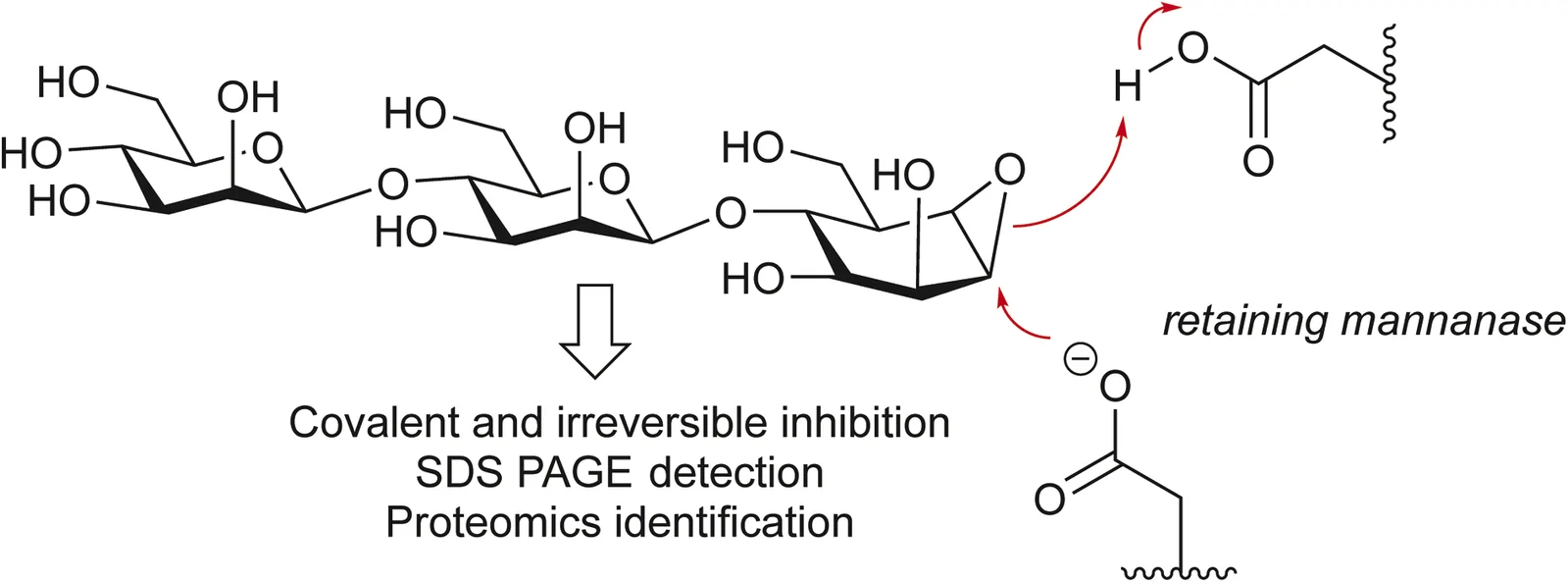

## Introduction

**Fig. 1:**
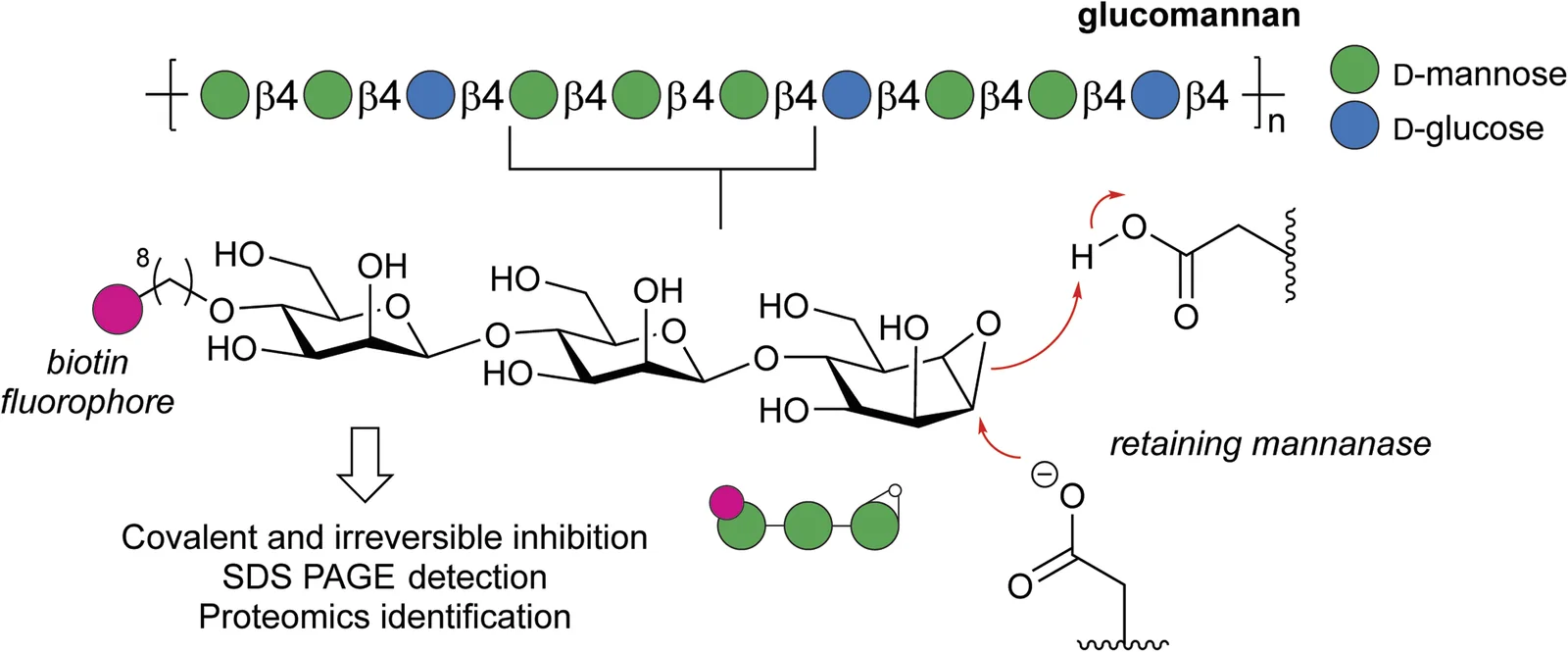
General strategy of the research reported here. GH5, GH26 and GH113 mannanases are retaining endoglycosidases that employ a Koshland double displacement mechanism in substrate hydrolysis. Tagged and glycosylated cyclophellitols covalently and irreversibly react with retaining glycosidases with substrate preference captured in the configuration and substitution pattern of the cyclophellitol ABP. This strategy allows for glycosidase capture, visualisation by SDS-PAGE and fluorescence scanning, and identification by affinity purification and mass spectrometry.

Hemicellulose, a group of heterogeneous polysaccharides, accounts for roughly 20–25% of lignocellulosic biomass.^1^ Among its main components is mannan which occurs predominantly as galactomannans and glucomannans, that contribute to the organization and mechanical strength of the plant cell wall through interactions with cellulose microfibrils. Galactomannan consists of a β-1,4-linked d-mannopyranose backbone with α-1,6-linked galactosyl side chains, while glucomannan features a mixed β-1,4-linked backbone of d-mannopyranose and d-glucopyranose, often acetylated at C2, C3, or C6.^2–4^ As the principal polysaccharide backbone of these hemicelluloses, mannan serves as the primary substrate for β-1,4-mannanases (E.C. 3.2.1.78), retaining glycosidases found in Carbohydrate Active enZyme (CAZy) families GH5, GH26 and GH113 (ref. 5 and 6) and that process their substrate following a Koshland two-step double displacement mechanism. The hydrolytic activity of mannanases is exploited in biotechnological processes to depolymerize plant biomass into valuable mannooligosaccharides and fermentable sugars. Mannanases are used in industry as detergent additives, for clarifying fruit juices and coffee extracts, in pulp bleaching during paper production, and in gel breaking during oil drilling.^7^ The ability to detect and report on mannanase activities is a prerequisite for improving and expanding on these industrial applications. One attractive strategy in this context is activity-based protein profiling (ABPP), which has been successfully applied to a variety of retaining endoglycosidases (xylanases, cellulases, amylases^8–10^) but not yet to mannanases. ABPP uses tagged, mechanism-based covalent and irreversible inhibitors to identify active enzymes within complex biological mixtures. These inhibitors, termed activity-based probes (ABPs), are composed of a recognition motif, a reactive group, and a reporter tag (Fig. 1).

Our work in this area is inspired by cyclophellitol, a natural product and mechanism-based inhibitor of retaining *exo*-β-glucosidases.^11^ Tagged cyclophellitol aziridines proved effective ABPs for a variety of retaining exoglycosidases with selectivity largely determined by the configuration and conformation of the ABP. Likewise, glycosylated, tagged cyclophellitol aziridines were found to select for retaining endoglycosidases from complex biological samples with selectivity again dictated by configuration and substitution pattern.^12,13^

Based on these studies and building on our prior work on retaining *exo*-mannosidase probes,^14^ we designed mannobiose, mannotriose, and glucomannose cyclophellitol inhibitors and ABPs for retaining *endo*-β-1,4-mannanases (and glucomannanases) to reflect the structural preferences of mannanases across GH5, GH26, and GH113 families (Fig. 1). Using gel-based and mass spectrometric approaches, we demonstrate selective, covalent labelling of mannanase active-site residues. We further establish activity-dependent probe binding by crystallography and proteomics and show that our probes report on mannanase activity in secretomes of the filamentous fungus *Aspergillus niger* and the Gram-negative bacterium *Cellvibrio japonicus* grown on mannan-containing media. Overall, our results expand the retaining endoglycosidase ABPP toolkit and may aid the discovery and optimisation of retaining GHs that process biomass polysaccharides for sustainable applications.

## Results

### Inhibitor and ABP synthesis

**Scheme 1:**
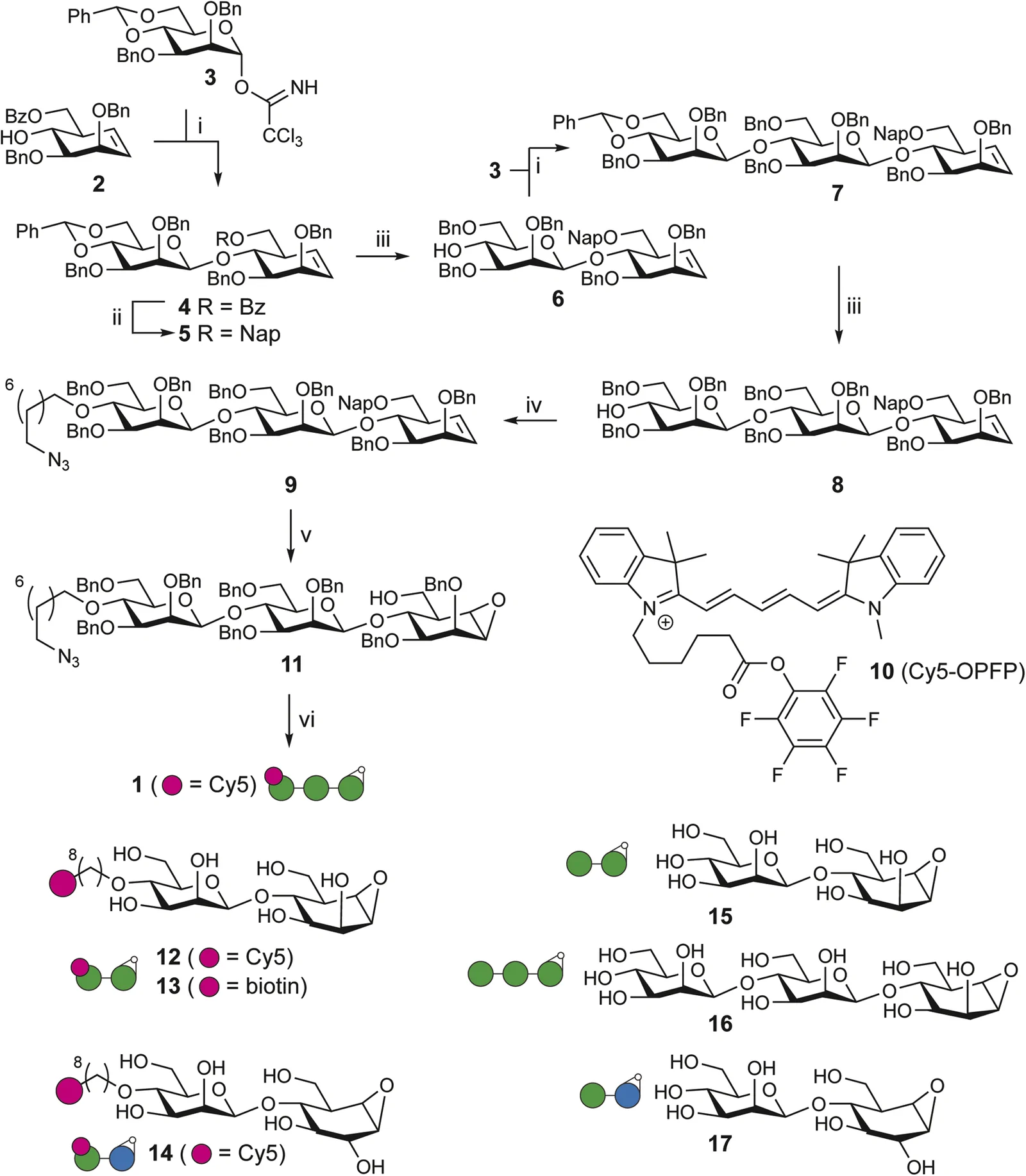
Inhibitor and probe design and synthesis of ABP 1. Reagents and conditions: (i) TMS-OTf, CH_2_Cl_2_, −45 °C (95% 4, 99% 7); (ii) (a) NaOMe, MeOH/CH_2_Cl_2_ (89%); (b) NapBr, NaH, TBAI, DMF (71%); (iii) CSA, MeOH, 50 °C, then 2-aminoethyl diphenylborinate, BnBr, KI, K_2_CO_3_, CH_3_CN (74% 6, 81% 8); (iv) 1-azido-8-iodooctane, NaH, DMF (77%); (v) DDQ, CH_2_Cl_2_/H_2_O (83%), then mCPBA, CH_2_Cl_2_, 0 °C (83%); (vi) Na, NH_3_, *t*-BuOH, THF, −60 °C (16%), then Cy5-OPFP 10, DiPEA, DMF (99%).

To study the range of mannanases across different systems, we first prepared a bespoke set of cyclophellitol-derived inhibitors and activity-based probes. Scheme 1 summarises this panel and presents, as a representative example, the synthesis of Cy5-Man-Man-*manno*-cyclophellitol 1. Full structures and synthetic/analytical details are provided in the SI. In addition to ABP 1, we prepared Cy5-Man-*manno*-cyclophellitol 12, biotin-Man-*manno*-cyclophellitol 13, and Cy5-Man-*gluco*-cyclophellitol 14 for comparative ABPP. We also synthesised non-tagged mechanism-based inhibitors 15–17 to verify the mode of action on which the ABPs were based. The synthesis of 1 commenced with glycosylation of orthogonally protected, mannose-configured cyclohexene 2 (the synthesis of which we described previously^15^) using 4,6-*O*-benzylidene-protected donor mannopyranoside 3 following the β-selective mannosylation protocol developed by Crich and coworkers.^16^ Following replacement of the 6′-*O*-benzoyl, for 6′-*O*-naphthyl, in the resultant pseudo-disaccharide 4, the benzylidene in 5 was removed under acidic conditions and the resulting free 6′-OH benzylated selectively over the free 4′-OH using Taylor's catalyst^17^ (2-ethyl diphenylborinate). Crich glycosylation of the thus obtained acceptor 6 with trichloroacetimidate mannoside 3 then afforded pseudotrisaccharide 7, treatment of which with camphor sulfonic acid (CSA) followed by regioselective benzylation following the sequence of events also used to transform 5 into 6 provided pseudotrisaccharide 8. A masked, late-stage conjugation handle (azide) was then introduced by alkylation of the 4″-OH in 8 with 1-azido-8-iodooctane. Selective demasking of the 6-OH in 9 (oxidative denaphthylation) was followed by homo-allylic epoxidation to give fully masked pseudotrisaccharidic epoxide 11. Birch reduction (lithium in ammonia) was then used to remove all benzyl protective groups as well as to reduce the azide in 11 to the primary amine. The latter was then condensed with Cy5-OPFP 10 to give Cy5-Man-Man-*manno*-cyclophellitol 1.

### Comparative ABPP on mannan-induced *A. niger* and *C. japonicus* secretomes

**Fig. 2:**
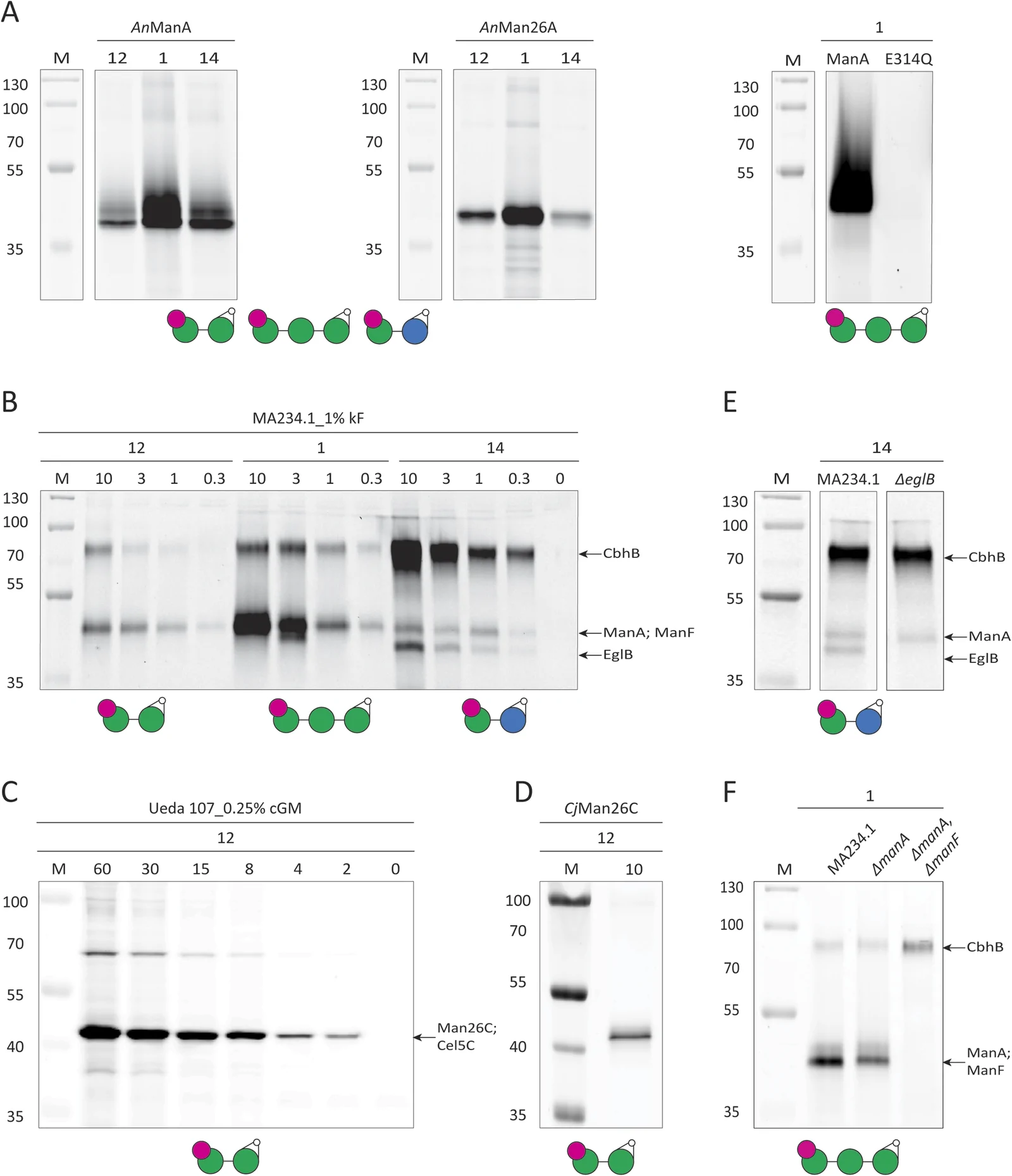
(A) Labelling of recombinant *A. niger* mannanases *An*ManA, *An*Man26A with 1, 12 and/or 14 and labelling with 1 of the secretomes of *A. niger* strains overexpressing either ManA or its E314Q mutant. (B) Comparative ABPP using ABPs 1, 12 and 14 at 0 to 10 µM final concentrations on secretomes of *A. niger* MA234.1 cultured on konjac flour (kF). (C) Comparative ABPP with 12 at 0 to 60 µM final concentrations of lysate of *C. japonicus* Ueda 107 grown on carob galactomannan (cGM). (D) Labelling of recombinant *C. japonicus* mannanase *Cj*Man26C with 12. (E) ABP labelling with 14 of the secretomes of the *A. niger* strains MA234.1 and Δ*eglB* grown on kF for 72 h. (F) Labelling with ABP 1 of the secretomes of *A. niger* MA234.1, Δ*manA* and Δ*manA;* Δ*manF* grown on kF for 72 h.

Following the synthesis, we tested the irreversible inhibition kinetics of Man-*manno*-cyclophellitol 15 on recombinant CjMan26C and CjMan26A. The obtained *k*_inact_/*K*_I_ were 18.65 M^−1^ s^−1^ for *Cj*Man26C (SI Fig. 1) and 4.12 M^−1^ s^−1^ for *Cj*Man26A (SI Fig. 2). Given the higher inhibition kinetics obtained with *Cj*Man26C we selected this enzyme over *Cj*Man26A for further biochemical and structural characterisation. To establish the ability of our probes to detect mannanase activity, we treated recombinant *A. niger* Man A (*An*ManA, NRRL3_08912) and *A. niger* Man26A (*An*Man26A, NRRL3_04196) with Cy5 ABPs 1, 12 and 14 at 5 µM final concentration. SDS PAGE of the probe-treated, denatured protein samples followed by in-gel fluorescence scanning revealed labelling of both proteins (fluorescent bands at around 40 kDa) for all three probes, with ABP 1 yielding the comparatively strongest signal with ABP 12 yielding the weakest one for *An*ManA and 14 the weakest one for *An*Man26A (Fig. 2A; SI Fig. 3). Treatment of the recombinant *An*ManA E314Q, thus the mutant in which the catalytic nucleophile (Glu) is substituted for an inactive amino acid residue (Gln), with ABP 1 gives no fluorescent band (Fig. 2A; SI Fig. 3 and 4). This result suggests that our probes are indeed activity-based – a result that is confirmed in experiments described in the sections on X-ray studies and mass spectrometry studies below.

We next performed comparative ABPP using Cy5-ABPs 1, 12 and 14 on secretomes of *A. niger* grown for 3 days on konjac flour (kF), a natural glucomannan derived from the tuber of *Amorphophallus konjac*. Secretomes were then harvested and incubated with the three ABPs at concentrations ranging from 0.3 to 10 µM. The secretome samples were denatured, the protein content resolved on SDS-PAGE and the resulting wet gel slabs scanned for in-gel fluorescence. Secretome treatment with Cy5-Man-*manno*-cyclophellitol 12 gave rise to two distinct fluorescence signals (Fig. 2B; SI Fig. 6–8) in a concentration-dependent manner, and which correspond to proteins with a molecular weight of around 40 kDa and around 75 kDa. The 40 kDa and 75 kDa signals also predominate in the Cy5-Man-Man-*manno*-cyclophellitol 1-treated samples, with fluorescence intensities indicating comparatively higher reactivity for (putative) enzymes corresponding to both protein bands. Cy5-Man-cyclophellitol 14 reveals a strong signal at 75 kDa, a 40 kDa signal of an intensity comparable with that elicited by 12, and a 37 kDa band that is not unveiled by the two b-mannose configured cyclophellitol probes, 1 and 12. In a similar comparative ABPP experiment, *C. japonicus* was grown on carob galactomannan (cGM) until cultures reached mid-log phase (OD_600_ ≈ 1.5), after which the cells were lysed, and the lysate treated with increasing concentrations of Cy5-Man-*manno*-cyclophellitol 12. A strong, ABP concentration-dependent band appeared between 40–55 kDa (Fig. 2C; SI Fig. 9), consistent with the known molecular weight of the GH26 mannanase, Man26C. Treatment of recombinant *Cj*Man26C with 12 revealed a fluorescent band at the same height (Fig. 2D), suggesting that the lower running band identified from ABPP on the *C. japonicus* extract may correspond to this GH26 mannanase. A fainter, higher molecular weight band (around 70 kDa) emerged in the gel derived from the *C. japonicus* extract labelling experiment, the identity of which could not be established at this time. The activity-based secretome profiling experiments described above indicate that our probes select for mannanases from secretomes of both *A. niger* and *C. japonicus* grown on mannan polysaccharides. Comparing the apparent molecular weight observed in the secretomes samples (Fig. 2B and C) with those of the ABP-treated recombinant enzymes (Fig. 2A and D), the lower band from the gel obtained from the ABP 1 and 12-treated *A. niger* samples may be either ManA or Man26A, while the lower band in the ABP 12-treated *C. japonicus* gel may be Man26C. No tentative assignment could be made for either the 80 kDa band labelled by both 1 and 12 or the 37 kDa band observed in the ABP 14-treated *A. niger* sample. To shed further light on this, we treated samples of *A. niger* secretomes and *C. japonicus* lysates also used for the experiments described in Fig. 2 with biotin-Man-*manno*-cyclophellitol 13. Biotin-modified proteins were then enriched using streptavidin-coated magnetic beads, digested with trypsin, and the resulting peptides were analysed by LC-MS/MS (Table 1). The *C. japonicus* pull-down experiment identified a single GH: the GH5 cellulase Cel5C. Man26C was not detected, suggesting that the major band in the lysate-labelling experiment (Fig. 2C) corresponds to Cel5C (≈42 kDa) rather than Man26C (≈47 kDa). No candidate GHs were identified for the higher-molecular-weight band (∼70 kDa) in Fig. 2C.

**Table 1:** Proteomics identification of GHs from biotin-Man-*manno*-cyclophellitol 13-treated secretomes. *C. japonicus*: Ueda 107 strain grown on 0.25% cGM; *A. niger*: NRRL3 strain grown on 1% kF. The complete list of protein groups are provided in the SI. emPAI = exponentially modified protein abundance index; log(Int) = base 10 logarithm of signal intensity

Organism	Uniprot ID	Protein	Score	Peptides	emPAI	Log(Int)
*C. japonicus*	B3PF55	Cel5C	64	2	0.2	NA
*A. niger*	A2QAI7	CbhB	105	3	NA	8.286
*A. niger*	A2QKT4	ManA	60	2	NA	8.546

From the *A. niger* pull-down experiment, two GHs were identified amongst the proteins with an identification score of above 10 and that were absent in the negative (non-ABP 13-treated samples) controls: the GH7 cellulase, CbhB (NRRL3_02584) and the GH5 mannanase, ManA. These results confirm that the fluorescent 40 kDa band observed in the gel depicted in Fig. 2B is indeed ManA and identifies the 75 kDa band in this gel as CbhB. The latter result demonstrates that these mannan-based cyclophellitol-based ABPs may be cross-reactive across multiple GH families, a feature we have observed in the past for retaining exoglycosidase-targeting probes, albeit rarely.^18^

The pulldown experiment did not produce a likely candidate for the 37 kDa protein identified in Fig. 2B (right panel) when using Cy5-Man-cyclophellitol 14 as the fluorescent probe. This 37 kDa protein did not appear in the comparative ABPP experiments using the mannose-configured ABPs 1 or 12, indicating that it is likely a cellulase rather than a classical mannanase. Whilst a biotin-modified version of Cy5-Man-cyclophellitol 14 might have identified the nature of this protein in a chemical proteomics experiment, we had not synthesised this probe, so we adopted an alternative strategy.

We realised that the *A. niger* GH5 endoglucanase, EglB (NRRL3_04917) has a molecular weight of about 36.6 kDa. We therefore created a *eglB* knock-out in the MA234.1 background (SI Fig. 10 and 11A), grew it on konjac flour for 3 days, and then harvested the secretomes. Treatment of these with ABP 14 indeed showed loss of the corresponding band, confirming the identity of this protein band as EglB (Fig. 2E; SI Fig. 12A).^19^ In a similar experiment, treatment of secretomes from a Δ*manA* strain cultured on konjac flour, with ABP 1, revealed the presence of protein having the same molecular weight as ManA (SI Fig. 11B, 12A andB). Therefore, we looked among the secreted proteins for a mannanase with a molecular weight like ManA (SI Fig. 10). A protein candidate fitting the description was ManF (NRRL3_02585). Consequently, the Δ*manF*; Δ*manA* strain was produced (SI Fig. 11C) and grown on kF. Treatment of the Δ*manF*; Δ*manA* secretome with 1 showed loss of the corresponding band confirming ManF as protein labelled in the Δ*manA* strain (Fig. 2F; SI Fig. 12C). *An*Man26A was not detected in these *A. niger* secretomes (SI Fig. 10, 11D and 12A).

### Mannanase ABPs react *via* active-site nucleophile modification

As described above, ABP 1 modified recombinant *An*ManA but not the catalytic nucleophile mutant *An*ManA E314Q (Fig. 2A), providing clear evidence that our probes modify their target enzymes by covalent and irreversible inhibition following the mechanism depicted in Fig. 1. To further establish this activity-based mode of action we performed a series of analytical and structural biology experiments on selected mannanases from *A. niger* and *C. japonicus*.

**Fig. 3:**
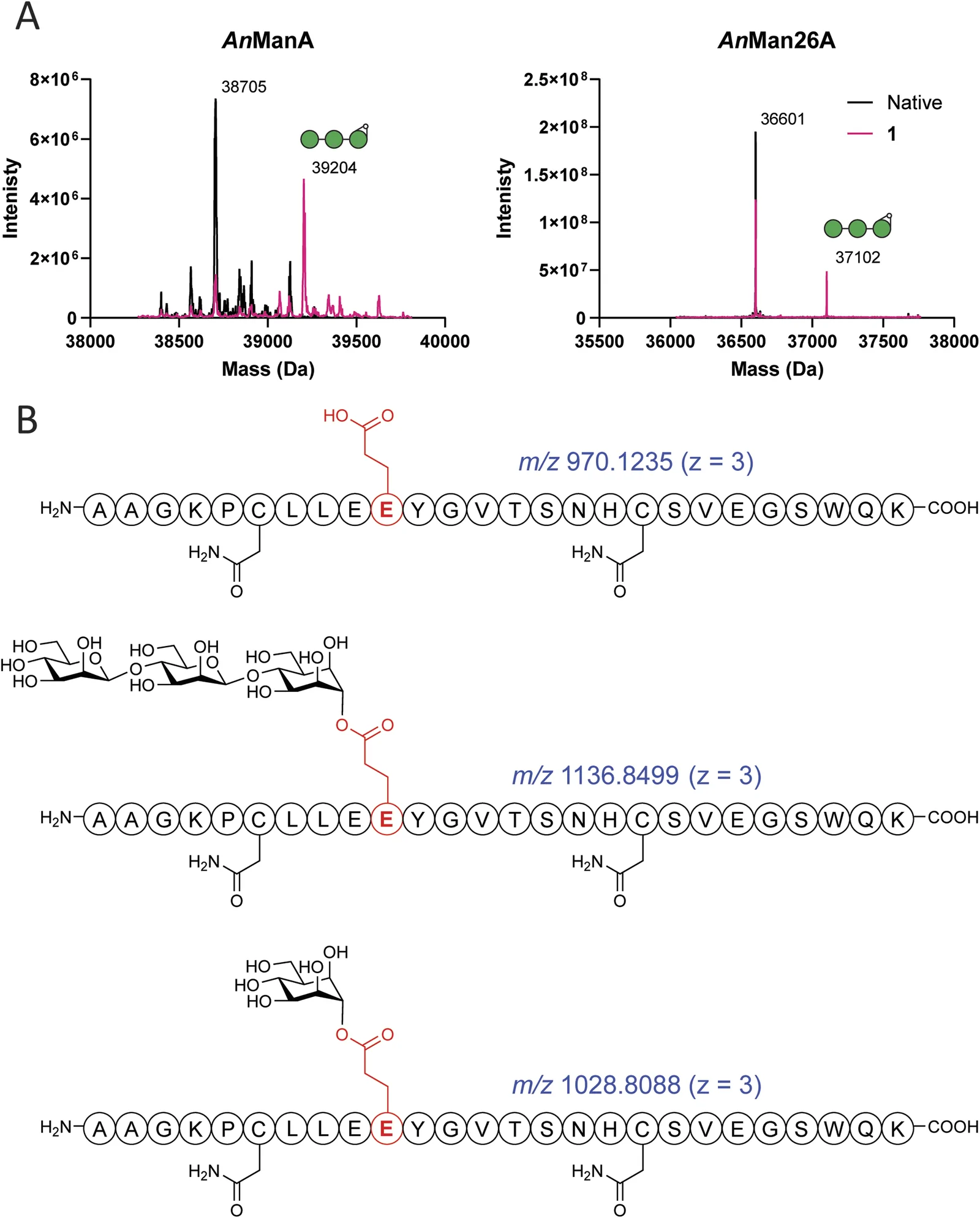
(A) Deconvoluted intact MS spectra of recombinant *An*ManA and *An*Man26A (black) overlaid with the same enzymes reacted with Man-Man-*manno*-cyclophellitol 16. (B) The *An*ManA tryptic peptide containing the active-site nucleophile E314 (highlighted in red) as identified by LC-MSMS before and after reacting the recombinant protein with Man-Man-*manno*-cyclophellitol 16.

As a first experiment in this context, we treated the recombinant and purified *A. niger* mannanases, *An*ManA and *An*Man26A (10 µM solution in McIlvaine buffer, pH 5, 25 µL) with Man-Man-*manno*-cyclophellitol 16 (2.5 µL, 5 mM in DMSO) for two hours at 37 °C. The intact masses of inhibitor-treated and non-treated proteins were then determined, revealing a mass difference of about 500 Da between treated and non-treated samples, which corresponds to the molecular weight of compound 16, for both proteins (Fig. 3A). In the same vein, treatment of recombinant, purified *Cj*Man26C with Man-*manno*-cyclophellitol 15 yielded a protein with an intact mass of 338 Da higher than non-treated protein (SI Fig. 13). Having established that Man-Man-*manno*-cyclophellitol 16 binds to *An*ManA and *An*Man26A tightly enough to form a mass spectrometry–identifiable complex, we set out to establish whether, as intended (see Fig. 1), the active site nucleophile reacts with the epoxide to form a covalent and irreversible bond. For this purpose, we incubated 2 µg of recombinant, purified *An*ManA in McIlvaine buffer at pH 5 (10 µL) with 1 µL of a 10 mM solution of 16 in DMSO for 2 hours at 37 °C. Inhibitor-treated and non-treated protein samples were then denatured with 8 M urea in 50 mM aqueous NH_4_HCO_3_ (pH 8) to 50 µL sample size. Potential disulfides were reduced with TCEP (50 mM, 1.2 µL) and cysteine thiols capped by treatment with 55 mM iodoacetamide (3.3 µL). Samples were then further processed (see SI) to allow tryptic digestion, and the tryptic digests then analysed and identified by LC-MSMS. Using this approach, a tryptic peptide containing the active-site nucleophile Glu314 was identified in the sample containing no-inhibitor-treated *An*ManA, with an *m*/*z* of 970.12 (*z* = 3) corresponding to the 26-mer peptide as shown in Fig. 3B in which both cysteines are alkylated (SI Fig. 14). Inhibitor 16-treated *An*ManA instead yields a tryptic peptide with an *m*/*z* of 1136.84 (*z* = 3), which corresponds to the same cysteine-alkylated 26-mer peptide, covalently bound to one equivalent of inhibitor 16. MS/MS fragmentation of this ABP-bound peptide revealed loss of a mannobiosyl moiety, yielding the 26-mer peptide bound to ring-opened *manno*-cyclophellitol with an *m/z* of 1028.81 (*z* = 3) (SI Fig. 15 and 16). The same phenomenon was observed with inhibitor 15 (SI Fig. 17 and 18). In the same vein, we could identify the tryptic peptides containing the active site nucleophile of *An*Man26A (SI Fig. 19). As discussed above, *An*ManA E314Q is not targeted by Cy5-Man-Man-*manno*-cyclophellitol 1 indicating that it is E314, and not one of the other glutamate residues in the tryptic peptides that is modified (neither are the cysteines modified as otherwise they would not be returned as *S*-alkyl moieties in this experiment).

**Fig. 4:**
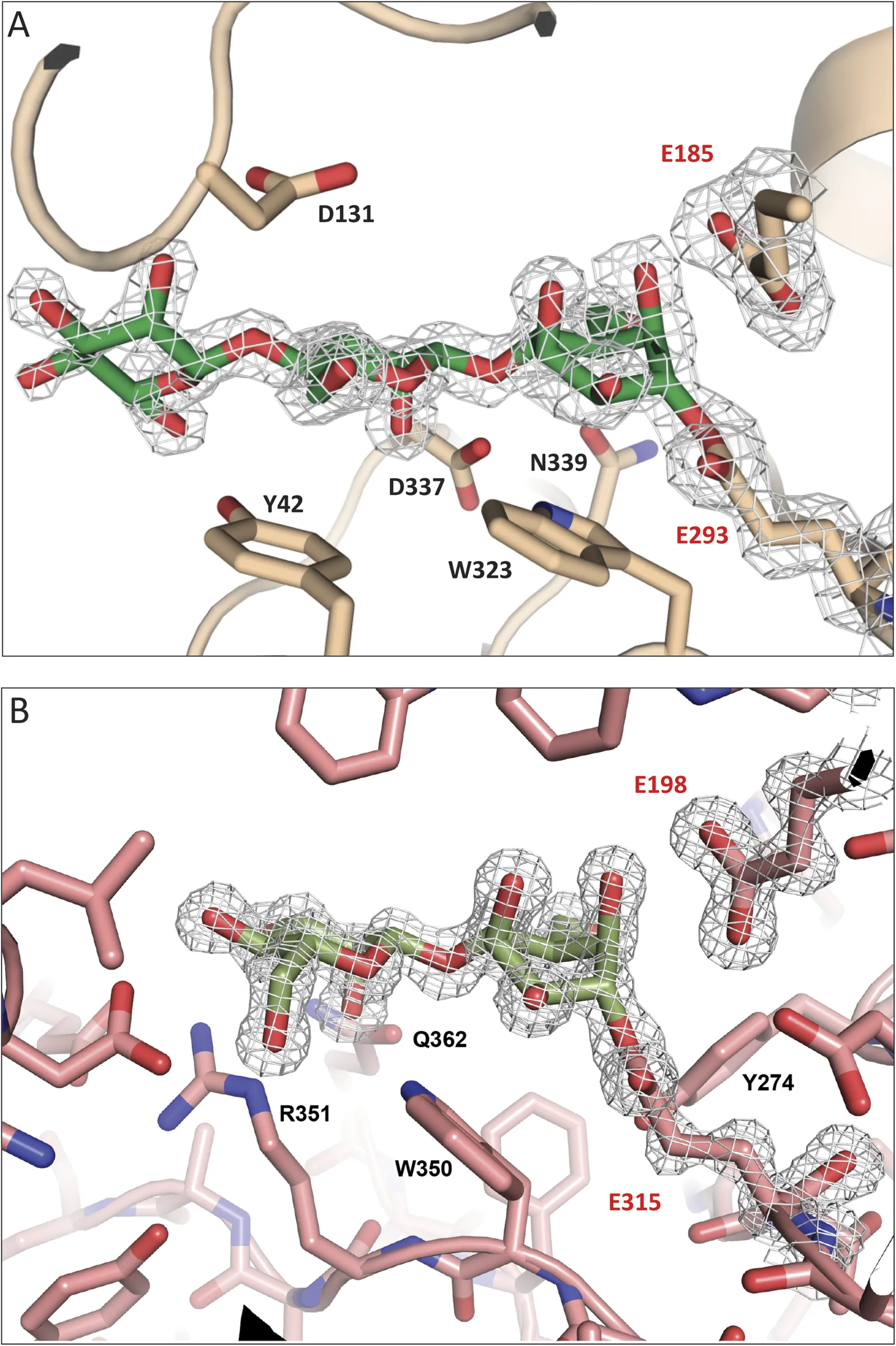
(A) Structure of *An*ManA reacted with Man-man-*manno*-cyclophellitol 16. Experimentally determined electron density (2*F*_o_–*F*_c_, contoured at 1*σ*, 0.49 e A^−3^) for 16 and the catalytic amino acid side chains (E185 and E293) are shown as a grey mesh. (B) Structure of *Cj*Man26C reacted with Man-*manno*-cyclophellitol 15. Experimentally determined electron density (2*F*_o_–*F*_c_, contoured at 1.5*σ*, 0.62 e A^−3^) for 15 and the catalytic amino acid side chains (E198 and E315) are shown as a grey mesh. H177, which forms a polar contact with the O3 hydroxyl oxygen of *manno*-cyclophellitol of 15 was omitted from the figure for visual clarity. For both structures the polypeptide is shown in cartoon form with active-site residues shown as sticks. The names of the catalytic nucleophile and acid/base residues have been reported in red.

To unambiguously establish the nature of the inhibitor/ABP-modified residue we obtained crystals from recombinant, purified *An*ManA and *Cj*Man26C, and incubated these with Man-man-*manno*-cyclophellitol 16 and Man-*manno*-cyclophellitol 15 respectively. These crystals yielded X-ray diffraction structures at 1.42 Å (*An*ManA) (Fig. 4A) and 1.16 Å (*Cj*Man26C) (Fig. 4B; SI Fig. 20). Both structures contained the inhibitors covalently attached to the requisite catalytic nucleophile (E293 for *An*ManA, E315 for *Cj*Man26C) through an ester linkage. These inhibitor-enzyme adducts are the expected products from the acid catalysed epoxide ring opening (see Fig. 1 for mechanism). In the structures, the catalytic acid/base residue (E185 for *An*ManA, E198 for *Cj*Man26C), is positioned within the expected distance for proton transfer to the ring-opened reducing end moiety. The −1 subsite ring adopts a ^O^S_2_ conformation consistent with the covalent intermediate conformation from the catalytic itinerary of *endo*-mannanases.^20–22^ The mannose residues in the −2 and −3 subsites, however, adopt unstrained ^4^C_1_ chair conformations. These structures not only confirm that 15 and 16 covalently inhibit *endo*-mannanases but also appear to faithfully recapitulate protein-substrate interactions and the catalytic itinerary of the natural substrate.

### ABP analysis of pH- and temperature-dependent mannanase activity

**Fig. 5:**
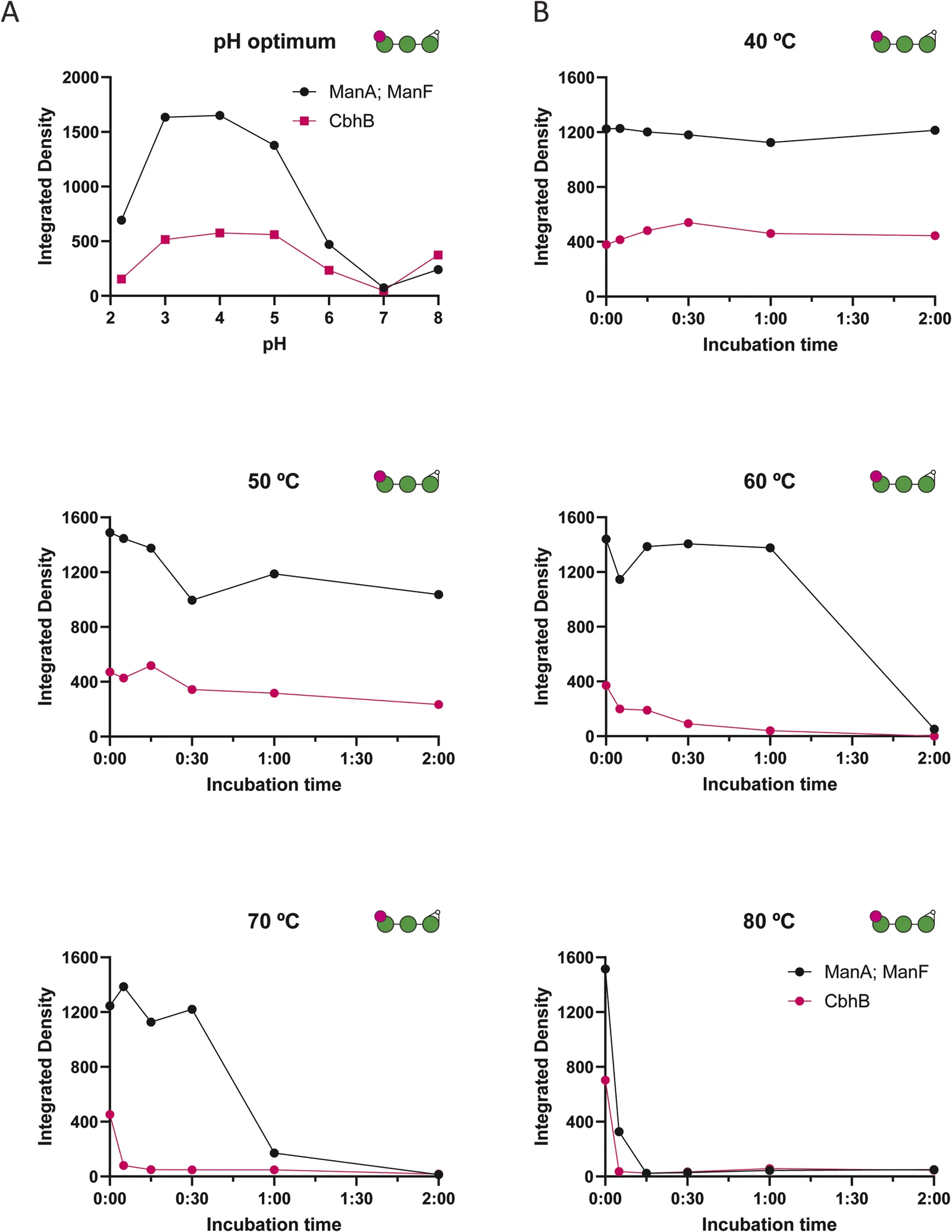
(A) Effect of pH on *A. niger* mannanase activities. The graph shows the integrated density of the SDS-page bands after treatment of secretomes of *A. niger* grown for 72 h on kF with Cy5-Man-Man-*manno*-cyclophellitol 1 and the samples were then exchanged into McIlvaine buffer at different pHs. (B) Effect of temperature on *A. niger* mannanase activities. The graphs show the integrated intensity of SDS-page bands after treatment of the secretome of *A. niger* grown on kF for 72 h with Cy5-Man-Man-*manno*-cyclophellitol 1 at different temperatures for up to 2 hours.

Industrial biomass degradation often occurs under challenging conditions. We therefore used ABPP as a rapid readout of enzyme resilience across pH and temperature. Specifically, we monitored the secretome endoglycosidases CbhB and ManA/ManF from *A. niger* cultures grown on konjac flour (Fig. 5). To establish pH sensitivity, secretomes were harvested 72 hours after biomass transfer, and individual aliquots were exchanged to McIlvaine buffer at pH 2.2, 3, 4, 5, 6, 7, and 8. Then, 15 µL of each sample was treated with 1.5 µL of a 50 µM stock of Cy5-Man-Man-*manno*-cyclophellitol 1. Following incubation for 1 hour at 37 °C, the samples were heat-denatured, resolved on SDS-PAGE and the wet gel slabs scanned for in-gel fluorescence as detailed before (SI Fig. 21). The integrated density of each band was quantified and used to generate the graph in Fig. 5A. Cy5-Man-man-*manno*-cyclophellitol 1 labelling appears most effective at pH 3–5, indicating that all probe-responsive enzymes perform best under acidic conditions. We next applied the same workflow to assess thermal stability across 40–80 °C.

To assess the effect of temperature on secretome GH activity, aliquots of *A. niger* secretomes obtained as for the pH sensitivity experiments were brought to 40 °C, 50 °C, 60 °C, 70 °C or 80 °C for varying time periods up to two hours. They were then treated with Man-Man-*manno*-cyclophellitol 1, denatured, resolved by SDS-PAGE, and analysed as before (SI Fig. 22), and the band integrated density plotted; ManA/ManF can resist elevated temperatures up to 1 h at 60 °C and 30 min at 70 °C (Fig. 5B).

## Discussion

Mannans are major components of renewable feedstocks, utilisation of which requires access to mannanases able to perform under harsh industrial conditions. To assist in the discovery and improvement of such enzymes, we developed activity-based mannanase profiling technology, the results of which are presented here. We synthesised three bespoke ABPs, Cy5-Man-man-manno-cyclophellitol 1, Cy5-Man-manno-cyclophellitol 12 and Cy5-Man-cyclophellitol 14 using a strategy that allows bespoke structural variation in both the oligosaccharide and the reporter entity. The activity-based nature of the probes was demonstrated on recombinant mannanases from *A. niger* and *C. japonicus* and their mechanism-based nature by proteomics and X-ray crystallography. Probe 1 proved to be the most efficient in modifying most enzymes tested, though recombinant GH5 *An*ManA and GH26 *An*Man26A appeared to react about equally well with each of the three probes.

As a proof of concept of the utility of our probes, they were then employed to report on mannanase resilience to variations in temperature and pH in secretomes of *A. niger* and *C. japonicus* (SI Fig. 23) grown on mannans. Our manno-cyclophellitol probes 1 and 12 show cross-reactivity for GH5 and GH7 cellulases both in *A. niger* secretomes and *C. japonicus* lysates. This cross-reactivity (reflecting “−” subsite preferences) may be because of the structural similarity of natural substrates such as glucomannan and galactoglucomannan, which possess a β-1,4-linked backbone containing both gluco- and mannopyranose units. Previous research has shown how both cellulases and mannanases can degrade these substrates.^23–26^ Furthermore, since both CAZy families GH5 and GH26 contain both cellulases and mannanases,^5^ cellulolytic enzymes might have evolved to degrade mannan-containing polysaccharides.^26^ Comparative ABPP with Man-man-*manno*-cyclophellitol 1, Man-cyclophellitol 14 and the previously developed^8^ Glu-cyclophellitol could bring further insight into the substrate specificity of GH5 and GH26 family cellulases and mannanases.

**Fig. 6:**
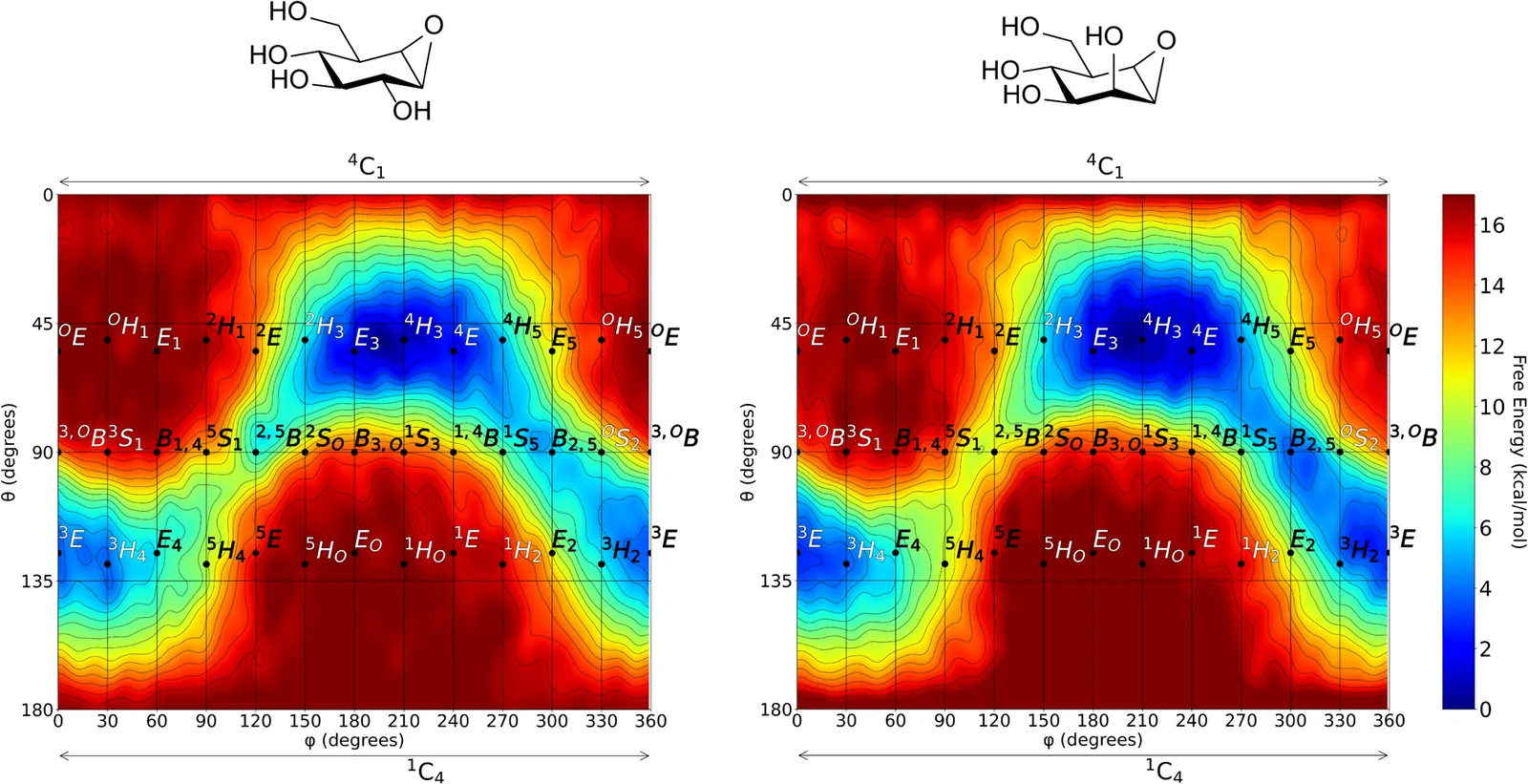
Free energy landscape of cyclophellitol (left) and *manno*-cyclophellitol (right). Isolines at 1 kcal mol^−1^.

An alternative explanation for the observed cross-reactivity may be in the conformational behaviour of our *manno*-cyclophellitol probes and how this reflects transition state mimicry of mannanase processing of β-mannosidase substrates besides that of β-glucosidase-processing of β-glucosides. We previously reported^27^ the free energy landscape (FEL) of cyclophellitol (which emulates β-glucopyranose configuration), with the ^4^H_3_ conformer as the energetically most stable (Fig. 6). β-Glucosidic bond cleavage as catalysed by retaining β-glucosidases (*endo* and *exo*) proceeds through ^4^H_3_ transition states which explains the observed activity and selectivity of cyclophellitol-based probes. Here we also calculated the FEL of *manno*-cyclophellitol (Fig. 6, right panel), which likewise displays ^4^H_3_ as the global minimum, closely resembling the landscape of *gluco*-cyclophellitol (Fig. 6, left panel). Despite the overall similarity with *gluco*-cyclophellitol, *manno*-cyclophellitol displays a slightly more pronounced local minimum near the B_2_,_5_ region, indicating a subtly different conformational balance towards the preferred TS conformation in mannanases and β-mannosidases, which typically process substrates through B_2,5_ or ^3^H_4_ transition states.^28^ Both conformations are accessible to *manno*-cyclophellitol: they appear as local minima approximately 2 and 3 kcal mol^−1^ above the ^4^H_3_ ground state, and the barrier for interconversion to B_2,5_ is about 4 kcal mol^−1^.

Thus, modest energetic adjustment from the enzyme is required to bias the inhibitor toward the catalytically preferred transition state.

In conclusion, we have developed and validated the first set of activity-based probes capable of reporting mannanase activity in complex biological samples, adding to the growing number of *endo-*GH targeting probes for analysing microbial machineries able to degrade biomass polysaccharides for a sustainable future.

## Author contributions

M. T., N. G. S. M., P. K. V., M. A., and Z. A. carried out the biochemical work. V. A. J. L. synthesised all compounds under the supervision of J. D. C. C. B. I. F., B. G., T. G. and N. G. S. M. conducted the structural and bio-analytical work. A. N-H. and C. R. calculated compound free energy landscapes. A. F. J. R., G. J. D. and H. S. O. supervised the work, which was conceived by G. J. D. and H. S. O. M. T., H. S. O. and G. J. D. wrote the manuscript with input from all authors.

## Conflicts of interest

There are no conflicts to declare.

## Supplementary Material

SC-017-D6SC04720C-s001

## Data Availability

Coordinates and observed structure factors have been deposited in the protein databank at https://www.rcsb.org/, reference number 31AO and 29QD. Metadynamics trajectory, hills and free energy surface files for the *manno*-cyclophellitol simulation performed in this work are provided in the Zenodo repository (https://www.zenodo.org), see: https://doi.org/10.5281/zenodo.19251019. *A. niger* mass spectrometry proteomics data have been deposited to the ProteomeXchange Consortium *via* the PRIDE^29^ partner repository with the dataset identifier PXD076800. Supplementary information (SI): detailed experimental conditions and methods: enzyme production, structure solution, characterization, biological assays, and organic synthesis. See DOI: https://doi.org/10.1039/d6sc04720c.
