# Supplementary material for: The development of activity-based mannanase probes
Source: Chem Sci. 2026 Jul 13. Online ahead of print. doi: 10.1039/d6sc04720c (PMC13358891; doi:10.1039/d6sc04720c)
Supplement: SC-OLF-D6SC04720C-s001 [file SC-OLF-D6SC04720C-s001.pdf]

Supplementary Information (SI) for Chemical Science.
This journal is © The Royal Society of Chemistry 2026

Supplementary information for

The Development of Activity-Based Mannanase Probes

Massimo Tedeschi,†[a,b] Vincent A. J. Lit,†[a] Nicholas G. S. McGregor,[c] Tessa Gote,[a] Prajeesh Kooloth Valappil,[b] Mark Arentshorst,[b] Bogdan I. Florea,[a] Berend Gagestein,[a] Zachary Armstrong,[a] Jeroen D. C. Codée,[a] Alba Nin-Hill, [d] Carme Rovira,[d,e] Arthur F. J. Ram,[b] Gideon J. Davies,*[c] and Herman S. Overkleeft*[a]

a. Leiden Institute of Chemistry, Leiden University, Einsteinweg 55, 2300 RA Leiden, The Netherlands
b. Institute of Biology Leiden, Leiden University, Sylviusweg 72, 2333 BE Leiden, The Netherlands
c. York Structural Biology Laboratory, Department of Chemistry, The University of York, Heslington, York, YO10 5DD, UK.
d. Department de Química Inorgànica I Orgànica & IQTCUB, Universitat de Barcelona, Barcelona 08028, Spain
e. Institució Catalana de Recerca I Estudis Avançats (ECREA), Barcelona 08020, Spain

Table of contents

Synthesis of β-mannanase probes and inhibitors 2
Biochemistry and molecular biology methods 18
Supporting Figures 29
NMR spectra 57
References 90

Synthesis of β-mannanase probes and inhibitors

General synthesis procedures: Reagents and solvents were purchased from Sigma Aldrich, Acros, Fluorochem, VWR, Carbosynth, Fischer Scientific and Merck. Chemicals were used as received unless stated otherwise. Toluene, THF, DMF and ACN were stored over flame-dried molecular sieves (either 3 or 4 Å) before use. For the reactions that required anhydrous conditions traces of water were removed by co-evaporation with toluene. All reactions were performed under a nitrogen atmosphere unless stated otherwise. Thin layer chromatography was performed on Merck aluminium sheets. (Silica gel 60 F_{254}) For initial UV detection a lamp set to 254 nm was used. Spots were further visualised by spraying with a solution of $(NH_4)_6Mo_7O_{24}·4H_2O$ (25 g/L) and $(NH_4)_4Ce(SO_4)_4·2H_2O$ (10 g/L) in 10% sulfuric acid or a solution of $KMnO_4$ (20 g/L) and K_2CO_3 (10 g/L) in water, followed by charring at ~150 ˚C. Flash column chromatography was performed using Screening Device b.v. silica gel (particle size of 40 – 63 µm, pore diameter of 60 Å) with the indicated eluents. ^{1}H NMR and ^{13}C NMR spectra were recorded on a Bruker DPX-300 (300 and 75 MHz respectively), Brüker AV-400 (400 and 101 MHz respectively), a Bruker AV--500 (500 and 126 MHz respectively), or a Bruker-850 (800 and 200 MHz respectively) spectrometer in the given solvent. Chemical shifts are reported in ppm (δ) relative to the residual solvent peak or tetramethylsilane (0 ppm) as internal standard and coupling constants are given in Hz. Gel filtration was performed on an Äkta explorer (GE Healthcare) using 1.6x60 cm Toyopearl HW-40S resin. Elution of the compounds was done with a solution of 1% AcOH in ACN/H_2O, 1/9, v/v and fraction monitoring was done using refractive index. For reversed phase HPLC purifications an Agilent Technologies 1200 series instrument equipped with a semi-preparative column (Gemini C18, 250 x 10 mm, 5 µm particle size, Phenomenex) was used. LC/MS analysis was performed on a Surveyor HPLC system (Thermo Finnigan) equipped with a C_{18} column (Gemini, 4.6 mm x 50 mm, 5 µm particle size, Phenomenex), coupled to a LCQ Adventage Max (Thermo Finnigan) ion-trap spectrometer (ESI^+). The applied buffers were H_2O, MeCN and 1% aqueous TFA. High-resolution mass spectrometry (HRMS) analysis was performed with a LTQ Orbitrap mass spectrometer (Thermo Finnigan), equipped with an electron spray ion source in positive mode (source voltage 3.5 kV, sheath gas flow 10 mL/min, capillary temperature 250 °C) with resolution R = 60000 at m/z 400 (mass range m/z = 150 – 2000) and dioctyl phthalate (m/z = 391.28428) as a “lock mass”. The high-resolution mass spectrometer was calibrated prior to measurements with a calibration mixture (Thermo Finnigan).

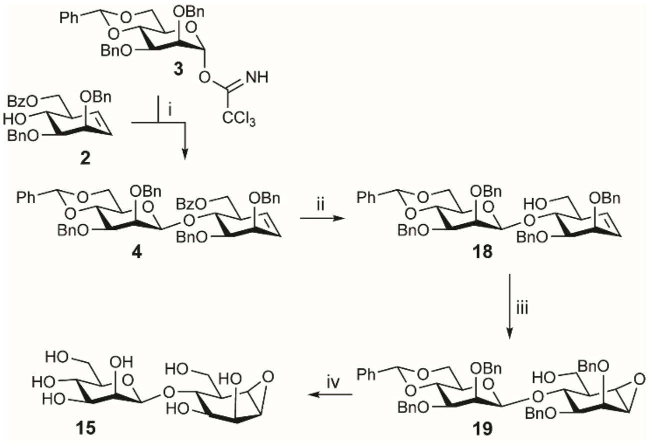

SI scheme 1. i) TMS-OTf, DCM, -45 °C, 95%. ii) NaOMe, MeOH/DCM, 89%. iii) mCPBA, DCM 4 °C, 83%. iv) Na, NH_3, *t*-BuOH, THF, -60 °C, 77%.

6-*O*-benzoyl-2,3-di-*O*-benzyl-manno-cyclohexene (**2**): 2,3-di-*O*-benzyl-manno-cyclohexene[1] (0.19 g, 0.56 mmol, 1 eq.) was co-evaporated with anhydrous toluene thrice and dissolved in 2.0 mL anhydrous DCM. Benzoyl chloride (0.078 mL, 0.67 mmol, 1.2 eq.) and Et_3N (0.38 mL, 2.80 mmol, 5 eq.) were added and the reaction was left to stir for one hour at 0 °C. After TLC showed full conversion of the starting material, the reaction was quenched with H_2O, concentrated *in vacuo* and re-dissolved in EtOAc. The

organic layer was washed with sat. aq. $NaHCO_3$, brine, dried over $MgSO_4$, filtered and concentrated *in vacuo*. The crude product was purified using flash silica column chromatography (EtOAc/pentane, 1/20 -> 1/10 -> 1/5, v/v) to obtain compound **2** as a colourless oil (0.25 g, 0.52 mmol, 92%). ^{1}H NMR (400 MHz, $CDCl_3$) δ 8.05 – 7.91 (m, 2H), 7.56 – 7.45 (m, 1H), 7.42 – 7.21 (m, 13H), 5.77 (dt, *J* = 10.2, 2.4 Hz, 1H), 5.67 (dt, *J* = 10.3, 2.0 Hz, 1H), 5.01 (d, *J* = 11.3 Hz, 1H), 4.76 (d, *J* = 11.3 Hz, 1H), 4.65 (q, 3H), 4.56 (dd, *J* = 10.9, 3.7 Hz, 1H), 4.42 (dd, *J* = 10.9, 5.5 Hz, 1H), 4.23 – 4.16 (m, 1H), 3.77 – 3.58 (m, 2H), 2.72 – 2.60 (m, 1H). ^{13}C NMR (101 MHz, $CDCl_3$) δ 166.7, 138.7, 138.0, 133.0, 131.6, 130.3, 129.8, 128.7, 128.5, 128.4, 128.1, 128.1, 128.0, 127.8, 126.1, 81.3, 77.5, 77.2, 76.8, 71.7, 71.4, 69.4, 66.8, 64.5, 44.6. HRMS calculated for $[C_{28}H_{28}O_5 + Na]^+$: 467.1829, found 467.1827.

2,3-di-*O*-benzyl-4,6-*O*-benzylidene-(1-4)-β-D-manopyranosyl-6-*O*-benzoyl-2,3-di-*O*-benzyl-manno-cyclohexene (**4**): Donor **3** (1.60 g, 2.70 mmol, 2.0 eq.) and acceptor **2** (0.60 g, 1.35 mmol, 1 eq.) were co-evaporated with anhydrous toluene thrice. The mixture was dissolved in 20 mL anhydrous DCM and cooled to -45 °C. TMSOTf (0.052 mL, 0.27 mmol, 0.2 eq.) was added and the reaction was left to stir for 30 minutes at -45 °C. Subsequently the reaction was quenched with sat. aq. $NaHCO_3$, the water layer re-extracted with DCM twice, the organic layers combined and washed with brine, dried over $MgSO_4$, filtered and concentrated *in vacuo*. The crude product was purified using flash silica column chromatography (EtOAc/pentane, 1/20 -> 3/20, v/v) followed by a SEC (Sephadex-LH20, MeOH/DCM, 1:1, v/v) to remove any traces of acceptor to obtain compound 4 as colourless oil (1.13 g, 1.28 mmol, 95%). ^{1}H NMR (500 MHz, $CDCl_3$) δ 8.02 (q, 2H), 7.59 – 7.53 (m, 1H), 7.51 – 7.19 (m, 35H), 5.96 – 5.91 (m, 1H), 5.76 – 5.71 (m, 1H), 5.56 (s, 1H), 4.86 – 4.74 (m, 3H), 4.71 – 4.54 (m, 7H), 4.46 – 4.36 (m, 2H), 4.28 – 4.22 (m, 2H), 4.13 (t, *J* = 9.6 Hz, 1H), 4.05 – 4.01 (m, 2H), 3.86 (dd, *J* = 3.1, 1.0 Hz, 1H), 3.79 (t, *J* = 10.2 Hz, 1H), 3.51 (dd, *J* = 9.9, 3.1 Hz, 1H), 3.13 – 3.07 (m, 1H), 2.65 – 2.59 (m, 1H).^{13}C NMR (126 MHz, $CDCl_3$) δ 166.4, 138.7, 138.6, 138.5, 138.4, 137.7, 133.4, 130.2, 129.8, 129.6, 129.0, 128.6, 128.6, 128.5, 128.5, 128.4, 128.4, 128.3, 128.3, 128.2, 128.1, 128.0, 128.0, 127.8, 127.8, 127.7, 127.7, 127.6, 127.6, 127.6, 126.7, 126.2, 101.6, 100.9, 78.6, 78.2, 77.4, 77.2, 76.9, 76.5, 75.8, 74.9, 74.3, 73.3, 72.9, 72.5, 71.4, 68.5, 67.7, 65.3, 41.3, 30.4, 29.8. HRMS calculated for $[C_{55}H_{54}O_{10} + Na]^+$: 897.3609, found 897.3609.

2,3-di-*O*-benzyl-4,6-*O*-benzylidene-(1-4)-β-D-manopyranosyl-2,3-di-*O*-benzyl-manno-cyclohexene (**18**): Compound **4** (0.25 g, 0.29 mmol, 1 eq.) was suspended in 2 mL MeOH containing a few drops of DCM. A catalytic amount of NaOMe was added, and the reaction mixture was left to stir overnight. The reaction mixture was quenched with Amberlite IRC120 H^+, filtered and concentrated *in vacuo*. The crude product was purified using column chromatography (EtOAc/pentane, 3/10, v/v) to obtain compound **18** (0.20 g, 0.26 mmol, 89%). ^{1}H NMR (500 MHz, $CDCl_3$) δ 7.51 – 7.47 (m, 2H), 7.42 – 7.22 (m, 28H), 5.94 – 5.89 (m, 1H), 5.68 – 5.63 (m, 1H), 5.59 (s, 1H), 4.83 – 4.77 (m, 3H), 4.68 (t, *J* = 12.6 Hz, 2H), 4.62 – 4.58 (m, 3H), 4.54 (s, 1H), 4.31 – 4.27 (m, 1H), 4.19 – 4.13 (m, 2H), 4.07 – 4.04 (m, 1H), 4.01 – 3.98 (m, 1H), 3.88 – 3.81 (m, 2H), 3.74 – 3.63 (m, 2H), 3.55 (dd, *J* = 9.9, 3.1 Hz, 1H), 3.23 (td, *J* = 9.8, 4.9 Hz, 1H), 2.38 – 2.33 (m, 1H). ^{13}C NMR (126 MHz, $CDCl_3$) δ 138.6, 138.4, 138.4, 138.1, 137.6, 129.0, 128.6, 128.5, 128.5, 128.3, 128.3, 128.3, 128.1, 127.8, 127.7, 127.7, 127.7, 127.7, 127.4, 126.2, 101.6, 101.5, 78.7, 78.1, 77.7, 77.4, 77.2, 76.9, 76.4, 74.8, 74.7, 73.5, 73.2, 72.6, 71.5, 68.6, 67.7, 64.2, 44.5. HRMS calculated for $[C_{48}H_{50}O_9 + Na]^+$: 793.3347, found 793.3347.

2,3-di-*O*-benzyl-4,6-*O*-benzylidene-(1-4)-β-D-manopyranosyl-2,3-di-*O*-benzyl-manno-cyclophellitol (**19**): Compound **18** (0.12 g, 0.15 mmol, 1 eq.) was dissolved in 1 mL DCM at 0 °C. mCPBA (0.053 g, 0.31 mmol, 2 eq.) was added and the reaction was left to stir for four days at 4 °C. The solvent was removed *in vacuo*, and the crude residue was re-dissolved in EtOAc, washed with H_2O and brine, filtered and concentrated *in vacuo*. The crude product was purified using column chromatography (EtOAc/pentane, 3/10 -> 1/2, v/v) to obtain compound **19** (0.98 g, 0.12 mmol, 83%). ^{1}H NMR (500 MHz, $CDCl_3$) δ 7.53 – 7.44 (m, 2H), 7.42 – 7.23 (m, 27H), 5.59 (s, 1H), 4.86 – 4.61 (m, 8H), 4.55 (d, *J* = 12.3 Hz, 1H), 4.42 (s, 1H), 4.17 – 4.08 (m, 3H), 3.95 (dd, *J* = 10.8, 6.5 Hz, 1H), 3.90 (t, *J* = 5.1 Hz, 1H), 3.86 – 3.78

(m, 4H), 3.56 (dd, J = 9.9, 3.0 Hz, 1H), 3.36 – 3.32 (m, 2H), 3.20 (td, J = 9.7, 4.9 Hz, 1H), 2.19 – 2.12 (m, 1H). ^{13}C NMR (126 MHz, $CDCl_3$) δ 138.4, 138.3, 138.3, 137.6, 133.6, 130.3, 129.9, 129.0, 128.5, 128.5, 128.4, 128.4, 128.3, 128.3, 128.3, 128.0, 128.0, 127.8, 127.8, 127.8, 127.7, 127.7, 126.2, 101.6, 101.5, 78.7, 78.3, 77.4, 77.2, 76.9, 76.5, 76.0, 75.8, 74.8, 73.8, 72.8, 72.7, 71.6, 68.6, 67.7, 62.6, 53.4, 52.0, 41.9. HRMS calculated for $[C_{48}H_{50}O_{10} + Na]^+$: 809.3296, found 809.3296.

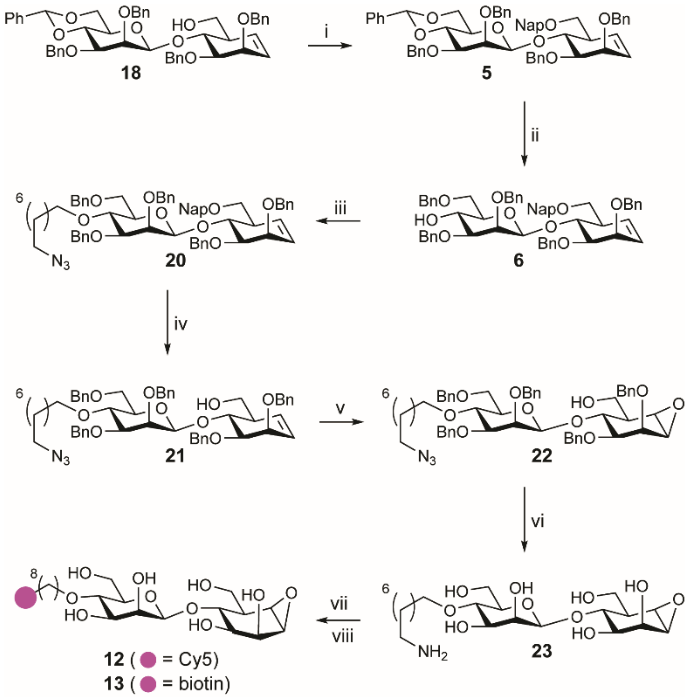

SI scheme 2. i) NapBr, NaH, TBAI, DMF, 71%. ii) i. CSA, MeOH, 50 °C. ii. 2-aminoethyl diphenylborinate, BnBr, KI, K_2CO_3, 74%. iii) 1-azido-8-iodooctane, NaH, DMF, 81%. iv) DDQ, DCM/H_2O, 69%. v) mCPBA, DCM, 0 °C, 89%. vi) Na, NH_3, *t*-BuOH, THF, -60 °C, 99%. vii) Cy5-COOH, pentrafluorophenyl trifluoroacetate, DIPEA, DMF, 5% **12**. viii) Biotin-TEG-COOH, pentrafluorophenyl trifluoroacetate, DIPEA, DMF, 10% **13**.

β-D-Mannose-(1-4)-manno-cyclophellitol (**15**): Compound **19** (0.028 g, 0.035 mmol, 1 eq.) was co-evaporated with anhydrous toluene thrice and dissolved in 1.45 mL anhydrous THF. *t*-BuOH (0.17 mL, 1.75 mmol, 50 eq.) was added to this solution. In a two-neck round bottom flask liquid ammonia was prepared fresh by cooling the round bottom flask to -60 °C while maintaining a gentle gaseous flow of ammonia in the flask. Upon reaching the desired amount of liquid ammonia the flask was sealed off and placed under nitrogen atmosphere. Solid sodium (0.040 g, 1.75 mmol, 50 eq.) was added to the liquid ammonia and the resulting solution turned blue. Compound **19** was added to the blue solution, and the reaction was left to stir for 30 minutes. The reaction was quenched with 1 mL AcOH/H_2O (1,75 mmol, 50 eq., 1/9, v/v) after which the blue colour disappeared. The reaction flask was transferred to a warm water bath and stirred for 30 minutes until all ammonia evaporated. The remaining solution was concentrated *in vacuo*. The crude residue was purified using gel filtration to obtain compound **15** (0.0084 g, 0.027 mmol, 71%). ^{1}H NMR (500 MHz, D_2O) δ 4.69 (s, 1H), 4.42 (t, J = 4.6, 3.6, 1.0 Hz, 1H), 4.05 (d, J = 3.2, 1.0 Hz, 1H), 3.94 – 3.89 (m, 2H), 3.87 – 3.80 (m, 1H), 3.77 – 3.70 (m, 3H), 3.64 (dd, J = 9.6, 3.2, 0.9 Hz, 1H), 3.60 – 3.49 (m, 3H), 3.42 – 3.36 (m, 1H), 2.30 – 2.24 (m, 1H). ^{13}C NMR (126 MHz, D_2O) δ 100.5, 76.5, 72.9, 71.1, 70.6, 66.7, 65.7, 61.0, 60.5, 56.2, 53.5, 42.9. HRMS calculated for $[C_{13}H_{22}O_{10} + Na]^+$: 361.1105, found 361.1105.

2,3-di-*O*-benzyl-4,6-*O*-benzylidene-(1-4)-β-D-manopyranosyl-2,3-di-*O*-benzyl-6-*O*-naphthyl-manno-cyclohexene (**5**): Compound **18** (0.27 g, 0.35 mmol, 1 eq.) was co-evaporated with anhydrous toluene thrice and dissolved in 3 mL anhydrous DMF. To this solution naphthyl bromide (0.12 g, 0.52 mmol, 1.5 eq.) and TBAI (0.006 g, 0.018 mmol, 0.05 eq.) were added. After the solution was cooled to 0 °C NaH (60% wt, 0.021 g, 0.52 mmol, 1.5 eq.) was added. The mixture was left to react for 2 hours and then

checked by TLC for full conversion. The reaction was quenched at 0 °C by the addition of H_2O followed by further dilution with H_2O and extraction of the aqueous layer with DCM thrice. The combined organic layers were washed with brine, dried of $MgSO_4$, filtered and concentrated *in vacuo*. The crude product was purified using flash column chromatography (EtOAc/pentane, 1/20 -> 2/5, v/v) to obtain compound **5** (0.23 g, 0.25 mmol, 71%). ^{1}H NMR (400 MHz, $CDCl_3$) δ 7.83 – 7.73 (m, 3H), 7.72 (s, 1H), 7.52 – 7.19 (m, 35H), 5.90 – 5.84 (m, 1H), 5.71 – 5.65 (m, 1H), 5.57 (s, 1H), 4.79 (q, 2H), 4.64 – 4.53 (m, 8H), 4.49 (d, *J* = 12.5 Hz, 1H), 4.29 – 4.24 (m, 1H), 4.24 – 4.18 (m, 1H), 4.20 – 4.08 (m, 2H), 4.02 – 3.98 (m, 1H), 3.86 – 3.76 (m, 2H), 3.61 (t, *J* = 8.7 Hz, 1H), 3.54 (dd, *J* = 9.0, 5.6 Hz, 1H), 3.45 (dd, *J* = 9.9, 3.0 Hz, 1H), 3.22 (td, *J* = 9.7, 4.8 Hz, 1H), 2.50 – 2.42 (m, 1H). ^{13}C NMR (101 MHz, $CDCl_3$) δ 138.8, 138.4, 138.4, 137.7, 135.8, 133.3, 133.1, 129.0, 128.5, 128.4, 128.4, 128.3, 128.3, 128.2, 127.9, 127.9, 127.6, 127.6, 127.5, 127.3, 127.3, 126.6, 126.4, 126.2, 126.1, 125.9, 101.5, 101.3, 78.7, 78.2, 76.8, 76.5, 75.4, 75.0, 74.8, 73.4, 73.2, 72.7, 72.4, 71.3, 71.3, 68.7, 67.6, 42.7. HRMS calculated for $[C_{59}H_{58}O_9 + Na]^+$: 933.3973, found 933.3973.

2,3,6-tri-*O*-benzyl-(1-4)-β-D-manopyranosyl-2,3-di-*O*-benzyl-6-*O*-naphthyl-manno-cyclohexene (**6**): Compound **5** (0.40 g, 0.43 mmol, 1 eq.) was dissolved in 4 mL MeOH and heated to 50 °C. CSA (0.050 g, 0.22 mmol, 0.5 eq.) was added and the reaction was left to stir for 1 hour. After TLC showed full conversion, the reaction was cooled in an ice-bath and quenched with Et_3N. The solvent was removed *in vacuo*, and the crude residue was re-dissolved in EtOAc, washed with brine, dried over $MgSO_4$, filtered and concentrated *in vacuo*. The residue was then co-evaporated with dry toluene thrice and dissolved in 1.5 mL dry ACN. To this solution was added Taylor's catalyst (0.030 g, 0.13 mmol, 0.3 eq.), benzyl bromide (0.077 mL, 0.65 mmol, 1.5 eq.), KI (0.078 g, 0.47 mmol, 1.1 eq.) and K_2CO_3 (0.065 g, 0.47 mmol, 1.1 eq.). The reaction mixture was heated to 65 °C and left to stir overnight. Solvent was removed *in vacuo*, and the crude residue was re-dissolved in EtOAc, washed with H_2O and brine, dried over $MgSO_4$, filtered and concentrated *in vacuo*. The crude residue was purified using flash column chromatography (EtOAc/pentane, 1/10 -> 3/10, v/v) to obtain compound **6** (0.31 g, 0.34 mmol, 79%). ^{1}H NMR (400 MHz, $CDCl_3$) δ 7.80 – 7.75 (m, 3H), 7.71 (s, 1H), 7.51 – 7.40 (m, 3H), 7.38 – 7.14 (m, 25H), 5.89 – 5.80 (m, 1H), 5.73 – 5.65 (m, 1H), 4.79 (d, *J* = 12.3 Hz, 1H), 4.69 – 4.47 (m, 11H), 4.41 (d, *J* = 11.8 Hz, 1H), 4.32 – 4.22 (m, 3H), 4.16 – 4.09 (m, 1H), 3.94 (d, *J* = 9.4 Hz, 1H), 3.80 – 3.68 (m, 3H), 3.64 – 3.51 (m, 2H), 3.36 (dt, *J* = 9.6, 4.8 Hz, 1H), 3.20 (dd, *J* = 9.4, 2.9 Hz, 1H), 2.68 (s, 1H), 2.54 – 2.48 (m, 1H). ^{13}C NMR (101 MHz, $CDCl_3$) δ 138.8, 138.8, 138.7, 138.1, 137.9, 135.8, 133.3, 133.0, 128.5, 128.4, 128.3, 128.3, 128.2, 128.2, 128.1, 127.9, 127.9, 127.9, 127.8, 127.7, 127.7, 127.6, 127.5, 127.4, 127.3, 127.2, 126.6, 126.3, 126.0, 125.9, 100.9, 81.6, 76.8, 75.3, 75.1, 74.7, 74.0, 73.9, 73.8, 73.4, 73.2, 72.7, 71.5, 71.3, 71.1, 70.9, 68.4, 42.7. HRMS calculated for $[C_{59}H_{60}O_9 + NH_4]^+$: 930.4576, found 930.4568.

4-*O*-(8-azidooctyl)-2,3,6-tri-*O*-benzyl-(1-4)-β-D-manopyranosyl-2,3-di-*O*-benzyl-6-*O*-naphthyl-manno-cyclohexene (**20**): Compound **6** (0.31 g, 0.34 mmol, 1 eq.) was co-evaporated with toluene thrice and dissolved in 3 mL dry DMF. To this solution was added 8-azidooctan-1-ol (0.36 g, 1.36 mmol, 4 eq.) and the resulting mixture was cooled to 0 °C. Sodium hydride (0.02 g, 0.51 mmol, 1.5 eq.) was added and the mixture was left to stir for 3 hours. TLC analysis (EtOAc/pentane, 3/20, v/v) showed the presence of starting material. Sodium hydride (0.014 g, 0.34 mmol, 1 eq.) and 8-azidooctan-1-ol (0.18 g, 0.68 mmol, 2 eq.) were added. After one hour TLC showed full conversion and the reaction mixture was quenched with H_2O. The resulting mixture was extracted with Et_2O thrice, the organic layers were combined, washed with H_2O five times and brine, dried over $MgSO_4$, filtered and concentrated *in vacuo*. The crude product was purified using flash column chromatography (EtOAc/pentane, 3/20 -> 3/10) to obtain compound **20** (0.34 g, 0.32 mmol, 93%). ^{1}H NMR (400 MHz, $CDCl_3$) δ 7.81 – 7.75 (m, 3H), 7.71 (s, 1H), 7.48 – 7.41 (m, 3H), 7.36 – 7.15 (m, 25H), 5.86 – 5.80 (m, 1H), 5.70 – 5.64 (m, 1H), 4.77 (q, 2H), 4.64 – 4.36 (m, 11H), 4.30 – 4.26 (m, 1H), 4.26 – 4.21 (m, 1H), 4.18 – 4.14 (m, 1H), 3.87 – 3.80 (m, 1H), 3.78 – 3.70 (m, 2H), 3.71 – 3.62 (m, 2H), 3.62 – 3.57 (m, 1H), 3.56 – 3.49 (m, 1H), 3.49 – 3.43 (m, 1H), 3.38 – 3.30 (m, 2H), 3.21 (t, *J* = 6.9 Hz, 2H), 2.53 – 2.46 (m, 1H), 1.59 – 1.52 (m, 2H), 1.52 – 1.43 (m, 2H), 1.36 – 1.21 (m, 8H). ^{13}C NMR (101 MHz, $CDCl_3$) δ 138.9, 138.9, 138.8, 138.6, 138.4, 135.9,

133.3, 133.0, 129.1, 128.5, 128.4, 128.3, 128.3, 128.2, 128.1, 128.1, 127.9, 127.8, 127.6, 127.5, 127.4, 127.4, 127.3, 127.1, 126.9, 126.6, 126.2, 126.0, 125.9, 101.0, 82.2, 77.5, 77.4, 77.2, 76.8, 76.2, 75.2, 74.9, 74.7, 74.4, 73.8, 73.6, 73.3, 73.2, 72.7, 71.5, 71.5, 71.0, 69.7, 58.2, 54.8, 51.5, 42.7, 30.4, 29.8, 29.4, 29.2, 28.9, 26.7, 26.1. HRMS calculated for $[C_{67}H_{75}N_3O_9 + Na]^+$: 1088.5396, found 1088.5396.

4-*O*-(8-azidooctyl)-2,3,6-tri-*O*-benzyl-(1-4)-β-D-manopyranosyl-2,3-di-*O*-benzyl-manno-cyclohexene (**21**): Compound **20** (0.25 g, 0.23 mmol, 1 eq.) was dissolved in a mixture of DCM/H_2O (18/1, 0.1M) and cooled to 0 °C. DDQ (0.075 g, 0.33 mmol, 1.4 eq.) was added and the reaction was left to stir at RT. After two hours TLC showed full conversion and the reaction was diluted with DCM, transferred to a separatory funnel and washed with 2M NaOH thrice and brine. The organic layer was dried over $MgSO_4$, filtered and concentrated *in vacuo*. The crude product was purified using flash column chromatography (EtOAc/pentane, 1/10 -> 3/10, v/v) to obtain compound **21** (0.15 g, 0.16 mmol, 69%). ^{1}H NMR (400 MHz, $CDCl_3$) δ 7.37 – 7.16 (m, 25H), 5.88 – 5.82 (m, 1H), 5.66 – 5.60 (m, 1H), 4.82 – 4.70 (m, 3H), 4.65 – 4.42 (m, 8H), 4.32 – 4.26 (m, 1H), 4.24 – 4.19 (m, 1H), 4.01 – 3.95 (m, 1H), 3.87 – 3.58 (m, 7H), 3.51 – 3.42 (m, 1H), 3.38 – 3.31 (m, 2H), 3.23 (t, *J* = 6.9 Hz, 2H), 2.95 (s, 1H), 2.39 – 2.34 (m, 1H), 1.61 – 1.42 (m, 4H), 1.36 – 1.17 (m, 8H). ^{13}C NMR (101 MHz, $CDCl_3$) δ 138.7, 138.7, 138.3, 138.2, 128.5, 128.4, 128.4, 128.4, 128.2, 127.9, 127.9, 127.7, 127.6, 127.5, 127.4, 127.4, 100.9, 82.1, 77.4, 76.8, 76.0, 75.0, 74.5, 74.4, 73.8, 73.7, 73.4, 73.1, 71.7, 71.2, 69.8, 64.2, 51.5, 44.5, 30.4, 29.4, 29.2, 28.9, 26.7, 26.1. HRMS calculated for $[C_{56}H_{67}N_3O_9 + NH_4]^+$: 943.5216, found 943.5206.

4-*O*-(8-azidooctyl)-2,3,6-tri-*O*-benzyl-(1-4)-β-D-manopyranosyl-2,3-di-*O*-benzyl-manno-cyclophellitol (**22**): Compound **21** (0.12 g, 0.12 mmol, 1 eq.) was dissolved in 1.2 mL DCM and cooled in an ice bath. mCPBA (0.043 g, 0.25 mmol, 2 eq.) was added to the solution and the reaction mixture was left to stir overnight. After TLC showed full conversion of the starting material the solvent was removed *in vacuo*. The crude residue was re-dissolved in EtOAc, washed with sat. aq. $NaHCO_3$, brine, dried over $MgSO_4$, filtered and concentrated *in vacuo*. The crude product was purified using flash column chromatography (EtOAc/pentane, 3/10 -> 1/2, v/v) to obtain compound **22** (0.10 g, 0.11 mmol, 88%). ^{1}H NMR (400 MHz, $CDCl_3$) δ 7.42 – 7.22 (m, 25H), 4.81 (s, 2H), 4.73 – 4.46 (m, 8H), 4.37 (s, 1H), 4.16 – 4.13 (m, 1H), 4.09 (t, *J* = 5.1 Hz, 1H), 3.95 (dd, *J* = 10.8, 6.6 Hz, 1H), 3.91 – 3.63 (m, 7H), 3.54 – 3.46 (m, 1H), 3.42 – 3.34 (m, 3H), 3.34 – 3.23 (m, 3H), 2.25 – 2.17 (m, 1H), 1.65 – 1.57 (m, 2H), 1.55 – 1.47 (m, 2H), 1.40 – 1.22 (m, 8H). ^{13}C NMR (101 MHz, $CDCl_3$) δ 138.8, 138.46, 138.36, 138.26, 138.21, 128.5, 128.5, 128.4, 128.4, 128.2, 128.0, 127.9, 127.9, 127.9, 127.7, 127.7, 127.6, 127.5, 100.8, 82.2, 76.0, 75.9, 75.6, 75.0, 74.7, 73.9, 73.7, 73.4, 72.5, 71.8, 71.3, 69.8, 62.6, 53.4, 52.1, 51.5, 42.2, 30.4, 29.4, 29.2, 28.9, 26.8, 26.1. HRMS calculated for $[C_{56}H_{67}N_3O_{10} + NH_4]^+$: 959.5165, found 959.5157.

4-*O*-(8-aminooctyl)-β-D-mannose-(1-4)-manno-cyclophellitol (**23**): Compound **22** (0.076 g, 0.081 mmol, 1 eq.) was co-evaporated with anhydrous toluene thrice and dissolved in 3.38 mL anhydrous THF. *t*-BuOH (0.46 mL, 4.83 mmol, 60 eq.) was added to this solution. In a two-neck round bottom flask liquid ammonia was prepared fresh by cooling the round bottom flask to -60 °C while maintaining a gentle gaseous flow of ammonia in the flask. Upon reaching the desired amount of liquid ammonia the flask was sealed off and placed under nitrogen atmosphere. Solid sodium (0.11 g, 4.83 mmol, 60 eq.) was added and the solution turned blue. To this solution was added the solution containing compound **22** and the reaction was left to stir for 30 minutes. The reaction was quenched with 0.48 mL AcOH/H_2O (10 mmol/mL) after which the blue colour disappeared. The reaction flask was transferred to a warm water bath and stirred for 30 minutes. The remaining solvents were removed *in vacuo*. The crude residue was purified using gel filtration twice to obtain compound **23** as the acetate salt (0.042 g, 0.080 mmol, 99%). ^{1}H NMR (400 MHz, D_2O) δ 4.71 (s, 1H), 4.47 (t, *J* = 4.4 Hz, 1H), 4.08 (d, *J* = 3.3 Hz, 1H), 4.01 – 3.92 (m, 2H), 3.92 – 3.73 (m, 6H), 3.72 – 3.61 (m, 2H), 3.56 (t, *J* = 4.2 Hz, 1H), 3.49 – 3.39 (m, 2H), 3.03 (t, *J* = 7.6 Hz, 2H), 2.35 – 2.29 (m, 1H), 1.76 – 1.57 (m, 4H), 1.47 – 1.34 (m, 8H). ^{13}C NMR (101 MHz, D_2O) δ 100.4, 76.3, 75.4, 75.2, 73.3, 72.8, 71.0, 70.8, 65.6, 60.7, 60.4, 56.1, 53.4, 42.8, 39.5, 29.2, 28.2, 28.0, 26.6, 25.4, 25.0. HRMS calculated for $[C_{21}H_{39}NO_{10} + H]^+$: 466.2647, found 466.2647.

4-*O*-(8-Cy5-octyl)-β-D-mannose-(1-4)-manno-cyclophellitol (**12**): Cy5-COOH (0.024 g ,0.050 mmol, 1 eq.) was dissolved in 0.2 mL dry DMF. PFP-TFA (0.021 mL, 0.13 mmol, 2.5 eq.) and DIPEA (0.043 mL, 0.25 mmol, 5 eq.) were added to the solution and the resulting mixture was stirred at RT. The reaction was analysed with LC-MS (reversed phase, ACN/H_2O/1% TFA, 1/10 -> 9/10) every 30 minutes. After LC-MS analysis confirmed the absence of starting material the reaction was quenched by adding H_2O (9 µL, 0.50 mmol, 10 eq.). After 15 minutes compound **22** (12.7 mg, 0.024 mmol, 0.5 eq.) and DIPEA (0.043 mL, 0.25 mmol, 5 eq.) were added. The reaction was left to stir until LC-MS showed full conversion of the starting material. Subsequently the mixture was concentrated *in vacuo* and purified using HPLC to obtain compound **12** (1.10 mg, 0.0012 mmol, 5%). ^{1}H NMR (850 MHz, D_2O) δ 7.96 (t, *J* = 13.0 Hz, 2H), 7.48 (d, *J* = 7.3 Hz, 1H), 7.41 – 7.34 (m, 2H), 7.26 – 7.22 (m, 2H), 6.50 (t, *J* = 12.4 Hz, 2H), 6.20 (d, *J* = 13.2 Hz, 2H), 4.56 (s, 1H), 4.38 (t, *J* = 4.5 Hz, 1H), 4.06 (t, *J* = 6.5 Hz, 2H), 3.96 (d, *J* = 3.3 Hz, 1H), 3.87 (dd, *J* = 11.2, 4.3 Hz, 2H), 3.80 (dd, *J* = 11.2, 7.3 Hz, 1H), 3.76 (dd, *J* = 12.4, 2.2 Hz, 1H), 3.69 – 3.61 (m, 3H), 3.61 – 3.56 (m, 2H), 3.55 – 3.51 (m, 3H), 3.47 (t, *J* = 4.3 Hz, 1H), 3.41 – 3.36 (m, 2H), 3.29 (t, *J* = 9.8 Hz, 2H), 3.24 – 3.21 (m, 1H), 2.97 (t, *J* = 7.2 Hz, 2H), 2.22 (s, 1H), 2.15 (t, *J* = 6.8 Hz, 2H), 1.87 (s, 6H), 1.83 – 1.79 (m, 2H), 1.62 – 1.60 (m, 3H), 1.59 – 1.55 (m, 2H), 1.40 – 1.34 (m, 2H), 1.33 – 1.23 (m, 4H), 1.18 – 1.10 (m, 8H). ^{13}C NMR (214 MHz, D_2O) δ 182.5, 177.1, 154.2, 154.2, 143.8, 143.1, 129.9, 126.5, 123.2, 123.1, 112.0, 111.6, 104.6, 101.3, 77.3, 76.4, 76.0, 74.2, 73.7, 72.0, 71.7, 69.7, 66.6, 61.6, 61.4, 57.1, 54.4, 43.8, 40.3, 36.3, 30.2, 29.5, 29.2, 29.1, 27.8, 27.7, 27.1, 26.2, 26.1, 25.9, 24.2. HRMS calculated for $[C_{53}H_{76}N_3O_{11}]^+$: 930.5474, found 930.5474.

4-*O*-(8-Cy5-octyl)-β-D-mannose-(1-4)-manno-cyclophellitol (**13**): PEG-Biotin (0.011 g, 0.025 mmol, 1 eq.) was dissolved in 0.2 mL dry DMF. PFP-TFA (0.011 mL, 0.0625 mmol, 2.5 eq.) and DIPEA (0.021 mL, 0.13 mmol, 5 eq.) were added to the solution and the resulting mixture was stirred at RT. The reaction was analysed with LC-MS every 30 minutes. After LC-MS analysis confirmed the absence of starting material the reaction was quenched by adding H_2O (5.6 µL, 0.31 mmol, 12.5 eq.). After 15 minutes compound **22** (6.3 mg, 0.012 mmol, 0.5 eq.) and DIPEA (0.021 mL, 0.13 mmol, 5 eq.) were added. The reaction was left to stir until LC-MS showed full conversion of the starting material. Subsequently the mixture was concentrated *in vacuo* and purified using HPLC to obtain compound **13** (1.1 mg, 0.0012 mmol, 10%). ^{1}H NMR (850 MHz, D_2O) δ 4.62 (s, 1H), 4.57 (dd, 1H), 4.39 – 4.37 (m, 2H), 4.03 (s, 2H), 3.99 (d, 1H), 3.89 – 3.85 (m, 2H), 3.80 (dd, *J* = 11.2, 7.3 Hz, 1H), 3.79 – 3.74 (m, 1H), 3.72 – 3.65 (m, 12H), 3.61 – 3.54 (m, 3H), 3.53 (dd, *J* = 4.0, 2.2 Hz, 1H), 3.47 (t, *J* = 4.3 Hz, 1H), 3.40 – 3.31 (m, 4H), 3.29 (dt, *J* = 9.8, 5.1 Hz, 1H), 3.20 (t, *J* = 7.0 Hz, 2H), 2.95 (dd, *J* = 13.1, 5.0 Hz, 1H), 2.74 (d, *J* = 13.0 Hz, 1H), 2.23 (t, *J* = 7.3 Hz, 3H), 1.72 – 1.66 (m, 2H), 1.65 – 1.46 (m, 4H), 1.40 – 1.34 (m, 4H), 1.31 – 1.25 (m, 8H). ^{13}C NMR (214 MHz, D2O) δ 177.8, 173.1, 166.3, 101.3, 77.3, 76.4, 76.1, 74.4, 73.7, 72.0, 71.8, 71.3, 70.6, 70.5, 70.4, 70.3, 69.8, 66.6, 63.0, 61.6, 61.4, 61.2, 57.1, 56.3, 54.4, 43.8, 40.6, 39.9, 39.8, 36.4, 30.2, 29.4, 29.3, 29.2, 28.8, 28.6, 26.9, 26.1, 26.1. HRMS calculated for $[C_{39}H_{68}N_4O_{16}S + H]^+$: 881.4424, found 881.4424.

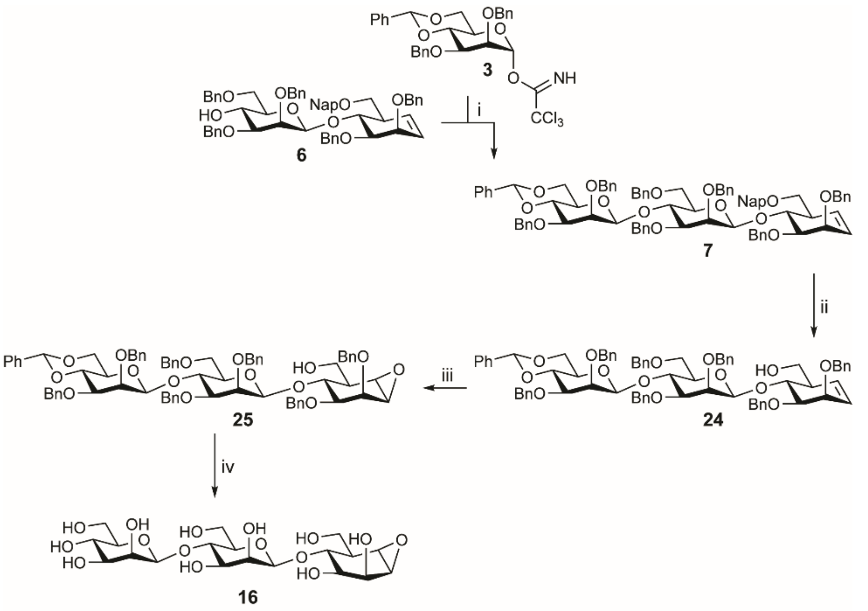

SI scheme 3. i) TMSOTf, DCM, -45 °C, 99%. ii) DDQ, DCM/H_2O, 0 °C, 55%. iii) mCPBA, DCM 4 °C, 91%. iv) Na, NH_3, *t*-BuOH, THF, -60 °C, 44%.

2,3-di-*O*-benzyl-4,6-*O*-benzylidene-(1-4)-β-D-mannopyranosyl-2,3,6-tri-*O*-benzyl-(1-4)-β-D-mannopyranosyl-2,3-di-*O*-benzyl-6-*O*-naphthyl-manno-cyclohexene (**7**): Donor **3** (0.32 g, 0.53 mmol, 2.0 eq.) and acceptor **6** (0.24 g, 0.27 mmol, 1 eq.) were co-evaporated with anhydrous toluene thrice under an argon atmosphere. The concentrated mixture was dissolved in 4 mL anhydrous DCM under an argon atmosphere. After cooling the solution down to -45 °C TMSOTf (9.26 µL, 0.053 mmol, 0.2 eq.) was added and the reaction was left to stir for 30 minutes at -45 °C. Subsequently the reaction was quenched with sat. aq. $NaHCO_3$, the water layer re-extracted with DCM twice, organic layers combined and washed with brine, dried over $MgSO_4$, filtered and concentrated *in vacuo*. The crude product was purified using flash silica column chromatography (EtOAc/pentane, 1/20 -> 3/20, v/v) followed by a SEC (Sephadex-LH20, MeOH/DCM, 1:1, v/v) to remove any traces of acceptor to obtain compound **7** as colourless oil (0.36 g, 0.26 mmol, 99%). ^{1}H NMR (400 MHz, $CDCl_3$) δ 7.81 – 7.71 (m, 4H), 7.48 – 7.15 (m, 43H), 5.83 (dt, *J* = 10.5, 2.4 Hz, 1H), 5.68 (dt, *J* = 10.3, 2.5 Hz, 1H), 5.49 (s, 1H), 4.83 (d, *J* = 11.9 Hz, 1H), 4.79 – 4.71 (m, 3H), 4.71 – 4.64 (m, 2H), 4.64 – 4.51 (m, 9H), 4.51 – 4.40 (m, 3H), 4.32 – 4.22 (m, 3H), 4.17 (t, *J* = 9.3 Hz, 1H), 4.11 – 4.04 (m, 2H), 4.00 (dd, *J* = 10.4, 4.8 Hz, 1H), 3.77 (dd, *J* = 6.3, 3.0 Hz, 2H), 3.66 – 3.51 (m, 4H), 3.45 – 3.39 (m, 2H), 3.36 – 3.30 (m, 1H), 3.06 (td, *J* = 9.7, 4.8 Hz, 1H), 2.55 – 2.49 (m, 1H). ^{13}C NMR (101 MHz, $CDCl_3$) δ 138.9, 138.9, 138.9, 138.8, 138.6, 138.3, 137.7, 135.8, 133.3, 133.0, 128.9, 128.4, 128.4, 128.3, 128.3, 128.2, 128.2, 128.1, 128.1, 127.9, 127.8, 127.8, 127.7, 127.6, 127.5, 127.5, 127.4, 127.3, 127.1, 126.6, 126.3, 126.2, 126.0, 125.9, 102.0, 101.4, 101.0, 80.4, 78.7, 78.5, 77.3, 75.7, 75.5, 75.3, 75.0, 74.8, 74.0, 73.6, 73.3, 73.2, 72.6, 72.5, 72.0, 71.4, 71.1, 69.4, 68.6, 67.3, 42.8. HRMS calculated for $[C_{86}H_{86}O_{14} + NH_4]^+$: 1360.6356, found 1360.6347.

2,3-di-*O*-benzyl-4,6-*O*-benzylidene-(1-4)-β-D-mannopyranosyl-2,3,6-tri-*O*-benzyl-(1-4)-β-D-mannopyranosyl-2,3-di-*O*-benzyl-manno-cyclohexene (**24**): Compound **7** (0.090 g, 0.067 mmol, 1 eq.) was dissolved in a mixture of DCM/H_2O (18/1, 0.1M) and cooled to 0 °C. DDQ (0.030 g, 0.13 mmol, 2 eq.) was added and the reaction was left to stir at RT. After two hours TLC showed full conversion and the reaction was diluted with DCM, transferred to a separatory funnel and washed with 2M NaOH thrice and brine, dried over $MgSO_4$, filtered and concentrated *in vacuo*. The crude product was purified using flash column chromatography (EtOAc/pentane, 1/5 -> 2/5, v/v) followed by a SEC (Sephadex-LH20, MeOH/DCM, 1:1, v/v) to obtain compound **24** (0.044 g, 0.037 mmol, 55%). ^{1}H NMR (400 MHz, $CDCl_3$) δ 7.47 – 7.11 (m, 40H), 5.91 – 5.80 (m, 1H), 5.67 – 5.58 (m, 1H), 5.52 (s, 1H), 4.86 – 4.49 (m, 14H), 4.35 (d, *J* = 11.9 Hz, 1H), 4.27 – 4.22 (m, 1H), 4.18 – 3.97 (m, 5H), 3.87 – 3.75 (m, 2H), 3.75 – 3.58 (m, 5H), 3.52 (dd, *J* = 8.8, 3.1 Hz, 1H), 3.48 – 3.35 (m, 2H), 3.13 – 3.03 (m, 1H), 2.89 (s, 1H), 2.40 (dt, *J* =

5.8, 3.0 Hz, 1H). ^{13}C NMR (126 MHz, $CDCl_3$) δ 139.0, 138.9, 138.8, 138.8, 138.7, 138.6, 138.5, 138.4, 138.3, 138.2, 138.1, 137.9, 137.8, 137.7, 128.9, 128.9, 128.8, 128.7, 128.6, 128.6, 128.5, 128.5, 128.5, 128.5, 128.4, 128.4, 128.4, 128.3, 128.3, 128.2, 128.2, 128.2, 128.2, 128.0, 128.0, 128.0, 127.9, 127.9, 127.8, 127.7, 127.7, 127.6, 127.6, 127.6, 127.6, 127.5, 127.5, 127.5, 127.3, 127.3, 127.2, 126.2, 126.2, 126.2, 102.1, 101.5, 101.4, 101.1, 100.7, 82.4, 80.0, 79.1, 78.8, 78.5, 77.6, 77.5, 77.4, 76.4, 75.7, 75.4, 75.3, 75.2, 75.1, 75.0, 74.3, 73.9, 73.9, 73.7, 73.6, 73.6, 73.4, 73.3, 73.1, 73.1, 72.7, 72.2, 71.4, 71.3, 70.9, 70.2, 69.7, 68.8, 68.7, 67.5, 65.2, 64.5, 64.3, 45.0, 44.7, 29.8. HRMS calculated for $[C_{75}H_{78}O_{14} + NH_4]^+$: 1220.5730, found 1220.5726.

2,3-di-*O*-benzyl-4,6-*O*-benzylidene-(1-4)-β-D-mannopyranosyl-2,3,6-tri-*O*-benzyl-(1-4)-β-D-mannopyranosyl-2,3-di-*O*-benzyl-manno-cyclophellitol (**25**): Compound **24** (0.040 g, 0.033 mmol, 1 eq.) was dissolved in 0.33 mL DCM and cooled in an ice-bath. mCPBA (0.011 g, 0.066 mmol, 2 eq.) was added to the solution and the reaction mixture was left to stir overnight at 4 °C. After TLC showed full conversion of the starting material the solvent was removed *in vacuo*. The crude residue was re-dissolved in EtOAc, washed with sat. aq. $NaHCO_3$, brine, dried over $MgSO_4$, filtered and concentrated *in vacuo.* The crude product was purified using flash column chromatography (EtOAc/pentane, 2/5 -> 3/5, v/v) to obtain compound **25** (0.037 g, 0.030 mmol, 91%). ^1H NMR (500 MHz, $CDCl_3$) δ 7.53 – 7.10 (m, 51H), 5.51 (s, 1H), 4.87 – 4.42 (m, 18H), 4.34 (d, *J* = 11.8 Hz, 1H), 4.17 – 3.97 (m, 5H), 3.94 – 3.73 (m, 8H), 3.68 – 3.54 (m, 3H), 3.51 (dd, *J* = 8.8, 3.0 Hz, 1H), 3.45 (dd, *J* = 9.9, 3.0 Hz, 1H), 3.37 (ddd, *J* = 7.8, 4.7, 2.8 Hz, 1H), 3.31 (t, *J* = 3.9 Hz, 1H), 3.25 (t, 1H), 3.08 (td, *J* = 9.7, 4.9 Hz, 1H), 2.22 – 2.14 (m, 1H). ^{13}C NMR (126 MHz, $CDCl_3$) δ 138.8, 138.7, 138.5, 138.3, 138.1, 137.7, 128.9, 128.5, 128.5, 128.4, 128.4, 128.3, 128.3, 128.2, 128.2, 128.2, 127.9, 127.9, 127.8, 127.8, 127.6, 127.6, 127.6, 127.5, 127.5, 127.3, 126.2, 102.0, 101.5, 100.7, 78.8, 78.5, 77.3, 76.7, 75.6, 75.5, 75.3, 75.1, 73.9, 73.7, 73.5, 72.7, 72.4, 72.4, 71.4, 69.6, 68.7, 67.4, 62.9, 53.8, 51.8, 42.6. HRMS calculated for $[C_{75}H_{78}O_{15} + NH_4]^+$: 1236.5679, found 1236.5771.

β-D-Mannose-(1-4)-β-D-mannose-(1-4)-manno-cyclophellitol (**16**): Compound **24** (0.035 g, 0.029 mmol, 1 eq.) was co-evaporated with anhydrous toluene thrice and dissolved in 1.20 mL anhydrous THF. *t*-BuOH (0.22 mL, 2.30 mmol, 60 eq.) was added to this solution. In a two-neck round bottom flask liquid ammonia was prepared fresh by cooling the round bottom flask to -60 °C while maintaining a gentle gaseous flow of ammonia in the flask. Upon reaching the desired amount of liquid ammonia the flask was sealed off and placed under nitrogen atmosphere. Solid sodium (0.053 g, 2.30 mmol, 60 eq.) was added and the solution turned blue. To this solution was added the solution containing compound **24** and the reaction was left to stir for 30 minutes. The reaction was quenched with 0.23 mL AcOH/H_2O (10 mmol/mL) after which the blue colour disappeared. The reaction flask was transferred to a warm water bath and stirred for 30 minutes. The remaining solvents were removed *in vacuo.* The crude residue was purified using gel filtration twice to obtain compound **16** (0.0063 g, 0.013 mmol, 43.9%). ^1H NMR (850 MHz, D_2O) δ 4.69 (s, 1H), 4.67 (s, 1H), 4.38 (t, *J* = 4.6 Hz, 1H), 4.08 – 4.06 (m, 1H), 4.03 – 4.00 (m, 1H), 3.92 – 3.83 (m, 3H), 3.82 – 3.73 (m, 3H), 3.72 – 3.66 (m, 4H), 3.61 (dd, *J* = 9.7, 3.3 Hz, 1H), 3.54 – 3.51 (m, 2H), 3.49 – 3.46 (m, 2H), 3.40 (ddd, *J* = 9.6, 6.9, 2.3 Hz, 1H), 2.25 – 2.22 (m, 1H). ^{13}C NMR (214 MHz, D_2O) δ 101.3, 101.3, 101.1, 101.1, 77.5, 77.3, 77.3, 75.9, 73.6, 72.4, 71.9, 71.4, 71.4, 70.9, 70.9, 67.6, 66.6, 66.6, 61.9, 61.4, 57.1, 54.4, 43.7. HRMS calculated for $[C_{19}H_{32}O_{15} + NH_4]^+$: 518.2079, found 518.2080.

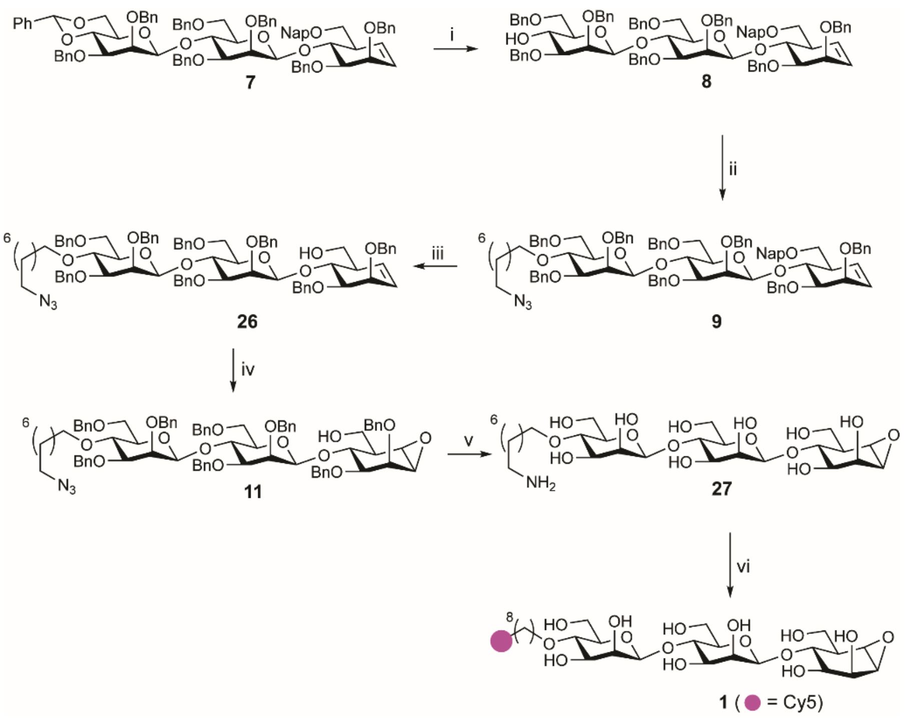

SI scheme 4. i) 1. CSA, MeOH, 50 °C. 2. 2-aminoethyl diphenylborinate, BnBr, KI, K_2CO_3, 81%. ii) 1-azido-8-iodooctane, NaH, DMF, 77%. iii) DDQ, DCM/H_2O, 83%. iv) mCPBA, DCM, 0 °C, 83%. v) Na, NH_3, *t*-BuOH, THF, -60 °C, 16%. vi) 1. Cy5-COOH, pentafluorophenyl trifluoroacetate, DIPEA, DMF. 2. 27, DIPEA, DMF, 99%.

2,3,6-tri-*O*-benzyl-(1-4)-β-D-mannopyranosyl-2,3,6-tri-*O*-benzyl-(1-4)-β-D-mannopyranosyl-2,3-di-*O*-benzyl-6-*O*-naphthyl-manno-cyclohexene (**8**): Compound **7** (0.48 g, 0.36 mmol, 1 eq.) was dissolved in 3.6 mL MeOH/DCE, v/v, 1/1 and heated to 50 °C. CSA (0.042 g, 0.18 mmol, 0.5 eq.) was added and the reaction was left to stir for 1 hour. After TLC showed full conversion, the reaction was cooled in an ice-bath and quenched with Et_3N. The solvent was removed *in vacuo*, and the crude residue was re-dissolved in EtOAc, washed with brine, dried over $MgSO_4$, filtered and concentrated *in vacuo.* The residue was then co-evaporated with dry toluene thrice and dissolved in 1.5 mL dry ACN. To this solution was added Taylor's catalyst (0.019 g, 0.086 mmol, 0.3 eq.), benzyl bromide (0.051 mL, 0.43 mmol, 1.5 eq.), KI (0.052 g, 0.32 mmol, 1.1 eq.) and K_2CO_3 (0.044 g, 0.32 mmol, 1.1 eq.). The reaction mixture was heated to 65 °C and left to stir overnight. Solvent was removed *in vacuo*, and the crude residue was re-dissolved in EtOAc, washed with H_2O and brine, dried over $MgSO_4$, filtered and concentrated in vacuo. The crude residue was purified using flash column chromatography (EtOAc/pentane, 1/10 -> 1/5, v/v) to obtain compound **8** (0.31 g, 0.39 mmol, 81%). ^1H NMR (400 MHz, $CDCl_3$) δ 7.83 – 7.68 (m, 4H), 7.46 – 7.11 (m, 43H), 5.85 – 5.77 (m, 1H), 5.71 – 5.61 (m, 1H), 4.80 – 4.72 (m, 3H), 4.68 (d, *J* = 12.2 Hz, 2H), 4.65 – 4.50 (m, 8H), 4.49 – 4.41 (m, 6H), 4.37 – 4.30 (m, 2H), 4.27 – 4.18 (m, 3H), 4.10 (ddd, *J* = 4.9, 3.8, 1.1 Hz, 1H), 3.92 (td, *J* = 9.4, 1.7 Hz, 1H), 3.77 (t, *J* = 3.1 Hz, 2H), 3.70 (dd, *J* = 11.2, 4.5 Hz, 1H), 3.65 – 3.39 (m, 7H), 3.23 – 3.13 (m, 2H), 2.55 – 2.46 (m, 1H). ^{13}C NMR (101 MHz, $CDCl_3$) δ 139.1, 139.0, 138.9, 138.8, 138.5, 138.2, 138.2, 135.9, 133.3, 133.1, 128.5, 128.4, 128.4, 128.3, 128.3, 128.2, 128.1, 127.9, 127.9, 127.8, 127.8, 127.8, 127.8, 127.7, 127.7, 127.6, 127.6, 127.5, 127.4, 127.4, 127.4, 127.3, 127.2, 126.6, 126.3, 126.0, 125.9, 101.3, 100.9, 81.9, 80.3, 75.5, 75.3, 75.0, 74.8, 74.8, 74.7, 74.3, 73.9, 73.7, 73.6, 73.4, 72.6, 72.1, 71.5, 71.5, 71.1, 71.1, 69.7, 68.7, 42.7. HRMS calculated for $[C_{86}H_{88}O_{14} + NH_4]^+$: 1362.6512, found 1362.6512.

4-*O*-(8-azidooctyl)-2,3,6-tri-*O*-benzyl-(1-4)-β-D-mannopyranosyl-2,3,6-tri-*O*-benzyl-(1-4)-β-D-mannopyranosyl-2,3-di-*O*-benzyl-6-*O*-naphthyl-manno-cyclohexene (**9**): Compound **8** (0.19 g, 0.14 mmol, 1 eq.) was co-evaporated with toluene thrice and dissolved in 1.39 mL dry DMF. To this solution was added 8-azidooctan-1-ol (0.19 g, 0.69 mmol, 6 eq.) and the resulting mixture was cooled to 0 °C. Sodium hydride (0.011 g, 0.46 mmol, 4 eq.) was added and the mixture was left to stir for 3 hours.

After TLC showed full conversion the reaction mixture was quenched with H_2O. The resulting mixture was extracted wit Et_2O thrice, the organic layers were combined, washed with H_2O five times and brine, dried over $MgSO_4$, filtered and concentrated *in vacuo*. The crude product was purified using flash column chromatography (EtOAc/pentane, 3/20 -> 3/10, v/v) to obtain compound **9** (0.16 g, 0.11 mmol, 77%). ^1H NMR (400 MHz, $CDCl_3$) δ 7.82 – 7.68 (m, 5H), 7.48 – 7.10 (m, 42H), 5.85 – 5.77 (m, 1H), 5.69 – 5.63 (m, 1H), 4.82 – 4.39 (m, 18H), 4.31 (d, *J* = 11.9 Hz, 1H), 4.27 – 4.17 (m, 3H), 4.10 (ddd, *J* = 5.2, 3.8, 1.1 Hz, 1H), 3.80 – 3.72 (m, 3H), 3.70 – 3.38 (m, 12H), 3.28 – 3.16 (m, 4H), 2.47 (dt, *J* = 5.6, 2.7 Hz, 1H), 1.60 – 1.53 (m, 2H), 1.46 – 1.38 (m, 2H), 1.35 – 1.18 (m, 8H). ^{13}C NMR (101 MHz, $CDCl_3$) δ 139.2, 139.0, 138.9, 138.8, 138.8, 138.5, 138.4, 135.8, 133.3, 133.0, 128.4, 128.3, 128.2, 128.2, 128.2, 128.1, 128.0, 127.9, 127.9, 127.8, 127.8, 127.7, 127.7, 127.5, 127.5, 127.4, 127.4, 127.3, 127.3, 127.2, 127.2, 127.1, 126.5, 126.2, 125.9, 125.8, 101.2, 100.8, 82.6, 80.2, 76.3, 75.5, 75.4, 75.3, 75.2, 74.9, 74.8, 74.6, 74.0, 73.8, 73.5, 73.5, 73.4, 73.3, 73.1, 72.5, 72.3, 71.6, 71.4, 71.0, 69.6, 69.5, 51.5, 42.6, 30.3, 29.4, 29.1, 28.8, 26.7, 26.0. HRMS calculated for $[C_{94}H_{103}N_3O_{14} + NH_4]^+$: 1515.7778, found 1515.7778.

4-*O*-(8-azidooctyl)-2,3,6-tri-*O*-benzyl-(1-4)-β-D-mannopyranosyl-2,3,6-tri-*O*-benzyl-(1-4)-β-D-mannopyranosyl-2,3-di-*O*-benzyl-manno-cyclophellitol-alkene (**26**): Compound **9** (0.063 g, 0.042 mmol, 1 eq.) was dissolved in a mixture of DCM/H_2O (18/1, 0.1M) and cooled to 0 °C. DDQ (0.19 g, 0.084 mmol, 2 eq.) was added and the reaction was left to stir at RT. After two hours TLC showed full conversion and the reaction was diluted with DCM, transferred to a separatory funnel, washed with 2M NaOH thrice and brine, dried over $MgSO_4$, filtered and concentrated *in vacuo*. The crude product was purified using flash column chromatography (EtOAc/pentane, 1/10 -> 2/5, v/v) followed by a SEC purification (Sephadex-LH20, MeOH/DCM, 1:1, v/v) to obtain compound **26** (0.048 g, 0.035 mmol, 83%). ^1H NMR (400 MHz, $CDCl_3$) δ 7.38 (dd, *J* = 6.7, 2.8 Hz, 2H), 7.35 – 7.15 (m, 38H), 5.84 (dt, *J* = 10.3, 2.7 Hz, 1H), 5.61 (dt, *J* = 10.4, 2.5 Hz, 1H), 4.85 – 4.40 (m, 18H), 4.37 (d, *J* = 11.7 Hz, 1H), 4.23 (dq, *J* = 4.2, 2.0 Hz, 1H), 4.17 (t, *J* = 8.7 Hz, 1H), 4.09 (dd, *J* = 6.1, 3.5 Hz, 1H), 4.00 (dd, *J* = 6.0, 3.1 Hz, 1H), 3.82 – 3.73 (m, 3H), 3.74 – 3.55 (m, 8H), 3.52 (ddd, *J* = 8.5, 5.0, 3.1 Hz, 1H), 3.44 (dt, *J* = 9.1, 6.7 Hz, 1H), 3.31 – 3.17 (m, 4H), 2.95 (s, 1H), 2.34 (dd, *J* = 5.9, 3.0 Hz, 1H), 1.55 (q, *J* = 7.2 Hz, 2H), 1.44 (d, *J* = 11.6 Hz, 2H), 1.24 (d, *J* = 9.6 Hz, 8H). ^{13}C NMR (101 MHz, $CDCl_3$) δ 139.2, 138.8, 138.7, 138.5, 138.3, 138.3, 128.5, 128.4, 128.4, 128.4, 128.3, 128.3, 128.2, 128.2, 128.1, 127.9, 127.8, 127.7, 127.7, 127.7, 127.6, 127.6, 127.5, 127.5, 127.4, 127.4, 127.3, 101.1, 100.5, 82.5, 77.6, 76.3, 75.4, 75.1, 74.9, 74.8, 74.1, 73.6, 73.6, 73.6, 73.3, 73.3, 73.1, 72.3, 71.7, 71.3, 70.0, 69.7, 64.5, 51.6, 44.9, 30.4, 29.8, 29.5, 29.2, 28.9, 26.8, 26.1. HRMS calculated for $[C_{83}H_{95}N_3O_{14} + NH_4]^+$: 1375.7152, found 1375.7152.

4-*O*-(8-azidooctyl)-2,3,6-tri-*O*-benzyl-(1-4)-β-D-mannopyranosyl-2,3,6-tri-*O*-benzyl-(1-4)-β-D-mannopyranosyl-2,3-di-*O*-benzyl-manno-cyclophellitol (**11**): Compound **25** (0.050 g, 0.037 mmol, 1 eq.) was dissolved in 0.37 mL DCM and cooled in an ice-bath. mCPBA (0.013 g, 0.074 mmol, 2 eq.) was added to the solution and the reaction mixture was left to stir overnight. After TLC showed full conversion of the starting material the solvent was removed in vacuo. The crude residue was re-dissolved in EtOAc, washed with sat. aq. $NaHCO_3$, brine, dried over $MgSO_4$, filtered and concentrated *in vacuo*. The crude product was purified using flash column chromatography (EtOAc/pentane, 3/10 -> 2/5, v/v) to obtain compound **11** (0.042 g, 0.031 mmol, 83%). ^1H NMR (400 MHz, $CDCl_3$) δ 7.39 – 7.35 (m, 2H), 7.35 – 7.15 (m, 38H), 4.83 – 4.69 (m, 3H), 4.68 – 4.61 (m, 3H), 4.57 – 4.43 (m, 11H), 4.35 (d, *J* = 11.8 Hz, 1H), 4.15 (t, *J* = 8.7 Hz, 1H), 4.05 (dd, *J* = 4.6, 2.8 Hz, 1H), 3.93 – 3.86 (m, 2H), 3.84 – 3.74 (m, 5H), 3.68 – 3.61 (m, 5H), 3.58 (dd, *J* = 8.7, 3.0 Hz, 1H), 3.52 – 3.41 (m, 2H), 3.30 – 3.27 (m, 2H), 3.27 – 3.20 (m, 5H), 2.16 – 2.10 (m, 1H), 1.62 – 1.50 (m, 3H), 1.51 – 1.41 (m, 2H), 1.34 – 1.19 (m, 8H). ^{13}C NMR (101 MHz, $CDCl_3$) δ 139.1, 138.8, 138.5, 138.5, 138.4, 138.2, 128.5, 128.5, 128.4, 128.4, 128.4, 128.3, 128.2, 128.2, 127.9, 127.9, 127.8, 127.8, 127.7, 127.6, 127.6, 127.5, 127.4, 127.4, 101.1, 100.4, 82.5, 76.5, 76.3, 75.5, 75.4, 75.3, 75.2, 74.9, 74.8, 74.1, 73.7, 73.6, 73.5, 73.3, 72.5, 72.5, 71.8, 71.4, 69.9, 69.7, 62.9, 53.7, 51.9, 51.6, 42.5, 30.4, 29.8, 29.5, 29.2, 28.9, 26.8, 26.1. HRMS calculated for $[C_{83}H_{95}N_3O_{15} + NH_4]^+$: 1391.7101, found 1391.7101.

4-*O*-(8-aminooctyl)-β-D-mannose-(1-4)-β-D-mannose-(1-4)-manno-cyclophellitol (**27**): Compound **11** (0.036 g, 0.026 mmol, 1 eq.) was co-evaporated with anhydrous toluene thrice and dissolved in 1 mL anhydrous THF. *t*-BuOH (0.23 mL, 2.36 mmol, 90 eq.) was added to this solution. In a two-neck round bottom flask liquid ammonia was prepared fresh by cooling the round bottom flask to -60 °C while maintaining a gentle gaseous flow of ammonia in the flask. Upon reaching the desired amount of liquid ammonia the flask was sealed off and placed under nitrogen atmosphere. Solid sodium (0.054 g, 2.36 mmol, 90 eq.) was added and the solution turned blue. To this solution was added the solution containing compound **11** and the reaction was left to stir for 15 minutes. The reaction was quenched with 0.23 mL AcOH/H_2O (10 mmol/mL) after which the blue colour disappeared. The reaction flask was transferred to a warm water bath and stirred for 30 minutes. The remaining solvents were removed *in vacuo*. The crude residue was purified using gel filtration twice to obtain compound **27** (2.9 mg, 4.62 µmol, 16%.). ^{1}H NMR (600 MHz, D_2O) δ 4.67 (dd, *J* = 7.8, 1.0 Hz, 2H), 4.38 (t, *J* = 4.5 Hz, 1H), 4.08 (d, *J* = 1.7 Hz, 1H), 4.00 (d, *J* = 2.8 Hz, 1H), 3.92 – 3.85 (m, 3H), 3.86 – 3.79 (m, 1H), 3.81 – 3.74 (m, 4H), 3.74 – 3.65 (m, 4H), 3.61 – 3.54 (m, 1H), 3.54 (dd, *J* = 4.1, 2.2 Hz, 1H), 3.50 – 3.45 (m, 2H), 3.41 – 3.36 (m, 2H), 2.94 (t, 2H), 2.26 – 2.20 (m, 1H), 1.61 (q, *J* = 7.6 Hz, 2H), 1.58 – 1.52 (m, 2H), 1.35 – 1.25 (m, 8H). ^{13}C NMR (151 MHz, D_2O) δ 101.3, 101.1, 77.4, 77.3, 76.4, 76.2, 75.9, 74.3, 73.6, 72.4, 72.0, 71.7, 70.9, 66.6, 61.7, 61.4, 61.4, 57.1, 54.4, 43.8, 40.4, 30.1, 29.1, 29.0, 27.6, 26.4, 26.0, 24.1. HRMS calculated for $[C_{27}H_{49}NO_{15} + H]^+$: 628.3175, found 628.3175.

4-*O*-(8-Cy5-octyl)-β-D-mannose-(1-4)-manno-cyclophellitol (**1**): Cy5-COOH (5.8 mg, 0.012 mmol, 1 eq.) was dissolved in 0.39 mL dry DMF. PFP-TFA (0.0062 mL, 0.036 mmol, 3 eq.) and DIPEA (0.021 mL, 0.12 mmol, 10 eq.) were added to the solution and the resulting mixture was stirred at RT. The reaction was analysed with LC-MS (reversed phase, ACN/H_2O/1% TFA, 1/10 -> 9/10) every 30 minutes. After LC-MS analysis confirmed the absence of starting material the reaction was quenched by adding H_2O (0.65 µL, 0.036 mmol, 3 eq.) and stirring for 15 minutes. From this reaction mixture 0.3 mL was added to an Eppendorf tube which contained freeze-dried compound **27** (2.9 mg, 4.62 µmol, 1 eq.). The reaction was left to stir until LC-MS showed full conversion of the starting material. Subsequently the mixture was concentrated *in vacuo* and purified using flash column chromatography (MeOH/DCM/AcOH, 1:1:0 -> 99:0:1, v/v) followed by a C_{18} column (ACN/H_2O, 1:5, v/v -> MeOH/AcOH, 99:1, v/v) to obtain compound **1** slightly impure (2.80 mg, 2.54 µmol, quant.). ^{1}H NMR (850 MHz, MeOD) δ 8.24 (td, J = 13.0, 3.1 Hz, 2H), 7.49 (d, J = 7.4 Hz, 2H), 7.44 – 7.39 (m, 2H), 7.32 – 7.25 (m, 4H), 6.63 (t, J = 12.4 Hz, 1H), 6.28 (dd, J = 13.7, 8.7 Hz, 2H), 4.60 – 4.57 (m, 1H), 4.32 – 4.30 (m, 1H), 4.11 (t, J = 7.5 Hz, 2H), 3.97 (d, J = 3.1, 0.9 Hz, 1H), 3.94 – 3.80 (m, 7H), 3.77 – 3.60 (m, 8H), 3.55 (dt, J = 9.2, 3.5 Hz, 1H), 3.53 – 3.49 (m, 1H), 3.47 – 3.44 (m, 1H), 3.37 – 3.33 (m, 2H), 3.28 – 3.24 (m, 1H), 3.12 (t, J = 7.2 Hz, 2H), 2.20 (t, J = 7.2 Hz, 2H), 2.16 (d, J = 7.2, 4.9, 2.4 Hz, 1H), 1.91 (s, 6H), 1.83 (p, J = 7.7 Hz, 2H), 1.73 (s, 12H), 1.59 – 1.48 (m, 3H), 1.48 – 1.43 (m, 4H), 1.34 (dd, J = 42.9, 5.2 Hz, 11H). ^{13}C NMR (214 MHz, MeOD) δ 175.7, 175.4, 174.7, 155.5, 144.3, 143.6, 142.7, 142.5, 129.8, 129.8, 126.6, 126.3, 126.3, 123.4, 123.3, 112.1, 111.9, 104.4, 104.3, 102.1, 101.9, 101.7, 77.8, 77.2, 77.1, 76.9, 76.6, 75.3, 74.7, 74.0, 74.0, 73.5, 73.2, 72.9, 72.8, 71.9, 71.9, 67.3, 67.3, 62.6, 62.3, 62.3, 61.9, 56.4, 54.8, 50.6, 50.5, 45.1, 44.8, 40.4, 36.7, 31.5, 31.4, 30.5, 30.4, 30.3, 28.2, 28.0, 27.8, 27.8, 27.4, 27.1, 26.5. HRMS calculated for $[C_{59}H_{86}N_3O_{16}]^+$: 1092.6003, found 1092.6003.

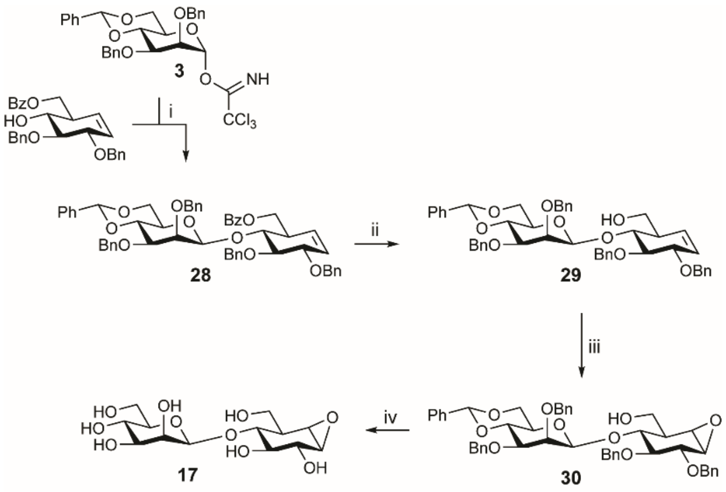

SI scheme 5. i) TMSOTf, DCM, -45 °C, 78%. ii) NaOMe, MeOH/DCM, 88%. iii) mCPBA, DCM 4 °C, 46%. iv) Na, NH_3, *t*-BuOH, THF, -60 °C, 8%.

2,3-di-*O*-benzyl-4,6-*O*-benzylidene-(1-4)-β-D-mannopyranosyl-6-*O*-benzoyl-2,3-di-*O*-benzyl-gluco-cyclohexene (**28**): Compound **3** (0.15 g, 0.26 mmol, 2.0 eq.) and 6-*O*-benzoyl-2,3-di-*O*-benzyl-gluco-cyclohexene-alkene[2] (0.057 g, 0.13 mmol, 1 eq.) were co-evaporated with anhydrous toluene thrice. The mixture was dissolved in 1.9 mL anhydrous DCM and cooled to -45 °C. TMSOTf (0.0046 mL, 0.026 mmol, 0.2 eq.) was added and the reaction was left to stir for 30 minutes at -45 °C. Subsequently the reaction was quenched with sat. $NaHCO_3$, the water layer re-extracted with DCM twice, organic layers combined and washed with brine, dried over $MgSO_4$, filtered and concentrated *in vacuo*. The crude product was purified using flash silica column chromatography (EtOAc/pentane, 1/10 -> 3/20, v/v) followed by a SEC (Sephadex-LH20, MeOH/DCM, 1:1, v/v) to remove any traces of acceptor to obtain compound **28** as colourless oil (0.088 g, 0.10 mmol, 78%). ^{1}H NMR (400 MHz, $CDCl_3$) δ 8.00 – 7.94 (m, 2H), 7.57 (t, *J* = 7.4 Hz, 1H), 7.47 – 7.40 (m, 6H), 7.40 – 7.21 (m, 24H), 5.80 (dt, *J* = 10.1, 2.6 Hz, 1H), 5.66 (dt, *J* = 10.2, 2.1 Hz, 1H), 5.52 (s, 1H), 5.11 (d, *J* = 10.9 Hz, 1H), 4.96 (d, *J* = 11.9 Hz, 1H), 4.87 (d, *J* = 11.9 Hz, 1H), 4.77 – 4.67 (m, 4H), 4.60 (d, *J* = 11.6 Hz, 2H), 4.42 – 4.31 (m, 2H), 4.21 (dq, *J* = 7.3, 2.6 Hz, 1H), 4.13 (t, *J* = 9.5 Hz, 1H), 4.02 (d, *J* = 3.0 Hz, 1H), 4.00 – 3.92 (m, 2H), 3.82 (dd, *J* = 9.5, 7.0 Hz, 1H), 3.58 – 3.50 (m, 2H), 3.10 (td, *J* = 9.7, 4.8 Hz, 1H), 2.72 (dq, *J* = 9.1, 3.3 Hz, 1H). ^{13}C NMR (101 MHz, $CDCl_3$) δ 139.3, 138.5, 137.7, 133.5, 129.6, 128.9, 128.7, 128.6, 128.5, 128.4, 128.3, 128.3, 128.2, 127.9, 127.8, 127.7, 127.6, 127.4, 126.2, 102.6, 101.4, 82.8, 79.7, 79.1, 78.6, 76.8, 75.1, 74.7, 72.6, 72.5, 68.6, 67.7, 64.0, 43.3. HRMS calculated for $[C_{55}H_{54}O_{10} + NH_4]^+$: 892.4055, found 892.4055.

2,3-di-*O*-benzyl-4,6-*O*-benzylidene-(1-4)-β-D-mannopyranosyl-2,3-di-*O*-benzyl-gluco-cyclohexene (**29**): Compound **28** (0.65 g, 0.74 mmol, 1 eq.) was suspended in 2 mL DCE/MeOH (1:5, v/v). NaOMe 4.3 M (0.069 mL, 0.30 mmol, 0.4 eq.) was added and the reaction mixture was left to stir overnight. The reaction mixture was quenched with Amberlite IR120 H^+ resin, filtered and concentrated *in vacuo.* The crude product was purified using flash silica column chromatography (EtOAc/pentane, 3/10, v/v) to obtain compound **29** (0.50 g, 0.65 mmol, 88%). ^{1}H NMR (400 MHz, $CDCl_3$) δ 7.50 – 7.20 (m, 26H), 5.80 (dt, *J* = 10.1, 2.6 Hz, 1H), 5.59 – 5.53 (m, 2H), 4.99 (dd, *J* = 23.8, 11.5 Hz, 2H), 4.84 (d, *J* = 11.9 Hz, 1H), 4.79 – 4.73 (m, 2H), 4.71 – 4.60 (m, 4H), 4.19 – 4.12 (m, 2H), 4.04 (dd, *J* = 10.4, 4.9 Hz, 1H), 4.00 – 3.92 (m, 2H), 3.83 (dd, *J* = 9.4, 6.8 Hz, 1H), 3.66 – 3.55 (m, 4H), 3.18 (td, *J* = 9.7, 4.8 Hz, 1H), 2.51 – 2.45 (m, 1H). ^{13}C NMR (101 MHz, $CDCl_3$) δ 139.2, 138.7, 138.6, 138.5, 137.7, 129.0, 128.7, 128.6, 128.5, 128.5, 128.4, 128.3, 128.3, 128.2, 128.0, 127.8, 127.7, 127.7, 127.6, 127.6, 127.5, 126.2, 102.0, 101.5, 82.4, 79.5, 78.9, 78.6, 76.7, 74.8, 74.4, 72.8, 72.3, 68.7, 67.6, 63.1, 45.5. HRMS calculated for $[C_{48}H_{50}O_9 + NH_4]^+$: 788.3793, found 788.3790.

2,3-di-*O*-benzyl-4,6-*O*-benzylidene-(1-4)-β-D-mannopyranosyl-2,3-di-*O*-benzyl-cyclophellitol (**30**): Compound **29** (0.096 g, 0.13 mmol, 1 eq.) was dissolved in 1.2 mL DCM at 0 °C. mCPBA (0.086 g, 0.50

mmol, 4 eq.) was added and the reaction was left to stir for four days at 4 °C. The solvent was removed *in vacuo*, and the crude residue was re-dissolved in EtOAc, washed with H_2O and brine, filtered and concentrated *in vacuo*. The crude product was purified using column chromatography (EtOAc/pentane, 3/10 -> 1/2, v/v) to obtain compound **30** (0.045 g, 0.057 mmol, 46%). ^1H NMR (400 MHz, $CDCl_3$) δ 7.48 – 7.39 (m, 4H), 7.37 – 7.21 (m, 21H), 5.51 (s, 1H), 5.00 (dd, *J* = 22.5, 11.4 Hz, 2H), 4.85 – 4.59 (m, 7H), 4.12 (t, *J* = 9.5 Hz, 1H), 4.03 – 3.93 (m, 2H), 3.87 – 3.82 (m, 2H), 3.77 – 3.70 (m, 2H), 3.61 – 3.49 (m, 3H), 3.29 (dt, *J* = 4.0, 1.1 Hz, 1H), 3.16 – 3.08 (m, 2H), 2.13 – 2.02 (m, 1H). ^{13}C NMR (101 MHz, $CDCl_3$) δ 139.2, 138.6, 138.5, 138.4, 137.7, 137.7, 129.1, 128.9, 128.6, 128.4, 128.4, 128.3, 128.2, 128.2, 128.0, 128.0, 127.8, 127.6, 127.6, 127.5, 127.4, 126.1, 125.4, 102.2, 101.4, 82.9, 79.2, 78.8, 77.4, 75.5, 74.9, 74.7, 73.3, 72.8, 68.6, 67.4, 62.1, 56.3, 52.6, 43.7, 29.7. HRMS calculated for $[C_{48}H_{50}O_{10} + NH_4]^+$: 804.3642, found 804.3742.

β-D-Mannose-(1-4)-cyclophellitol (**17**): Compound **30** (0.025 g, 0.032 mmol, 1 eq.) was co-evaporated with anhydrous toluene thrice and dissolved in 1.3 mL anhydrous THF. *t*-BuOH (0.15 mL, 1.59 mmol, 50 eq.) was added to this solution. In an empty round bottom flask liquid ammonia was prepared fresh by cooling the round bottom flask to -60 °C while maintaining a gentle gaseous flow of ammonia in the flask. Upon reaching the desired amount of liquid ammonia the flask was sealed off and placed under nitrogen atmosphere. Solid sodium (0.037 g, 1.59 mmol, 50 eq.) was added to the liquid ammonia and the resulting solution turned blue. Compound **22** was added to the blue solution, and the reaction was left to stir for 15 minutes. The reaction was quenched with 1 mL AcOH/H_2O (0.091 mL, 1.59 mmol, 50 eq.) after which the blue colour disappeared. The reaction flask was transferred to a warm water bath and stirred for 30 minutes. The remaining solution was concentrated *in vacuo*. The crude residue was purified using gel filtration to obtain compound **17** (0.81 mg, 2.4 µmol, 7.5%). ^1H NMR (850 MHz, D_2O) δ 4.69 (s, 1H), 4.10 (d, *J* = 3.3, 0.9 Hz, 1H), 3.97 (dd, *J* = 11.3, 3.4 Hz, 1H), 3.92 (dd, *J* = 12.4, 2.3 Hz, 1H), 3.88 – 3.84 (m, 2H), 3.75 (dd, *J* = 12.4, 6.3 Hz, 1H), 3.65 (dd, *J* = 9.6, 3.2 Hz, 1H), 3.60 (t, *J* = 9.7 Hz, 1H), 3.57 – 3.56 (m, 1H), 3.56 – 3.54 (m, 2H), 3.40 – 3.37 (m, 1H), 3.24 (d, *J* = 3.8 Hz, 1H), 2.30 – 2.26 (m, 1H). ^{13}C NMR (214 MHz, D_2O) δ 100.8, 77.7, 76.4, 74.9, 72.8, 70.8, 70.5, 70.5, 66.5, 60.8, 60.0, 56.6, 56.5, 55.1, 42.6. HRMS calculated for $[C_{13}H_{22}O_{10} + Na]^+$: 361.1105, found 361.1105.

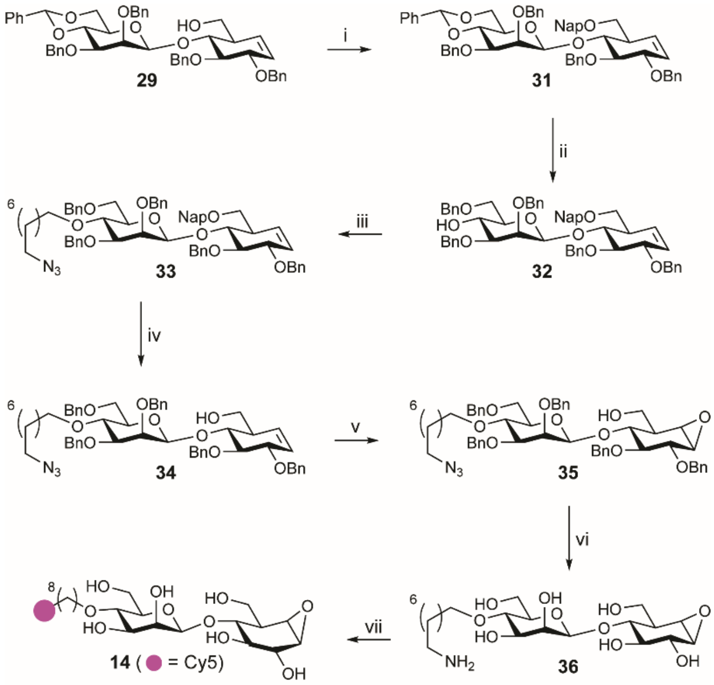

SI scheme 6. i) NapBr, NaH, TBAI, DMF, 99%. ii) 1) CSA, MeOH, 50 °C. 2) 2-aminoethyl diphenylborinate, BnBr, KI, K_2CO_3, 73%. iii) 1-azido-8-iodooctane, NaH, DMF, 96%. iv) DDQ, DCM/H_2O, 62%. v) 1) Boc_2O, DMAP, THF. 2) NIS, AcOH. 3) NaOMe, DCM/MeOH, 58%. vi) Na, NH_3, t-BuOH, THF, -60 °C, crude. vii) 1) Cy5-COOH, pentafluorophenyl trifluoroacetate, DIPEA, DMF, 2) **36**, DIPEA, DMF, 12%.

2,3-di-*O*-benzyl-4,6-*O*-benzylidene-(1-4)-β-D-mannopyranosyl-2,3-di-*O*-benzyl-6-*O*-naphthyl-gluco-cyclohexene (**31**): Compound **29** (1.80 g, 2.35 mmol, 1 eq.) was co-evaporated with anhydrous toluene thrice and dissolved in 23.4 mL anhydrous DMF. To this solution naphthyl bromide (1.03 g, 4.67 mmol, 1.5 eq.) was added. After the solution was cooled to 0 °C NaH (60% wt, 0.14 g, 3.5 mmol, 1.5 eq.) was added. The mixture was left to react for 2 hours and then checked by TLC for full conversion. The reaction was quenched at 0 °C by the addition of H_2O followed by further dilution with H_2O and extraction of the aqueous layer with Et_2O thrice. The combined organic layers were washed with water five times, brine, dried over $MgSO_4$, filtered and concentrated *in vacuo*. The crude product was purified using flash column chromatography (EtOAc/pentane, 1/20 -> 3/20, v/v) to obtain compound **31** (2.1 g, 2.30 mmol, 99%). ^{1}H NMR (400 MHz, $CDCl_3$) δ 7.83 – 7.69 (m, 4H), 7.51 – 7.17 (m, 28H), 5.76 (dt, *J* = 10.1, 2.6 Hz, 1H), 5.61 (dt, *J* = 10.1, 2.1 Hz, 1H), 5.48 (s, 1H), 5.09 (d, *J* = 10.9 Hz, 1H), 4.84 – 4.64 (m, 6H), 4.48 (d, *J* = 12.4 Hz, 2H), 4.37 (d, *J* = 1.0 Hz, 1H), 4.28 – 4.18 (m, 2H), 4.06 – 3.94 (m, 3H), 3.74 (dd, *J* = 9.7, 7.4 Hz, 1H), 3.68 (dd, *J* = 3.2, 0.9 Hz, 1H), 3.54 – 3.45 (m, 3H), 3.07 – 2.98 (m, 2H), 2.56 – 2.48 (m, 1H). ^{13}C NMR (101 MHz, $CDCl_3$) δ 139.6, 138.8, 138.8, 137.9, 135.4, 133.2, 133.1, 128.9, 128.7, 128.6, 128.5, 128.4, 128.3, 128.3, 128.2, 128.0, 128.0, 127.9, 127.9, 127.8, 127.7, 127.5, 127.5, 127.3, 127.0, 126.6, 126.4, 126.2, 126.1, 102.3, 101.3, 83.7, 83.2, 79.8, 79.4, 78.7, 78.6, 77.1, 75.0, 74.7, 73.5, 72.6, 72.5, 69.6, 67.4, 44.5. HRMS calculated for $[C_{59}H_{58}O_9 + NH_4]^+$: 928.4419, found 928.4419.

2,3,6-di-*O*-benzyl-(1-4)-β-D-mannopyranosyl-2,3-di-*O*-benzyl-6-*O*-naphthyl-gluco-cyclohexene (**32**): Compound **31** (2.13 g, 2.34 mmol, 1 eq.) was dissolved in 23.40 mL MeOH/DCE, v/v, 1/1 and heated to 50 °C. CSA (0.12 g, 1.17 mmol, 0.5 eq.) was added and the reaction was left to stir for 1 hour. After TLC showed full conversion, the reaction was cooled in an ice-bath and quenched with Et_3N. The solvent was removed *in vacuo*, and the crude residue was re-dissolved in EtOAc, washed with brine, dried over $MgSO_4$, filtered and concentrated *in vacuo*. It was then co-evaporated with dry toluene thrice and dissolved in 1.5 mL dry ACN. To this solution was added Taylor's catalyst (0.14 g, 0.61 mmol, 0.3 eq.), benzyl bromide (0.36 mL, 3.06 mmol, 1.5 eq.), KI (0.37 g, 2.25 mmol, 1.1 eq.) and K_2CO_3 (0.31 g, 2.25 mmol, 1.1 eq.). The reaction mixture was heated to 65 °C and left to stir overnight. Solvent was removed *in vacuo*, and the crude residue was re-dissolved in EtOAc, washed with H_2O and brine, dried over $MgSO_4$, filtered and concentrated *in vacuo*. The crude residue was purified using flash column chromatography (acetone/toluene, 1/20 -> 1/10, v/v) to obtain compound **32** (1.56 g, 1.7 mmol, 73%). ^{1}H NMR (400 MHz, $CDCl_3$) δ 7.82 – 7.75 (m, 3H), 7.71 (s, 1H), 7.47 – 7.38 (m, 4H), 7.37 – 7.11 (m, 24H), 5.74 (dt, *J* = 10.2, 2.6 Hz, 1H), 5.62 (dt, *J* = 10.1, 2.1 Hz, 1H), 5.08 (d, *J* = 11.2 Hz, 1H), 4.80 (d, *J* = 12.1 Hz, 1H), 4.73 – 4.61 (m, 5H), 4.53 – 4.39 (m, 3H), 4.34 (d, *J* = 12.0 Hz, 1H), 4.23 – 4.11 (m, 2H), 4.02 (dd, *J* = 18.8, 10.3 Hz, 2H), 3.89 – 3.77 (m, 2H), 3.65 (dd, *J* = 9.8, 3.6 Hz, 2H), 3.51 (ddt, *J* = 26.2, 10.5, 5.4 Hz, 3H), 3.24 (ddd, *J* = 9.9, 5.9, 4.4 Hz, 1H), 2.87 (dd, *J* = 9.4, 2.9 Hz, 1H), 2.67 – 2.59 (m, 2H). ^{13}C NMR (101 MHz, $CDCl_3$) δ 139.6, 138.9, 138.7, 138.5, 138.2, 135.4, 133.2, 133.1, 128.8, 128.6, 128.4, 128.4, 128.3, 128.2, 128.1, 128.1, 128.1, 128.0, 127.9, 127.9, 127.9, 127.8, 127.7, 127.7, 127.7, 127.7, 127.6, 127.6, 127.6, 127.5, 127.4, 127.2, 127.1, 126.9, 126.5, 126.3, 126.2, 126.1, 125.9, 101.5, 82.8, 81.9, 79.8, 78.4, 75.1, 74.5, 74.4, 74.2, 73.7, 73.5, 72.3, 71.3, 71.2, 69.8, 68.8, 44.2. HRMS calculated for $[C_{59}H_{60}O_9 + NH_4]^+$: 930.4576, found 930.4576.

4-*O*-(8-azidooctyl)-2,3,6-tri-*O*-benzyl-(1-4)-β-D-mannopyranosyl-2,3-di-*O*-benzyl-6-*O*-naphthyl-gluco-cyclohexene (**33**): Compound **32** (1.06 g, 1.16 mmol, 1 eq.) was co-evaporated with toluene thrice and dissolved in 5.8 mL dry DMF. To this solution was added 8-azidooctan-1-ol (3.26 g, 11.61 mmol, 10 eq.) and the resulting mixture was cooled to 0 °C. Sodium hydride (60% wt) (0.19 g, 4.64 mmol, 4 eq.) was added and the mixture was left to stir for 3 hours. After TLC showed full conversion the reaction mixture was quenched with H_2O. The resulting mixture was extracted wit Et_2O thrice, the organic layers were combined, washed with H_2O five times and brine, dried over $MgSO_4$, filtered and concentrated *in vacuo*. The crude product was purified using flash column chromatography (acetone/pentane, 1/20 -> 1/10, v/v) to obtain compound **33** (1.19 g, 1.1 mmol, 96%). ^{1}H NMR (400 MHz, $CDCl_3$) δ 7.81 – 7.76 (m, 3H), 7.71 – 7.68 (m, 1H), 7.45 – 7.40 (m, 3H), 7.37 – 7.14 (m, 25H), 5.72

(dt, *J* = 10.1, 2.6 Hz, 1H), 5.61 (dt, *J* = 10.2, 2.0 Hz, 1H), 5.13 (d, *J* = 11.5 Hz, 1H), 4.82 – 4.77 (m, 2H), 4.70 – 4.65 (m, 2H), 4.62 (d, *J* = 3.3 Hz, 2H), 4.59 – 4.45 (m, 3H), 4.45 – 4.35 (m, 2H), 4.19 – 4.11 (m, 3H), 4.01 (t, *J* = 9.2 Hz, 1H), 3.83 – 3.71 (m, 3H), 3.69 (dd, *J* = 4.0, 2.3 Hz, 2H), 3.58 – 3.48 (m, 4H), 3.46 – 3.39 (m, 1H), 3.29 – 3.24 (m, 1H), 3.21 (t, *J* = 6.9 Hz, 2H), 3.02 (dd, *J* = 9.3, 3.0 Hz, 1H), 2.65 – 2.59 (m, 1H), 1.59 – 1.42 (m, 4H), 1.36 – 1.18 (m, 8H). ^{13}C NMR (101 MHz, $CDCl_3$) δ 139.7, 139.1, 139.1, 138.7, 138.6, 135.5, 133.2, 133.1, 128.9, 128.6, 128.4, 128.3, 128.2, 128.1, 128.0, 128.0, 128.0, 127.9, 127.9, 127.6, 127.6, 127.5, 127.4, 127.3, 127.3, 127.2, 127.1, 126.8, 126.4, 126.1, 126.0, 101.4, 82.9, 82.6, 79.8, 78.2, 76.6, 75.1, 74.9, 74.5, 74.0, 73.6, 73.5, 73.4, 73.1, 72.3, 71.4, 69.9, 69.8, 51.5, 44.2, 30.4, 29.4, 29.4, 29.2, 29.2, 28.9, 26.7, 26.1, 26.1. HRMS calculated for $[C_{67}H_{75}N_3O_9 + NH_4]^+$: 1083.5842, found 1083.5842.

4-*O*-(8-azidooctyl)-2,3,6-tri-*O*-benzyl-(1-4)-β-D-mannopyranosyl-2,3-di-*O*-benzyl-gluco-cyclohexene (**34**): Compound **33** (0.41 g, 0.39 mmol, 1 eq.) was dissolved in a mixture of DCM/H_2O (3.9 mL, 18/2, 0.1M) and cooled to 0 °C. β-Pinene (0.30 mL, 1.94 mmol, 5 eq.) and DDQ (0.35 g, 1.55 mmol, 4 eq.) were added and the reaction was left to stir at RT. After two hours TLC showed full conversion and the reaction was diluted with DCM, transferred to a separatory funnel, washed with 2M NaOH thrice and brine, dried over $MgSO_4$, filtered and concentrated *in vacuo*. The crude product was purified using flash column chromatography (EtOAc/pentane, 1/20 -> 1/10, v/v) to obtain compound **34** (0.22 g, 0.24 mmol, 62%). ^{1}H NMR (400 MHz, $CDCl_3$) δ 7.41 – 7.15 (m, 25H), 5.73 (dt, *J* = 10.1, 2.6 Hz, 1H), 5.60 (dt, *J* = 10.2, 2.1 Hz, 1H), 4.88 (dd, *J* = 11.7, 7.7 Hz, 2H), 4.81 – 4.76 (m, 2H), 4.64 (s, 1H), 4.59 (d, *J* = 4.3 Hz, 2H), 4.53 (s, 2H), 4.45 (d, *J* = 4.2 Hz, 2H), 4.09 (t, *J* = 9.0 Hz, 1H), 4.06 – 4.02 (m, 1H), 3.89 (d, *J* = 2.8 Hz, 1H), 3.87 – 3.82 (m, 1H), 3.80 – 3.75 (m, 1H), 3.75 – 3.68 (m, 2H), 3.67 – 3.59 (m, 3H), 3.44 – 3.39 (m, 1H), 3.39 – 3.32 (m, 2H), 3.22 (t, *J* = 7.0 Hz, 2H), 2.55 – 2.47 (m, 1H), 1.55 (q, *J* = 7.1 Hz, 2H), 1.49 – 1.40 (m, 2H), 1.34 – 1.17 (m, 8H). ^{13}C NMR (101 MHz, $CDCl_3$) δ 139.1, 139.1, 138.5, 138.4, 128.8, 128.5, 128.5, 128.4, 128.3, 128.2, 128.0, 127.9, 127.8, 127.7, 127.7, 127.6, 127.6, 127.5, 127.5, 127.3, 99.6, 82.6, 81.9, 79.7, 76.8, 76.1, 75.0, 75.0, 74.1, 73.6, 73.5, 73.2, 71.9, 71.7, 69.7, 63.4, 51.5, 45.5, 30.4, 29.4, 29.2, 28.9, 26.8, 26.1. HRMS calculated for $[C_{56}H_{67}N_3O_9 + NH_4]^+$: 943.5216, found 943.5216.

4-*O*-(8-azidooctyl)-2,3,6-tri-*O*-benzyl-(1-4)-β-D-mannopyranosyl-2,3-di-*O*-benzyl-6-*O*-(tert-butoxycarbonyl)-gluco-cyclohexene (**35a**): Compound **34** (0.22 g, 0.24 mmol, 1 eq.) was co-evaporated thrice with anhydrous toluene and subsequently dissolved in 1.6 mL anhydrous THF. Boc_2O (0.11 mL, 0.48 mmol, 2 eq.) and DMAP (0.023 g, 0.19 mmol, 0.8 eq.) were added and the reaction mixture was stirred overnight. The reaction mixture was quenched and diluted with H_2O. The water layer was extracted thrice with Et_2O, the combined organic layers were washed with sat. NH_4Cl (aq.), sat. aq. $NaHCO_3$, brine, dried over $MgSO_4$, filtered and concentrated *in vacuo*. The crude product was used without purification in the next step.

4-*O*-(8-azidooctyl)-2,3,6-tri-*O*-benzyl-(1-4)-β-D-mannopyranosyl-2,3-di-*O*-benzyl-6,7-carbonate-glycopyranosyl iodide (**35b**): Crude compound **35a** (0.049, 0.048 mmol, 1 eq.) was dissolved in 0.24 mL AcOH. NIS (0.021 g, 0.095 mmol, 2 eq.) was added and the reaction was stirred overnight. The reaction mixture was diluted with Et_2O, quenched with Et_3N, washed with sat. aq. $NaHCO_3$, 10% aq. $Na_2S_2O_3$, brine, dried over $MgSO_4$, filtered and concentrated *in vacuo*. The crude product was used without further purification in the next step.

4-*O*-(8-azidooctyl)-2,3,6-tri-*O*-benzyl-(1-4)-β-D-mannopyranosyl-2,3-di-*O*-benzyl-cyclophellitol (**35**): Compound **35b** (crude, 0.048 mmol, 1 eq.) was dissolved in 0.24 mL MeOH/DCM, 1/1, v/v and NaOMe (5.4M in MeOH) was added catalytically and the reaction was stirred overnight. The reaction mixture was diluted with EtOAc, washed with H_2O, brine, dried over $MgSO_4$, filtered and concentrated *in vacuo*. The crude product was purified using flash silica column chromatography (EtOAc/pentane, 1/10 -> 3/10, v/v) to obtain compound **35** (0.026 g, 0.028 mmol, 58%). ^{1}H NMR (400 MHz, $CDCl_3$) δ 7.37 – 7.20 (m, 25H), 4.90 – 4.83 (m, 2H), 4.79 – 4.63 (m, 4H), 4.59 – 4.55 (m, 1H), 4.53 – 4.44 (m, 4H), 4.06 – 3.96 (m, 1H), 3.85 (dd, J = 2.9, 0.9 Hz, 1H), 3.81 – 3.73 (m, 4H), 3.66 (dd, J = 10.8, 2.2 Hz, 1H), 3.62 – 3.55 (m,

3H), 3.43 – 3.37 (m, 1H), 3.37 – 3.27 (m, 3H), 3.23 (t, J = 7.0 Hz, 2H), 3.14 (d, J = 3.7 Hz, 1H), 2.50 (d, J = 9.0 Hz, 1H), 2.25 – 2.15 (m, 1H), 1.59 – 1.50 (m, 2H), 1.48 – 1.39 (m, 2H), 1.31 – 1.18 (m, 8H). ^{13}C NMR (101 MHz, $CDCl_3$) δ 139.1, 139.0, 138.5, 138.4, 137.7, 128.7, 128.6, 128.5, 128.4, 128.4, 128.3, 128.3, 128.2, 128.1, 128.1, 128.0, 128.0, 127.9, 127.7, 127.7, 127.6, 127.6, 127.4, 127.4, 99.8, 82.5, 82.0, 79.4, 76.0, 75.1, 75.0, 74.1, 74.0, 73.9, 73.5, 73.2, 73.2, 71.9, 69.8, 62.6, 56.4, 53.0, 51.6, 44.0, 30.4, 29.8, 29.5, 29.2, 28.9, 26.8, 26.1. HRMS calculated for $[C_{56}H_{67}N_3O_{10} + NH_4]^+$: 959.5165, found 959.5165.

4-*O*-(8-aminooctyl)-β-D-mannose-(1-4)-cyclophellitol (**36**): Compound **35** (0.009 g, 9.4 µmol, 1 eq.) was co-evaporated with anhydrous toluene thrice and dissolved in 0.4 mL anhydrous THF. *t*-BuOH (0.054 mL, 0.57 mmol, 60 eq.) was added to this solution. In an empty round bottom flask liquid ammonia was prepared fresh by cooling the round bottom flask to -60 °C while maintaining a gentle gaseous flow of ammonia in the flask. Upon reaching the desired amount of liquid ammonia the flask was sealed off and placed under nitrogen atmosphere. Solid sodium (0.013 g, 0.57 mmol, 60 eq.) was added to the liquid ammonia and the resulting solution turned blue. Compound **35** was added to the blue solution, and the reaction was left to stir for 10 minutes. The reaction was quenched with 1 mL AcOH/H_2O (0.032 mL, 0.57 mmol, 60 eq.) after which the blue colour disappeared. The reaction flask was transferred to a warm water bath and stirred for 30 minutes. The remaining solution was concentrated *in vacuo*. An attempt was made to purify the crude product using gel filtration, but salts remained. Compound **36** (0.002 g, 4.3 µmol, 46%) was used crude in the next step.

4-*O*-(8-Cy5-octyl)-β-D-mannose-(1-4)-cyclophellitol (**14**): Cy5-COOH (5.50 mg ,0.011 mmol, 1 eq.) was dissolved in 0.2 mL anhydrous DMF. PFP-TFA (from a stock solution of 60 µL PFP-TFA in 440 µL DMF: 0.04 mL, 0.034 mmol, 3 eq.) and DIPEA (200 µL in 300 µL DMF) (0.05 mL, 0.11 mmol, 10 eq.) were added to the solution and the resulting mixture was stirred at RT. The reaction was analysed with LC-MS (reversed phase, ACN/H_2O/1% TFA, 1/10 -> 9/10) every 30 minutes. After LC-MS analysis confirmed the absence of starting material the reaction was quenched by adding H_2O (from a stock solution of 60 µL H_2O in 2.5 mL DMF: 25 µL, 0.034 mmol, 3 eq.) and stirred for 15 minutes. Compound **36** (2 mg, 4.30 µmol, 1 eq.) was dissolved in 285 µL anhydrous DMF. To this was added 175 µL of the activated Cy5 stock (6.44 µmol, 1.5 eq.) and the reaction was left to stir overnight. LC-MS showed full conversion of the starting material. Subsequently the mixture was concentrated *in vacuo* and purified using a BAKERBOND spe™ C18 reverse phase column to obtain slightly impure compound **14** (0.48 mg, 0.52 µmol, 12%). ^{1}H NMR (850 MHz, MeOD) δ 8.26 (t, J = 11.9 Hz, 2H), 7.51 (d, J = 7.3 Hz, 2H), 7.43 (qd, J = 7.8, 1.2 Hz, 2H), 7.33 – 7.27 (m, 4H), 6.64 (t, J = 12.4 Hz, 2H), 6.29 (dd, J = 13.7, 8.9 Hz, 2H), 4.52 (s, 1H), 4.12 (t, J = 7.5 Hz, 2H), 3.95 – 3.89 (m, 3H), 3.87 – 3.81 (m, 3H), 3.79 – 3.74 (m, 2H), 3.69 – 3.63 (m, 5H), 3.58 – 3.51 (m, 3H), 3.44 – 3.42 (m, 1H), 3.42 – 3.40 (m, 2H), 3.25 – 3.24 (m, 1H), 3.23 – 3.21 (m, 1H), 3.15 – 3.13 (m, 2H), 3.06 (d, J = 3.6 Hz, 1H), 2.21 (t, J = 7.2 Hz, 2H), 2.14 – 2.09 (m, 2H), 1.92 (s, 6H), 1.87 – 1.83 (m, 3H), 1.75 (s, 9H), 1.73 – 1.69 (m, 2H), 1.55 (ddt, J = 17.8, 13.0, 6.9 Hz, 4H), 1.51 – 1.45 (m, 4H), 1.33 (tt, J = 16.8, 5.0 Hz, 8H). ^{13}C NMR (214 MHz, MeOD) δ 175.7, 155.5, 144.3, 143.6, 142.7, 142.5, 129.8, 129.8, 126.3, 126.3, 123.4, 123.3, 112.1, 111.9, 104.3, 102.4, 79.0, 77.8, 77.2, 76.5, 75.4, 72.8, 62.5, 61.8, 56.7, 49.5, 49.4, 45.2, 44.8, 40.4, 36.7, 31.3, 30.5, 30.4, 30.3, 28.2, 28.0, 27.8, 27.4, 27.1, 26.5. HRMS calculated for $[C_{53}H_{76}N_3O_{11}]^+$: 930.5474, found 930.5474.

Biochemistry and molecular biology methods

The *A. niger* strains used in this study are listed in Supplementary Table 1. MA234.1 is a parental strain of *A. niger*. MA1029.4 is characterised by 10 glucoamylase landing sites (GLS) carrying the *PglaA* promoter region (-635 to -247 upstream of ATG) and the *Tgla* terminator region (+142 to +547 after stop codon) separated by unique DNA sequences (dubbed KORE) which replace 10 genes hindering protein expression. The GLS were designed to allow easy integration of multiple copies of a gene of interest (GOI). Furthermore, they are *ku70* deficient, thus can repair DNA damage only by homologous recombination (HR).[3] Strains were grown on liquid or solidified (containing 1.5 % (w/v) Scharlau agar (Scharlau) minimal medium (MM) or on complete medium (CM). MM contains 7 mM KCl, 8 mM KH_2PO_4, 70 mM $NaNO_3$, and 2 mM $MgSO_4$ (pH adjusted to 5.5) and spore elements as described.[4] MM was supplemented with 50 mM carbon source. CM consists of MM supplemented with 0.1% Bacto casamino acids (Gibco, Thermo Fisher Scientific) and 0.5% w/v yeast extract and 50 mM glucose. Transformants of *A. niger* were isolated and purified as described[4] using a final concentration of 100 µg/mL hygromycin (InvivoGen). *Escherichia coli* DH5α was used for plasmid propagation and cultured at 37 °C in Lysogeny broth (LB) medium, with ampicillin (100 µg/mL).

For complex secretome production spores of *A. niger* were seeded on CM agar plates. After at least three days of incubation at 30 °C, the spores were harvested in physiological solution (PS). Freshly harvested spores were inoculated in liquid CM with 1% w/v D-fructose (Sigma) (CMF) at a concentration of 1 x 10^6 spores/mL. The liquid cultures were incubated at 30°C in a shaker at 200 RPM. After overnight incubation, 5g (wet weight) of mycelium was added to flasks containing 100 mL MM. The flasks were supplemented with 1% w/v UV-sterilised konjac flour (kF; non-industrial grade), guar gum (GG; non-industrial grade), locust bean gum (LBG; non-industrial grade), carob galactomannan (cGM; Megazyme) or konjac glucomannan (kG; Megazyme). The cultures were incubated at 30 °C in a rotary shaker at 200 RPM for at least three days. For recombinant protein production freshly harvested spores of the mannanase overexpressing strains were inoculated in liquid CM at a concentration of 1 x 10^6 spores/mL. The cultures were incubated at 30 °C in a rotary shaker at 200 RPM for at least three days. Secretome samples were filter sterilized with 0.02 µm filters (Amplitude EcoCloth), snap-frozen in liquid N_2 and stored at -80 °C.

Supplemental table 1. Strains of *Aspergillus niger* used in this study.

Strain	Genotype	Reference
MA234.1	*cspA, kusA::DR-amdS-DR*	5
MA1029.4	*ΔglaA; ΔaamA; ΔagdA; ΔamyA; ΔprtT; ΔpepA; ΔpepB; ΔpepN; ΔNRRL3_10267; ΔgoxC; ΔoahA; ΔNRRL3_06629* in MA234.1	3
MT1.5	*pepA :: manA-6xHis ; pepB :: manA-6xHis* in MA1029.4	This study
MT2.5	*pepA :: man26A-6xHis ; pepB :: man26A-6xHis* in MA1029.4	This study
RJAL1.5	*NRRL3_10267 :: man26A ; NRRL3_06629 :: man26A ; goxC :: man26A ; oahA :: man26A* in MA1029.4	This study
MA1178.5	*ΔmanA* in MA1029.4	This study
MA1179.1	*Δman26A* in MA1029.4	This study
MA1182.4	*ΔmanA; pepA::manA* E314Q-*6xHis; pepB::manA* E314Q-*6xHis* in MA1178.5	This study
MA1183.16	*Δman26A; pepA::man26A* E279Q-*6xHis; pepB::man26A* E279Q-*6xHis* in MA1179.1	This study
MA1114.1	*ΔmanA* in MA234.1	This study
MA1115.1	*Δman26A* in MA234.1	This study
MA1116.1	*ΔeglB* in MA234.1	This study
MA1117.1	*ΔcbhB* in MA234.1	This study
AMB3.3	*Δalg3* in MT1.5	This study
AMB4.1	*ΔmanF* in MA1114.1	This study

PCR amplifications were performed using either Phire Hot Start II DNA Polymerase (diagnostic PCR and bipartite flank amplification) or Phusion High-Fidelity DNA Polymerase (whole gene amplification) (Thermo Fisher Scientific) according to the instructions provided. All primers are listed in Supplementary Table 2. DNA fragments were purified using the GeneJET Gel Extraction Kit (Thermo Fisher Scientific) and ligations were carried out using the CloneJET PCR Cloning Kit or the Rapid DNA

Ligation Kit (Thermo Fisher Scientific). DNA sequencing of specific genes was performed by Macrogen (The Netherlands).

Supplemental table 2. List of primers employed to produce genetically engineered strains of *A. niger*.

Primer name	Sequence 5' - 3'	Scope
8912_P1f	GGCCGCGGTA CAGTAACTCC TCGG	*manA* KO in MA234.1 and MA1029.4
8912_P2r	AAACCCGAGG AGTTACTGTA CCGC	*manA* KO in MA234.1 and MA1029.4
8912_P3f	GGTTCTGGAGTCACACGCACT	*manA* KO in MA234.1 and MA1029.4
8912_P4r	TGTGACCGTT TGATTGATTT CC	*manA* KO in MA234.1 and MA1029.4
8912_P5f	GGAAATCAAT CAAACGGTCA CAGGGGGATACTGAGACTGAGATG	*manA* KO in MA234.1 and MA1029.4
8912_P6r	TCCGAGATCT CCTTCATGCT G	*manA* KO in MA234.1 and MA1029.4
8912_P7f	GCATGGATGACAAACCCGTT	dPCR *manA* KO in MA234.1 and MA1029.4
8912_P8r	TCAAACGACTCCAGATCGAGC	dPCR *manA* KO in MA234.1 and MA1029.4
4196_P1f	GGCCGGTGTG GCCCCTGCGA TTCT	*Man26A* KO in MA234.1 and MA1029.4
4196_P2r	AAACAGAATC GCAGGGGCCA CACC	*Man26A* KO in MA234.1 and MA1029.4
4196_P3f	CGTTTCCCTT GCCCAACA	*Man26A* KO in MA234.1 and MA1029.4
4196_P4r	TTTCGAAATG TACCAAATAC	*Man26A* KO in MA234.1 and MA1029.4
4196_P5f	GTATTTGGTA CATTTCGAAA GTGGATATGG TGGTTTTGAT G	*Man26A* KO in MA234.1 and MA1029.4
4196_P6r	CGATTCAACG TCTCGATGAG G	*Man26A* KO in MA234.1 and MA1029.4
4196_P7f	CGTTCGGTCA TCTCCTTTGG	dPCR *man26A* KO in MA234.1 and MA1029.4
4196_P8r	AAGCAGAAGG GACAAACAGG C	dPCR *man26A* KO in MA234.1 and MA1029.4
4917_P1f	GGCCGTGGTG AAAGCGGTCA CGGA	*eglB* KO in MA234.1
4917_P2r	AAACTCCGTG ACCGCTTTCA CCAC	*eglB* KO in MA234.1
4917_P3f	TCAATGATGA TGGGACGTCG	*eglB* KO in MA234.1
4917_P4r	CGCTACGACT GCTCGATCAA C	*eglB* KO in MA234.1
4917_P5f	GTTGATCGAG CAGTCGTAGC GGAACTGGGT GGGGTCGCAG ATG	*eglB* KO in MA234.1
4917_P6r	TTAGCAAGGC AACTGGCGTA A	*eglB* KO in MA234.1
4917_P7f	GTGTTTATGT GGGTGGGCTT G	dPCR *eglB* KO in MA234.1
4917_P8r	CGTATTCCCT GCTGTCCGTC	dPCR *eglB* KO in MA234.1
2584_P1f	GGCCGACGTG TCCAACCTTC CCTG	*cbhB* KO in MA234.1
2584_P2r	AAACCAGGGA AGGTTGGACA CGTC	*cbhB* KO in MA234.1
2584_P3f	AGTGCACGCG CATGAAGA	*cbhB* KO in MA234.1
2584_P4r	TGTGATTCAC TGCTTGAGCT C	*cbhB* KO in MA234.1
2584_P5f	GAGCTCAAGC AGTGAATCAC AACACTACTC GAAGGGGGAT G	*cbhB* KO in MA234.1
2584_P6r	CATCATCAAT CATCGCGCA	*cbhB* KO in MA234.1
2584_P7f	CTGGAAGGTC ATGGTCAGGG	dPCR *cbhB* KO in MA234.1
2584_P8r	GACGAGCAAG ACAGCTGCAA	dPCR *cbhB* KO in MA234.1
KOmanF_P1f	ACACTACTCGAAGGGGGATGAAGA	*manF* KO in MA1114.1
KOmanF_P2r	CTTGATCTGTCACTCTGGCAGTTACT	*manF* KO in MA1114.1
KOmanF_P3f	CCAGAGTGACAGATCAAGTTGGTTAGCTATATCTCTCT	*manF* KO in MA1114.1
KOmanF_P4r	AGTGAAGTGGGAGAGCAAAAGATGC	*manF* KO in MA1114.1
KOmanF_P5f	GTGAGTGGGTACACTTGCACGTAT	dPCR *manF* KO in MA1114.1
KOmanF_P6r	GTTGAGAAAAGAGAGAGGAACGAGC	dPCR *manF* KO in MA1114.1
sg_manF_P1F	GGCCACTGTGTCTGATTACAATGG	*manF* KO in MA1114.1
sg_manF_P2R	AAACCCATTGTAATCAGACACAGT	*manF* KO in MA1114.1
KOalg3P1f	GATGATAATGTCGGCAGATGGGCAAC	*alg3* KO in MT1.5
KOalg3P2r	CTTGCATGCGGTTGTTGAAC	*alg3* KO in MT1.5
KOalg3P3f	GTTCAACAACCGCATGCAAGAAGTGACACCCGGCCAGT	*alg3* KO in MT1.5
KOalg3P4r	TCGACGGTTTCTCGCATTTTG	*alg3* KO in MT1.5
KOalg3P5f	CCACATGTCTTTCCCTGTCC	*alg3* KO in MT1.5
KOalg3P6r	AGAGAGCGAGATTGGCAAAG	*alg3* KO in MT1.5
KOalg3P7r	GGAGCCATCCATTTTGTGTGTC	*alg3* KO in MT1.5
sgAlg3P1f	GGCCGCTGTGCTGTTATAGACAGT	*alg3* KO in MT1.5
sgAlg3P2r	AAACACTGTCTATAACAGCACAGC	*alg3* KO in MT1.5
PglaAP11F	TGATTTCCGCAACGGGAC	Expression of *manA* and *man26A* in MA1029.4
PglaAP19f_PmeI	ATAAGAATGTTTAAACTCCGGACGGTCAGGAACTT	Expression of *manA* and *man26A* in MA1029.4
man5P6f	CCAGCATCATTACACCTCAGCAATGAAGCTTTCCAACGCCCT	Expression of *manA* in MA1029.4
man26_P6f	CCAGCATCATTACACCTCAGCAATGTTCGCCAAACTGTCCCTC	Expression of *man26A* in MA1029.4
PglaAP18r	TGCTGAGGTGTAATGATGCTGG	Expression of *manA* and *man26A* in MA1029.4
TglaAP21f	CATCACCATCACCATCACTAGACAATCAATCCATTTCGC	Expression of *manA* and *man26A* in MA1029.4
TglaAP_HIS_man5P7r	GCGAAATGGATTGATTGTCTAGTGATGGTGATGGTGATGAGCACTACCAATAGCAGCAACAT	Expression of *manA* in MA1029.4
TglaAP_HIS_man26_P7r	GCGAAATGGATTGATTGTCTAGTGATGGTGATGGTGATGAGCCCCCTCCCAGTTCAG	Expression of *man26A* in MA1029.4
TglaA_no-his_P21f	TAGACAATCAATCCATTTCGC	Expression of *man26A* in MA1029.4
TglaAP23r_PmeI	ATAAGAATGTTTAAACCCGACATTCCAGCAATACTGC	Expression of *manA* and *man26A* in MA1029.4
TglaAP9r	TGGAGCCGATCAGACCAGTAG	Expression of *manA* and *man26A* in MA1029.4
8912_P9f	GCAAGCCATG TCTCCTGGAG CAGTACGGAG TCACCTCGAA CC	Production of *manA* E314Q mutant
8912_P10r	GGTTCGAGGT GACTCCGTAC TGCTCCAGGA GACATGGCTT GC	Production of *manA* E314Q mutant
8912_P11f	TCTCAACATC GACTCGGACG	Production of *manA* E314Q mutant
4196_P9f	CGAGAGAGTT TTGGCTATGG CACAGGTCGG TCCCATTCCG GACC	Production of *man26A* E279Q mutant
4196_P10r	GGTCCGGAAT GGGACCGACC TGTGCCATAG CCAAAACTCT CTCG	Production of *man26A* E279Q mutant
4196_P11f	CCCGGAAACG ACAAGTGTG	Production of *man26A* E279Q mutant
PepAP9f	GATGTTGGCTAACGTATGGCG	dPCR *manA* and *man26A* expression and mutant strains
PepAP10r	ATTGCGGAGC GGTGGTAGT	dPCR *manA* and *man26A* expression and mutant strains
PepBP7f	TGGACGAAGAAAGGGAGATGA	dPCR *manA* and *man26A* expression and mutant strains
PepBP8r	CTGCTGTGCTCGATTCCTGT T	dPCR *manA* and *man26A* expression and mutant strains

pepA_P17f	GTCGACTTGG ATGATGGAGG A	dPCR *manA* and *man26A* expression and mutant strains
pepA_P18r	GACGAGGCAC CGCAGTAGAT	dPCR *manA* and *man26A* expression and mutant strains
pepB_P15f	CGGCGGATTG CATGCTAT	dPCR *manA* and *man26A* expression and mutant strains
pepB_P16r	GATCGGCAGC ACAGCTTTCT	dPCR *manA* and *man26A* expression and mutant strains
10267_P9f	CATCGTTTCT GCACCGACC	dPCR *man26A* expression strain
10267_P10r	TGAACCTGAA GCGATGGGAT A	dPCR *man26A* expression strain
6629_P9f	TTCTCGAGTG GCTTGTGGTG	dPCR *man26A* expression strain
6629_P10r	ACGACATCCT GCTGAGGCA	dPCR *man26A* expression strain
goxC_P23f	TCGGTCCTCC TGTCACCTTC	dPCR *man26A* expression strain
goxC_P24r	GCATATTTAG CGAGGGCTTG C	dPCR *man26A* expression strain
oahA_P17f	TGGTCACCTT CTGGCCCTT	dPCR *man26A* expression strain
oahA_P18r	TTCAATCCCA TCCAATGCAG T	dPCR *man26A* expression strain

Production of *C. japonicus* lysate: The strain of *C. japonicus* Ueda 107 was grown in MOPS minimal medium[6] containing 0.25% carob galactomannan (Megazyme) as the sole carbon source. The lysate was prepared by pelleting cells from OD_{600}=1.5 culture and resuspending in 0.1 volumes of lysis reagent (0.02 mg/mL lysozyme, 100 U/mL benzonase, 20 mM pH 7.0 NaPi buffer, 1x BugBuster detergent (Thermo Fisher Scientific) and incubating for 15 minutes at 30 °C. Insoluble material was pelleted by centrifugation at 12000 x g. Finally, the supernatant was transferred to a new tube for further analyses.

Supplemental table 3. Plasmids used in this study.

Plasmid Name	Vector	Insert
pMT1.1	pJet1.2	*PmeI-PglaA-manA-6xHis-TglaA-PmeI*
pMT2.1	pJet1.2	*PmeI-PglaA-man26A-6xHis-TglaA-PmeI*
pMT3.1	pJet1.2	*PmeI-PglaA-man26A-TglaA-PmeI*
pMT4.1	pJet1.2	*PmeI-PglaA-manA E314Q-6xHis-TglaA-PmeI*
pMT5.1	pJet1.2	*PmeI-PglaA-man26A E279Q-6xHis-TglaA-PmeI*
pMT6.1	pLML001	sgRNA *ΔmanA*
pMT7.1	pLML001	sgRNA for *Δman26A*
pMT8.1	pLML001	sgRNA for *ΔeglB*
pMT9.1	pLML001	sgRNA for *ΔcbhB*
pMT10.1	pLML001	sgRNA for *Δalg3*
pMT11.1	pLML001	sgRNA for *ΔmanF*
pFC332 K4	pFC332	KORE4 sgRNA
pFC332 K2	pFC332	KORE2 sgRNA
pFC332 K3	pFC332	KORE3 sgRNA

Engineering of *A. niger* strains: Genetically modified strains of *A. niger* were produced as described.[3] In short, for gene knockout the 5' and 3' UTR of the gene of interest (GOI) were amplified and then fused together by PCR with specific primers. The PCR product was directly used for transformation in *A. niger*. For expression of recombinant mannanases, *PglA*, *TglaA* and either *manA* or *man26A* were amplified and then fused together by PCR with specific primers. *manA* E314Q and *man26A* E279Q mutants were generated by using specific primers introducing the desired mutation using the pMT1.1 and pMT2.1 plasmids as template DNA. The PCR products were cloned into the plasmid pJet 1.2 (Thermo Fisher Scientific) by blunt end ligation and transformed into competent *E. coli* DH5α cells via a heat shock protocol. Positive transformants were identified by restriction-digestion with *Hind*III (Thermo Fisher Scientific) and sequenced. For the knockouts, CRISPR/Cas9 single guide RNAs (sgRNAs) specific for each GOIs were obtained by annealing two complementary primers together to obtain a double-stranded sgRNA. This was done by incubating the two primers at 100 °C with 10x T4 ligase buffer (Thermo Fisher Scientific) for 5 min and then letting the temperature gradually decrease to 25 °C. The thus obtained sgRNA was ligated into the plasmid pLML001, which was previously linearized with *Esp3*I (Thermo Fisher Scientific), and then transformed in *E. coli* competent cells DH5α via a heat-shock protocol. The plasmid pLML001 contains an *A. niger* codon-optimized *cas9* gene from *S. pyogenes*, a gRNA expression cassette, a fungal *hph* gene for hygromycin resistance, a bacterial ampicillin resistance marker and an AMA1 sequence to provide autonomous replication.[3]

For recombinant mannanase expression in MA1029.4 and derived strains, a pFC322 plasmid was used, which carries a sgRNA specific for a KORE4 sequence, this way two copies of the GOI are integrated into the GLS in the *pepA* and *pepB* loci. Instead, when two pFC322 plasmid carrying a sgRNA specific

for KORE2 and KORE3 are used together, four copies of the GOI are integrated into the *NRRL3_10267*; *NRRL3_06629*; *goxC* and *oahA* loci.[3]
The transformation in *A. niger* was performed as described.[5] In short, *A. niger* protoplasts were transformed with 2-5 µg of the pLML001 plasmid containing the target sgRNA and with 1-2 µg of dDNA. Prior to the transformation the dDNA is digested from the plasmid with *Pme*I (Thermo Fisher Scientific). Plasmid curation was performed via two culturing steps first on MM agar plates with 100 µg/mL hygromycin and on MM agar without hygromycin. GOI knock out and knock in were confirmed by diagnostic PCR with specific primers. The difference in amplicon size compared to the background strain was assessed by electrophoresis on 1% agarose gel.

Purification of recombinant mannanases: One litre of secretome of both MT1.5, AMB3.3, MT2.5 and RJAL1.5 were produced by inoculating 1 x 10^9 spores in 1 L of liquid CM. The cultures were maintained for 3 days in rotary shaker at 200 rpm at 30 °C. On day 3, the secretomes were separated from the biomass by using a vacuum pump and 0.4 µm Whataman filters (Cytiva). The secretome were then re-filtered with a 0.2 µm Filtropur V50 Vacuum filtration unit (Sarstedt) and stored at -80 °C.

***An*ManA**: Prior to purification, the secretome of MT1.5 and AMB3.3 were concentrated to around 10-20 mL with 10,000 MWCO Amicon Ultra-15 Centrifugal Filter Units (Merk Millipore) and brought to 50 mL with ion exchange chromatography (IEX) buffer A (20 mM Na_2HPO_4; 20 mM NaCl; pH 7.4). ManA was purified on an NGC Medium-Pressure Chromatography System (Bio-Rad). IEX was performed by making use of a quaternary ammonium column HiTrap Q XL (Cytiva). The sample was loaded on the column with IEX buffer A and eluted with a gradient to 100% IEX buffer B (20 mM Na_2HPO_4; 1 M NaCl; pH 7.4) over 20 column volumes. The UV positive fractions were separated on SDS-PAGE. The fractions containing the target protein were pooled together, concentrated to around 500 µL and buffer exchanged to 20 mM McIlvaine buffer at pH 5. ManA from MT1.5 was treated with 5 µL of endoHf (NEB) in native conditions. ManA from AMB3.3 was not treated with endoHf (NEB). The protein sample was then purified by size exclusion chromatography (SEC) on a Superdex 75 increase 10/300 GL column (Cytiva) with a buffer containing 20 mM Na_2HPO_4 and 200 mM NaCl pH 5. The UV positive fractions were separated by SDS-PAGE. The fractions showing a band corresponding to the molecular weight of ManA were pooled together, concentrated to 15 mg/mL, snap-frozen in liquid nitrogen and stored at -80 °C.

***An*Man26A**: Prior to purification the secretome of MT2.5 and RJAL1.5 were concentrated to around 450 mL with 10 000 MWCO Amicon Ultra-15 Centrifugal Filter Units (Merk Millipore). $(NH_4)_2SO_4$ and Na_2HPO_4 were dissolved in sample to obtain a final concentration of 1.7 M and 50 mM respectively. The sample volume was brought to 500 mL with hydrophobic interaction chromatography (HIC) buffer A (1.7 M $(NH_4)_2SO_4$; 50 mM Na_2HPO_4; pH 7.0). Before loading onto the column, the sample was filtered with Filtropur V50 Vacuum filtration unit (Sarstedt). Man26A was purified by HIC with a HiScreen Butyl HP column (Cytiva). Elution was performed with a gradient of HIC buffer B (50 mM Na_2HPO_4; pH 7.0) over 20 CV. The UV positive fractions were separated by SDS-page. The fraction containing a band corresponding to the molecular weight of Man26A were pooled together, concentrated to around 500 µL and buffer exchanged to McIlvaine buffer at pH 5. EndoHf treatment and SEC were performed with the same protocol employed for ManA. Purified Man26A was concentrated to 15 mg/mL, snap-frozen in liquid nitrogen and stored at -80 °C.

EndoH/Hf treatment: During the purification, before SEC, *An*ManA from MT1.5 and *An*Man26A from both MT2.5 and RJAL1.5 were treated with endoH/Hf (NEB). Prior to the treatment, the protein solution was buffer exchanged to McIlvaine buffer at pH5 to around 500 µL. Next Glycobuffer 3 10x (NEB) and 5 µL of enzymes were added to the protein solutions. The samples were incubated ON at 37 °C. After the incubation, 4x Laemmli was added to a diluted sample aliquot and loaded on SDS-PAGE gel.

Production of recombinant mannanases in *E. coli*

***Cj*Man26A**, ***Cj*Man26C** and ***Aa*GH113**: Plasmids encoding *Aa*GH113 and *Cj*Man26C were reused from our previous study.[7] A gene encoding residues 39-419 of *Cj*Man26A[8] (Uniprot: P49424) was synthesized (Genscript) and cloned into pET24a between NdeI and SalI, giving a C-terminal His tag with the sequence VVDKLAAALEHHHHHH. E. coli BL21(DE3) (NEB) cells were transformed with each plasmid via heat shock and grown on LB-Kan (*Aa*GH113, *Cj*Man26A) or LB-Amp (*Cj*Man26C). A single colony from each transformation was used to inoculate 500 mL of pre-warmed ZYM-5052[9] (50 mM NaH_2PO_4, 17.5 g/L tryptonee, 10 g/L yeast extract, 3.3 g/L $(NH_4)_2SO_4$, 0.3 g/L $MgSO_4.7H_2O$, 5 g/L glycerol, 0.5 g/L glucose, 2 g/L lactose, titrated to pH 7.0 with NaOH and filter-sterilised) in a 4 L baffled flask, which was shaken vigorously with a foam cap at 37 °C for 4 hours, then shifted to 16C for overnight shaking incubation. Cells were collected by centrifugation (4000xg, 5 min) and resuspended in histrap binding buffer (50 mM NaPi, 500 mM NaCl, pH 7.5). Cells were lysed by sonication on ice, then the lysate was clarified at 16,000 xg for 10 minutes. Clarified lysate was loaded onto a 5 mL histrap column, washed with 2 CV of histrap binding buffer, then 2 CV of 5% elution buffer (50 mM NaPi, 500 mM NaCl, 500 mM imidazole, pH 7.5), then eluted with a gradient from 5-100% elution buffer. *Aa*GH113 purification yielded 20 mg of soluble protein and *Cj*Man26C and *Cj*Man26A purifications yielded >150 mg. 20 mg of each protein was concentrated down to 10-20 mg/mL using a 10 kDa centrifugal concentration then purified over a Superdex 200 26/60 SEC column into 20 mM MOPS pH 7.0. The heart cut from the largest SEC elution peak was pooled and concentrated down to 40-70 mg/mL and frozen at -80 °C in 100 µL aliquots. The material for crystallography, mass spectrometry, and kinetics came from a single preparation run.

In gel activity-based protein profiling: Fluorescent ABPs **1**, **12** and **14** were dissolved in DMSO to different concentrations. Besides the concentration range ABPP experiments, all other gel-based assays were performed with a 5 µM final concentration of probe. 1.5 µL of 10x probe solution was added to 15 µL of either secretome, lysate or purified protein solution. The secretome was used undiluted while the purified proteins were used at 0.1 µg/µL in McIlvaine buffer at pH 5. Where necessary, a negative control was included containing 1.5 µL of DMSO instead of a fluorescent probe. Samples were incubated for 1h at 37 °C. Following the incubation, 4x Laemmli buffer was added and samples were heated to 95°C for 5 min. Samples were separated by SDS-PAGE on 12.5% polyacrylamide gels (Biorad). Gels were imaged with either a Typhoon 5 laser scanner (GE Healthcare) or a Chemidoc system (Biorad). After imaging the gels were stained with Coomassie brilliant blue R250 (Biorad) and analysed with a Chemidoc system (Biorad).

PNGase F treatment: The secretomes of MT1.5 and MT2.5 and purified *An*ManA and *An*Man26A were treated with PNGase F (NEB) after labelling with fluorescent ABPs, as previously described. 1 µL of denaturation buffer (NEB) was added to the samples containing 15 µL of protein solution + 1.5 µL of 10x ABP solution. The proteins where denatured for 5 min at 95 °C. The pH of the samples was then adjusted with McIlvaine buffer at pH 8. To the samples were added 2 µL of 10x glycobuffer 2 (NEB), 2 µL of 10% NP-40 (NEB) and 1 µL of PNGase F enzyme (NEB). The samples were incubated for 2 hours at 37 °C. After the incubation, 4x Laemmli was added to all samples before loading them on SDS-PAGE gel.

Western blotting: Secretome samples were mixed with 4x Laemmli buffer and separated by SDS-PAGE. Two gels were run in parallel. One was stained with Coomassie blue R250 (Biorad) and used as reference, the other was transferred to a nitrocellulose membrane with Trans-Blot Turbo system (Biorad). The transfer was performed at 25 V, 1.3 A for 3 min. The membrane was washed for 15 min in demi water and for 15 min in 1x TBS (Tris buffer saline). Next the membrane was blocked for 90 min with a 5% w/v solution of powder milk (Campina) in TBST (Tris buffer saline + Tween-20). An anti C-terminal HIS-tag antibody (Thermo Fisher Scientific) was diluted 1:10 000 in TBST and poured onto the membrane after removal of the blocking solution. Antibody labelling was performed ON at room

temperature in the dark. The following day the antibody solution was removed, and the membrane was washed one time with demi water followed by four 5 min washes with TBST. The western blots were developed with TMB Enhanced One Component HRP Membrane Substrate (Sigma Aldrich) in the dark for around 2 min and photographed.

Total protein identification

***Aspergillus niger* MA234.1 grown on 1% kF**: 500 µL of secretome was reduced with tris(2-carboxyethyl) phosphine (TCEP) at final concentration of 5 mM for 30 min at 37 °C. Thiols were alkylated via the addition of iodoacetamide at a final concentration of 10 mM. Samples were incubated in the dark for 30 min at RT. The total volume was brought to 800 µL with 50 mM NH_4HCO_3 and proteins were digested ON at 37 °C with 10 µL of 0.5 µg/µl trypsin (Promega) in 1 mM HCl. Trypsin digestion was stopped with 20µL of a 1% aqueous formic acid solution. The samples were de-salted by stage-tipping (Empore) with the following solutions: pure MeOH, solution A (80% ACN, 0.1 % TFA and 19.9% water) and solution B (99% water, 0.1% trifluoracetic acid). The samples were then dried in speed-vac for 4h at 45°C V-AQ. Desalted peptide samples were reconstituted in 20-50µL of 97% water, 3% ACN, 0.05% TFA and injected on a Q Exactive Orbitrap MS (Thermo Fisher Scientific).

Pulldown experiments

***Aspergillus niger* MA234.1 grown on 1% kF**: Biotin probe **13** was dissolved in 1:10 DMSO and 9:10 Milli-Q water to a 100 µM concentration. For the negative control, a 1:10 dilution of DMSO in Milli-Q water was made. 30 µL of probe was added 270 µL of secretome. 30 µL of diluted aqueous DMSO was added to the negative control. Samples were incubated for 2h at 37 °C and the pH was adjusted to 7.5 with 1M NH_4HCO_3. Secretome proteins were denatured by adding 36 µL of denaturing reagent (760 µL Milli-Q water + 40 µL 1M dithiothreitol (Merck) + 200µL 10% SDS). 15 µL of 0.5M aqueous iodoacetamide was added and samples were incubated in the dark for 30 min at room temperature. 1.5 mL of acetone was added to the samples, followed by 1h incubation at -20 °C. Precipitates were collected by centrifugation at 15,000×g for 15 minutes. Supernatants were decanted and pellets were dried for 15-20 min at room temperature. Pellets were redissolved overnight in 100 µL 10M urea in a shaker at 750 RPM. 400 µL of a 0.05% w/v solution of SDS was added and samples were transferred to low protein binding tubes (Sarstedt). 20µL of Streptavidin Mag Sepharose beads suspension (Cytiva) were added and samples were incubated for 1h in a thermomixer at 25 °C at 1500 RPM. The samples were transferred to a magnetic rack and supernatant was removed. The beads were washed twice with 500 µL of 2% SDS, followed by an additional wash with 500 µL of 2M urea and three wash steps with 500 µL Milli-Q water. After the final wash steps, the aqueous layer was removed again, and the beads were suspended in 20 mM NH_4HCO_3 and 1 µL of 0.5 µg/µL sequencing grade trypsin (Promega). Samples were incubated overnight in a thermomixer at 37 °C and 1350 RPM. Trypsin digestion was stopped with 20µL of a 1% aqueous formic acid solution. The samples were de-salted by stage-tipping as previously described. The samples were dried in speed-vac for 4h at 45°C V-AQ. Desalted peptide samples were reconstituted in 20-50 µL of 97% water, 3% ACN, 0.05% TFA and injected on a Q Exactive HF Orbitrap MS.

***Cellvibrio japonicus* Ueda 107 grown on 0.25% cG**: Biotin probe **13** was dissolved in 20 µL of DMSO to give a 5 mM stock. 1.5 µL of 5 mM **13** were added to 37.5 µL of lysate tube buffered with 2.5 µL of 1 M pH 7 buffer. A negative control was prepared by adding 1 µL of DMSO instead of **13**. The samples were incubated for 3 hours at 37 °C. 4 µL of 10x denaturing/reducing buffer was added to each tube and they were heated to 80°C for 5 minutes, then cooled to RT. 2 µL of 0.5 M aqueous iodoacetamide (~20 mM final concentration) was added to each reaction and they were incubated at RT for 30 minutes in the dark to alkylate all thiols. To each sample was added 200 µL of MeOH, then 50 µL of $CHCl_3$, then 150 µL of water. The tubes were centrifuged at 14000 x g for 2 minutes and as much of the top layer was removed as possible, as well as some of the bottom layer. 200 µL of MeOH was added and the tube was vortexed and centrifuged again. The supernatant was removed and the pellet was partially air-dried and stored in the fridge. The pellets were dissolved in 50 µL of 10 M aqueous urea. 450 µL of

0.05% SDS was then added and the samples was transferred to lo-bind tubes (Eppendorf). 20 µL of Strep Mag Sepharose beads (Cytiva) were added. The mixture was incubated at 25 °C with constant shaking for 1 hour. The beads were then collected, and the supernatant was removed and discarded. The beads were washed with 1 mL 2% SDS at RT once, 1 mL of 2% SDS at 65 °C (10 minutes) once, 1 mL of 2 M aqueous urea (5 mins) once, then 1 mL of water twice. The beads were finally resuspended in 20 µL of 0.05 M TEAB. 1 µL of 0.5 µg/µL trypsin (Promega) in 50 mM aqueous acetic acid was added to each tube and the solutions were incubated overnight at 37 °C with constant shaking at 1200 RPM. The samples were then spun down; the beads were pulled down and the 20 µL supernatant was transferred to a PCR tube. TMT2 (tandem mass tag) solutions were taken from samples stored at -80 °C. 2 µL of TMT2-126 in EtOH was added to each negative control sample. 2 µL of TMT2-127 in EtOH was added to each probed sample. Excess reagent was quenched with 1 µL of 5% aqueous hydroxylamine (65 mM final) for 15 minutes at RT. 9 µL of the DMSO control peptides and 9 µL of **12** pulldown peptides were then mixed in a separate PCR tube and analysed by LC-MS.

Tryptic peptide mass spectrometry

***An*ManA**: Purified *An*ManA (2 µg) in 20 mM McIlvaine buffer at pH 5 were labelled with 1 mM of either inhibitor **15** or **16**. A negative control was prepared by replacing the inhibitor with DMSO. The samples were incubated for 2 hours at 37 °C. Next the samples were brought to 50 µL with 8M aqueous urea in 50 mM NH_4HCO_3 at pH 8 and then reduced with 1 mM TCEP and alkylated with 3 mM iodoacetamide in a one pot reaction for 30 min at 37 °C shaking at 800 RPM. 2 mM DTT was then added to quench excess iodoacetamide. Samples were incubated at RT for 30 min. The reaction volume was brought to 600 µL with 10 mM NH_4HCO_3 to reduce the concentration of urea to below 1 M and to bring the pH to a value between 7-8. The proteins samples were digested ON at 37°C with 0.4 µL of a 0.5 µg/µL trypsin (Promega) solution in 1 mM HCl. The next day the trypsin digestion was stopped by bringing the pH to 3 with 3 µL of formic acid. The samples were de-salted by stage-tipping. The samples were dried in speed-vac for 4h at 45°C V-AQ. Desalted peptide samples were reconstituted in 20-50 µL of 97% water, 3% ACN, 0.05% TFA and injected on a Q Exactive Orbitrap MS (Thermo Fisher Scientific).

***An*Man26A**: 20 µL of 0.5 µg/µL enzyme solution in McIlvaine buffer at pH 5 was labelled for 2 hours at 37 °C degrees with 10 mM of **16** in DMSO. After the incubation the volume was brought to 50 µL with lysis buffer (5% (w/v) SDS, 50 mM tetraethylammonium bicarbonate pH 8.5). The samples were then reduced with DTT at a final concentration of 5 mM for 15 min at 65 °C. Next the samples were treated with iodoacetamide (final concentration 10 mM) in the dark for 15 min. The pH of the samples was then brought to 1 by adding 12% phosphoric acid in water (1.1% final concentration). 400 µL of binding/wash buffer (100 mM TEAB pH 7.5 in 90% MeOH) were then added to the samples which were then transferred to spin filter columns (Zymo Research). The samples were spun down for 1 min at 1400 x g and washed 4 times with binding/wash buffer. The remaining liquid was removed with an extra centrifugation step. The protein was then digested ON at 37 °C with 1 µg of trypsin freshly dissolved in 50 µL of a 50 mM ammonium bicarbonate solution. The next day the digested peptides were collected by centrifugation at 1400 x g for 1 min. 75 µL of a 0.1% solution of formic acid in water were then added followed by a centrifugation step. Next 75 µL of a solution containing 60% MeCN and 1% formic acid in water was added and the samples were spun down again. The peptides were concentrated to near dryness in a Speedvac (Eppendorf) at 45 °C. Peptide samples were reconstituted in 20-50 µL of 97% water, 3% ACN, 0.05% TFA and analysed on a Orbitrap Eclipse MS.

Q Exactive MS analysis: Resuspended peptides were analysed on a Q Exactive HF Orbitrap MS (Thermo Fisher Scientific). Peptides were resolved on a UltiMate 3000 RSLCnano system set in a trap-elute configuration with a nanoEase M/Z Symmetry C18 100 Å, 5 µm, 180 µm x 20 mm (Waters) trap column and nanoEase M/Z HSS C18 T3 100 Å, 1.8 µm, 75 µm x 250 mm (Waters) analytical column, both kept at 40 °C. Samples were loaded on the trap column for 2 min at a flow rate of 15 µL/min with 99% mobile phase A (0.1% formic acid in H_2O), 1% mobile phase B (0.1% formic in acetonitrile) eluent. The 85 min LC method, using mobile phase A and mobile phase B controlled by a flow sensor at 0.3 µL/min

with average pressure of 400-500 bar (5500-7000 psi), was programmed as gradient with linear increment to 1% B from 0 to 2 min, 5% B at 5 min, 22% B at 55 min, 40% B at 64 min, 90% B at 65 to 74 min and 1% B at 75 to 85 min. The eluent was introduced by electron spray ionisation (ESI) via the nanoESI source (Thermo) using stainless steel Nano-bore emitters (40 mm, OD 1/32", ES542, Thermo Scientific). The QExactive HF was operated in positive mode with data dependent acquisition without the use of lock mass, default charge of 2+ and external calibration with LTQ Velos ESI positive ion calibration solution (88323, Pierce, Thermo) every 5 days to less than 2 ppm. The tune file for the survey scan was set to scan range of 350 – 1400 m/z, 120,000 resolution (m/z 200), 1 microscan, automatic gain control (AGC) of 3e6, max injection time of 100 ms, no sheath, aux or sweep gas, spray voltage ranging from 1.7 to 3.0 kV, capillary temp of 250 °C and an S-lens value of 80. For the data dependent MS/MS measurements the loop count was set to 10 and the general settings were resolution to 15,000, AGC target 1e5, max IT time 50 ms, isolation window of 1.8 m/z, fixed first mass of 120 m/z and normalized collision energy (NCE) of 28 eV. For individual peaks the data dependent settings were 1.00e3 for the minimum AGC target yielding an intensity threshold of 2.0e4 that needs to be reached prior of triggering an MS/MS event. No apex trigger was used, unassigned, +1 and charges >+5 were excluded with peptide match mode preferred, isotope exclusion on and dynamic exclusion of 10 sec. In between experiments, routine wash and quality control runs were done by injecting 5 µl of 10 fmol/µL BSA digest. Raw files were analysed with MaxQuant version 1.6.17.0 and 2.4.9.0 against a proteome FASTA file of *Aspergillus niger* ATCC MYA-4892 / CBS 513.88 / FGSC A1513 from 2007 with 14,069 entries.

Eclipse MS analysis: Resuspended peptides were separated via nanoflow reversed-phase liquid chromatography using a Vanquish Neo UHPLC system setup in a trap-elute configuration. For trapping peptides, a PepMap Neo 5 µm C18, 300 µm x 5 mm trap (Thermo Scientific, 174500) was used which was eluted into an Easy-Spray PepMap Neo 2 µm C18, 75 µm x 500 mm analytical column (Thermo Scientific, ES75500PN) kept at 50 °C with a constant flowrate of 0.3 µL/min. The mobile phase consisted of a mixture of mobile phase A (0.1% FA in ULC-MS grade water (Biosolve) and mobile phase B (0.1% FA in 80% ULC-MS grade acetonitrile (Biosolve), and the separation was performed with a gradient from 6 – 44 % B over 40 min, followed by an increase to 90 % B over 5 min and a column wash with 90 % B for 14 min before column re-equilibration. Peptides were introduced to an Orbitrap Eclipse Tribrid Mass Spectrometer (Thermo Scientific) with a spray voltage of 1500 V. Data was acquired in data-dependent acquisition (DDA) mode with the following scan sequence: MS1 master scan (Orbitrap analysis, 240K resolution, 375-1500 m/z, RF lens 30%, 80 ms maximum injection time) with dynamic exclusion enabled (repeat count 1, exclusion duration 60 s, mass tolerance 10 ppm, dependent scan on single charge state per precursor only). The top precursors were then selected for MS^2 analysis with an intensity threshold of $1.0{\cdot}10^4$ within a 1.7 second duty cycle through quadrupole isolation (1.2 m/z isolation window) followed by higher-energy collisional dissociation in the ion routing multipole (normalized collision energy 30%) and analysis of the resulting fragments in the orbitrap (15K resolution, 100 ms maximum injection time, centroid mode).

Active-site peptide nucleophile identification: The identities of the active-site nucleophile of *An*ManA and *An*Man26A were taken from Uniprot.[10] The sequences of the tryptic peptide containing the active-site nucleophile were obtained with PeptideCutter (Expasy). The m/z of the naked active-site peptide and the m/z of the inhibitor-peptide complex was predicted with Chemdraw (Revity signals). For *An*ManA, the peaks corresponding to the predicted m/z of the target active-site peptide with and without bound inhibitor were identified with the software Xcalibur (Thermo Fisher Scientific) and they were manually assigned. The same was done for the LC-MS/MS fragments. the covalent complexes between inhibitor and active-site tryptic peptide were computationally confirmed with FragPipe v23.1 (Nesvilab). [11] In brief, a shortlist FASTA file was generated by searching the data against the *A. niger* proteome supplemented with a list of common contaminants.[12] With this shortlist FASTA, a labile search was run by modifying the labile-phospho workflow,[13] to account for the non-reducing end sugar

loss observed during MS/MS fragmentation, which resulted in more fragments being assigned in the MS2 spectra. Intact Man-man-*manno*-cyclophellitol **16** produces a mass shift of 500.1746 Da, Man-*manno*-cyclophellitol **15** produces a shift of 338.1213 Da, and both modifications have a fragment remainder modification mass of 176.0685 Da due to the remaining *manno*-cyclophellitol after MS/MS fragmentation. The mass shift caused by methionine oxidation and cysteine alkylation was also accounted for during the labile-search.

Intact protein mass spectrometry

***An*ManA and *An*Man26A**: 10 µM solutions of purified *An*ManA and *An*Man26A in McIlvaine buffer at pH 5 were labelled with 500 µM **16** for 2 hours at 37 °C. After the incubation the buffer was exchanged to 10 mM ammonium acetate wit Micro Bio-Spin 6 columns (Biorad) and the samples very directly injected in the LC-MS. The analysis was performed on a nanoAcquity UPLC system hyphenated by electrospray ionization (ESI) to a Synapt G2Si high resolution TOF mass spectrometer operated with the Masslynx software (all from Waters). Proteins were desalted by reversed phase liquid chromatography on an Acquity UPLC M-Class 300 µm x 50 mm column, packed with BEH C4 material of 1.7µm diameter and 300Å pore size particles with a gradient of 10 min from 10 – 90 % mobile phase B (0.1% formic acid in acetonitrile) in mobile phase A (0.1% FA in H2O) and 5 min equilibration in 10% B at a flow of 2 µl/min. All solvents were of ULC/MS grade quality (Biosolve). Electro-spray ionization (ESI) was done via the nano-spray source with ESI emitters (New Objectives) made of fused silica tubing 360 µm OD x 25 µm ID tapered to 5 ± 0.5 µm (5 nl/cm void volume). The Synapt G2Si mass spectrometer (Waters) was mass calibrated to 5 ppm accuracy with GluFib fragmentation ions and operated in positive, resolution mode using the following settings: source temperature of 80°C, capillary voltage 4.5 kV, nano flow gas of 0.25 Bar, purge gas 250 L/h, trap gas flow 2.0 ml/min, cone gas 100 L/h, sampling cone 25V, source offset 25, trap CE 32V, scan time 3.0 sec, mass range 400-2400 m/z. Lock mass acquiring was done with a mixture of Leu Enk (556.2771) and Glu Fib (785.84265), lockspray voltage 3.5kV. The MaxEnt 1 software was used for mass deconvolution of the charge state envelopes.

***Cj*Man26C, *Cj*Man26A and *Aa*GH113**: *Cj*Man26C, *Cj*Man26A and *Aa*GH113 were diluted to 1 mg/mL in 20 mM pH 7 NaPi buffer. To 20 µL of enzyme in buffer in a PCR tube was added 5 µL of 0.5 mM of the inhibitor. Samples were incubated at 37 °C. At regular intervals, 2 µL of the protein+inhibitor mixture was sampled and analysed as described.[14]

Crystallography

***An*ManA:** Initial crystallization conditions for *An*ManA from AMB3.3 were identified using the sitting-drop vapor-diffusion method at 20 °C. Crystals were obtained from a condition containing 2.5 M ammonium sulfate and 0.1 M Tris pH 7.8 by mixing 100 nL protein solution with 300 nL reservoir solution. Needle-shaped crystals appeared after 4 days. These crystals were used to generate seed crystals using the Hampton Research Seed Bead protocol. To obtain a co-structure of ManA in complex with compound **16**, *An*ManA was incubated with compound **16** (1 mM) for 2 h prior to crystallization. This protein-ligand solution was then used to obtain crystals using the sitting-drop vapor-diffusion method with seeding. The protein-ligand solution, reservoir solution (2.2 M ammonium sulfate and 0.1 M Tris pH 7.8) and seed stock (described above) were mixed, respectively, in a ratio of 100:250:50 nL and incubated at 20 °C. Needle-shaped crystals appeared after approximately 7 days and continued to grow for up to 35 days. Prior to crystal harvesting, a solution containing 2.4 M ammonium sulfate, 0.1 M Tris pH 7.4, and 2 mM compound **16** was added directly to the crystallization drop, and crystals were incubated for 4 h. Crystals were then harvested and flash-cooled in liquid N_2.

X-ray diffraction data were collected at 100 K at beamline MASSIF-3 of the European Synchrotron Radiation Facility. Data were processed using the XIA2 pipeline.[15] The structure of ManA from AMB3.3 in complex with compound **16** was determined by molecular replacement using an AlphaFold-predicted model of ManA (AF-A2QKT4)[16] as the search model in PHASER.[17] The model was refined

through iterative cycles of manual rebuilding in COOT[18] and maximum-likelihood refinement in REFMAC5.[19] Ligand coordinates and restraint parameters for compound **16** were generated using AceDRG.[20] Data collection and refinement statistics can be found in Supplemental table 7. Figures were prepared using PyMOL (Schrödinger, LLC).

***Cj*Man26C:** *Cj*Man26C readily formed crystals as described.[21] Soaking with inhibitors failed to yield a covalent complex, likely due to poor activity in the crystallised state. Thus, 2.25 mg of ChMan26C (50 nmol) in 120 µL of 20 mM MOPS pH 7.0 was mixed with 20 µL of 5 mM inhibitor (100 nmol) in water, giving a final protein concentration of 16 mg/mL. This mixture was incubated at ambient temperature for 1 hour to allow the reaction to proceed to completion before mixing the protein solution 1:1 in a sitting drop configuration with 2% tacsimate, 0.1 M sodium citrate tribasic dihydrate pH 5.6 and 16% PEG3350. A single large crystal grew within 48 hours. This was fished, flash-frozen in LN2 and shot at Diamond Light Source (UK). Data were collected at a wavelength of 0.8 Å out to a maximum resolution of 1.16 Å. The images were processed with XDS[22] and DIALS[23], respectively, as incorporated in Xia2 pipeline[24], and scaled with AIMLESS. The structure was solved using the CCP4 package[25] using MOLREP[26] with PDB entry 1GVY as an initial model and refined with cycles of REFMAC5[19] alternating with manual model correction in COOT.[27] The dictionaries for the ligand and its covalent linkage were created in JLigand[28], and the ligand was built using COOT. Data collection and refinement statistics can be found in Supplemental table 8. Figures were prepared using PyMOL (Schrödinger, LLC).

Irreversible inhibition kinetics

***Cj*Man26C and *Cj*Man26A**: Inhibition kinetics were measured using the residual activity method. Enzymes in 100 mM pH 7 NaPi buffer at 40 µg/mL were mixed 1:1 with inhibitor solutions ranging from 400 down to 0 µM and incubated at 37 °C. At various time points (2 minutes, 5 minutes, 10 minutes, 20 minutes, 30 minutes, 40 minutes, 50 minutes, 60 minutes), samples were collected using a multichannel pipette and diluted 10-fold into 2 g/L cGM in 20 mM pH 7 NaPi buffer, dramatically reducing the rate of inhibition and initiating the enzymatic hydrolysis. The hydrolysis reaction was incubated for 15 minutes at 37 °C, then stopped by mixing 1:1 with BCA reagent (250 mM Na_2CO_3, 140 mM $NaHCO_3$, 2.5 mM bicinchoninic acid disodium salt, supplemented with 1.25 mM $CuSO_4$ and 2.5 mM L-serine before use). Colour, as quantified by measuring absorbance at 563 nm, was developed by heating samples to 80 °C for 10 minutes. Reducing ends were quantified by comparison to a glucose standard series ranging from 0 to 500 µM. Activity at each time point was normalised to an uninhibited control to account for background activity loss. Inhibition was modelled as a pseudo-first order exponential decay and apparent decay constants (kapp) were then plotted against inhibitor concentration and fit with either a linear model forced through 0 (where slope = ki/KI) or a site saturation model (Kitz-Wilson) to extract ki and KI as well as ki/KI values.

ABP analysis of pH optimum

***Aspergillus niger* MA234.1 grown on 1 1% kF**: Aliquots of the 72h secretome of MA234.1 grown on 1% kF were exchanged to McIlvaine buffer at pH 2.2, 3, 4, 5, 6, 7 and 8. This process was performed with Vivaspin 10.000 MWCO centrifugal filter units (Sartorius). 1.5 µL of a 50 µM solution of compound in DMSO was added to 15 µL of each sample. The ABP labelling reaction was carried out at 37 °C with different incubation times. The zero-time point was kept on ice after the addition of the probe. After the incubation 4x Laemmli buffer was added and samples were heated to 95 °C for 5 min. Samples were separated by SDS-PAGE on 12.5% polyacrylamide gels (Biorad). Gels were imaged with the Cy5 laser set in the Typhoon 5 laser scanner (GE Healthcare). After imaging the gels were stained with Coomassie brilliant blue R250 (Biorad) and acquired with a Chemidoc system (Biorad). Band integrated density was quantified with the software imageJ (National Institute of Health). Integrated density values of the 2h timepoints were used to plot line graphs with the software Graphpad Prism.

***Cellvibrio japonicus* Ueda 107 grown on 0.25% cGM**: 50 µL of *Cellvibrio japonicus* Ueda 107 lysate prepared from cells grown on 0.25% w/v cGM in MOPS minimal medium was mixed 1:1 with 100 mM

succinic acid-phosphoric acid-glycine (SPG) buffer titrated to pH values of 4, 5, 6, 7, 8, 9, or 10 with sodium hydroxide and supplemented with probe to a final concentration of 2 µM. Lysates were incubated at 30 °C for 1 hour with a heated lid to prevent evaporation. Enzyme labeling was stopped by addition of Laemmli buffer to a final concentration of 1x and heating the samples to 95 °C for 5 minutes. Samples were separated through a 10% SDS-PAGE gel (Bio-Rad) at 200 V and imaged for Cy5 fluorescence as above.

ABP analysis of temperature stability

***Aspergillus niger* MA234.1 grown on 1 1% kF**: The 72h secretome of MA234.1 grown on 1% kF was incubated at 40 °C, 50 °C, 60 °C, 70 °C and 80 °C for up to 2 hours. For each time point, at the end of each incubation the respective sample was snap-cooled on ice. The 0' sample was directly kept on ice. At the end of the 2 hours incubation a 50 µL solution of **1** in DMSO was added to a 10x larger volume of the pre-heated secretome samples. The ABP labelling reaction was carried out for 1h at 37 °C. 4x Laemmli buffer was added and samples were heated to 95°C for 5 min. Samples were separated by SDS-PAGE on 12.5% polyacrylamide gels (Biorad). Gels were imaged with the Cy5 laser set in the Typhoon 5 laser scanner (GE Healthcare). After imaging the gels were stained with Coomassie brilliant blue R250 (Biorad) and acquired with a Chemidoc system (Biorad). Band integrated density was quantified with the software imageJ (National Institute of Health) and used to plot line graphs with the software Graphpad Prism.

***Cellvibrio japonicus* Ueda 107 grown on 0.25% cGM**: 50 µL of *Cellvibrio japonicus* Ueda 107 lysate prepared from cells grown on 0.25% w/v cGM in MOPS minimal medium was supplemented with probe to a final concentration of 2 µM. Lysates were incubated for 1 hour at temperatures ranging from 30 to 60 °C, with a heated lid to prevent evaporation. Enzyme labeling was stopped by addition of Laemmli buffer to a final concentration of 1x and heating the samples to 95 °C for 5 minutes. Samples were separated through a 10% SDS-PAGE gel (Bio-Rad) at 200 V and imaged for Cy5 fluorescence as above.

Molecular Modelling

Calculation of free energy landscape: The free-energy landscape of *manno*-cyclophellitol in the gas phase was obtained using first-principles molecular dynamics based on Density Functional Theory within the Car–Parrinello framework.[29] The molecule was placed in a 13Å-wide cubic simulation cell under isolated-molecule conditions. A fictitious electronic mass of 700 au and an integration step of 0.12 fs were employed. Kohn–Sham orbitals were represented with a plane-wave basis set using a kinetic-energy cutoff of 70 Ry, and norm-conserving pseudopotentials were used following the Troullier–Martins protocol.[30] The Perdew–Burke–Ernzerhof (PBE) generalised-gradient functional[31] was selected because of its previously demonstrated accuracy for modelling isolated carbohydrate analogues.[32-35] Conformational free-energy surfaces were explored through metadynamics simulations,[36,37] using the Cremer–Pople puckering angles θ and ϕ as collective variables.[38] Gaussian bias potentials of 0.6 kcal·mol^{-1} were initially applied and reduced to 0.3 kcal·mol^{-1} after 60 ps to enhance late-stage convergence for this system. A Gaussian width of 0.1 rad and a deposition interval of 250 steps (first 60ps) or 500 steps (after 60ps) were used. The simulation was conducted for 323 ps, at which point the landscape was considered converged based on the stability of relative free-energy differences between the major minima (standard deviation < 1 kcal·mol^{-1} over the final 20 ps).

Supporting Figures

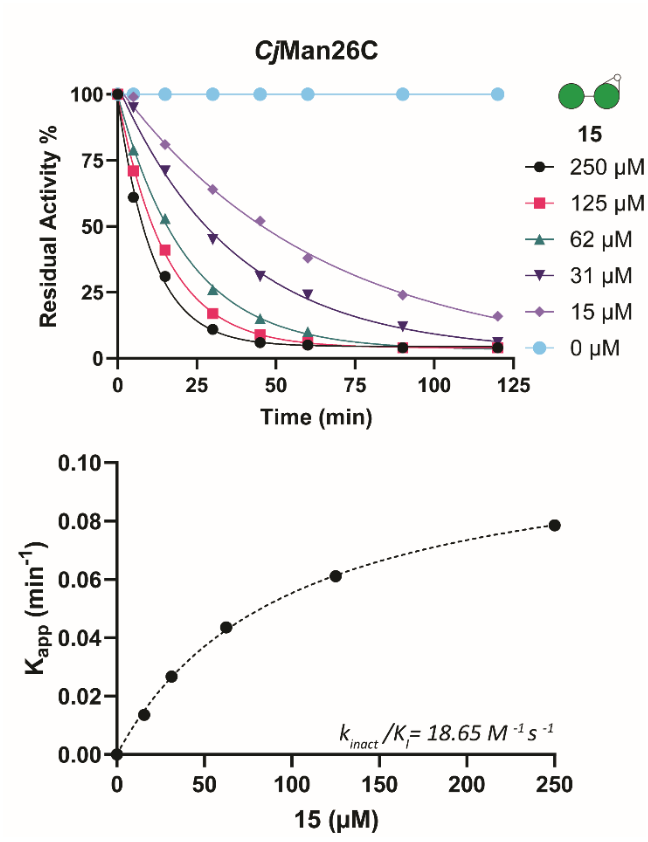

Supplemental figure 1. Irreversible inhibition kinetics of *Cj*Man26C with inhibitor **15** using carob galactomannan as substrate. Residual activity plot vs. time with increasing concentrations of **15** and plot of apparent decay constant (Kapp).

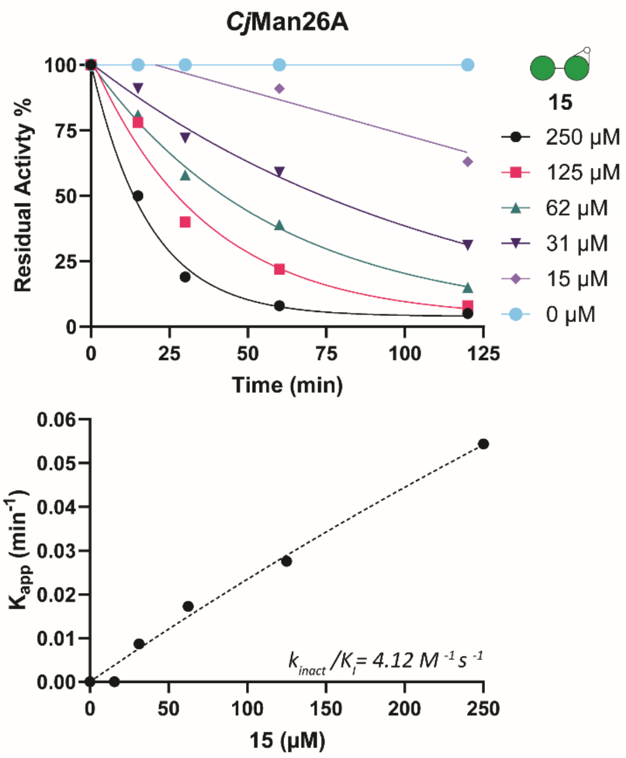

Supplemental figure 2. Irreversible inhibition kinetics of *Cj*Man26A with inhibitor **15** using carob galactomannan as substrate. Residual activity plot vs. time with increasing concentrations of **15** and plot of apparent decay constant (Kapp).

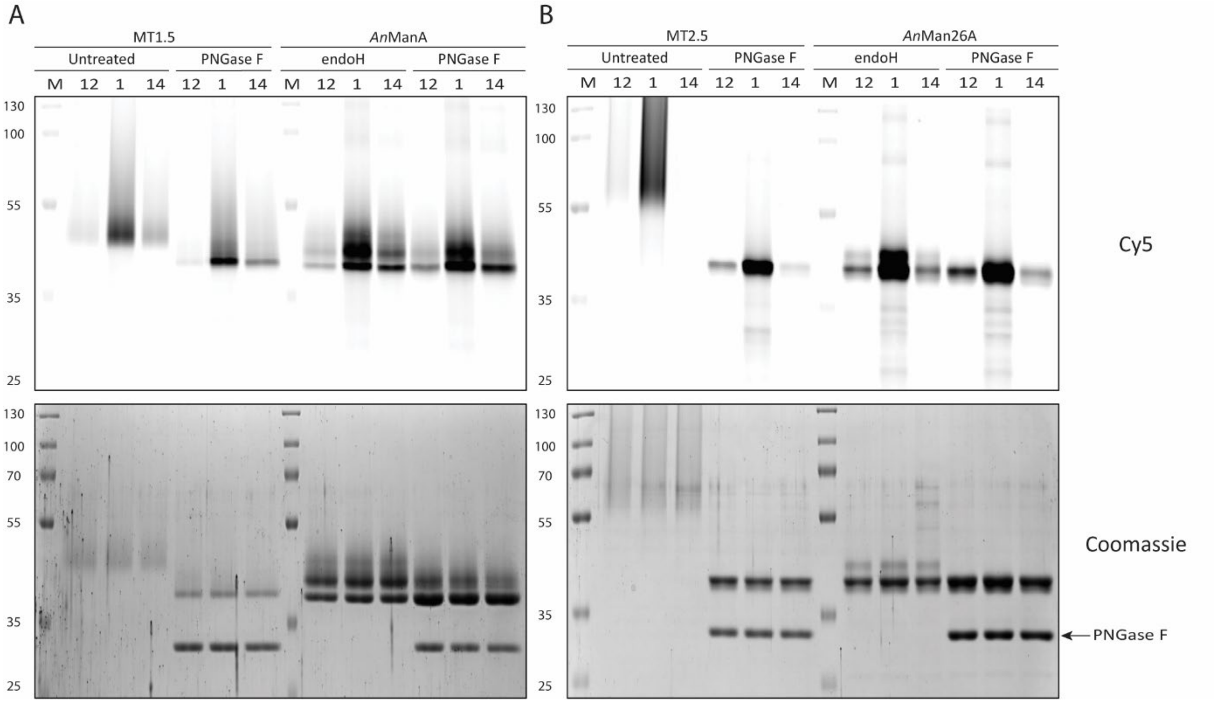

Supplemental figure 3. A. Cy5 signal (upper panel) and Coomassie strain (lower panel) of SDS-gel after ABP labelling on the secretome of the ManA overexpressing strain MT1.5 and purified ManA. The purified mannanase was treated with endoH as part of the purification protocol. Both the secretome and the purified protein were labelled before and after treatment with PNGase F. The fluorescent probes employed are Cy5-Man-*manno*-cyclophellitol **12**, Cy5-Man-man-*manno*-cyclophellitol **1** and Cy5-Man-*manno*-cyclophellitol **14**. **B.** Cy5 signal (upper panel) and Coomassie strain (lower panel) of SDS-gel after ABP labelling on the secretome of the Man26A overexpressing strain MT2.5 and purified Man26A. The labelling was performed with the same conditions as A. The purified mannanase was treated with endoH as part of the purification protocol.

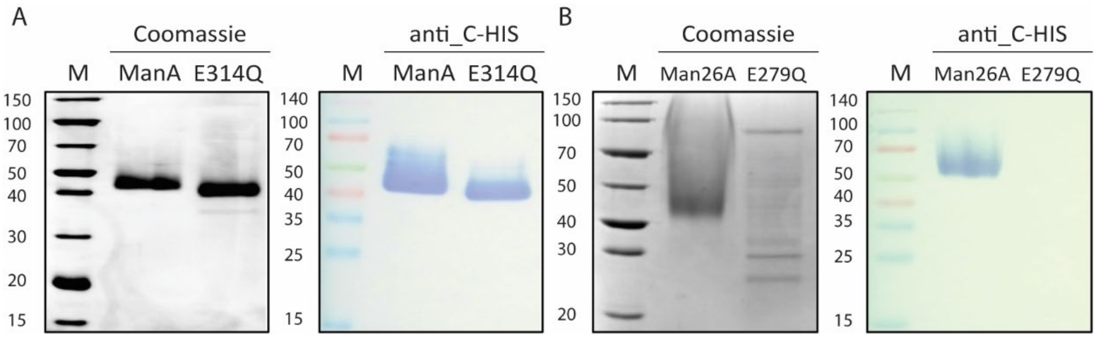

Supplemental figure 4. A. Coomassie staining and anti-C-terminal HIS-tag Western blot of the secretomes of the MT1.5 strain overexpressing *An*ManA and of the MA1182.5 overexpressing the active-site mutant of ManA E314Q. **B.** Coomassie staining and anti-C-terminal HIS-tag Western blot of the secretomes of the MT2.5 strain overexpressing *An*Man26A and of the MA1183.16 overexpressing the active-site mutant of Man26A E279Q.

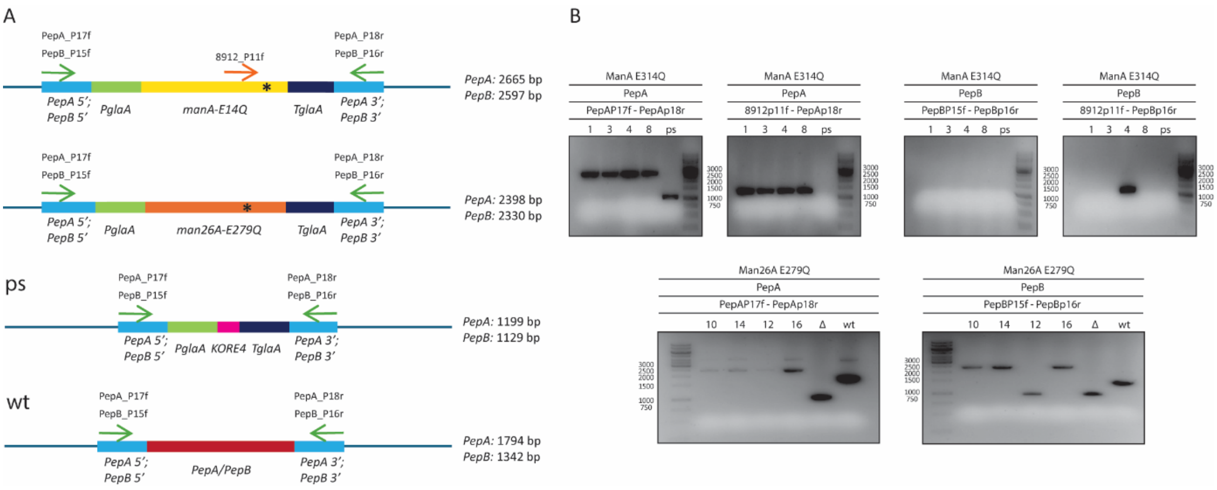

Supplemental figure 5. A. Scheme of the *pepA* and *pepB loci* of the active-site mutant strains of *manA* (MA1182.5) and *man26A* (MA1183.16) of *A. niger*. Parental strain (ps) shows the *pepA* and *pepB loci* of the background strains used for the transformation, which contain glucoamylase landing sites. Wild type (wt) shows the original *pepA* and *pepB loci*, which contain the *pepA* and *pepB* coding sequence. The schemes show the name and position of the primers used for the dPCR and the amplicon size. **B.** DNA electrophoresis of the dPCR of the *pepA* and *pepB loci* of the active-site mutant strains shown in A.

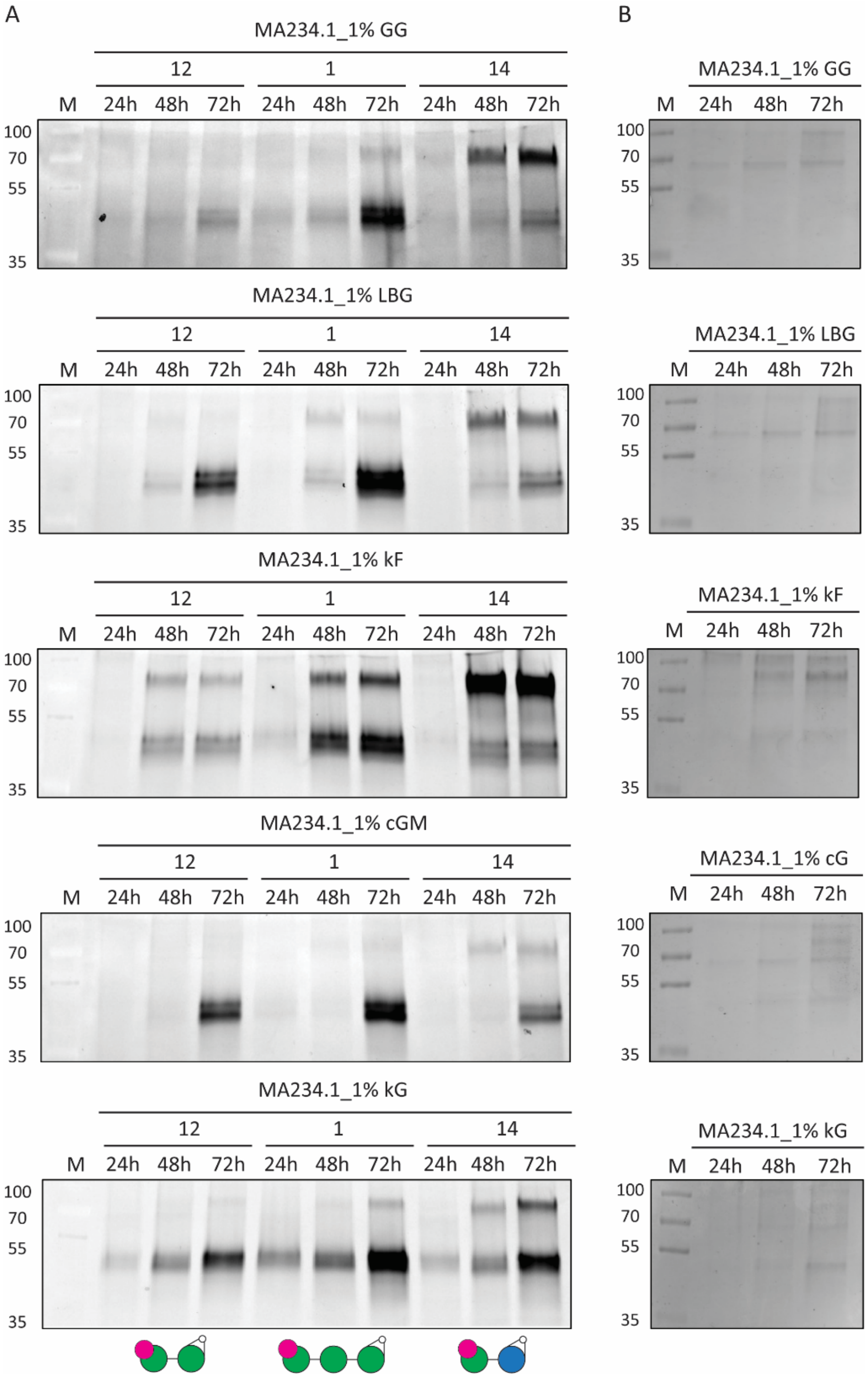

Supplemental figure 6. A. Cy5 signal of SDS-page gel of secretomes of *A. niger* MA234.1 grown on different mannanase-inducing carbon sources: guar gum (GG), locust bean gum (LBG), konjac flour (kG), carob galctomannan (cGM) and konjac glucomannan (kG). Secretome samples were collected at 24, 48 and 72 hours after biomass transfer. The secretomes were treated with Cy5-Man-*manno*-cyclophellitol **12**, Cy5-Man-man-*manno*-cyclophellitol **1** and Cy5-Man-*manno*-cyclophellitol **14**. **B.** Coomassie staining of the secretomes shown in panel A. Per each carbon source the same three secretome samples were labelled with the three probes, consequently the total protein content of each sample is shown only once.

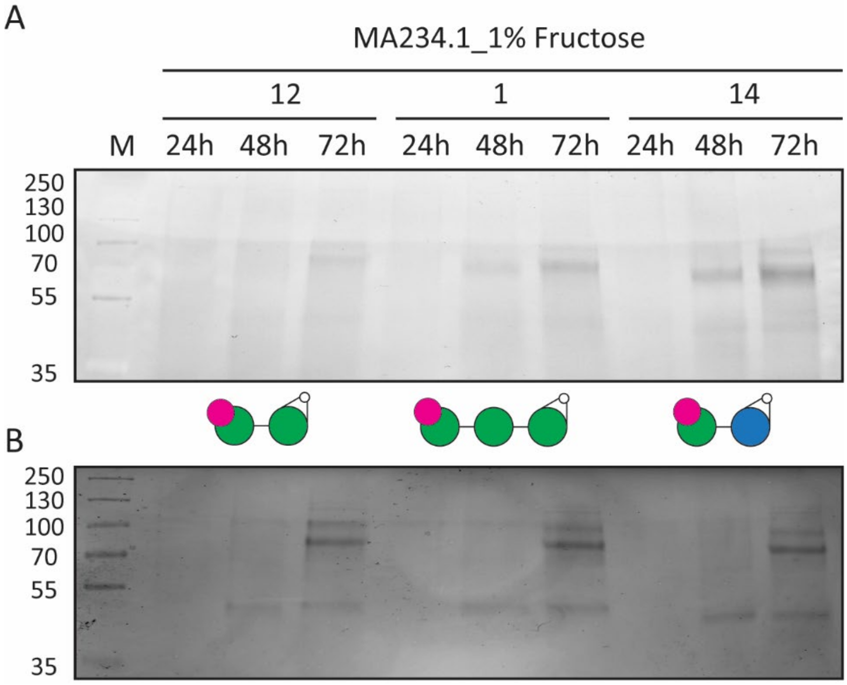

Supplemental figure 7. A. Cy5 signal of SDS-page gel of secretomes of *A. niger* MA234.1 grown on Fructose. Secretome samples were collected at 24, 48 and 72 hours. The secretomes were treated with Cy5-Man-*manno*-cyclophellitol **12**, Cy5-Man-man-*manno*-cyclophellitol **1** and Cy5-Man-*manno*-cyclophellitol **14**. **B.** Coomassie staining of the secretomes shown in panel A.

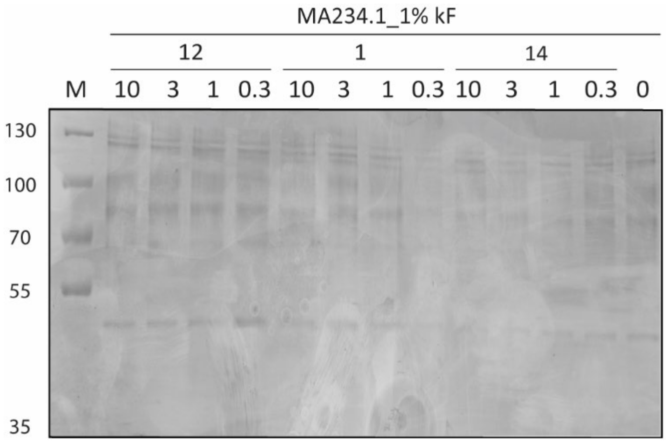

Supplemental figure 8. Coomassie stain of the concentration range ABP labelling on the secretome of *A. niger* MA234.1 grown on 1% konjac flour (kF) for 72h. The fluorescent probes employed are Cy5-Man-*manno*-cyclophellitol **12**, Cy5-Man-man-*manno*-cyclophellitol **1** and Cy5-Man-*manno*-cyclophellitol **14.**

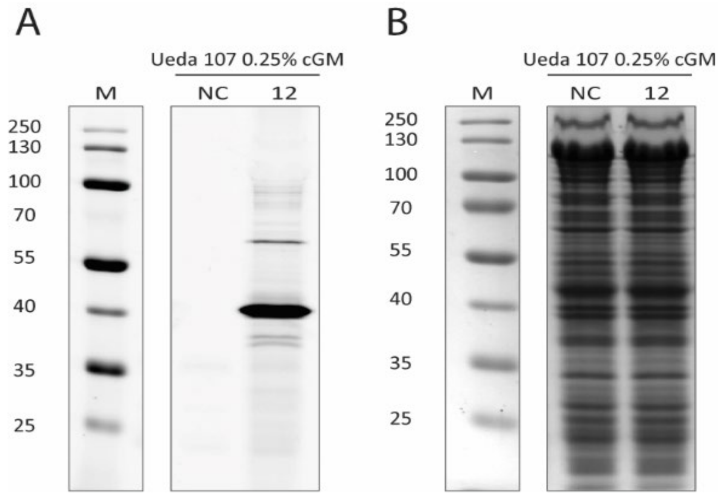

Supplemental figure 9. A. Cy5 fluorescent signal of SDS-PAGE gel of lysate of *C. japonicus* Ueda 107 grown on cGM before (NC) and after treatment with Cy5-Man-*mann*-cyclophellitol **12**. **B.** Coomassie staining of the gel shown in panel A.

Supplemental table 4. List of protein groups identified in the pulldown with probe **13** on the secretome of *A. niger* MA234.1 grown on konjac flour. LFQ = label free quantification intensity; NC = negative control.

Uniprot ID	Protein Name	MW (kDa)	Score	Avg_log2_LFQ	NC_Avg_log2_LFQ	Avg_peptides	NC_Avg_peptides
A2Q8R2	ATP-dependent DNA helicase mph1	125.66	5.73	0.00	0.00	0.33	0
A2QAI7;A2QPG2	1,4-beta-D-glucan cellobiohydrolase B	56.20	105.07	27.33	0.00	3.00	0
A2QEJ9	alpha-galactosidase B	48.83	6.80	0.00	0.00	0.67	0
A2QKT4	mannan endo-1,4-beta-mannosidase A	41.25	60.08	28.13	0.00	2.00	0
A2QNW5	rRNA-processing protein efg1	35.57	6.15	0.00	0.00	0.33	0
A2R3L3	Aspergillopepsin-1	41.28	12.58	21.19	0.00	2.00	1
A2Q7F2	Contig An01c0030	102.97	6.64	0.00	0.00	0.33	0
A2Q9F7	Contig An01c0270	45.83	5.76	0.00	0.00	0.00	1
A2Q9N9	Contig An01c0290	157.46	5.95	0.00	0.00	0.67	1
A2Q9V3	Contig An01c0310	44.68	5.74	0.00	0.00	0.33	0
A2QAS5	Contig An01c0370	34.86	5.73	19.66	0.00	0.67	0
A2QAW3	amidase	65.03	5.76	0.00	0.00	0.33	0
A2QB77	Contig An01c0450	64.56	6.19	29.27	31.32	1.00	1
A2QC23	Vacuolar proton pump subunit B	56.71	5.73	0.00	0.00	1.00	1
A2QC71	Uncharacterized protein	62.95	5.96	0.00	0.00	0.33	0
A2QCG5	Contig An02c0090	51.77	6.10	0.00	0.00	1.00	1
A2QES4	Contig An02c0390	41.25	5.77	0.00	0.00	0.00	1
A2QFG4	Secretory pathway Ca2+ATPase pmrA- Aspergillus niger	110.79	5.72	0.00	0.00	0.33	0
A2QFL8	Uncharacterized protein (Fragment)	8.39	5.77	0.00	0.00	0.33	0
A2QH61	Contig An03c0180	74.63	5.73	0.00	0.00	0.33	0
A2QHE1	Glucoamylase	68.31	6.12	0.00	0.00	1.00	0
A2QI92	Contig An04c0110	104.52	5.74	0.00	0.00	0.33	1
A2QJR6	Contig An04c0250	84.84	12.41	21.67	0.00	1.00	0
A2QJX2	Uncharacterized protein	13.12	5.73	18.83	0.00	1.00	0
A2QK47	Uncharacterized protein	18.27	5.75	0.00	0.00	0.00	1
A2QK50	Uncharacterized protein	14.79	5.74	0.00	0.00	0.33	1
A2QKG6	Uncharacterized protein	32.68	7.04	25.47	19.65	1.00	1
A2QLU5	Contig An06c0110	48.40	5.72	0.00	0.00	0.00	1
A2QLZ3	Contig An07c0010	53.32	5.74	0.00	0.00	0.00	1
A2QN01	Contig An07c0100	181.48	5.73	0.00	0.00	0.33	0
A2QN08	Contig An07c0100	129.03	6.15	0.00	0.00	0.67	0
A2QN24	Contig An07c0100	32.47	5.75	0.00	0.00	0.67	1
A2QQ70	non-specific serine/threonine protein kinase	78.98	5.76	17.38	0.00	0.67	0
A2QQH8	Contig An08c0100	76.81	5.73	21.69	0.00	0.67	0
A2QRC0	Contig An08c0130	32.37	61.12	0.00	0.00	1.00	1
A2QRX4	Contig An08c0170	102.99	5.95	0.00	0.00	1.00	0
A2QRZ8	Contig An08c0200	63.47	5.76	17.86	23.46	0.33	1
A2QSS1	Uncharacterized protein	52.82	5.77	0.00	0.00	1.00	1
A2QT53	Contig An09c0040	160.76	5.98	18.71	0.00	0.67	0
A2QTJ2	Uncharacterized protein	11.43	5.98	0.00	0.00	0.33	0
A2QU98	Contig An09c0140	71.52	5.72	0.00	0.00	0.33	0

A2QUT6	Contig An09c0220	61.91	5.73	16.14	0.00	0.67	0
A2QW02	alpha-amylase	55.24	6.25	21.50	0.00	1.00	0
A2QWA4	Contig An11c0170	175.22	5.72	0.00	0.00	0.33	0
A2QWJ5	Contig An11c0210	149.51	5.79	0.00	0.00	0.67	0
A2QX57	Uncharacterized protein	40.84	5.75	23.05	0.00	0.67	0
A2QXA3	Contig An11c0270	147.74	5.73	0.00	0.00	0.33	0
A2QZQ9	Contig An12c0160	66.14	5.74	0.00	0.00	0.67	1
A2QZZ0	Contig An12c0200	58.60	5.76	23.10	24.07	1.00	1
A2R0D5	Contig An12c0280	34.76	5.73	0.00	0.00	0.67	1
A2R0Q9	Contig An12c0330	74.61	5.76	18.72	0.00	0.67	0
A2R1M5	Contig An13c0060	89.18	5.96	0.00	0.00	0.33	1
A2R1P3	Contig An13c0060	60.96	5.96	0.00	0.00	0.67	1
A2R2T7	Contig An14c0090 (Fragment)	392.44	6.07	0.00	0.00	0.00	1
A2R3A2	Contig An14c0130	159.45	5.95	0.00	0.00	0.00	1
A2R3B9	Contig An14c0130	153.30	5.73	19.95	0.00	0.67	0
A2R406	Contig An14c0190	61.75	5.76	0.00	0.00	0.33	0
A2R467	Contig An14c0200	31.51	5.75	0.00	0.00	0.33	0
A2R4C6	Uncharacterized protein	31.06	5.75	0.00	0.00	0.00	1
A2R500	Contig An15c0100	58.10	6.30	0.00	0.00	1.00	1
A2R5J8	Uncharacterized protein	34.48	5.84	18.51	22.02	0.67	1
A2R5X1	Contig An15c0200	38.76	5.73	0.00	0.00	0.00	1
A2R634	Contig An15c0220	149.28	5.73	19.19	22.80	0.33	1
A2R7K4	Contig An16c0140	110.36	5.73	0.00	0.00	0.33	0
A2R7V0	Contig An16c0170	39.45	5.74	19.76	23.41	0.33	1
A2R9J1	Uncharacterized protein	47.23	6.05	23.23	0.00	0.67	0
A2RBG2	Autophagy-related E2-like conjugation enzyme atg3	16.48	6.10	0.00	0.00	0.67	1
A5AAA6	Contig An02c0200	87.88	5.74	0.00	0.00	0.00	1
A5AB05	Uncharacterized protein	43.52	6.28	23.60	0.00	1.00	0
A5AB92	Contig An08c0230	71.14	5.72	0.00	0.00	0.00	1
A5ABR0	Mediator of RNA polymerase II transcription subunit 12	174.67	5.75	0.00	0.00	0.33	0
E2PSX5	Contig An17c0060	53.54	5.73	0.00	0.00	1.00	1

Supplemental table 5. List of protein groups identified in the pulldown with probe **13** on the lysate of *C. japonic*us Ueda 107 grown on carob galactomannan. emPAI = exponentially modified protein abundance index.

Accession	Description	Score	Mass (Da)	Num. of matches	Num. of sequences	emPAI
B3PBJ0	Oxaloacetate decarboxylase alpha subunit C. japonicus (strain Ueda107)	167	71007	8	8	0.61
B3PF55	Cellulase, putative, cel5C C. japonicus	147	46018	8	8	1.08
B3PK23	Elongation factor Tu C. japonicus (strain Ueda107)	72	49316	3	3	0.29
B3PB97	Putative TonB-dependent receptor C. japonicus (strain Ueda107)	58	120762	4	4	0.15
B3PK36	30S ribosomal protein S10 C. japonicus (strain Ueda107)	53	13758	2	2	0.82
B3PK47	50S ribosomal protein L14 C. japonicus (strain Ueda107)	52	16376	2	2	0.66
B3PK42	50S ribosomal protein L22 C. japonicus (strain Ueda107)	46	15572	3	2	0.71
B3PK29	50S ribosomal protein L7/L12 C. japonicus (strain Ueda107)	44	15910	2	2	0.69

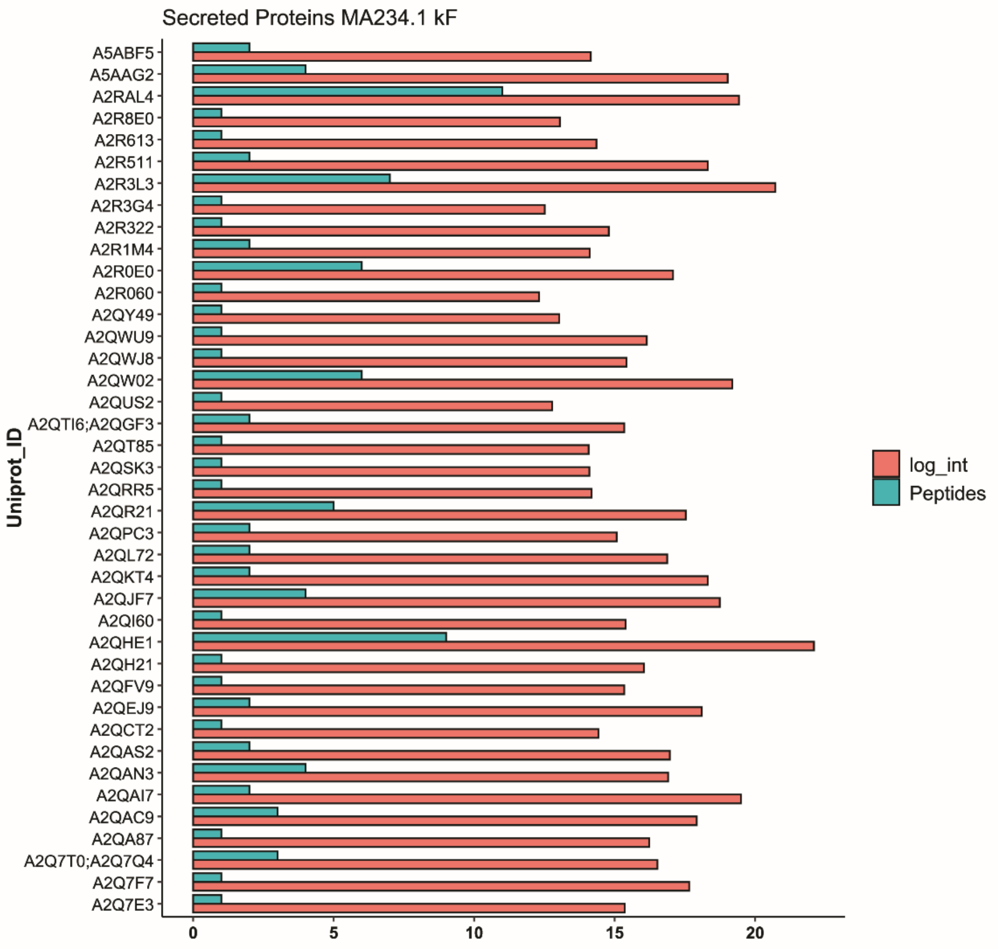

Supplemental figure 10. Qualitative total protein LC-MS identification of the secretome of *A. niger* MA234.1 grown of konjac flour for 72 hours after transfer. The graph reports the unique peptides (Peptides) and the log_{10} intensity (log_int) produced by MaxQuant. *An*Man26A (Uniprot ID: A2R6F) was not detected in the secretome. Unidentified protein reported as Genomic Contig were excluded from the dataset. The complete list of protein groups is provided in Supplemental table 6. A2QKT4 = *An*ManA; A2R8E0 = *An*ManF; A2QAI7 = *An*CbhB; A2QPC3 = *An*EglB

Supplemental table 6. Complete protein group list obtained from total protein identification of the secretome of *A. niger* MA234.1 grown on konjac flour.

Uniprot ID	Protein Name	MW (kDa)	Score	Log10_Intensity
A2QAI7	1,4-beta-D-glucan cellobiohydrolase B	56.20	59.39	19.49
A2QAN3	Beta-galactosidase A	109.71	90.92	16.90
A2QAS2	mannosyl-oligosaccharide alpha-1,2-mannosidase 1B	55.93	15.44	16.96
A2QEJ9	alpha-galactosidase B	48.83	17.94	18.10
A2QFV9	alpha-L-arabinofuranosidase axhA	35.84	86.19	15.34
A2QH21	glucan endo-1,3-beta-glucosidase eglC	46.35	15.90	16.04
A2QKT4	mannan endo-1,4-beta-mannosidase A	41.25	53.26	18.31
A2QL72	alpha-galactosidase A	59.21	58.58	16.87
A2QL84	beta-galactosidase C	108.64	7.18	0.00
A2QPC3	endo-beta-1,4-glucanase B	36.56	12.19	15.07
A2QPG2	1,4-beta-D-glucan cellobiohydrolase A	48.25	6.28	0.00
A2QRR5	Histone H3	15.33	21.70	14.18
A2QT85	arabinan endo-1,5-alpha-L-arabinosidase A	34.48	8.52	14.07
A2QWU9	Beta-mannosidase A	104.03	21.61	16.14
A2QY49	Histone H2B	15.00	6.20	13.03
A2R060	exopolygalacturonase X	47.29	7.80	12.31
A2R0E0	Extracellular exo-inulinase inuE	59.15	83.15	17.08
A2R2S6;A2R2S5	alpha-galactosidase D	71.23	41.70	15.55
A2R3G4	rhamnogalacturonase B	57.93	6.30	12.51
A2R3L3	Aspergillopepsin-1	41.28	323.31	20.72
A2R4K5	rRNA biogenesis protein rrp36	42.50	6.21	0.00
A2R511	alpha-L-arabinofuranosidase B	52.51	184.34	18.31
A2RAL4	beta-glucosidase A	93.23	186.16	19.42
A5AAG2	arabinan endo-1,5-alpha-L-arabinosidase C	34.05	208.40	19.02
A5ABF5	beta-glucosidase M	82.12	48.12	14.15
A2Q7E3	Aspergillopepsin-1	42.76	8.95	15.36
A2Q7F7	Proteinase aspergillopepsin II-Aspergillus niger	29.98	42.12	17.65
A2Q7S3	Contig An01c0070	70.07	51.28	12.06
A2Q7T0;A2Q7Q4	Catalase	80.50	43.62	16.52
A2Q913	Contig An01c0220	19.60	6.18	14.49
A2QA87	ribonuclease T2	29.21	13.29	16.23
A2QAC9	Glycosidase	39.89	60.29	17.92
A2QAR3	Contig An01c0370	98.80	71.04	18.34
A2QBE3	Contig An01c0480	49.68	133.76	14.86
A2QC72	DNA polymerase	166.30	-2.00	0.00
A2QCT2	Carboxypeptidase	58.13	13.43	14.42
A2QCY4	Contig An02c0150	54.02	12.58	0.00
A2QE24	Contig An02c0270	44.14	49.72	17.04
A2QF42	Lysophospholipase	69.41	6.27	0.00
A2QF95	Contig An02c0450	75.52	26.48	14.52
A2QFW4	Contig An03c0040	73.12	11.66	13.88
A2QFW8	Contig An03c0040	47.19	27.08	17.01
A2QH12	Contig An03c0160	62.60	45.54	15.34

A2QH44	Contig An03c0180	27.04	7.13	14.83
A2QHE1	Glucoamylase	68.31	323.31	22.09
A2QHW0	Contig An04c0070	41.05	72.54	15.45
A2QI60	Glutathione S-transferase kappa	25.32	6.22	15.39
A2QIR6	Contig An04c0140	10.10	6.78	0.00
A2QJF7	alpha-glucosidase	108.91	105.42	18.74
A2QJY8	Contig An04c0280	58.98	40.36	16.66
A2QL74	Contig An06c0020	64.49	31.17	0.00
A2QM98	Contig An07c0020	10.00	47.23	15.37
A2QMD7	Contig An07c0020	32.72	6.41	13.79
A2QMJ7	Contig An07c0050	59.76	24.90	14.20
A2QP32	Carboxypeptidase	59.54	12.99	0.00
A2QPM8	Contig An07c0380	69.35	83.50	13.12
A2QR21	Endoprotease Endo-Pro-Aspergillus niger	58.71	70.97	17.53
A2QR36	Contig An08c0130	62.13	40.21	16.03
A2QRR3;A2QCZ0	Histone H4	11.37	6.29	13.76
A2QS54	Contig An08c0220	86.27	13.32	14.27
A2QSH9	Contig An08c0280	47.61	52.55	15.49
A2QSK3	3-phytase	52.49	11.72	14.10
A2QT39	1,3-beta-glucanosyltransferase	56.76	17.23	0.00
A2QTI6;A2QGF3	Beta-hexosaminidase	67.74	18.69	15.34
A2QUK3	Sulphydryl oxidase Sox from patent EP565172-A1-Aspergillus niger	43.50	26.57	0.00
A2QUS2	Heat shock protein sspB-Aspergillus niger	79.78	9.09	12.78
A2QV29	Contig An10c0050	43.52	185.07	16.95
A2QVU0	Contig An11c0090	63.74	6.29	0.00
A2QW02	alpha-amylase	55.24	323.31	19.19
A2QW80	Endoplasmic reticulum chaperone BiP	73.46	7.07	0.00
A2QWJ8	Uncharacterized protein	76.30	6.18	15.42
A2QXG2	Contig An11c0300	36.89	18.06	14.78
A2QYN8	Contig An12c0060	53.88	79.02	14.33
A2QZ72	Carboxylic ester hydrolase	58.36	8.33	0.00
A2QZS1	Contig An12c0170	62.93	21.19	0.00
A2R129	Contig An12c0380	65.89	6.62	0.00
A2R1M4	Purple acid phosphatase	67.27	18.98	14.11
A2R2K9	Contig An14c0040	57.96	14.88	16.39
A2R2Z3	Contig An14c0100	65.70	38.51	14.13
A2R312	Contig An14c0110	26.24	15.28	13.98
A2R322	Endoglucanase A eglA-Aspergillus niger	25.87	7.22	14.79
A2R3N5	Triosephosphate isomerase	26.91	10.29	0.00
A2R4I7	Contig An15c0040	41.61	35.25	15.53
A2R4X5;A2QL34	Contig An15c0100	63.61	7.71	0.00
A2R613	Aspergillopepsin-1	40.20	10.21	14.35
A2R645	Contig An15c0220	90.40	6.53	0.00
A2R6F8	Contig An15c0250	21.82	7.93	0.00
A2R6N9	Contig An16c0020	26.87	11.24	13.33
A2R6P5	Contig An16c0020	33.52	7.86	0.00

A2R707	Contig An16c0070	133.35	6.28	16.51
A2R709	Contig An16c0070	31.69	19.99	0.00
A2R8E0	Endo-beta-1,4-mannanase F	45.25	35.88	13.05
REV__A5AAH3	Contig An02c0310	35.93	6.24	17.00
A5AAV0	Contig An07c0220	102.82	6.27	15.90
A5AAX7;A2RBC8	Contig An07c0220	28.97	6.70	12.33
A5ABC8	Contig An08c0230	51.65	30.77	16.50
E2PSR4	Methylated-DNA--protein-cysteine methyltransferase	257.45	7.17	0.00

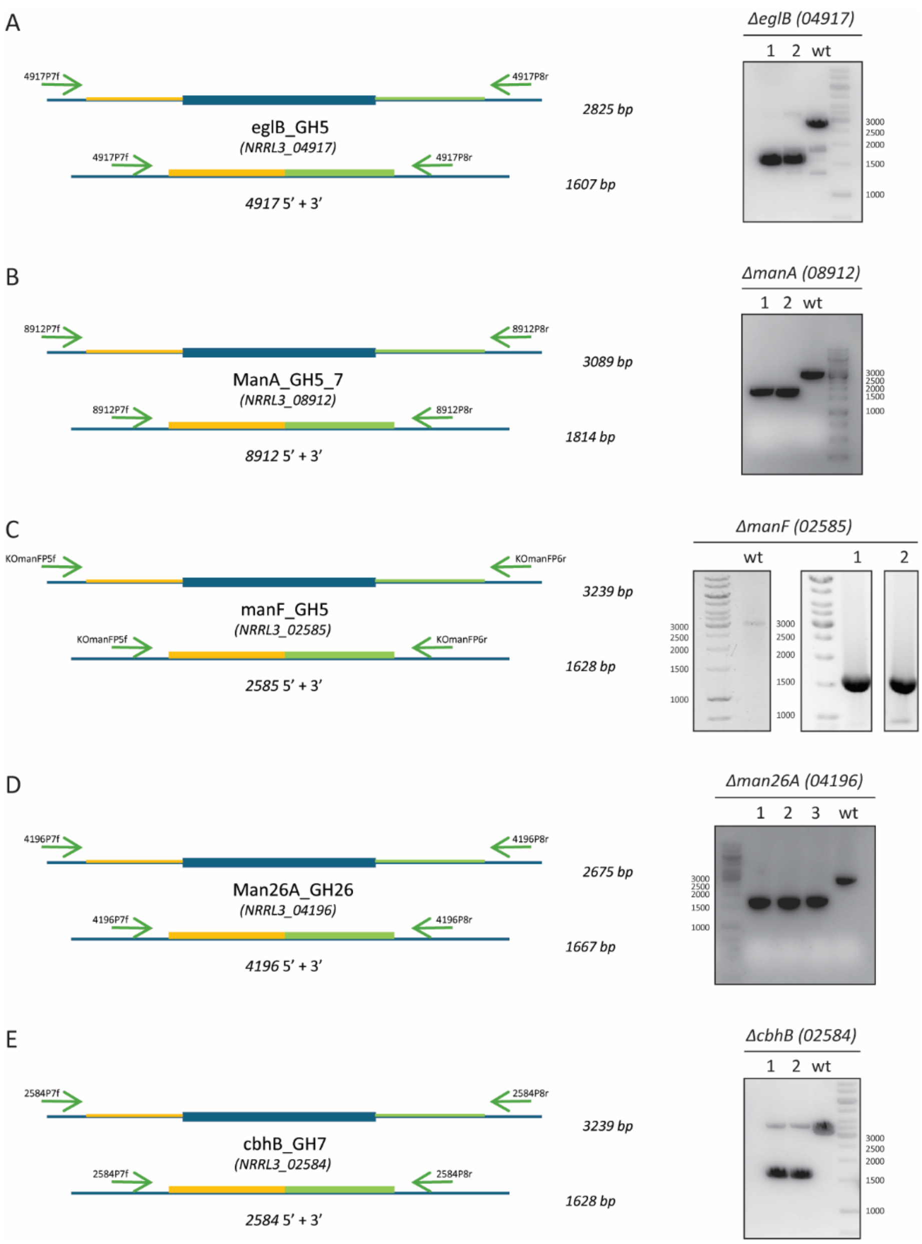

Supplemental figure 11. A. Schematic overview and DNA electrophoresis of the diagnostic PCR (dPCR) of the EglB knockout strain MA1116.1 **B.** Schematic overview and DNA electrophoresis of the dPCR of the ManA knockout strains MA1178.5 and MA1114.1. **C.** Schematic overview and DNA electrophoresis of the dPCR of the manF knockout strain AMB4.4. **D.** Schematic overview and DNA electrophoresis of the dPCR of the Man26A knockout strains MA1179.1 and MA1115.1. **E.** Schematic overview and DNA electrophoresis of the dPCR of the cbhB knockout strain MA1117.1.

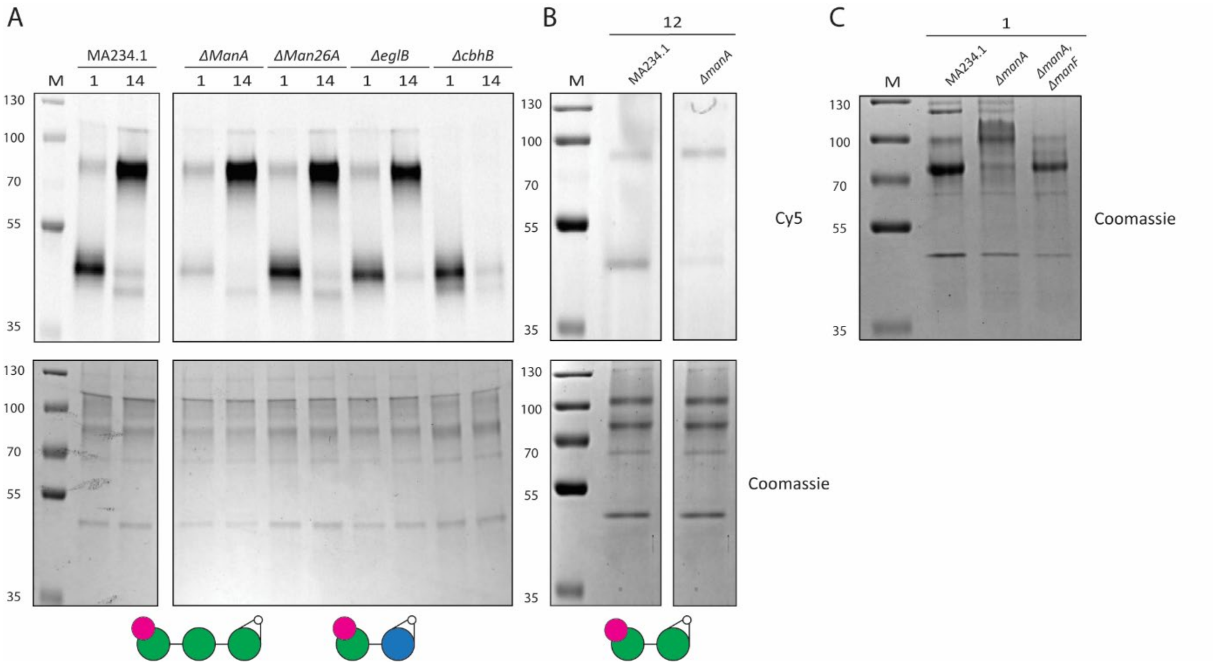

Supplemental figure 12. A. Cy5 fluorescence scanning (upper panels) and Coomassie staining (lower panels) of the knockout strains of *A. niger* grown for at least 72h in 1% kF. The parental strain MA234.1 was used as positive control. The secretomes were labelled with Cy5-Man-man-*manno*-cyclophellitol **1** and Cy5-Man-*manno*-cyclophellitol **14**. **B.** Cy5 fluorescence scanning (upper panels) and Coomassie staining (lower panels) of the *ΔmanA* knockout strain of *A. niger* grown for at least 72h in 1% kF. The parental strain MA234.1 was used as positive control. The secretomes were labelled with Cy5-Man-*manno*-cyclophellitol **12**. **C.** Coomassie staining of the *ΔmanA* knockout strain and the *ΔmanA; ΔmanF* double knockout strain of *A. niger* grown for at least 72h in 1% kF. The parental strain MA234.1 was used as positive control. The secretomes were labelled with Cy5-Man-man-*manno*-cyclophellitol **1**.

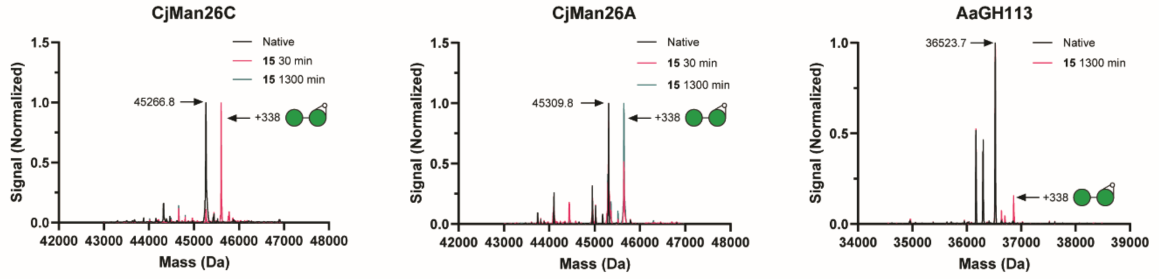

Supplemental figure 13. Deconvoluted intact MS spectra of recombinant *Cj*Man26C, *Cj*Man26A and GH113 of *Alicyclobacillus acidocaldarius* before and after treatment with Man-*manno*-cyclophellitol **15** for either 30 or 1300 min at 37 °C.

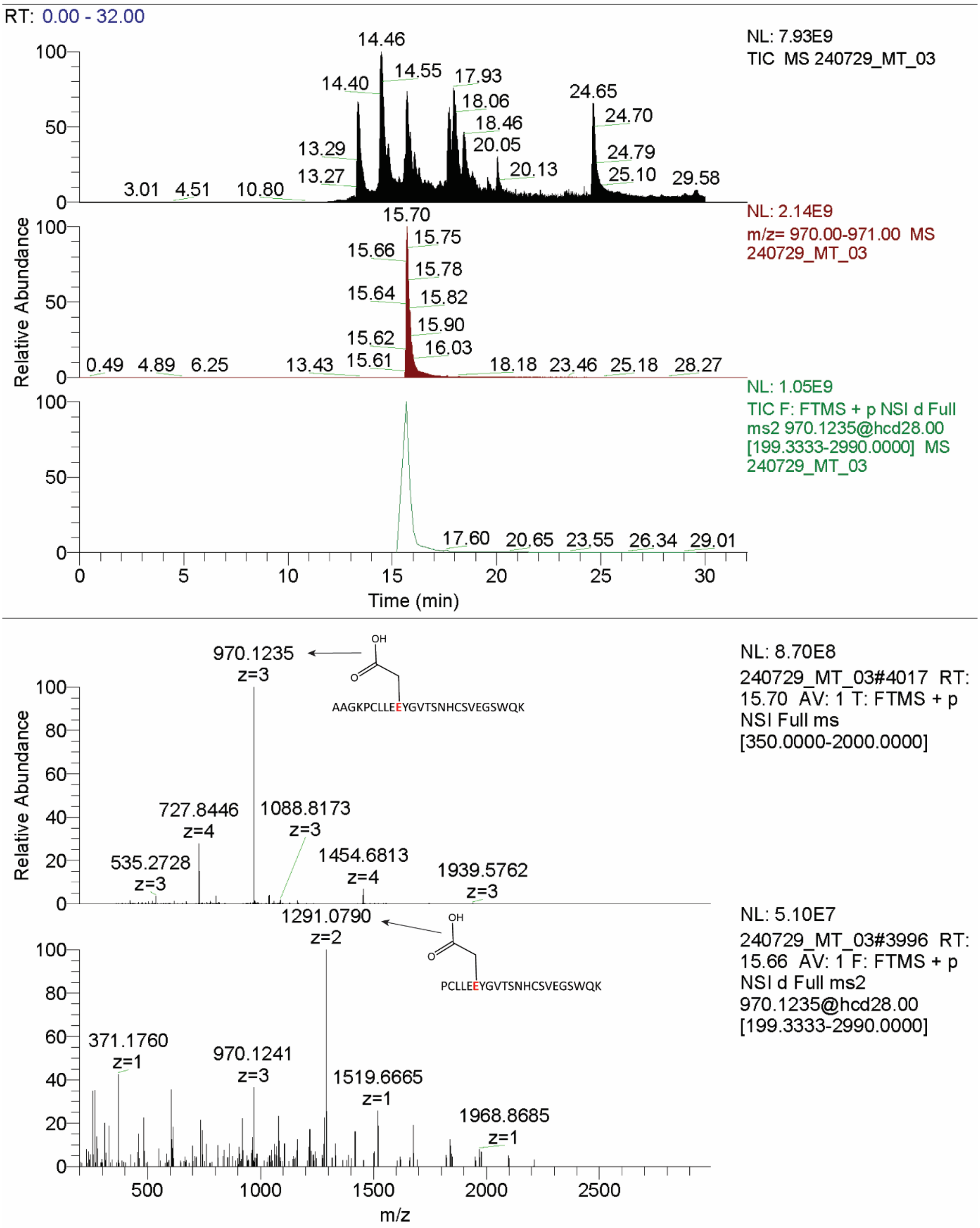

Supplemental figure 14. Chromatogram, MS1 and MS2 spectra of untreated recombinant *An*ManA. The structure of the target tryptic peptide and fragment has been reported on the figure and assigned to the respective m/z.

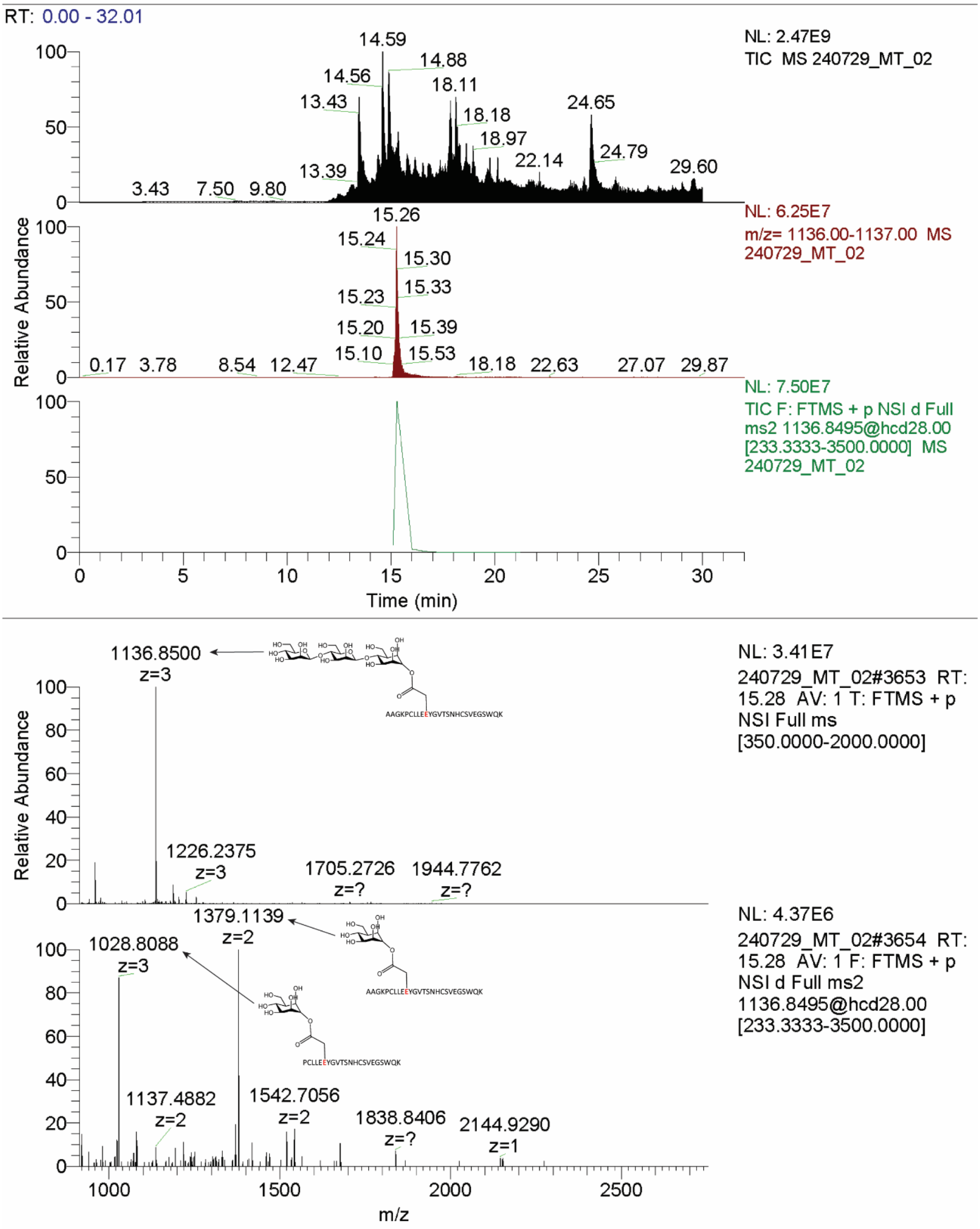

Supplemental figure 15. Chromatogram, MS1 and MS2 spectra of recombinant *An*ManA after labelling with Man-*manno*-cyclophellitol **15**. The structure of the target tryptic peptide and fragment has been reported on the figure and assigned to the respective m/z.

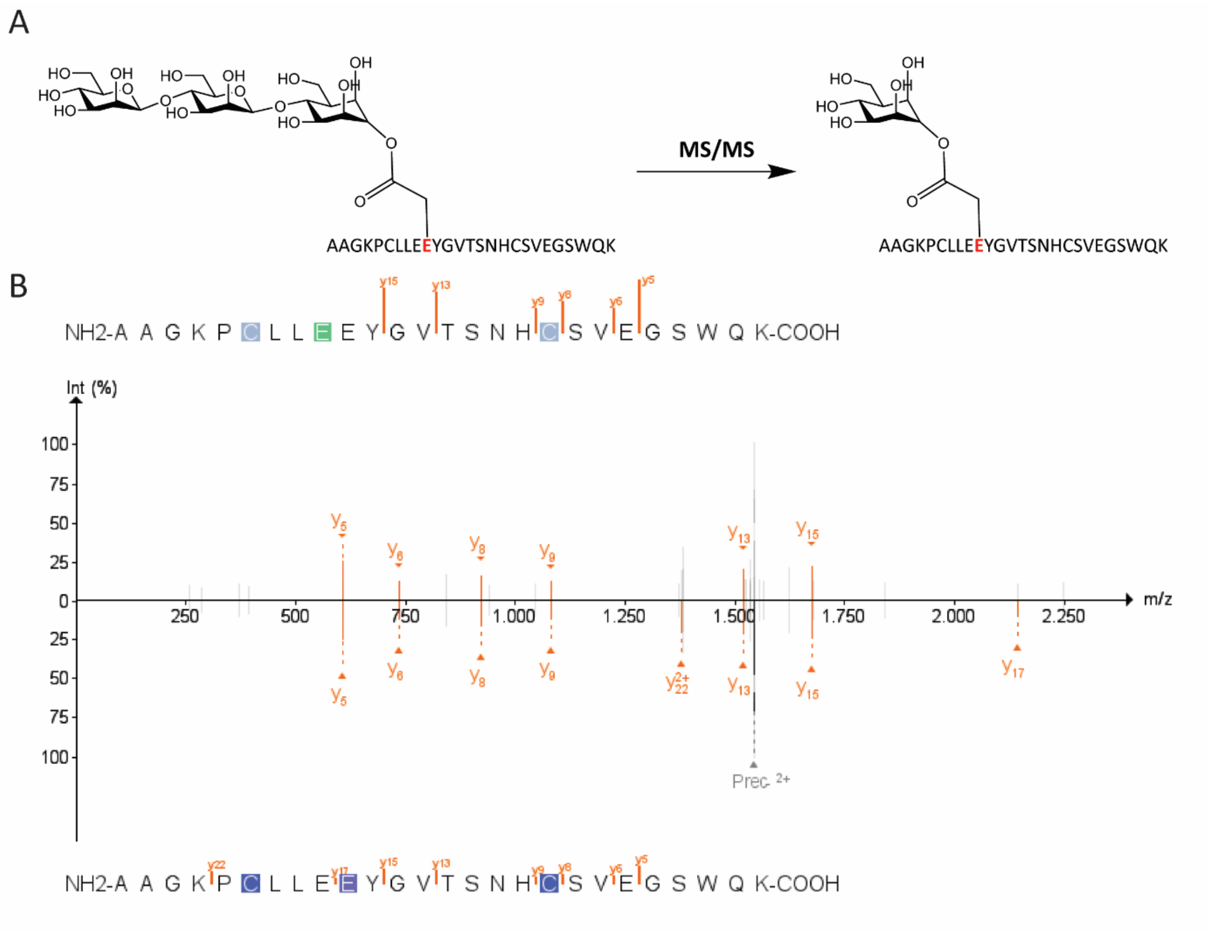

Supplemental figure 16. A. Schematic representation of the non-reducing-end sugar hydrolysis observed after MS/MS fragmentation. **B.** FragPipe labile search output of the active-site tryptic peptide of *An*ManA after labelling with Man-*manno*-cyclophellitol **16**. The upper part of the graph shows the fragment matching without accounting for the cleavage of the inhibitor. The lower part shows an increase in the number of assigned fragments after accounting for the mass difference.

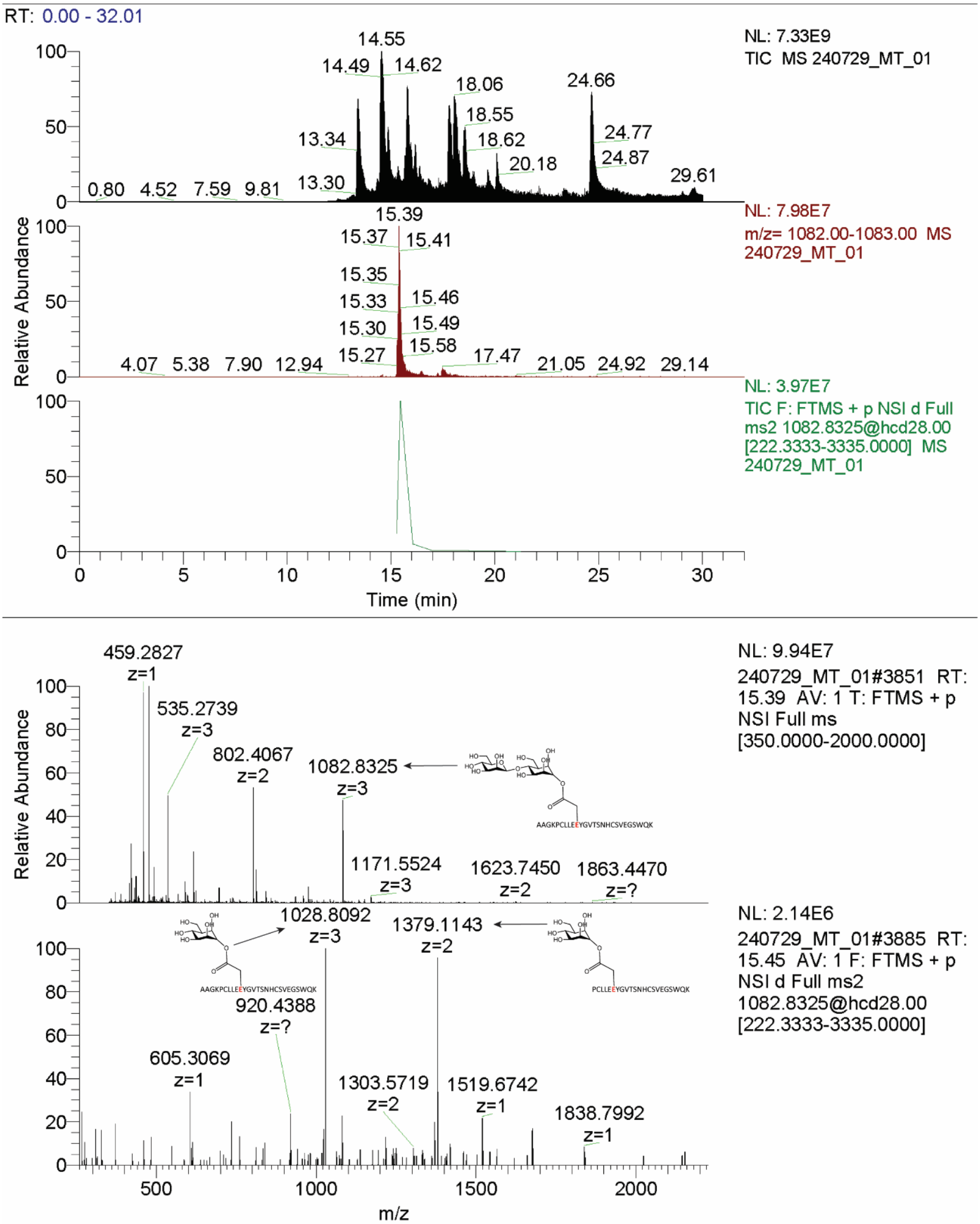

Supplemental figure 17. Chromatogram, MS1 and MS2 spectra of recombinant *An*ManA after labelling with Man-man-*manno*-cyclophellitol **16**. The structure of the target tryptic peptide and fragment has been reported on the figure and assigned to the respective m/z.

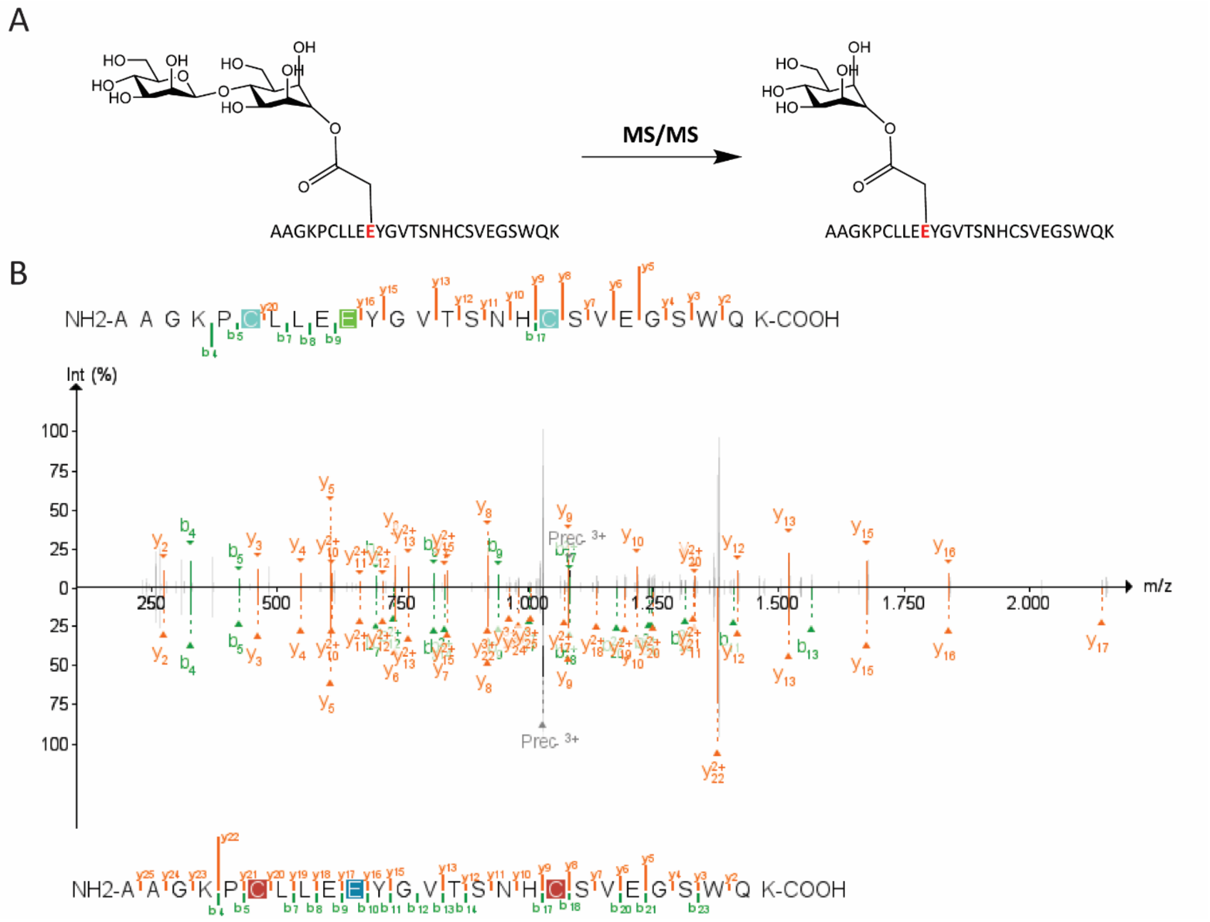

Supplemental figure 18. A. Schematic representation of the non-reducing-end sugar hydrolysis observed after MS/MS fragmentation. **B.** FragPipe labile search output of the active-site tryptic peptide of *An*ManA after labelling with Man-*manno*-cyclophellitol **15**. The upper part of the graph shows the fragment matching without accounting for the cleavage of the inhibitor. The lower part shows an increase in the number of assigned fragments after accounting for the mass difference.

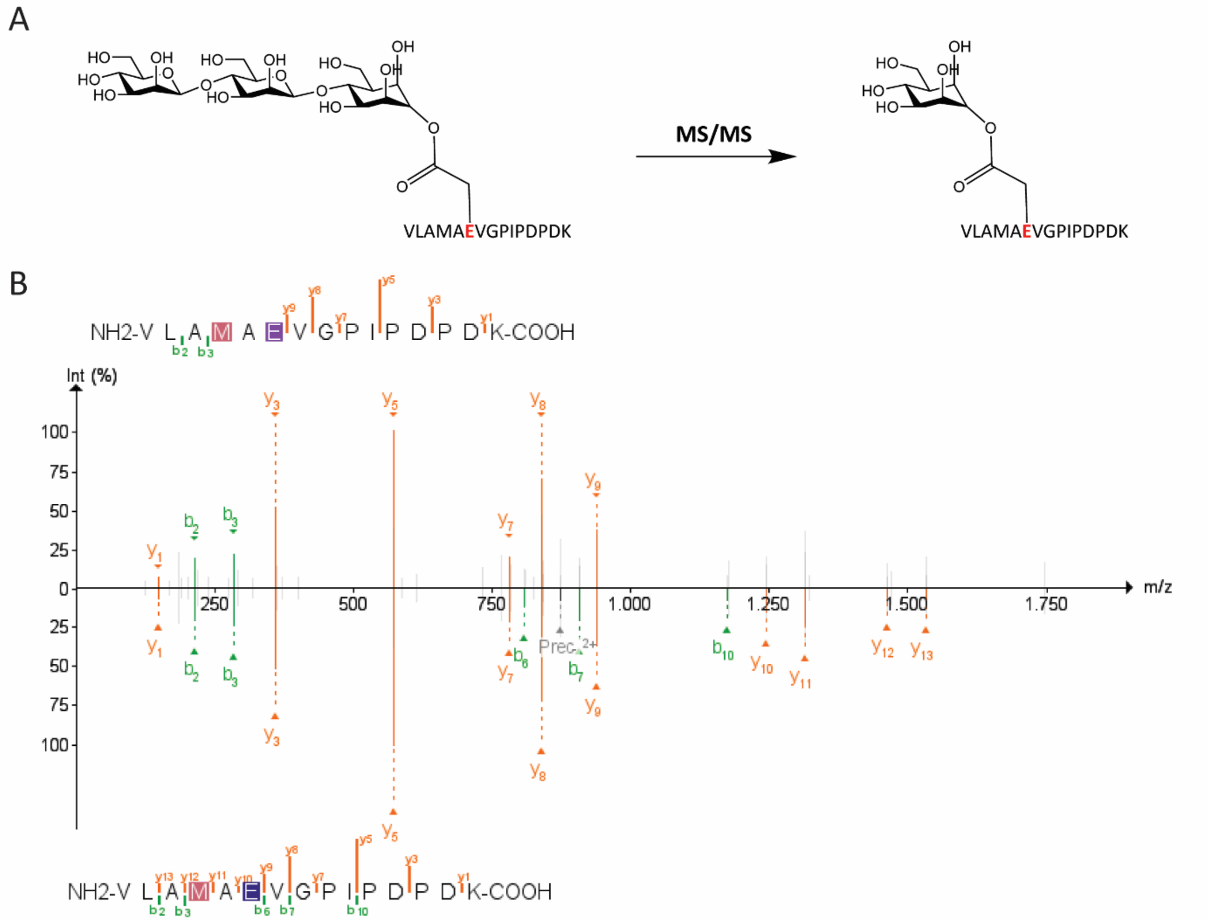

Supplemental figure 19. A. Schematic representation of the non-reducing-end sugar hydrolysis observed after MS/MS fragmentation. **B.** FragPipe labile search output of the active-site tryptic peptide of *An*Man26A after labelling with Man-*manno*-cyclophellitol **16**. The upper part of the graph shows the fragment matching without accounting for the cleavage of the inhibitor. The lower part shows an increase in the number of assigned fragments after accounting for the mass difference.

Supplemental Table 7. Data collection and refinement statistics (molecular replacement)

	ManA - **16** (PDBID: 31AO)
**Data collection**	
Space group	P 2_1 2_1 2_1
Cell dimensions	
a, *b*, *c* (Å)	67.04, 86.23, 73.32
α, β, γ (°)	90.00, 90.00, 90.00
Resolution (Å)	30.95-1.42 (1.44-1.42) *
$CC_{1/2}$	0.988 (0.209)
$I / \sigma I$	4.6 (0.8)
Completeness (%)	99.9 (100.0)
Redundancy	5.9 (6.0)
**Refinement**	
Resolution (Å)	30.95-1.42
No. reflections	64027
R_{work} / R_{free}	0.18 / 0.20
No. atoms	
Protein	2694
Ligand/ion	62/40
Water	308
B-factors	
Protein	12.2
Ligand/ion	23.7/59.7
Water	22.9
R.m.s. deviations	
Bond lengths (Å)	0.009
Bond angles (°)	1.7

*Values in parentheses are for highest-resolution shell.

Supplemental Table 8. Data collection and refinement statistics (molecular replacement).

	*Cj*Man26C-inhibitor complex (PDB 29QD)
**Data collection**	
Space group	$P6_122$
a, *b*, *c* (Å)	84.790, 84.790, 245.940
α, β, γ (°)	90.0, 90.0, 120.0
Resolution (Å)	73.43-1.20 (1.22-1.20)
R_{meas}	0.230 (5.811)
R_{pim}	0.037 (0.946)
$I / \sigma I$	11.6 (2.2)
Completeness (%)	100 (100)
Redundancy	36 (6.1)
$CC_{1/2}$	0.999 (0.330)
**Refinement**	
Unique reflections	180225 (8769)
R_{work} / R_{free}	0.130/0.149
No. atoms	
Protein	6051
Ligand/ion	49
Water	413
B-factors	
Protein	10.8
Ligand/ion	14.5
Water	27.1
R.m.s. deviations	
Bond lengths (Å)	0.0116
Bond angles (°)	1.91
Ramachandran plot	
Favoured (%)	98
Allowed (%)	2

*Values in parentheses are for highest-resolution shell.

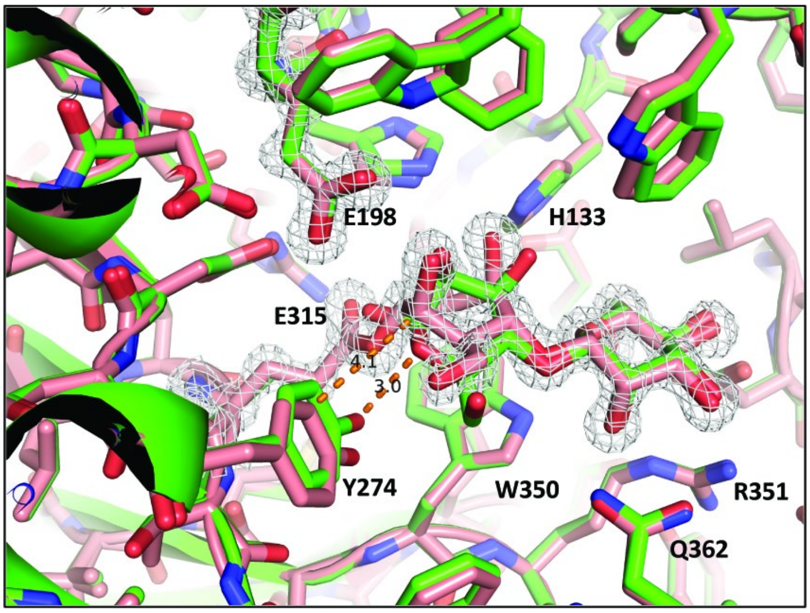

Supplemental figure 20. Overlay of the covalent complex between **15** and *Cj*Man26C shown in salmon (this work) and the previously deposited co-crystal structure of *Cj*Man26C E315A mutant with mannobiose, superposed in green (PDB ID: 2VX7). The experimentally determined electron density (contoured to 2σ) for **15** and the catalytic amino acid side chains is shown as a white mesh. The measured distances between Y274 and the ring oxygen of mannobiose or the C7 of **15** are shown as orange dashes. The names of the residues forming polar interactions with **15** are reported in the figure.

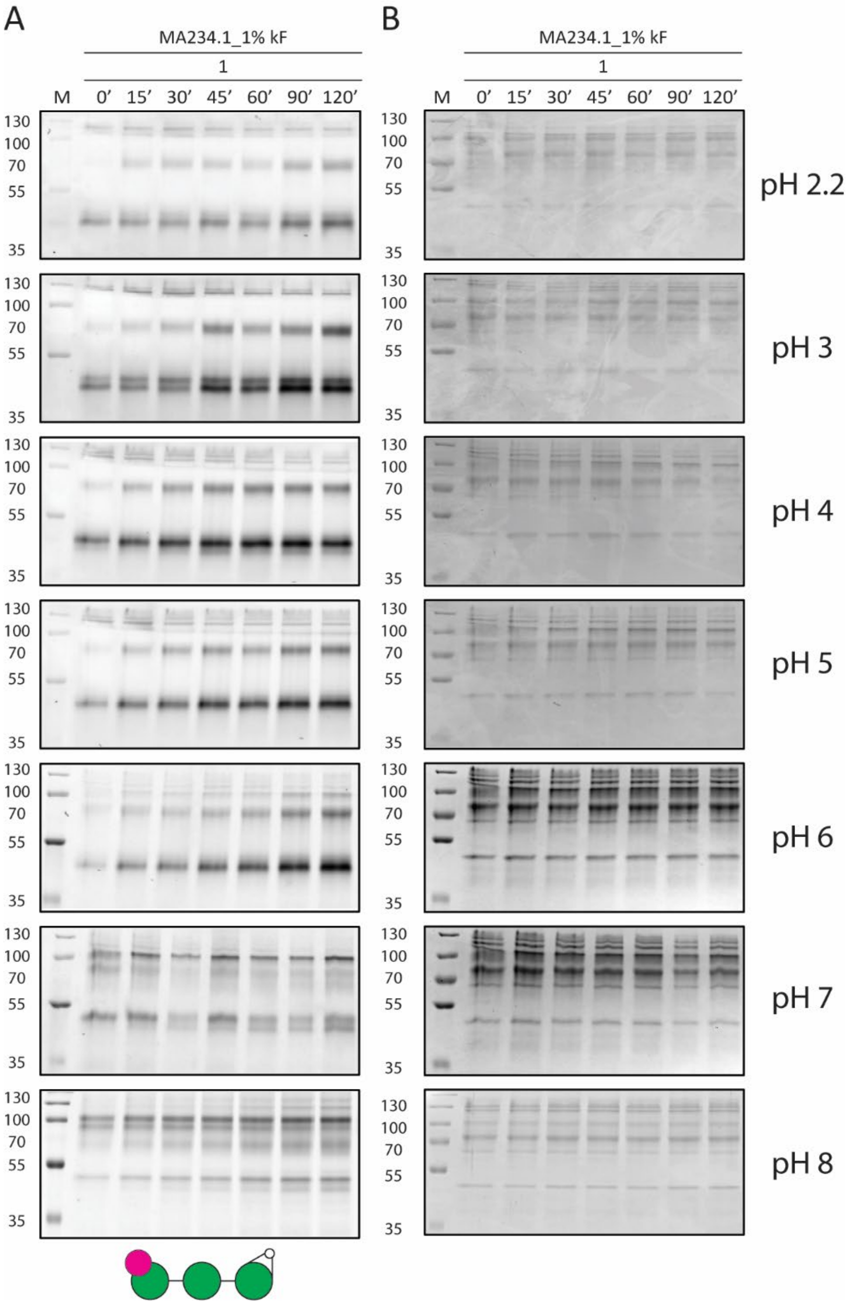

Supplemental figure 21. A. Cy5 fluorescence of ABP analysis of pH optimum. The MA234.1 secretome of *A. niger* grown on kF was exchanged to McIlvaine buffer at different pHs and then labelled with Cy5-Man-man-*manno*-cyclophellitol **1** for up to 2 hours 37 °C. After the labelling the proteins were separated by SDS-page. **B.** Coomassie stain of ABP analysis shown in A.

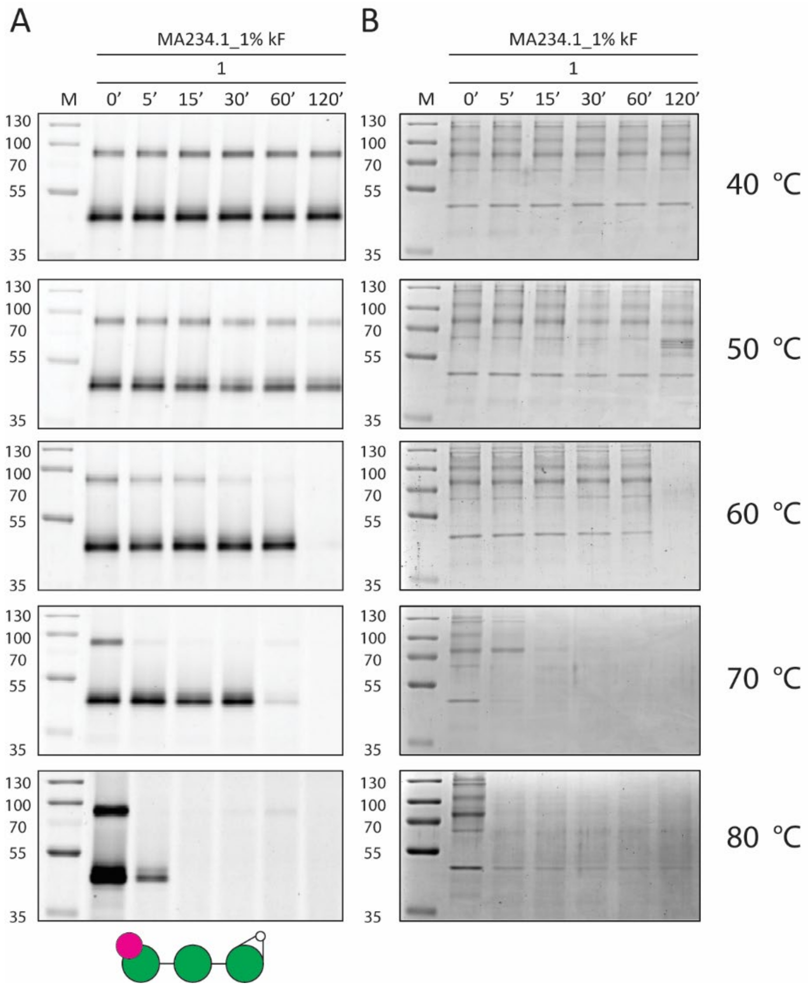

Supplemental figure 22. A. Fluorescent signal of SDS-page gel of the secretome of the MA234.1 strain of *A. niger* grown of kF labelled with Cy5-Man-man-manno-cyclophellitol **1**. The secretome was preincubated at different temperatures for up to 2 hours before labelling. **B.** Coomassie staining of the gel shown in panel A.

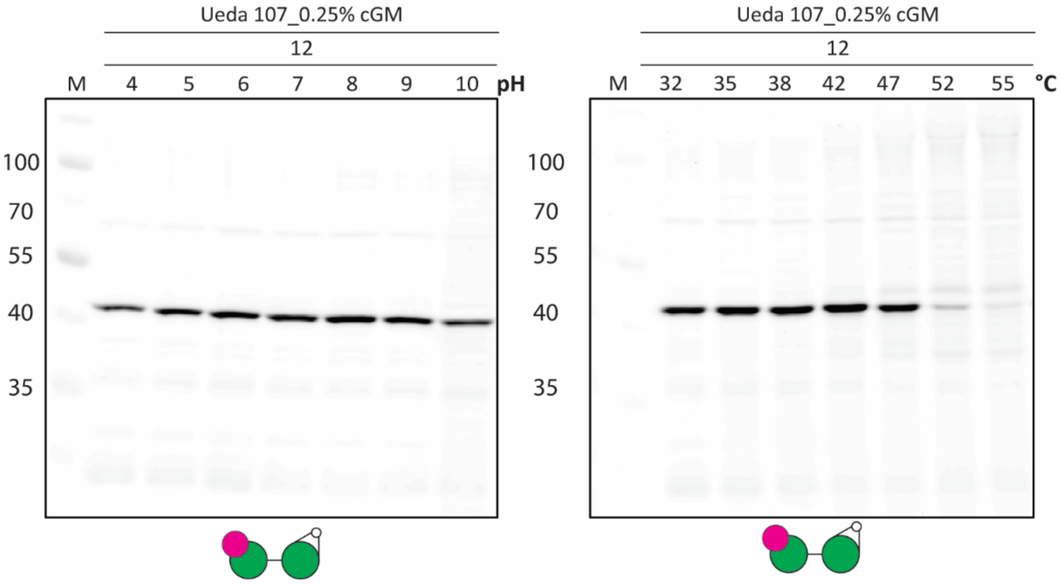

Supplemental figure 23. Cy5 fluorescence of the ABP analysis of pH optimum and temperature stability of the lysate of *C. japonicus* Ueda 107 grown on carob galactomannan (cGM). The protein in the lysate were labelled with Cy5-mannobiose **12**.

NMR Sperctra

6-*O*-benzoyl-2,3-*O*-benzyl-manno-cyclohexene (2)

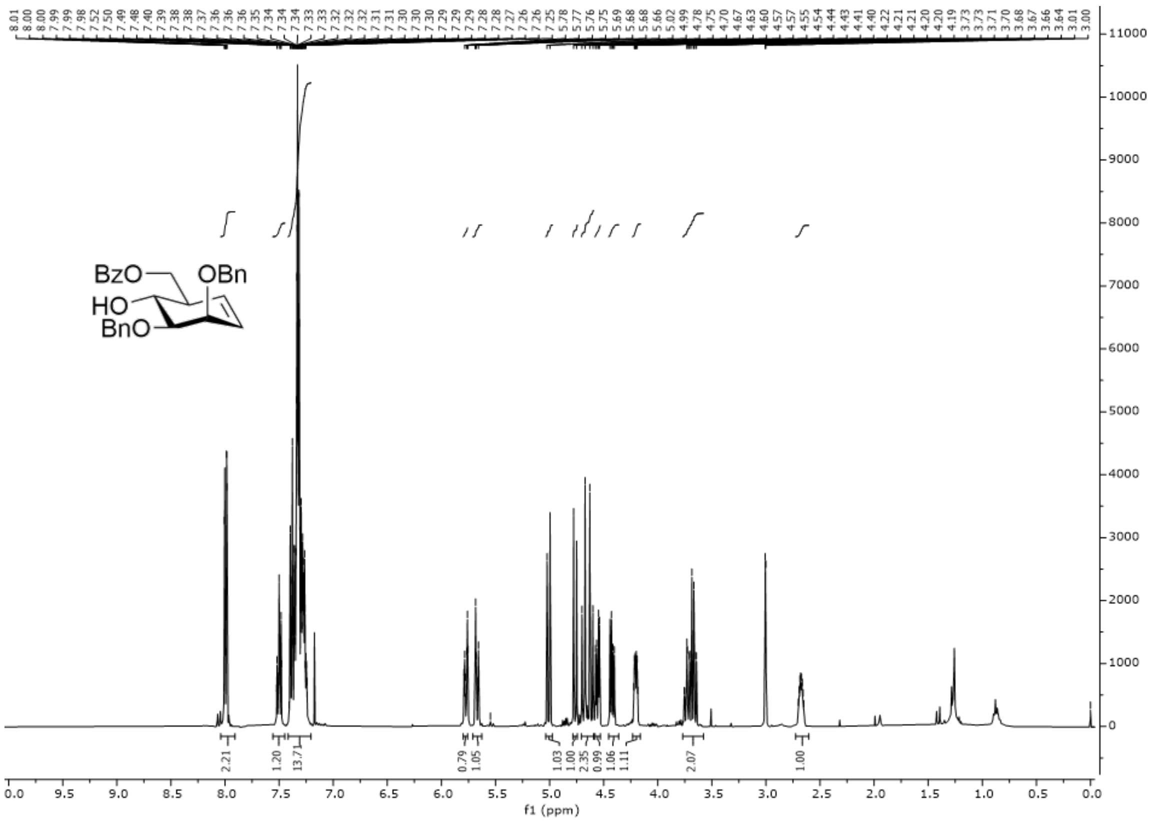

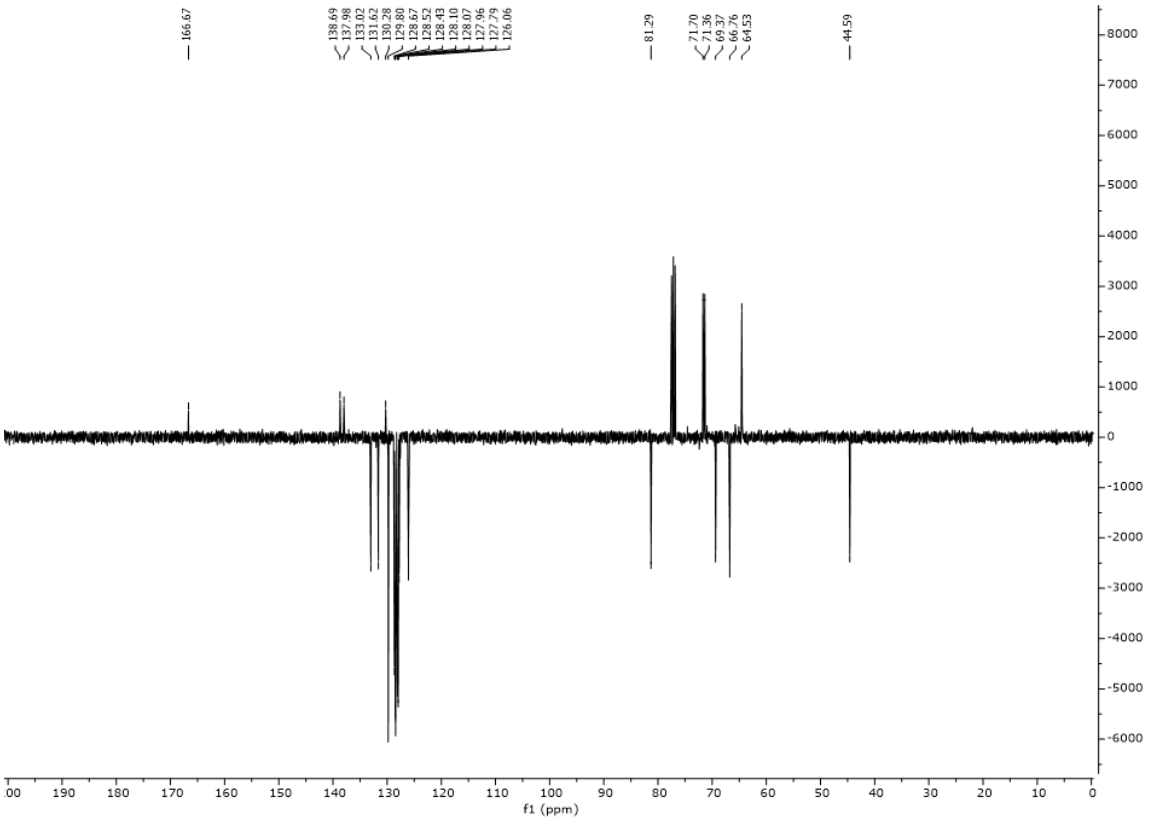

2,3-di-*O*-benzyl-4,6-*O*-benzylidene-(1-4)-β-D-mannopyranosyl-6-*O*-benzoyl-2,3-*O*-benzyl-manno-cyclohexene (4)

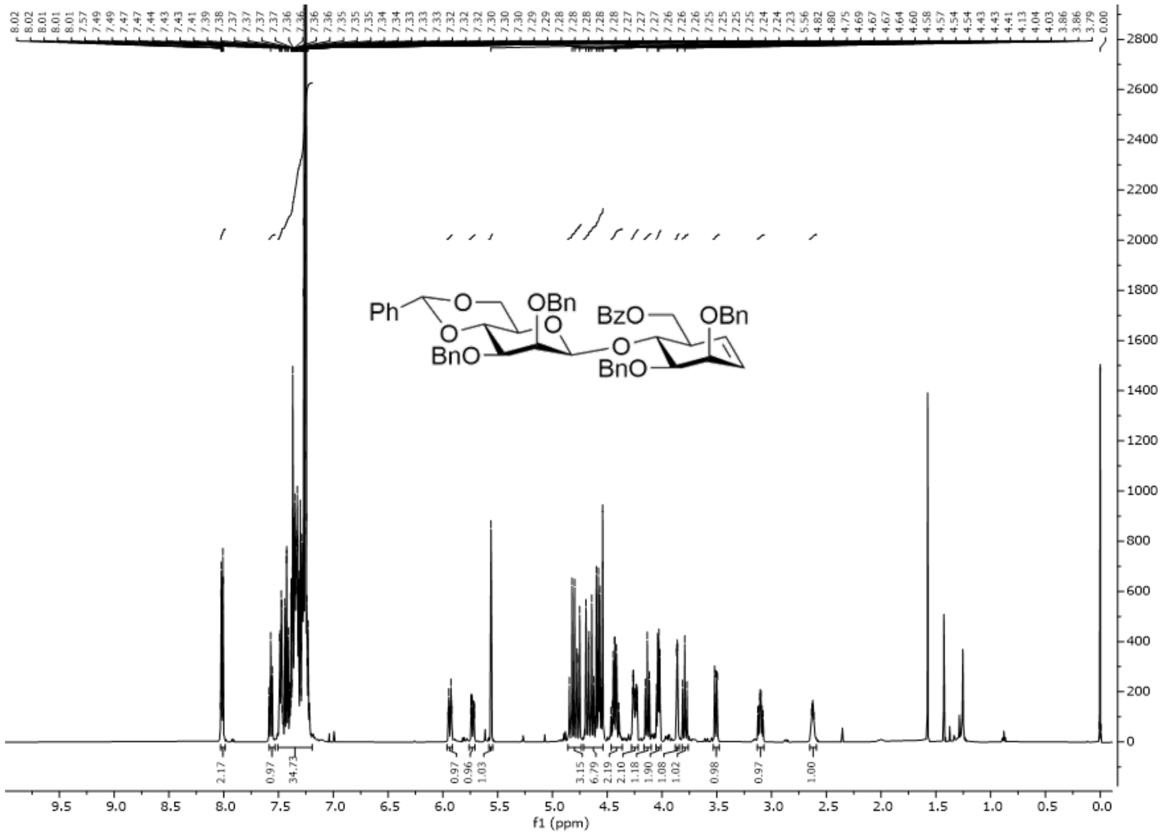

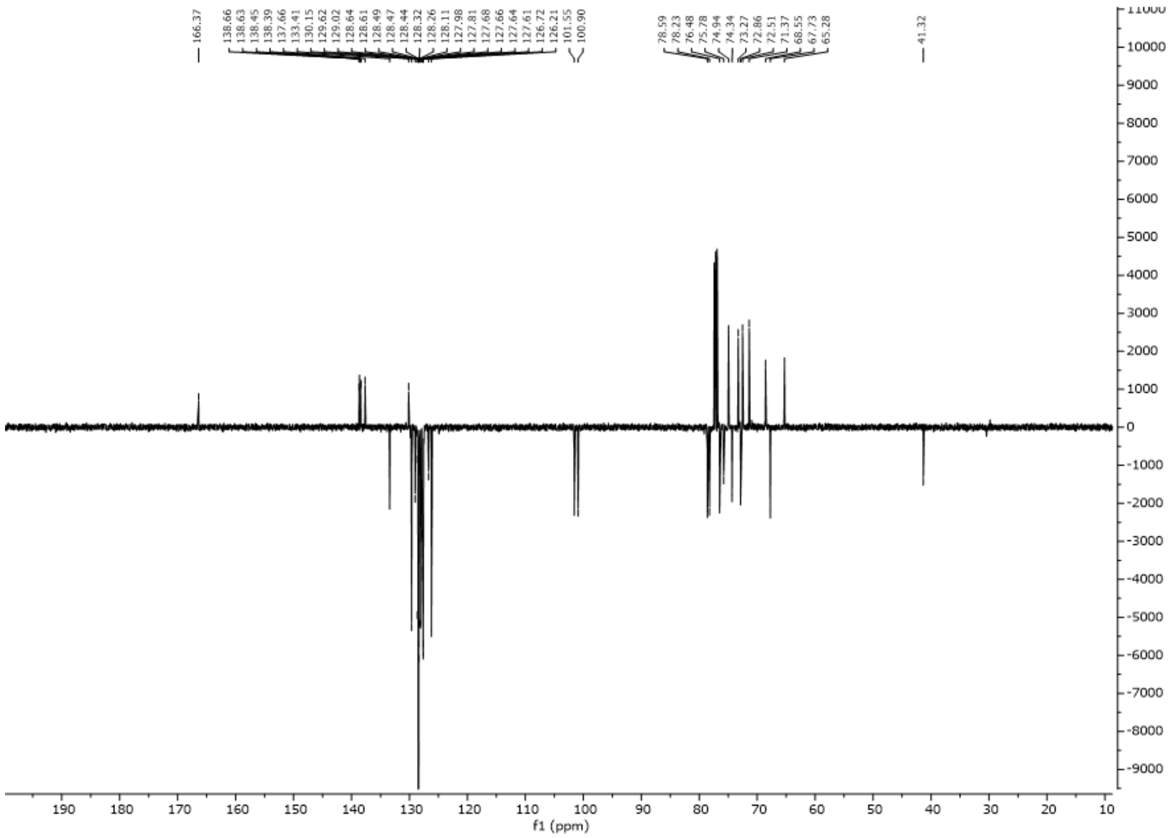

2,3-di-*O*-benzyl-4,6-*O*-benzylidene-(1-4)-β-D-mannopyranosyl-2,3-*O*-benzyl-manno-cyclohexene (18)

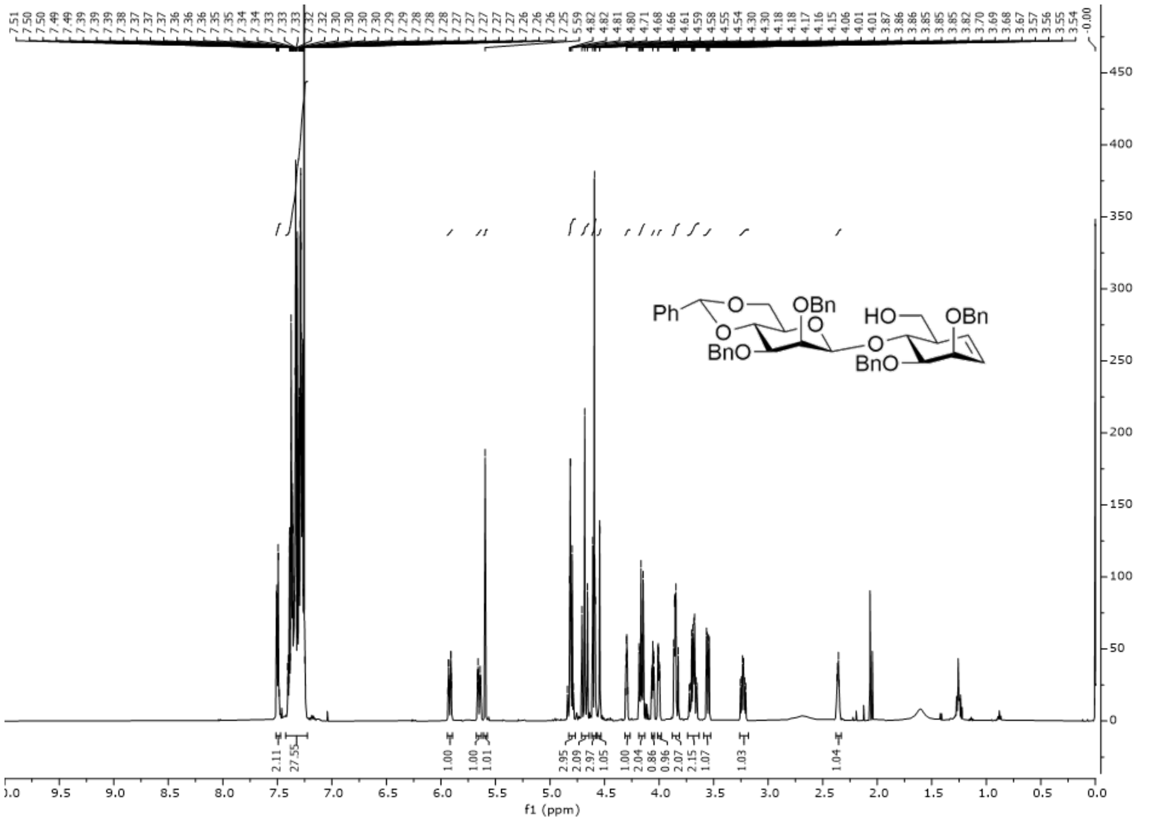

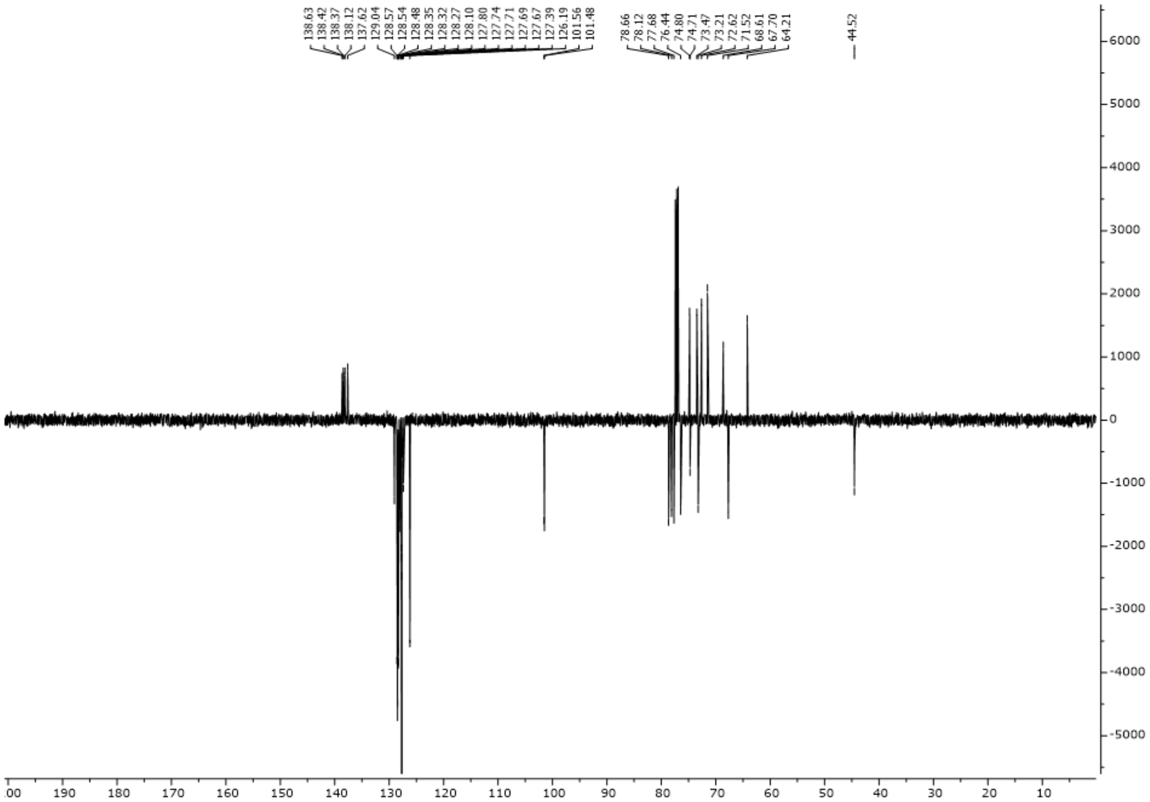

2,3-di-*O*-benzyl-4,6-*O*-benzylidene-(1-4)-β-D-mannopyranosyl-2,3-*O*-benzyl-manno-cyclophellitol (19)

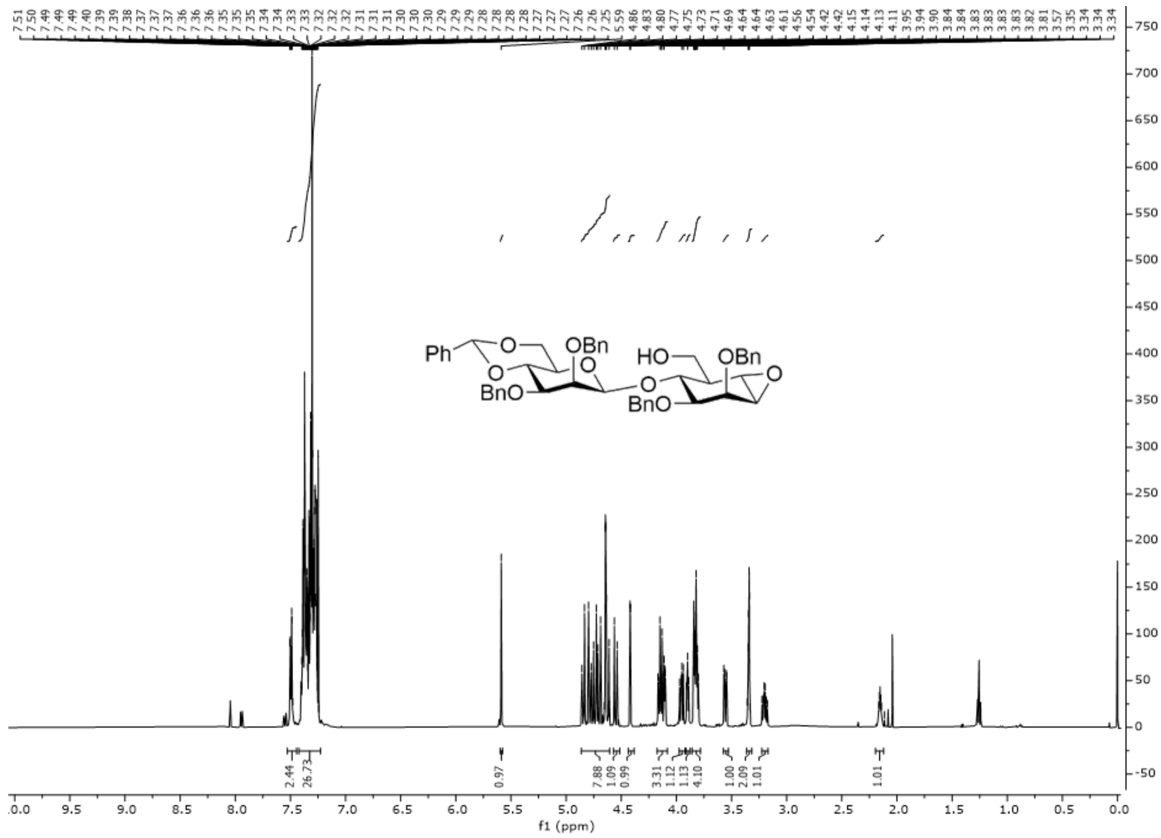

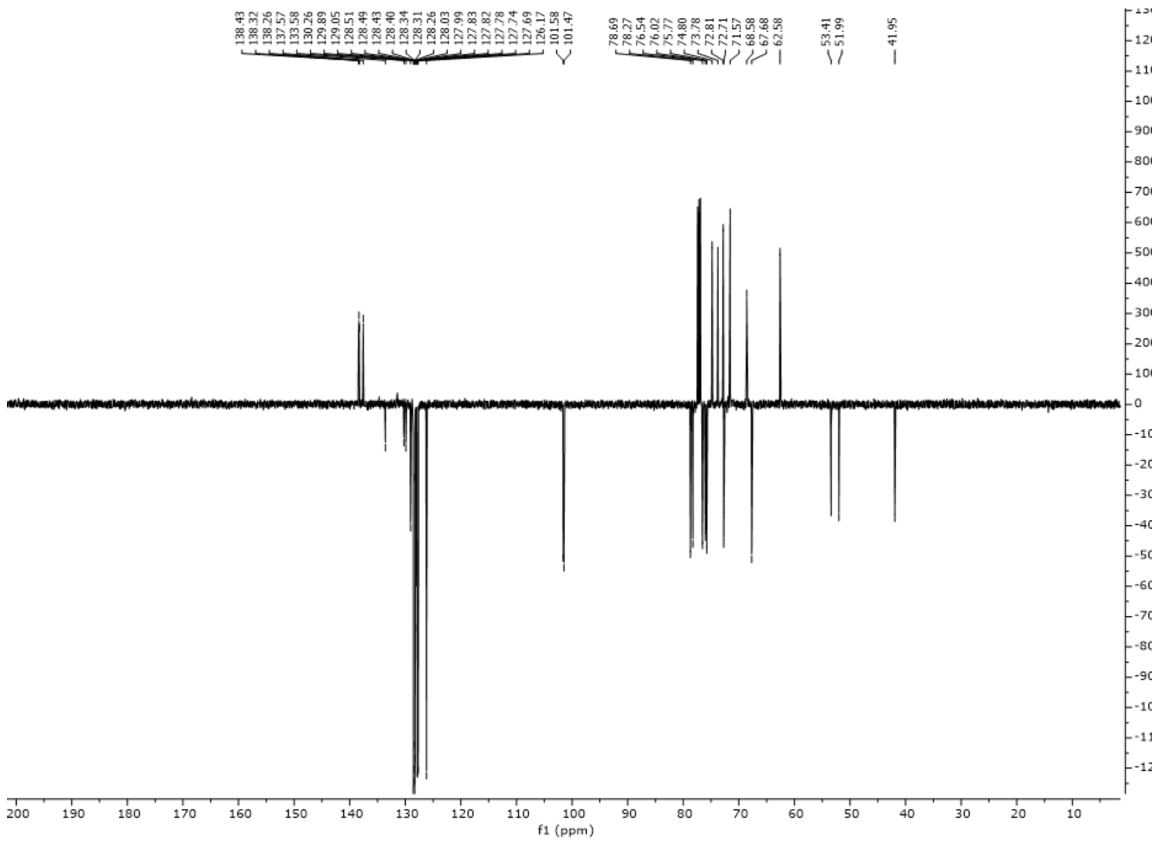

β-D-Mannose-(1-4)-manno-cyclophellitol (15)

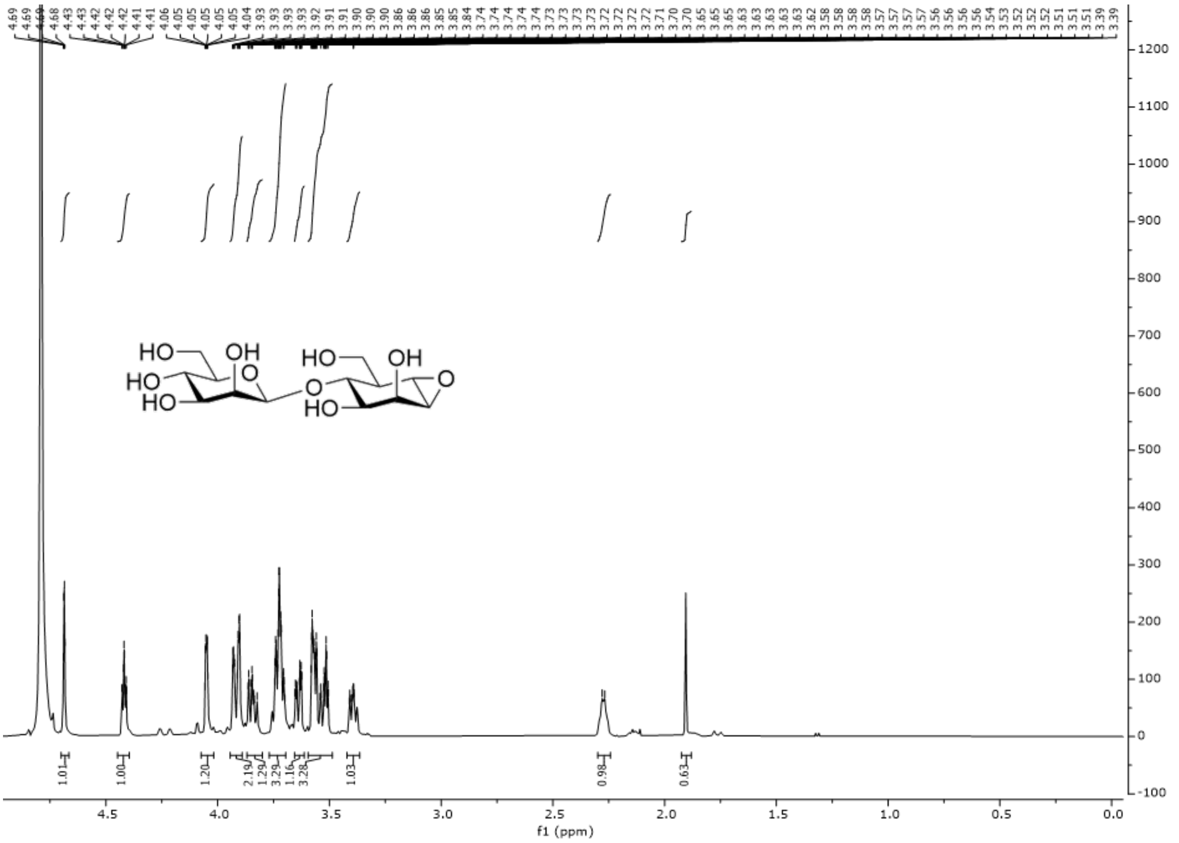

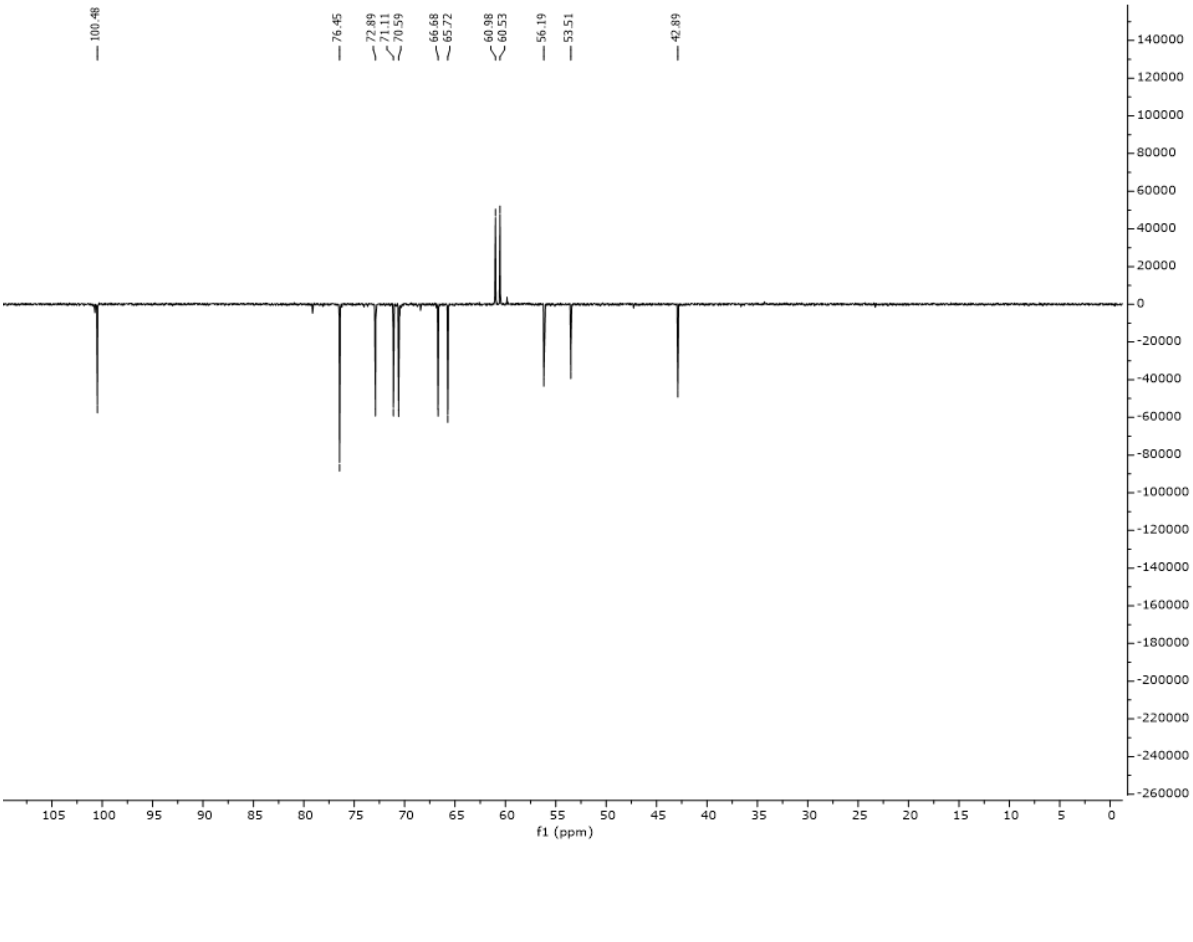

2,3-di-*O*-benzyl-4,6-*O*-benzylidene-(1-4)-β-D-mannopyranosyl-6-*O*-naphthyl-2,3-*O*-benzyl-manno-cyclohexene (5)

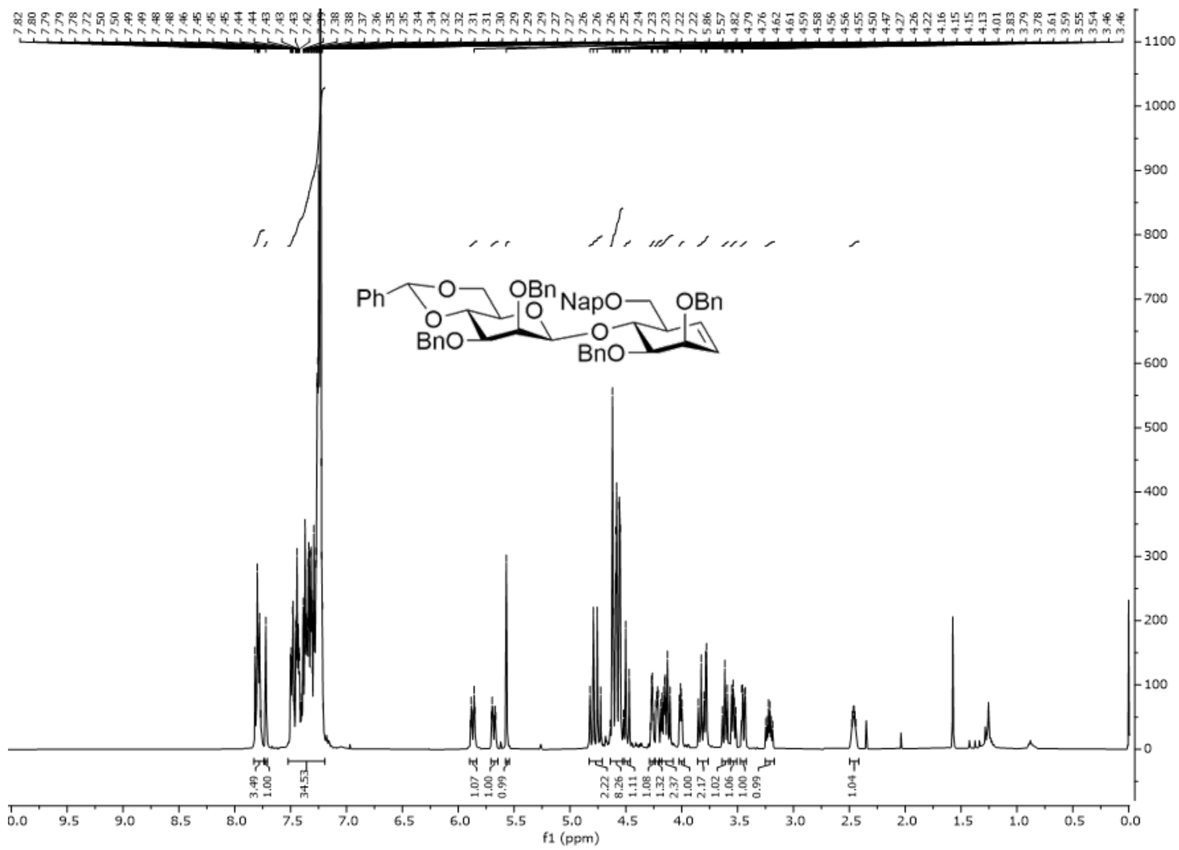

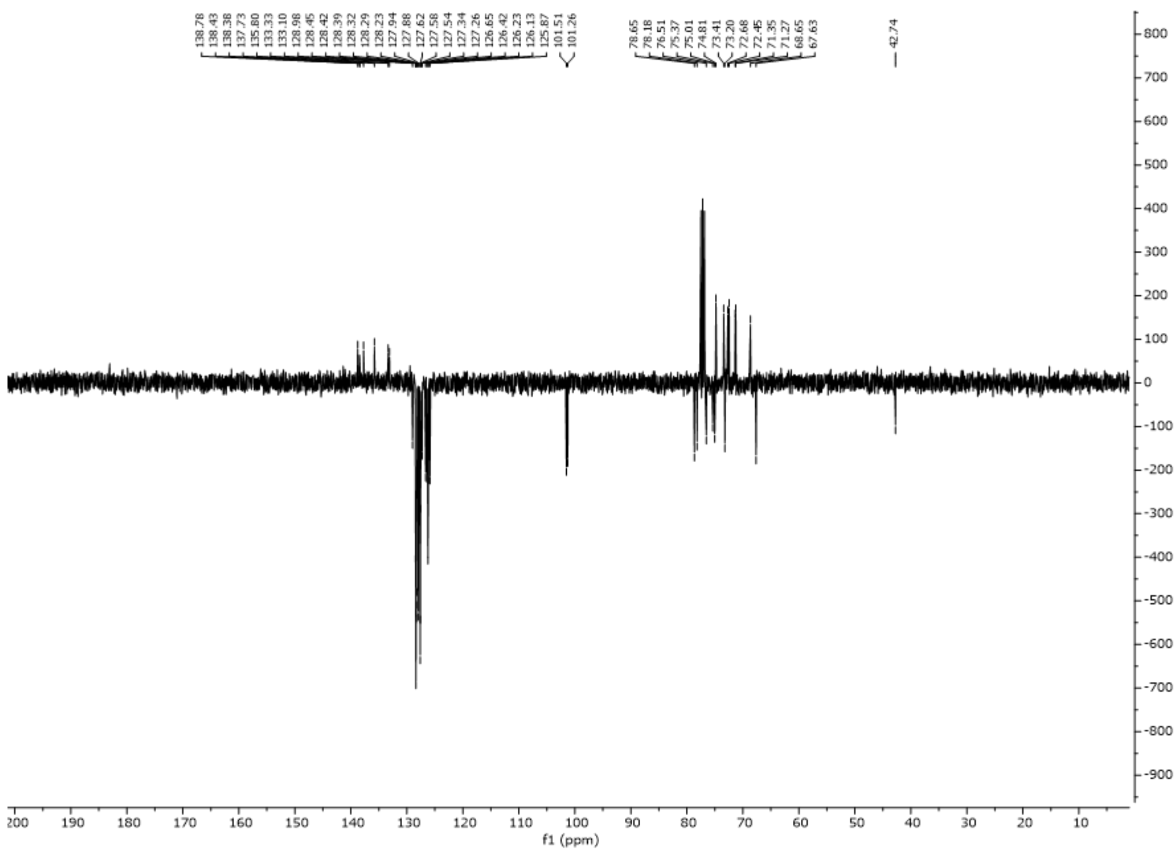

2,3,6-di-*O*-benzyl-(1-4)-β-D-mannopyranosyl-6-*O*-naphthyl-2,3-*O*-benzyl-manno-cyclohexene (6)

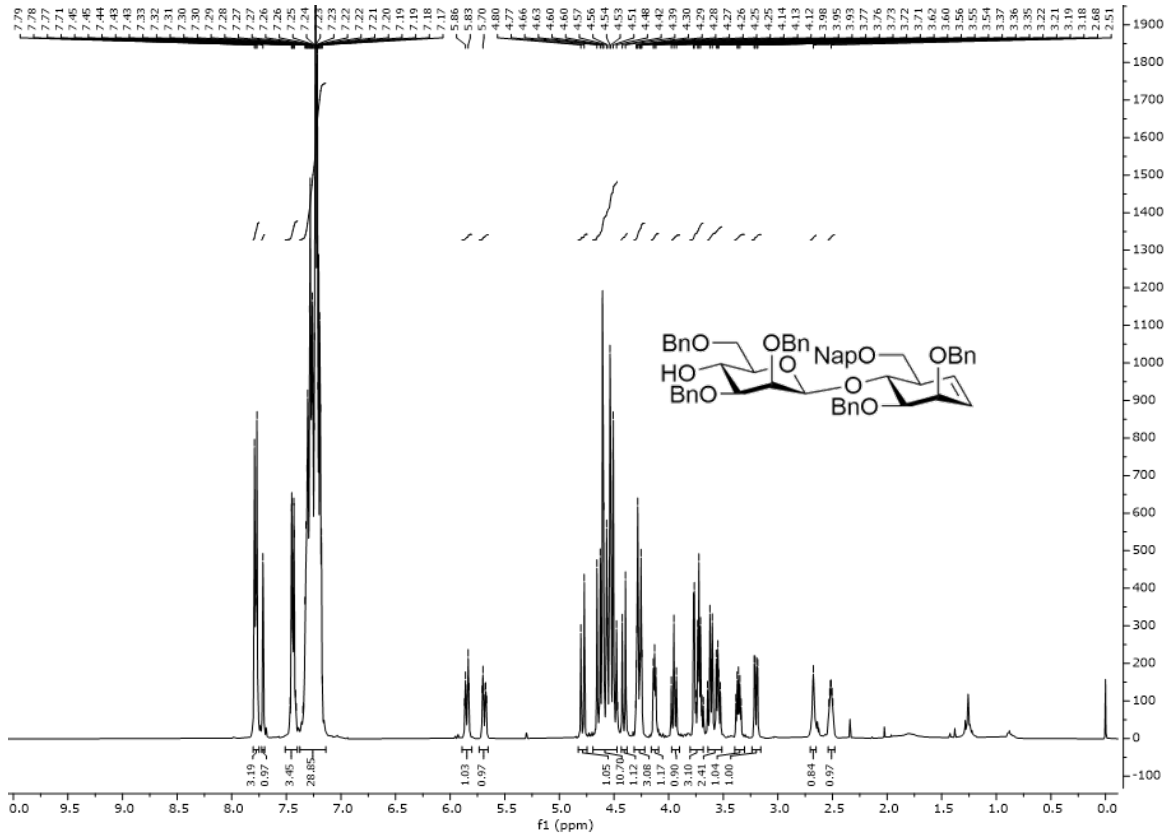

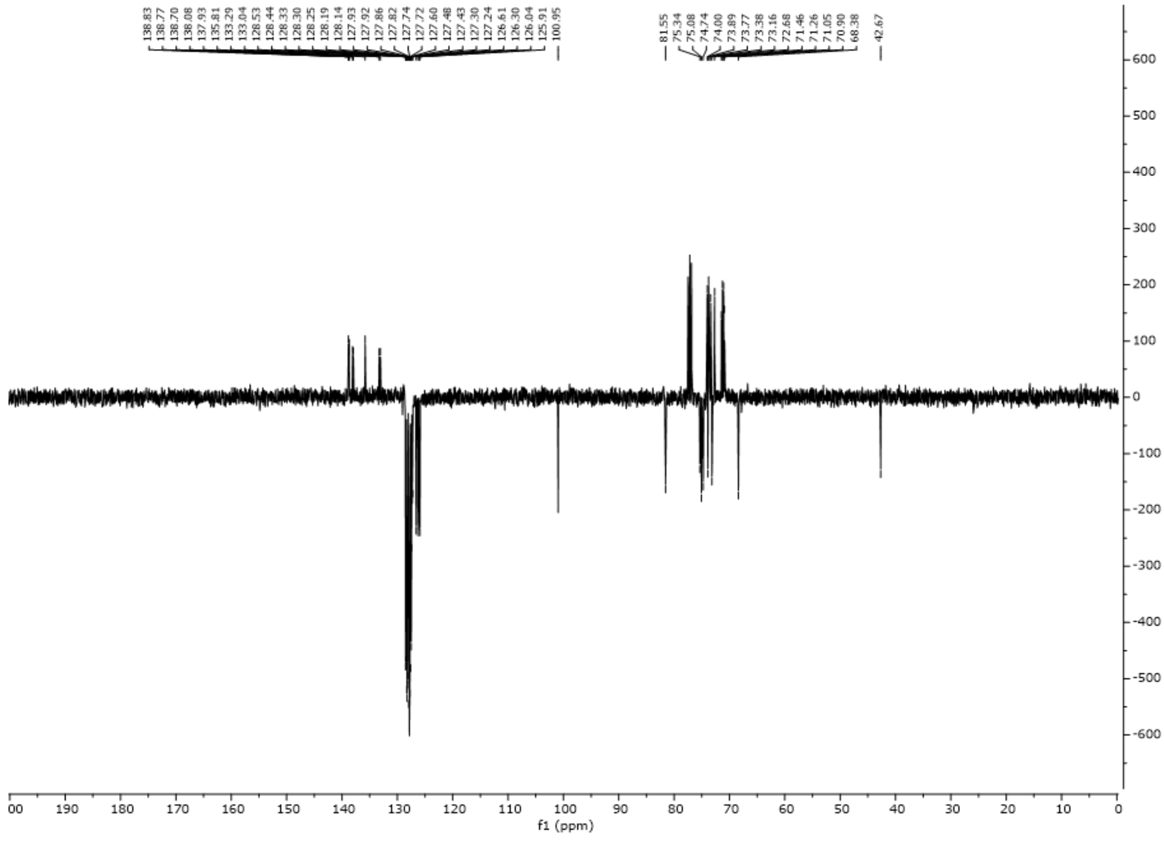

4-*O*-(8-azidooctyl)-2,3,6-di-*O*-benzyl-(1-4)-β-D-mannopyranosyl-6-*O*-naphthyl-2,3-*O*-benzyl-manno-cyclohexene (20)

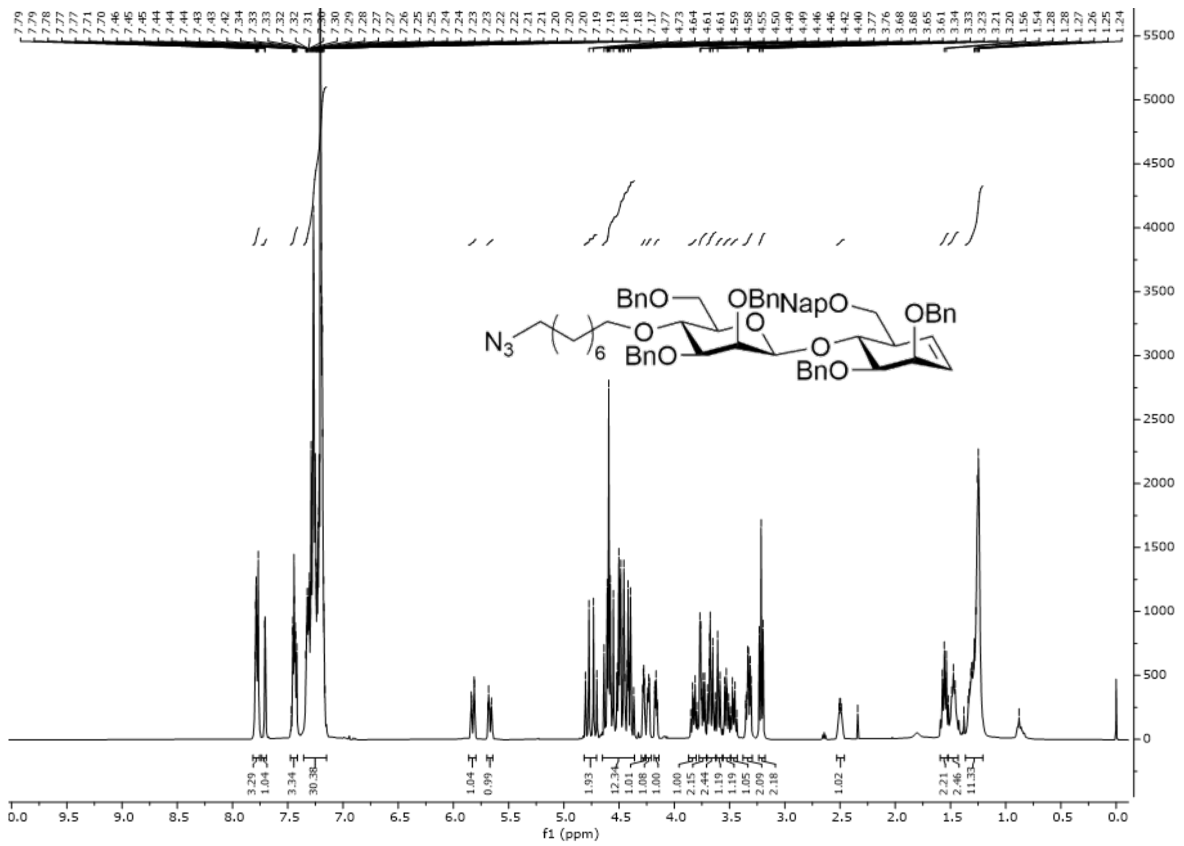

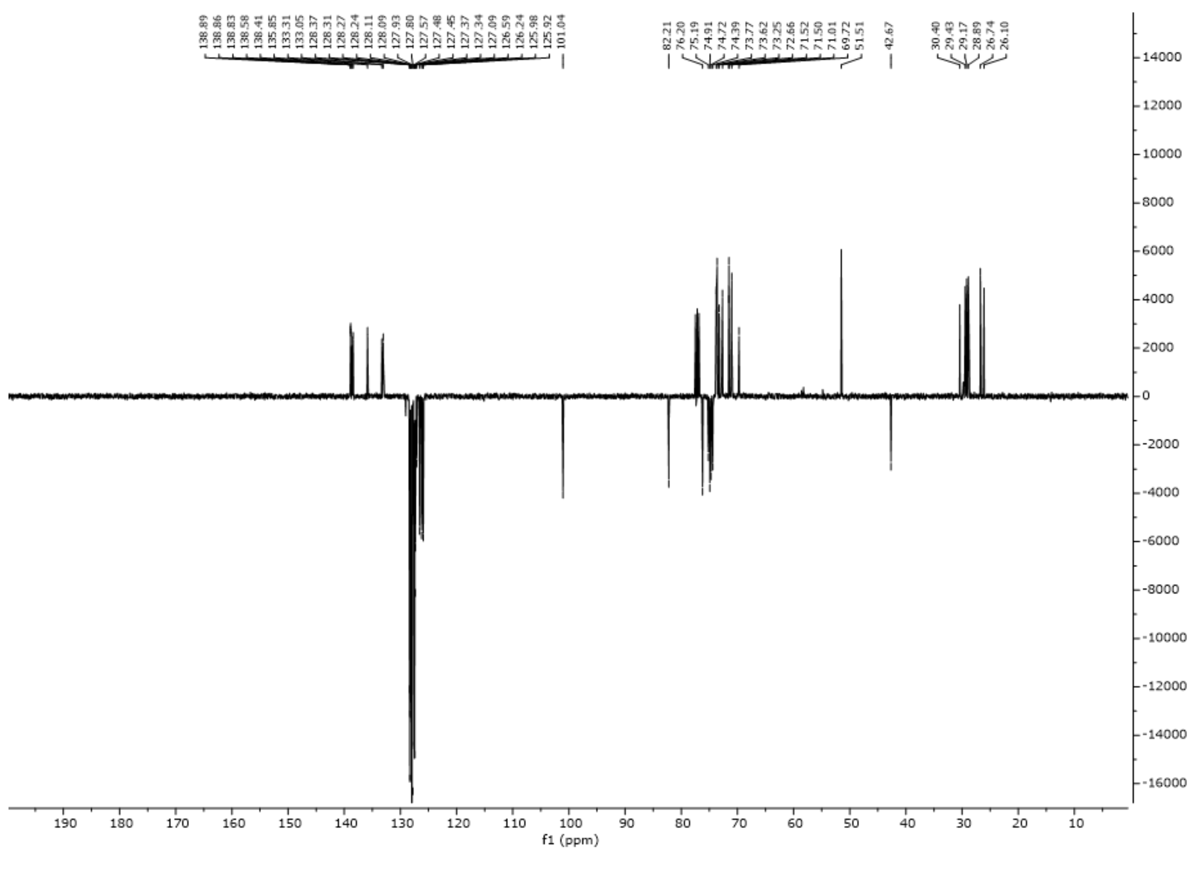

4-*O*-(8-azidooctyl)-2,3,6-di-*O*-benzyl-(1-4)-β-D-mannopyranosyl-2,3-*O*-benzyl-manno-cyclohexene (21)

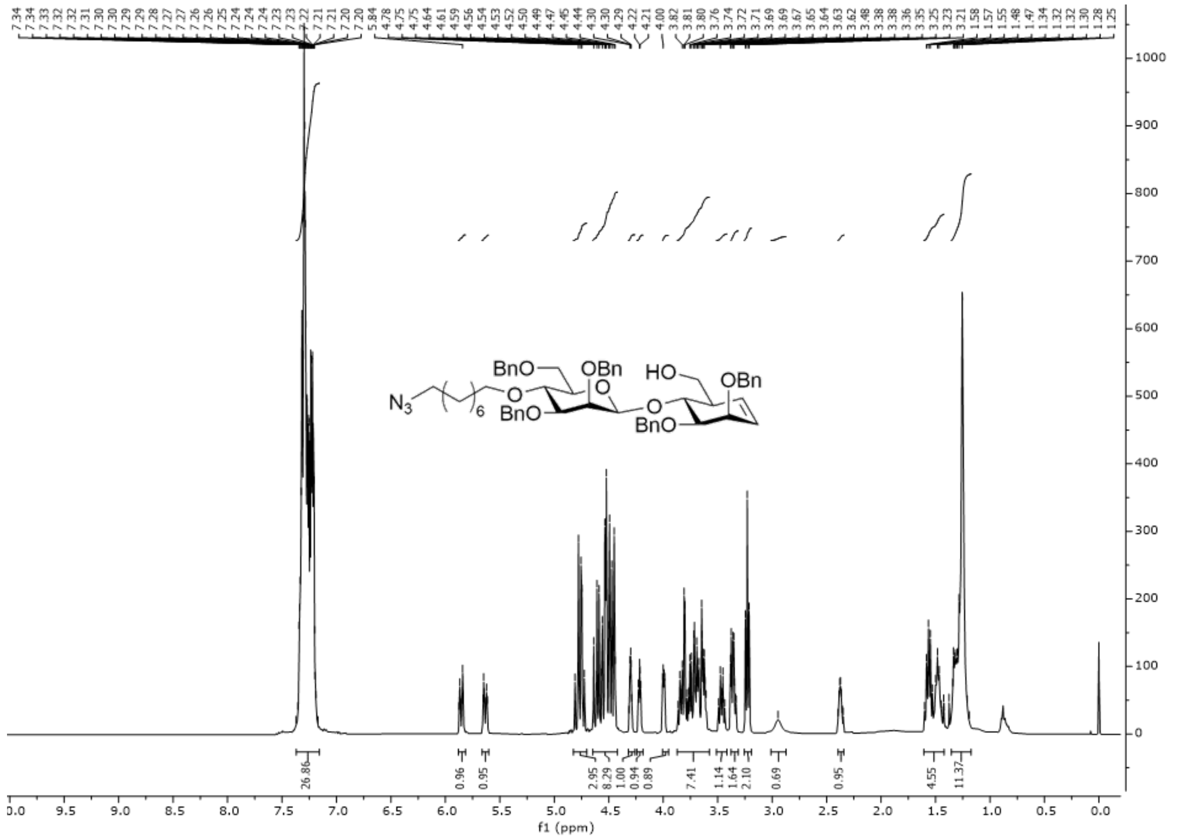

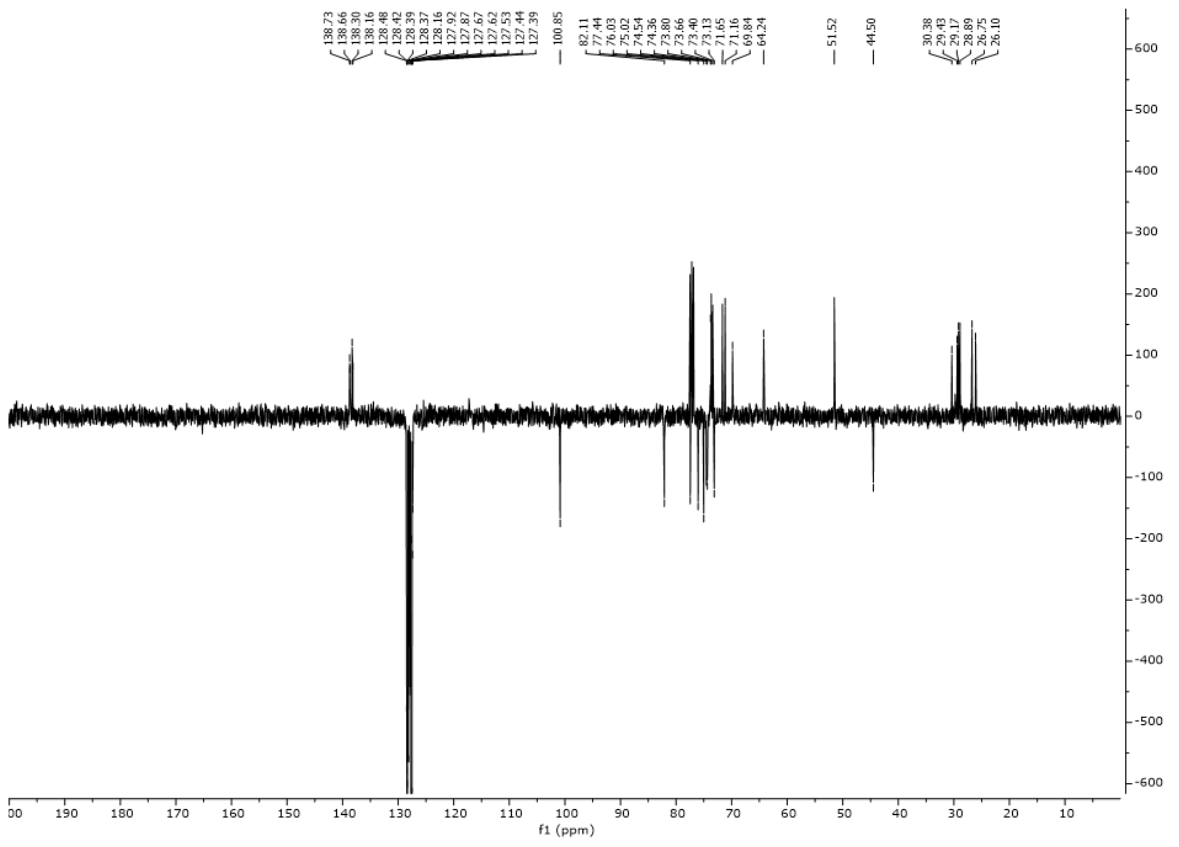

4-*O*-(8-azidooctyl)-2,3,6-di-*O*-benzyl-(1-4)-β-D-mannopyranosyl-2,3-*O*-benzyl-manno-cyclophellitol (22)

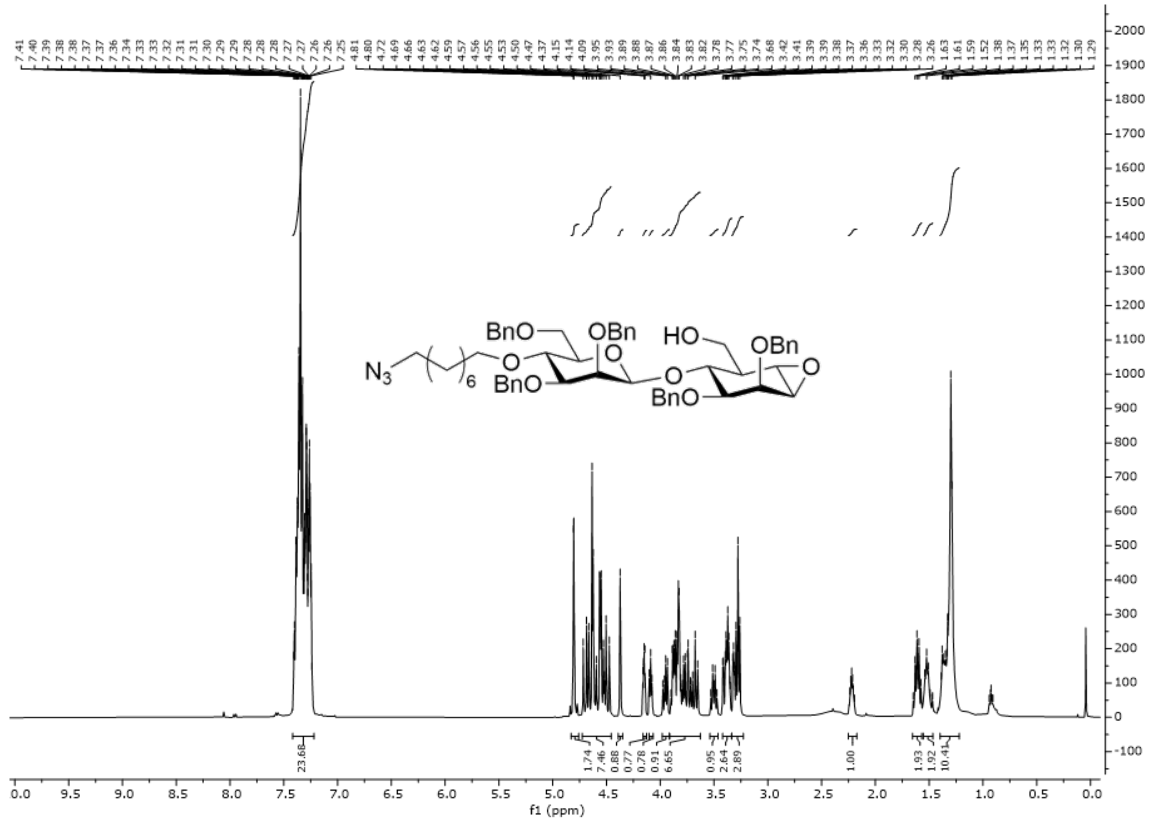

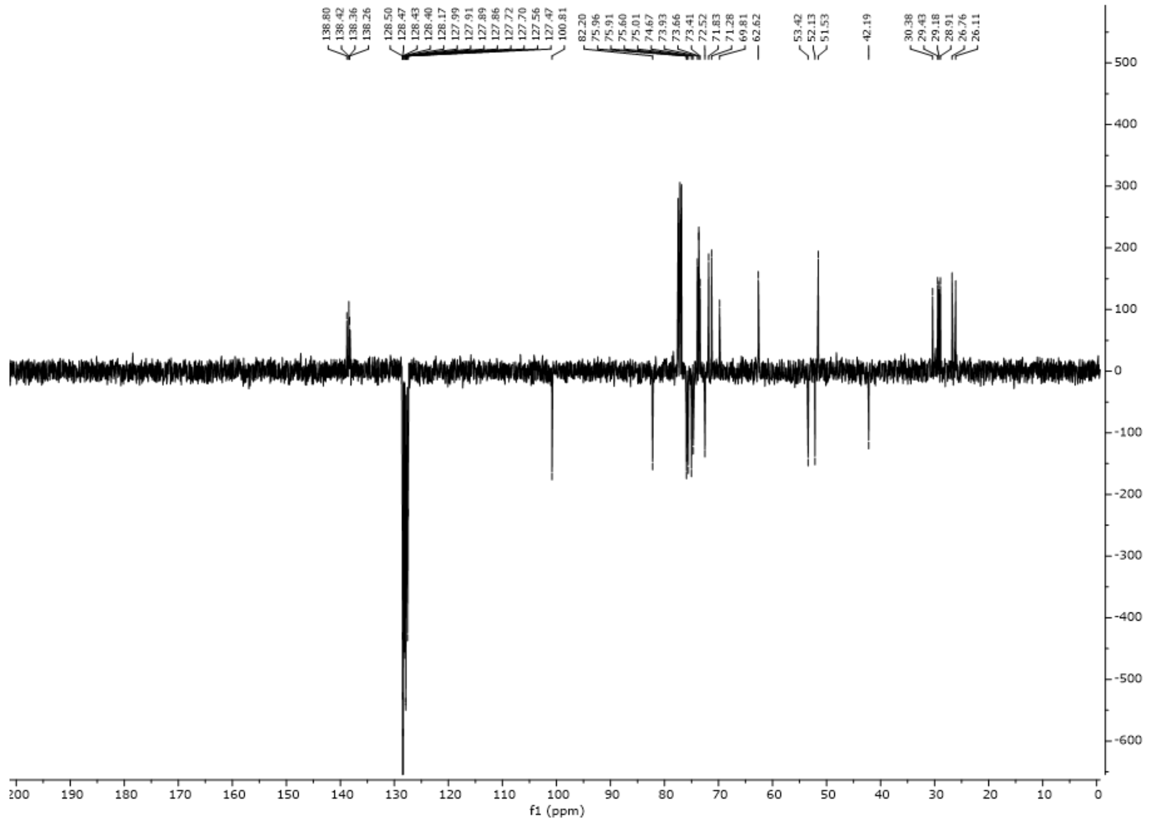

4-*O*-(8-aminooctyl)-β-D-mannose-(1-4)-manno-cyclophellitol (23)

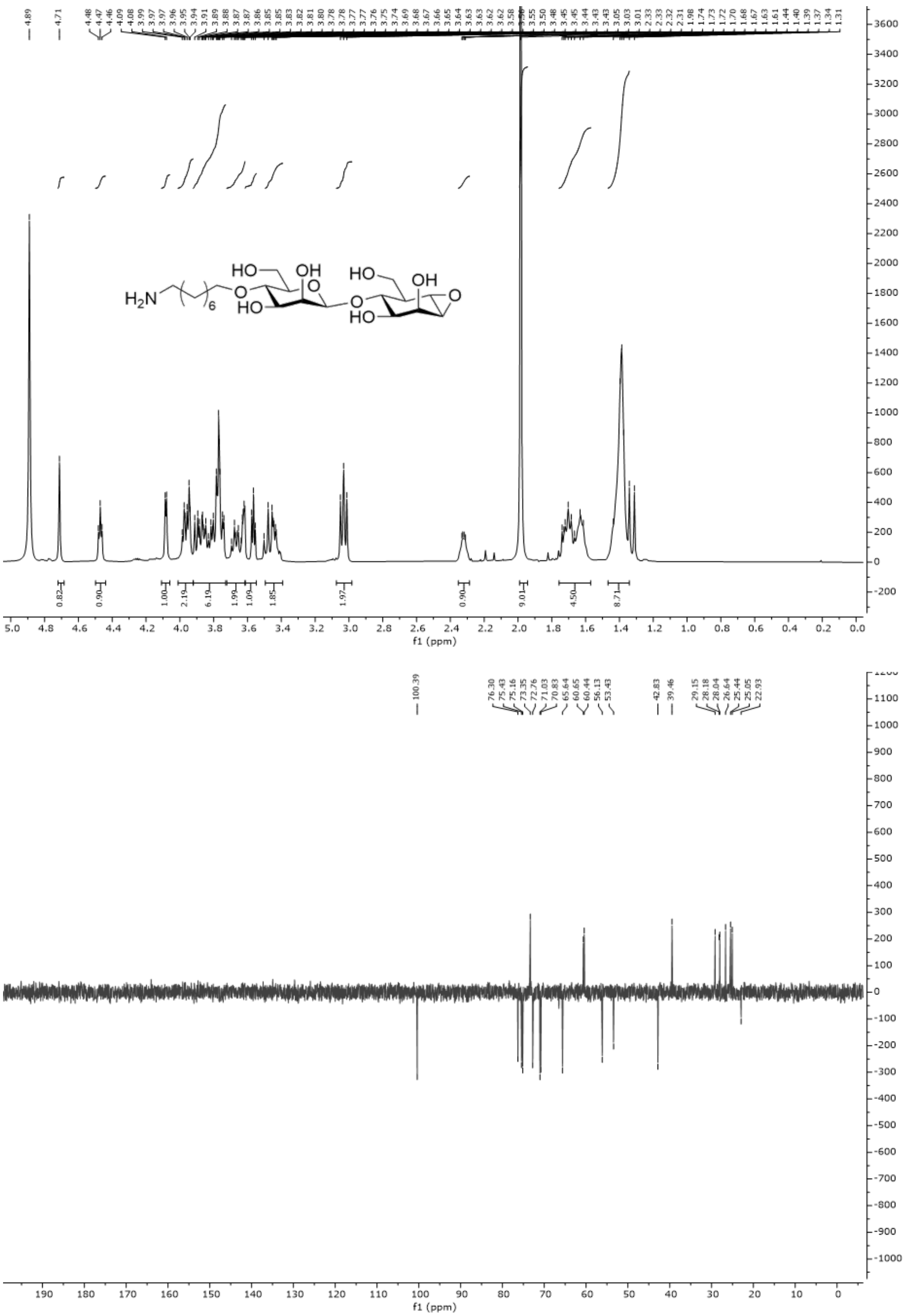

4-*O*-(8-biotinyl-octyl)-β-D-mannose-(1-4)-manno-cyclophellitol (12)

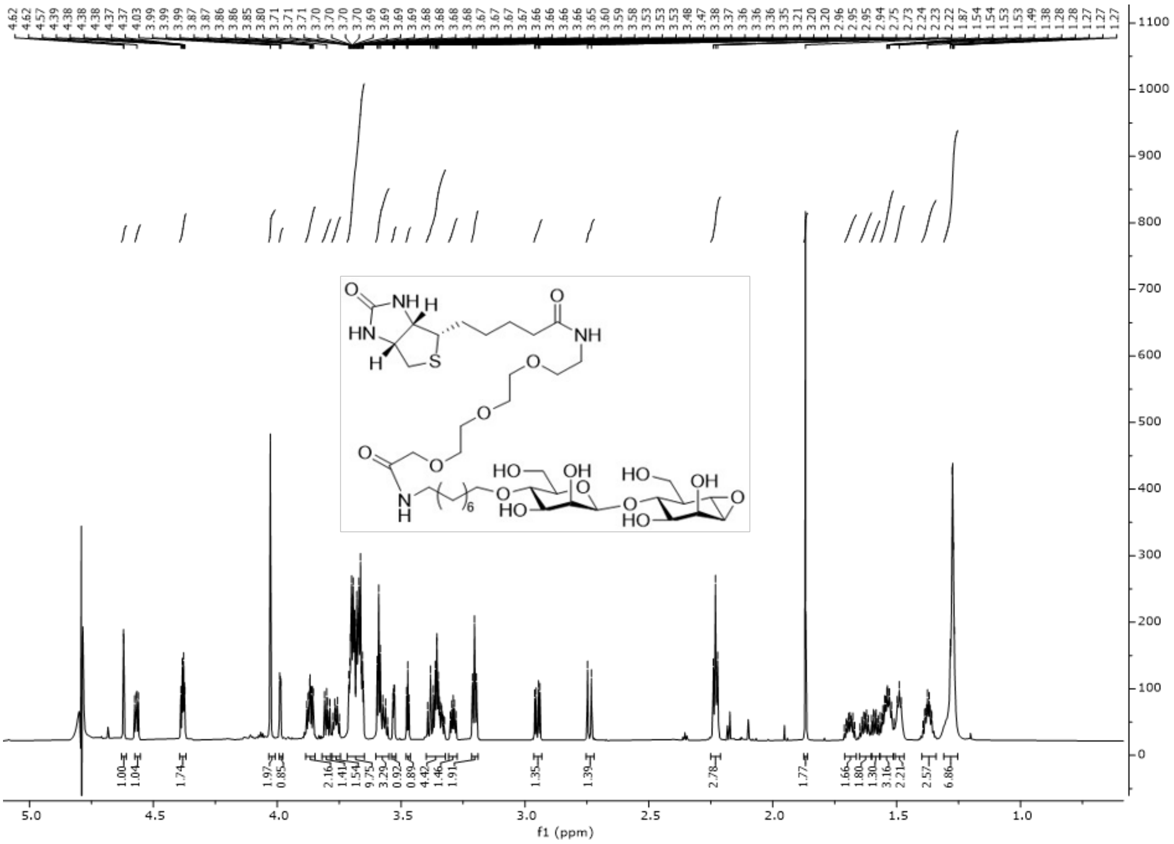

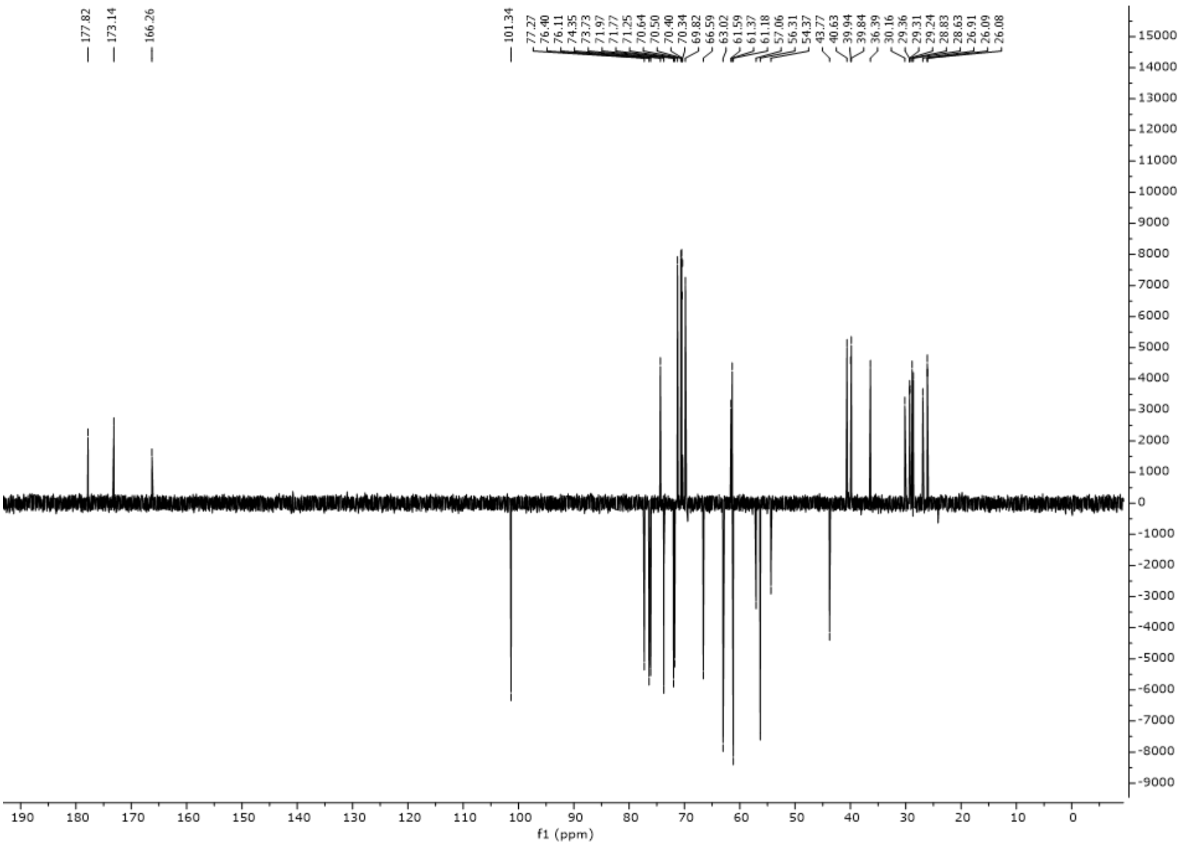

4-*O*-(8-Cy5-octyl)-β-D-mannose-(1-4)-manno-cyclophellitol (13)

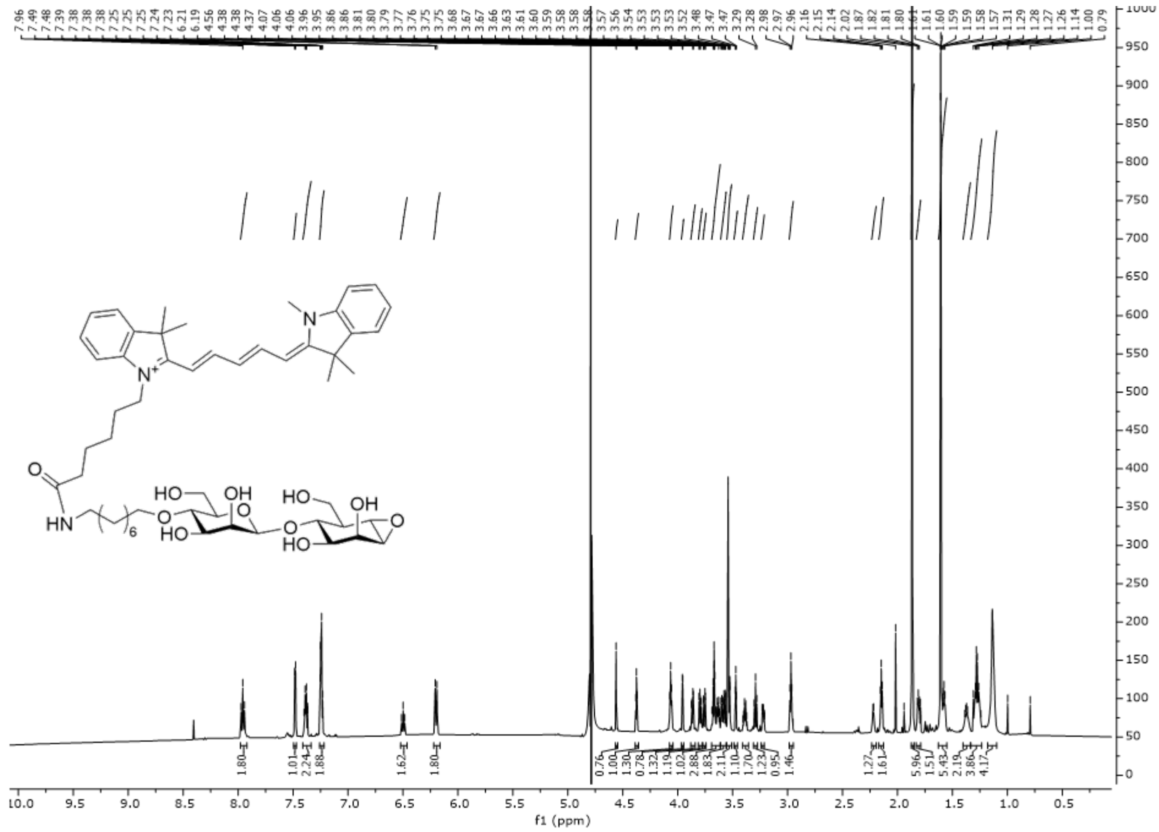

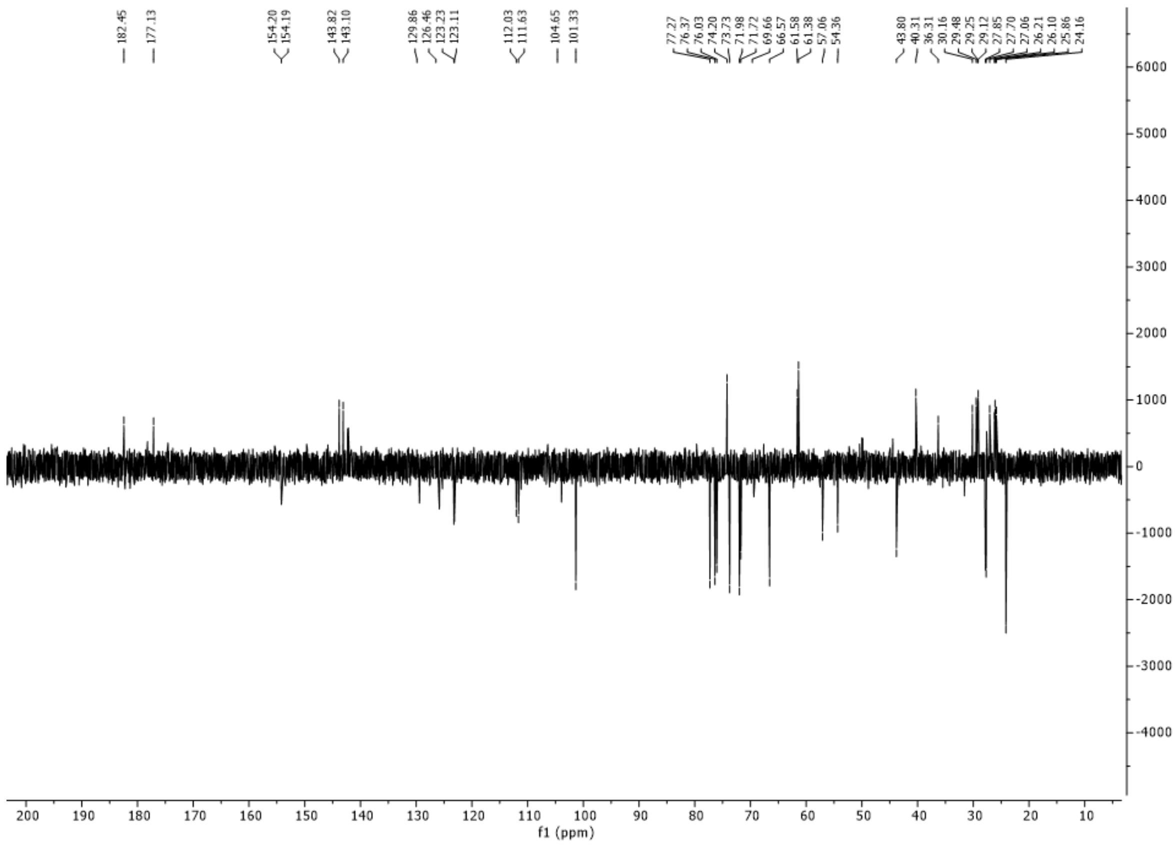

2,3-di-*O*-benzyl-4,6-*O*-benzylidene-(1-4)-β-D-mannopyranosyl-2,3,6-tri-*O*-benzyl-(1-4)-β-D-mannopyranosyl-2,3-di-*O*-benzyl-6-*O*-naphthyl-manno-cyclohexene (7)

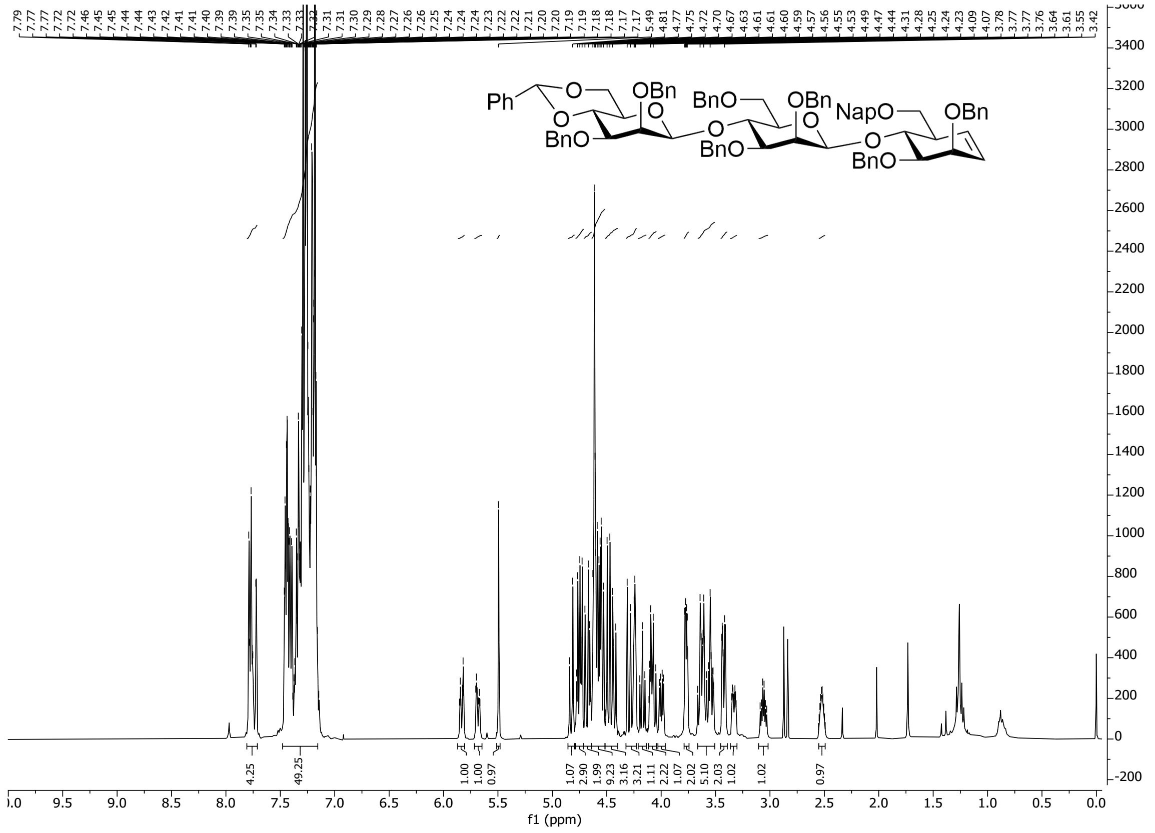

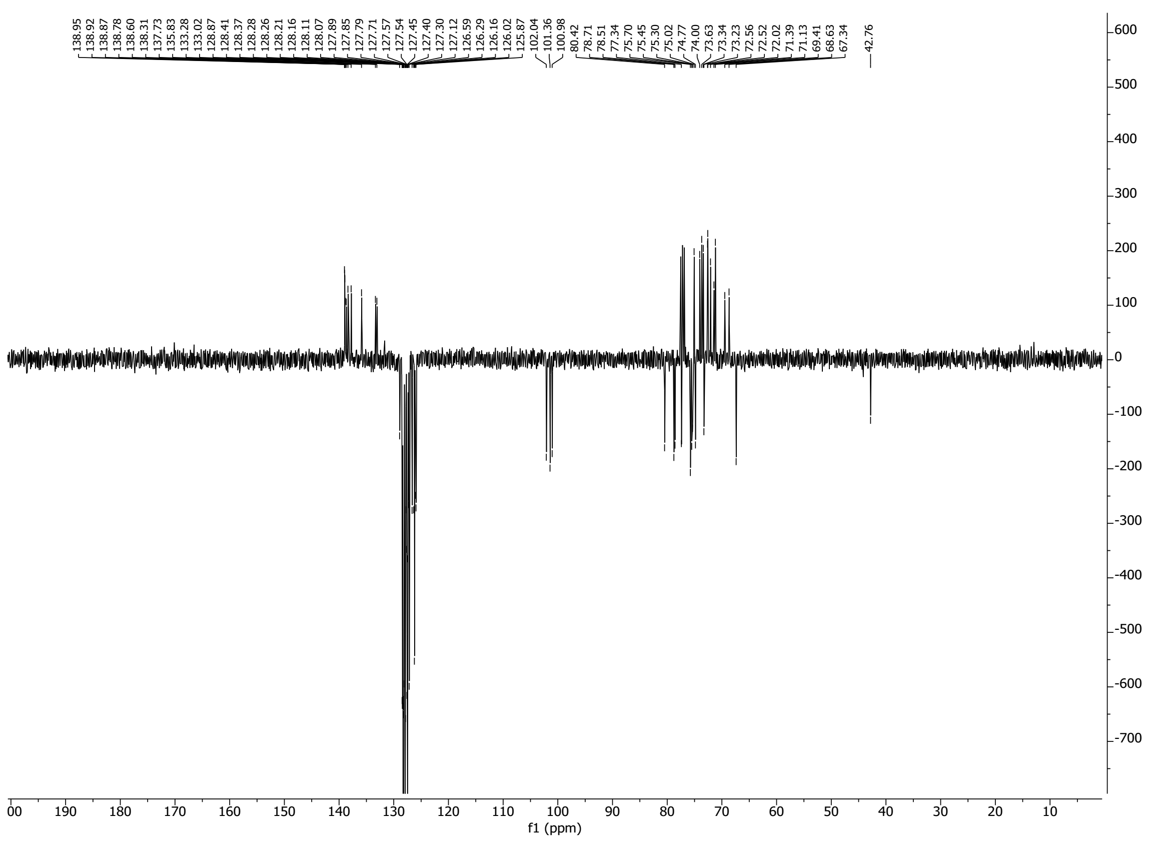

2,3-di-*O*-benzyl-4,6-*O*-benzylidene-(1-4)-β-D-mannopyranosyl-2,3,6-tri-*O*-benzyl-(1-4)-β-D-mannopyranosyl-2,3-di-*O*-benzyl-manno-cyclohexene (24)

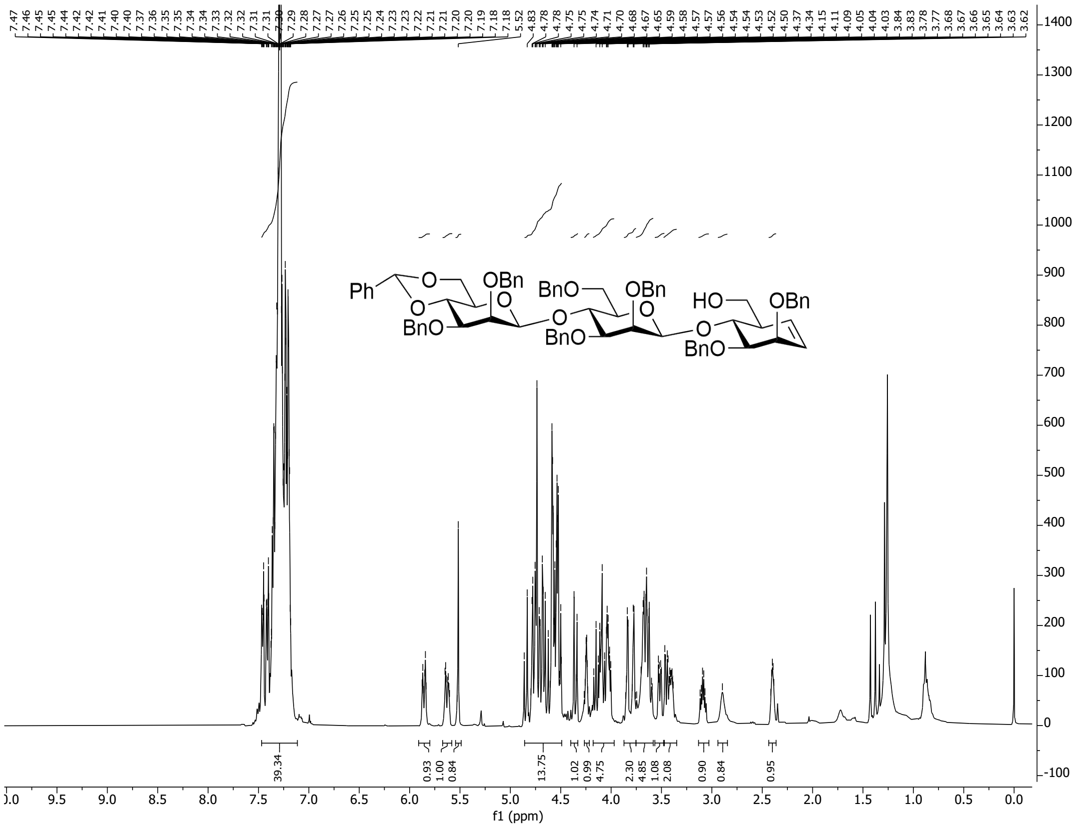

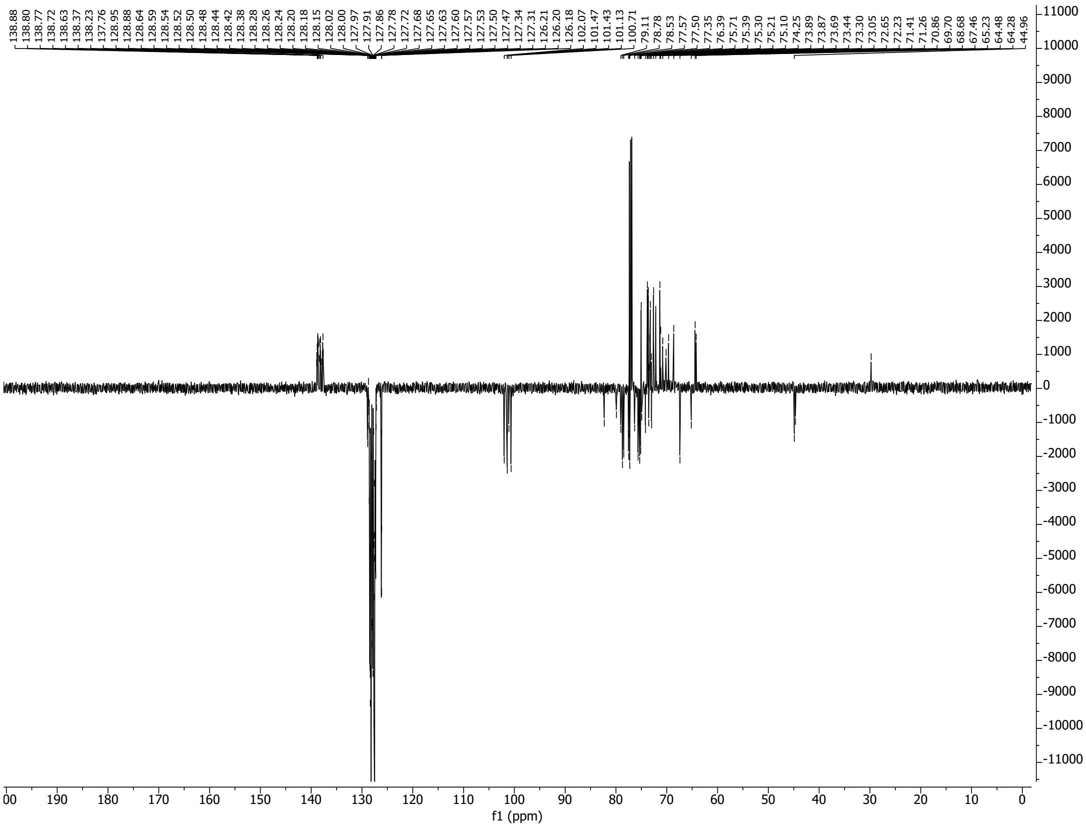

2,3-di-*O*-benzyl-4,6-*O*-benzylidene-(1-4)-β-D-mannopyranosyl-2,3,6-tri-*O*-benzyl-(1-4)-β-D-mannopyranosyl-2,3-di-*O*-benzyl-manno-cyclophellitol (25)

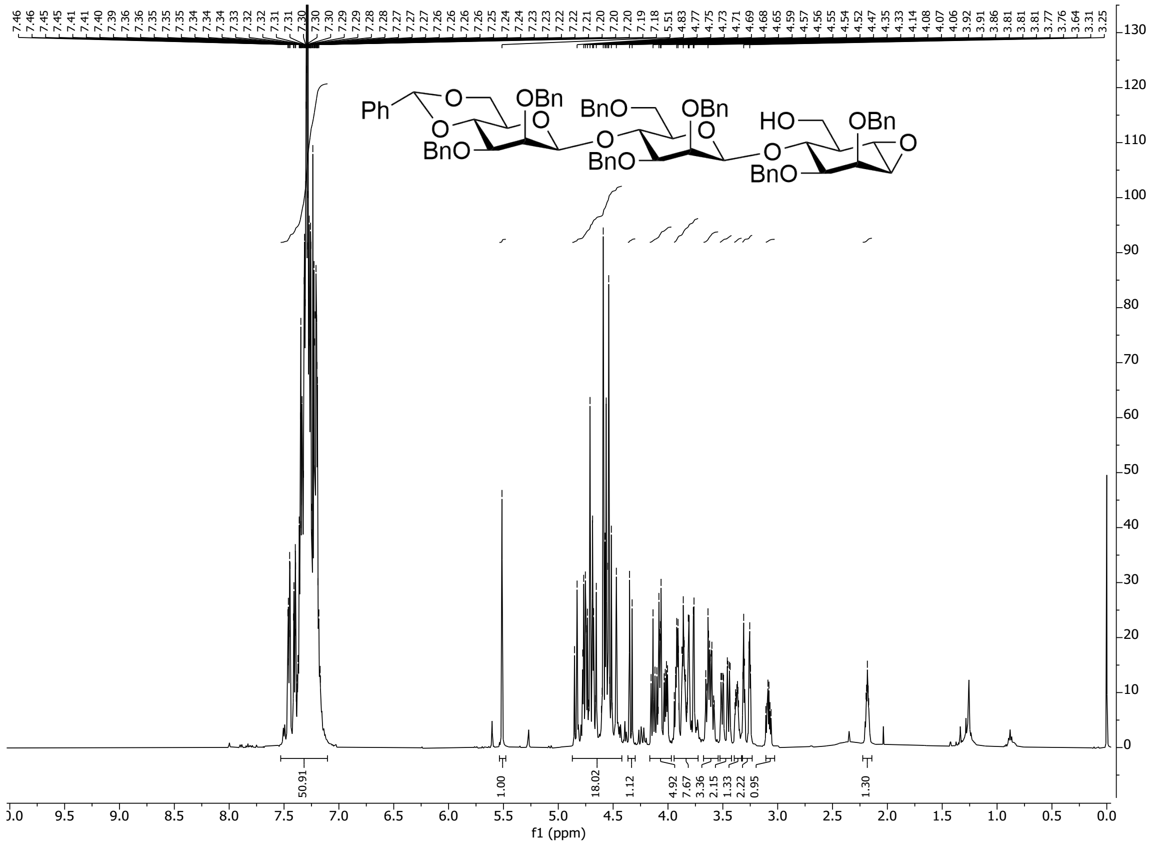

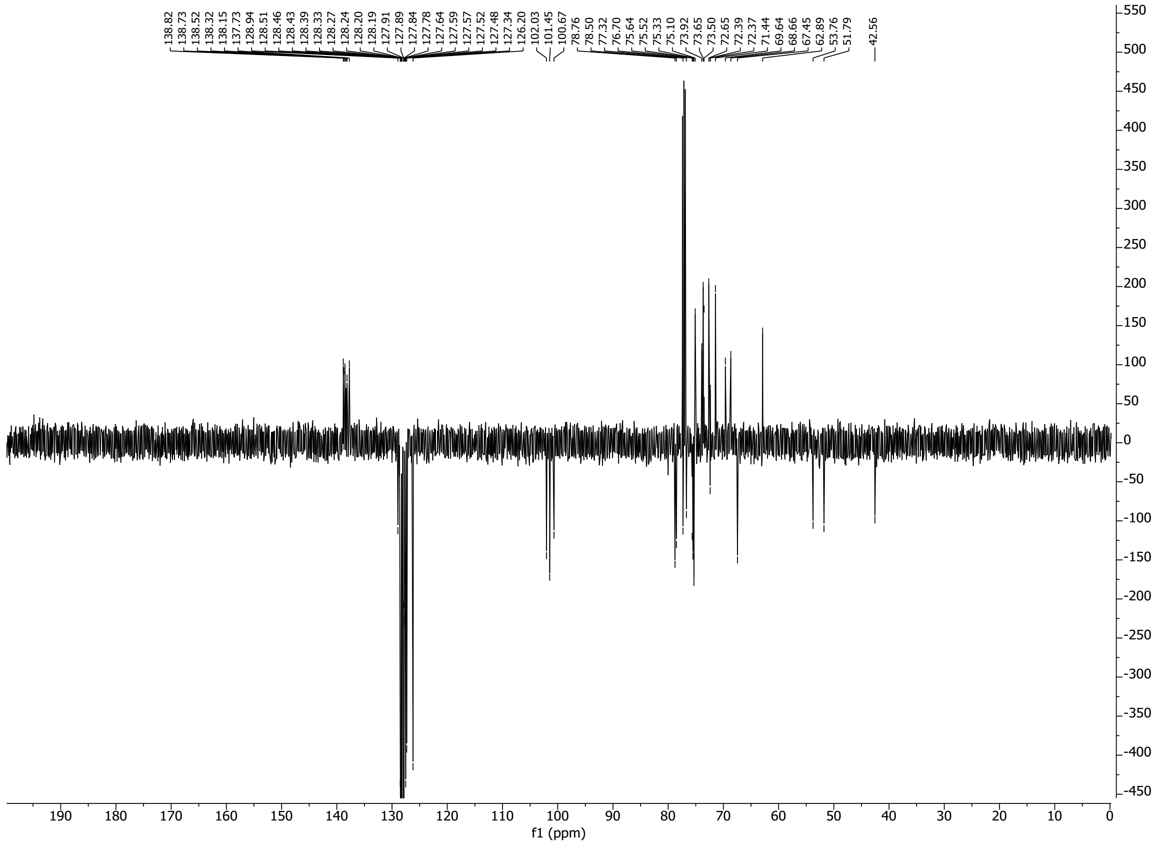

β-D-Mannose-(1-4)-β-D-Mannose-(1-4)-manno-cyclophellitol (16)

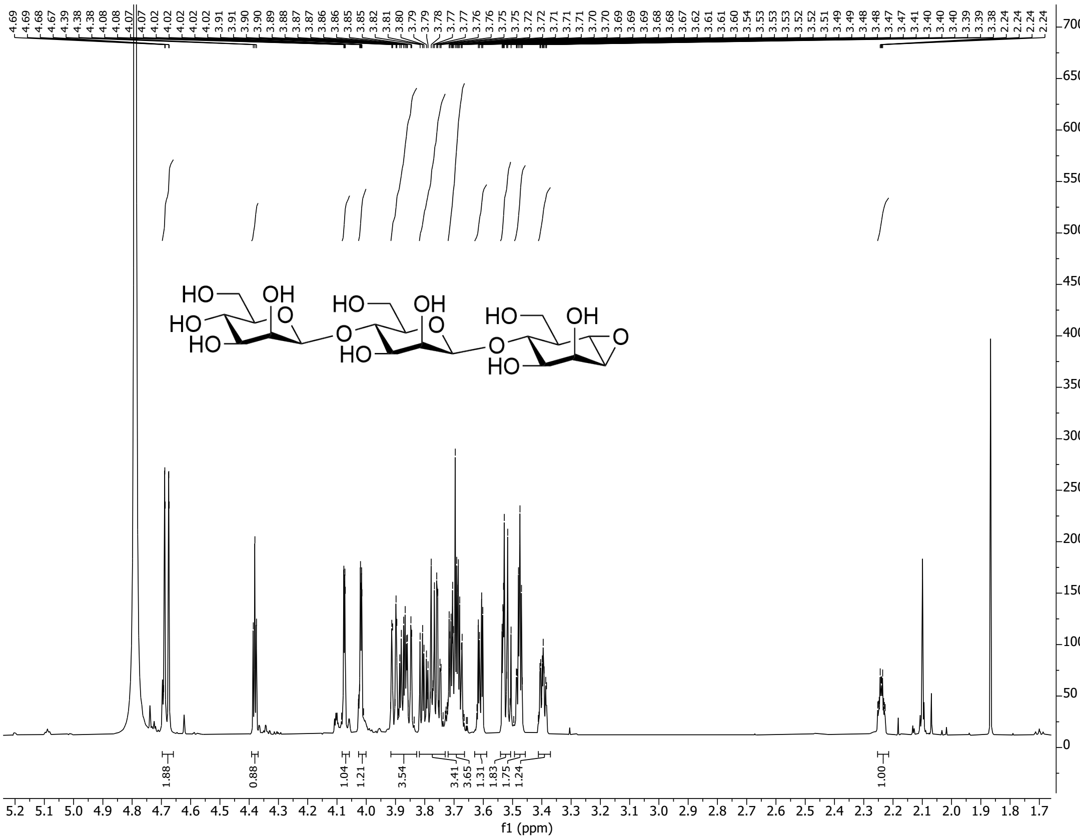

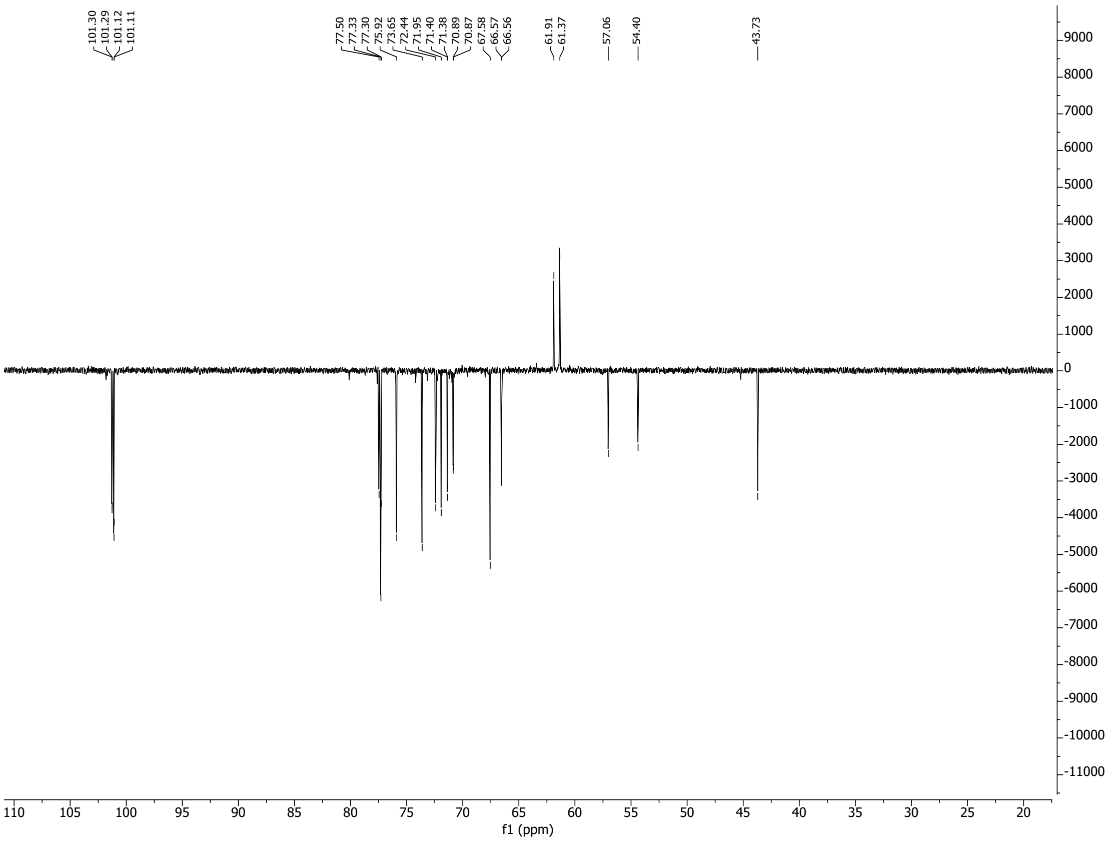

2,3,6-tri-*O*-benzyl-(1-4)-β-D-mannopyranosyl-2,3,6-tri-*O*-benzyl-(1-4)-β-D-mannopyranosyl-2,3-di-*O*-benzyl-6-*O*-naphthyl-manno-cyclohexene (8)

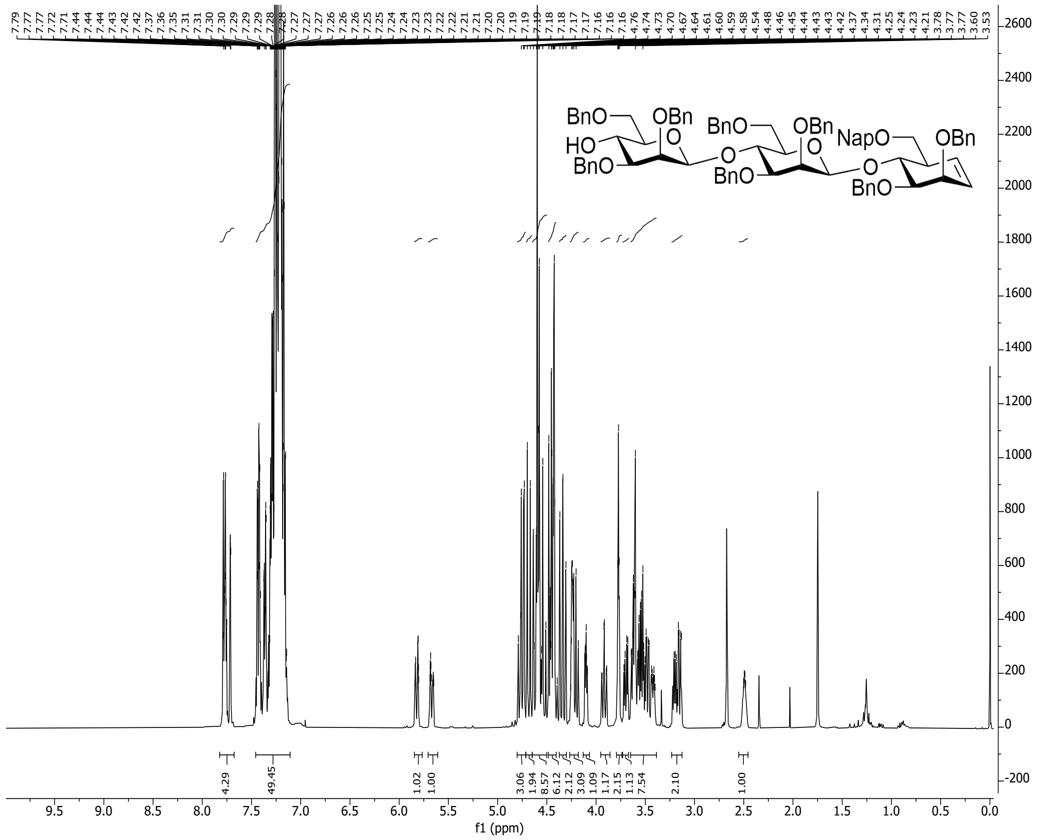

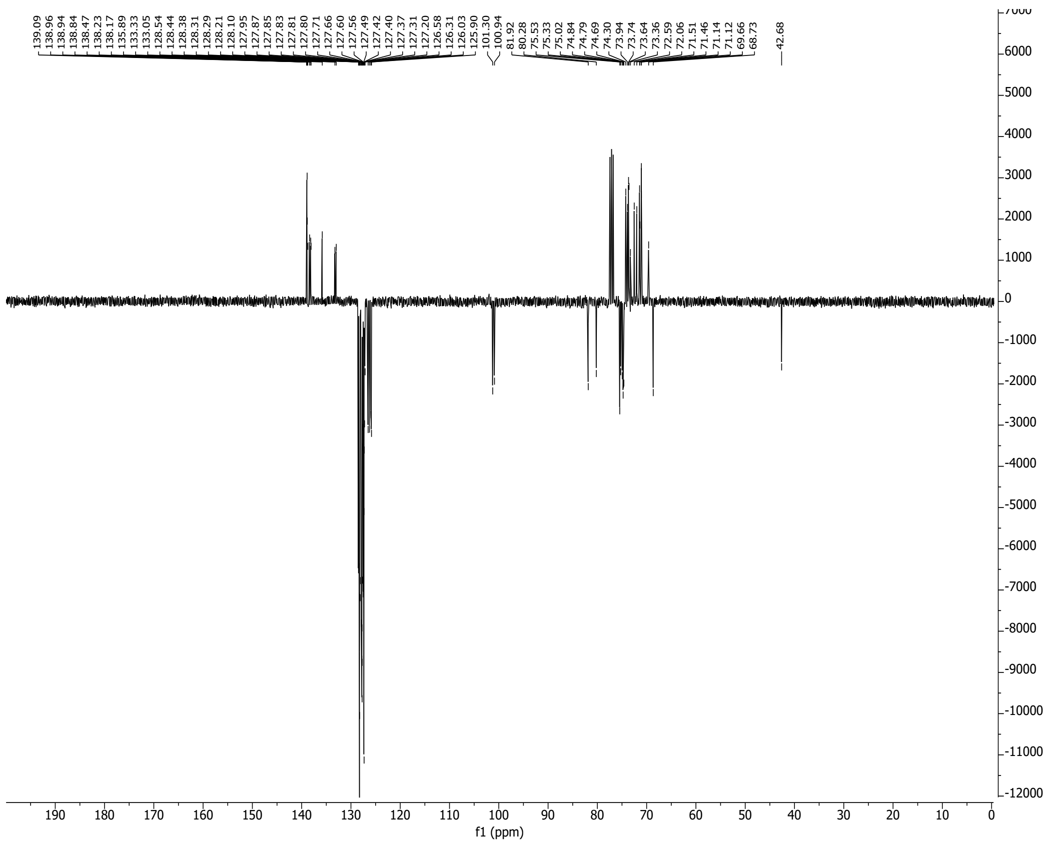

4-*O*-(8-azidooctyl)-2,3,6-tri-*O*-benzyl-(1-4)-β-D-mannopyranosyl-2,3,6-tri-*O*-benzyl-(1-4)-β-D-mannopyranosyl-2,3-di-*O*-benzyl-6-*O*-naphthyl-manno-cyclohexene (9)

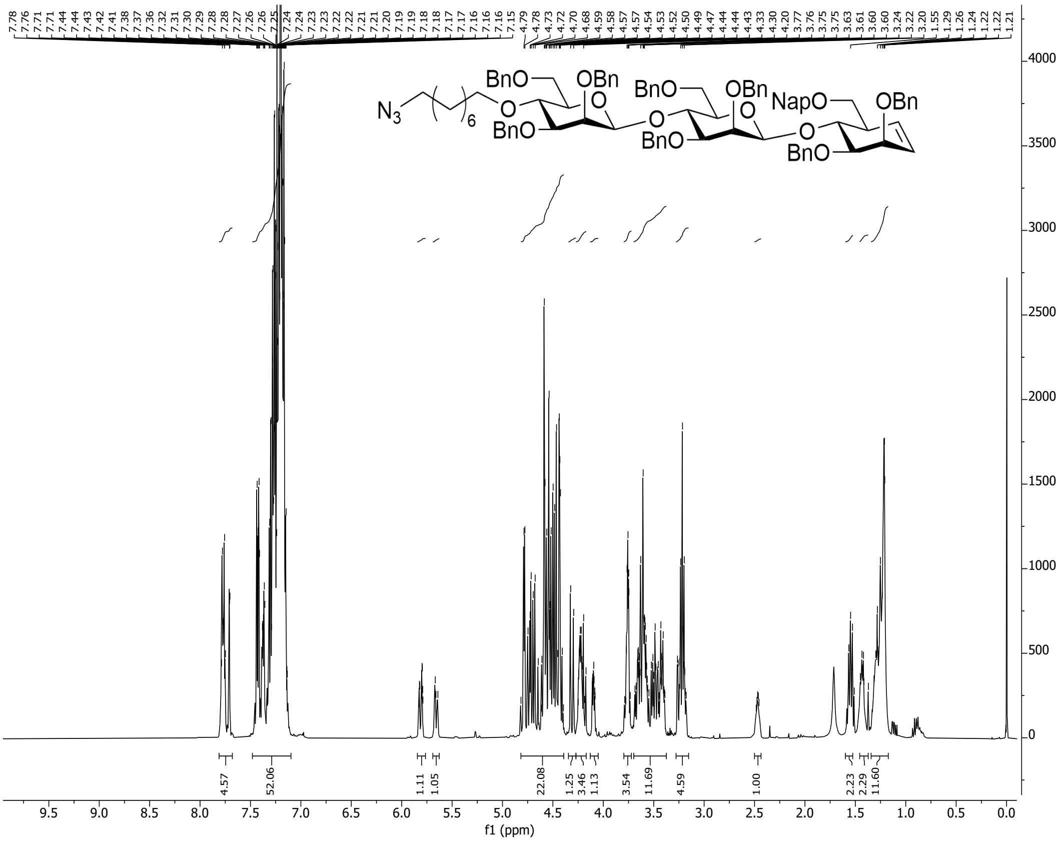

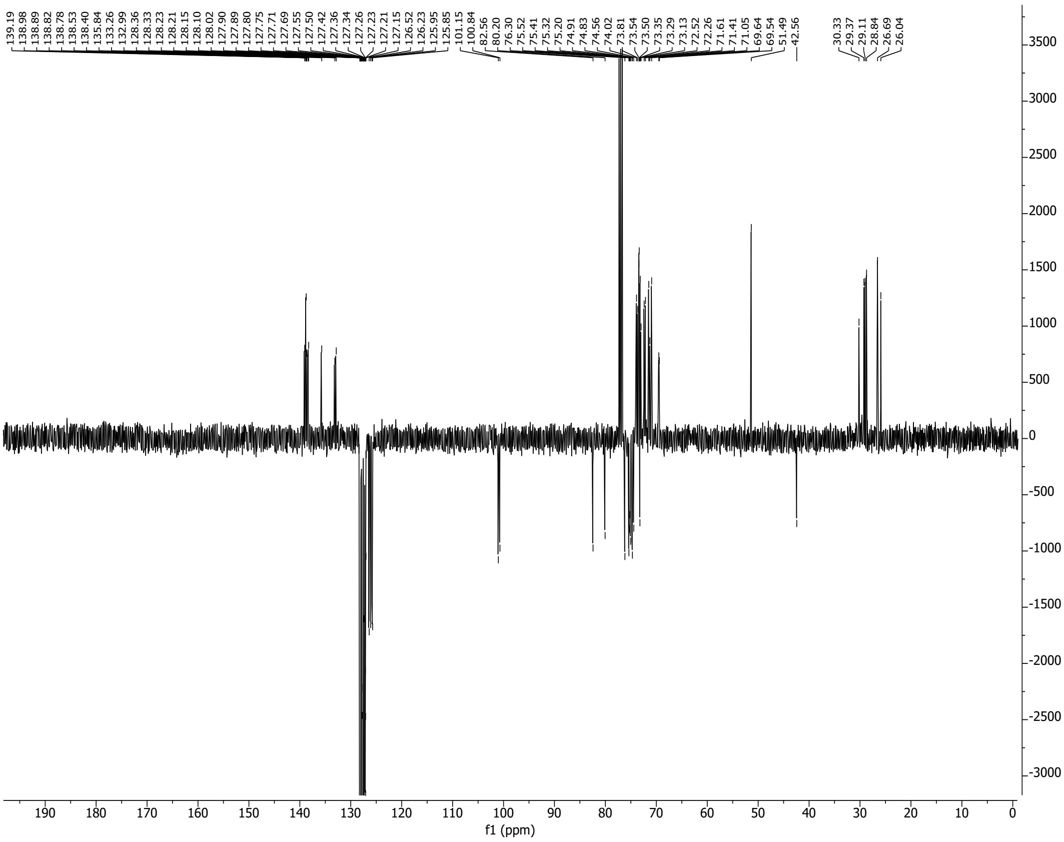

4-*O*-(8-azidooctyl)-2,3,6-tri-*O*-benzyl-(1-4)-β-D-mannopyranosyl-2,3,6-tri-*O*-benzyl-(1-4)-β-D-mannopyranosyl-2,3-di-*O*-benzyl-manno-cyclohexene (26)

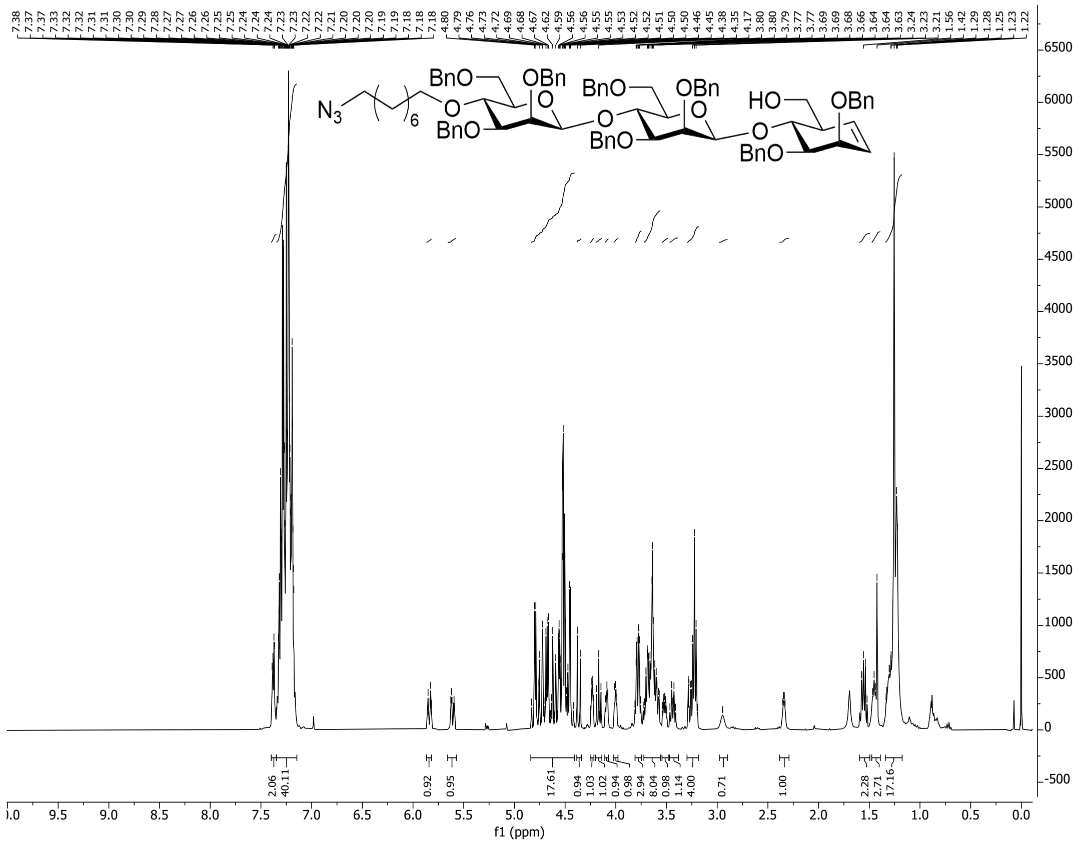

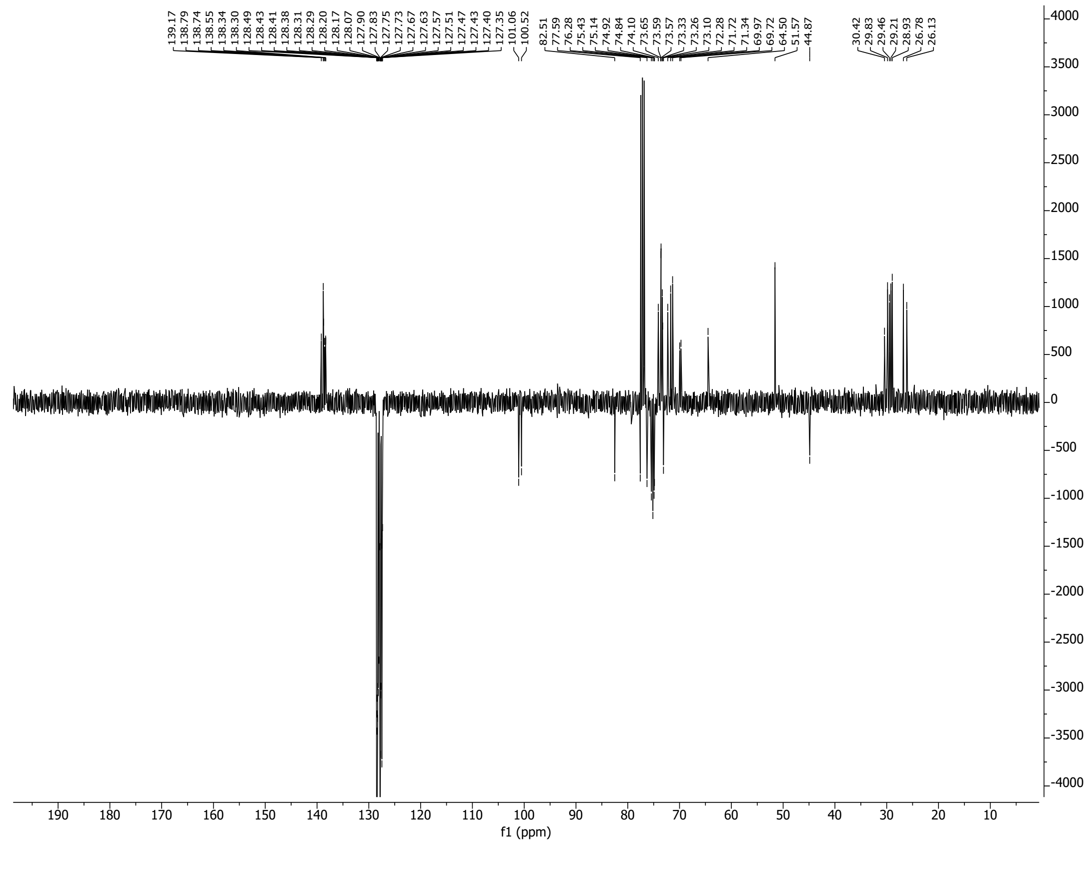

4-*O*-(8-azidooctyl)-2,3,6-tri-*O*-benzyl-(1-4)-β-D-mannopyranosyl-2,3,6-tri-*O*-benzyl-(1-4)-β-D-mannopyranosyl-2,3-di-*O*-benzyl-manno-cyclophellitol (11)

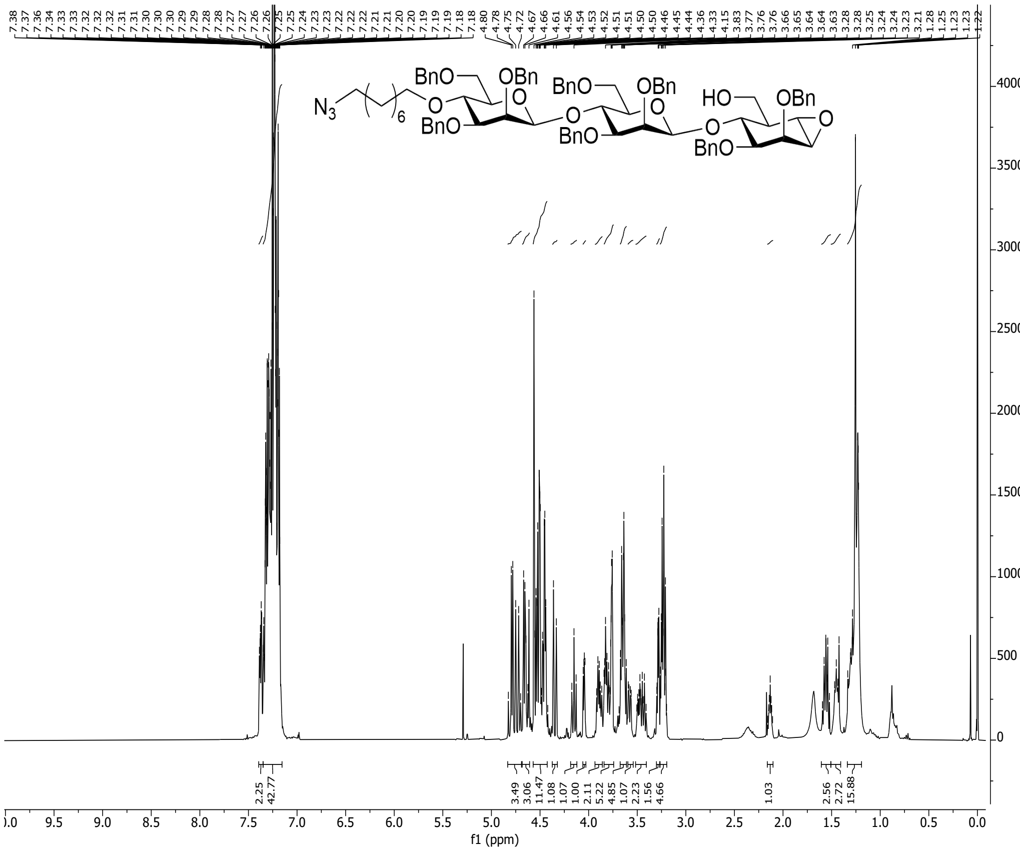

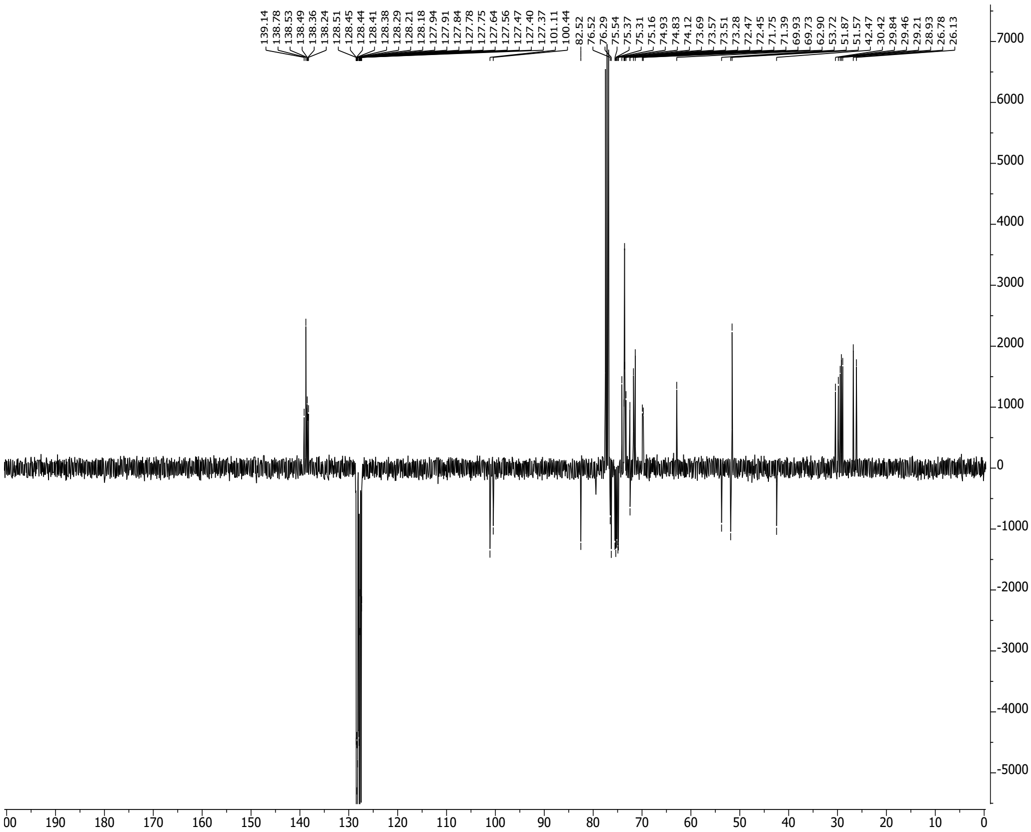

4-*O*-(8-aminooctyl)-β-D-mannose-(1-4)-β-D-mannose-(1-4)-manno-cyclophellitol (27)

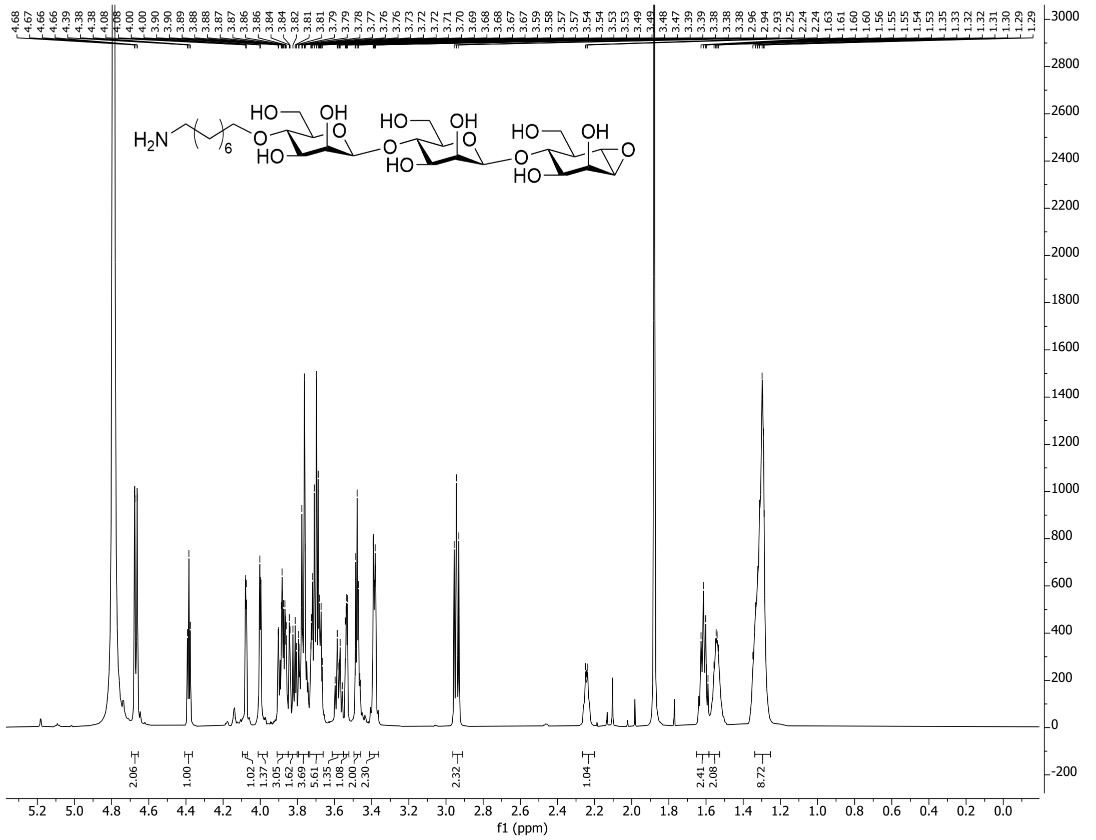

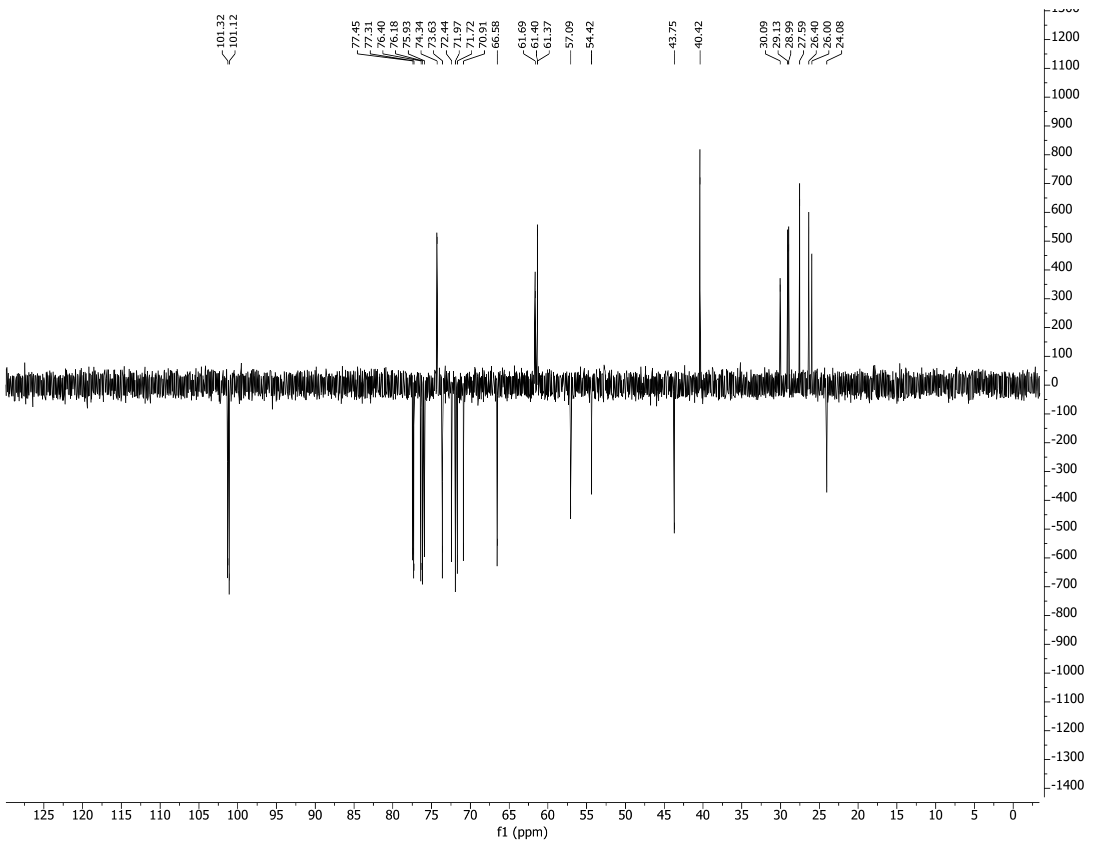

4-*O*-(8-Cy5-octyl)-β-D-mannose-(1-4)-manno-cyclophellitol (1)

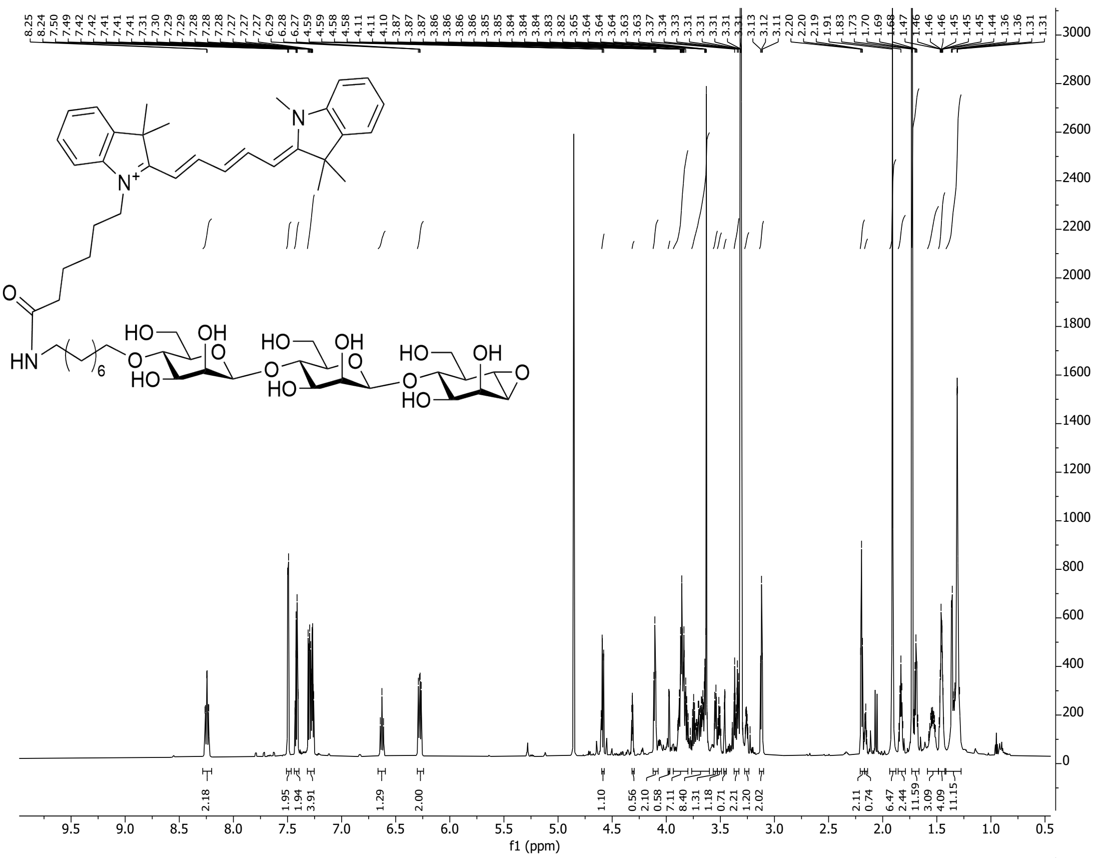

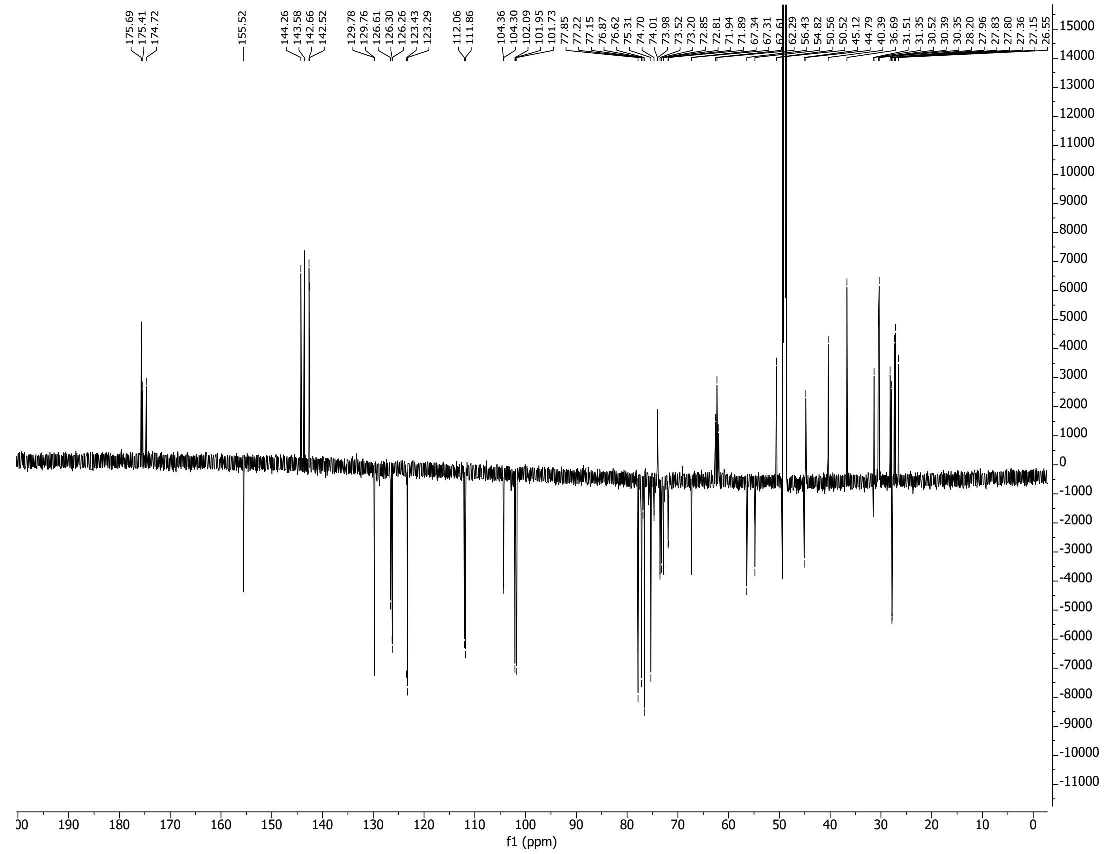

2,3-di-*O*-benzyl-4,6-*O*-benzylidene-(1-4)-β-D-mannopyranosyl-6-*O*-benzoyl-2,3-di-*O*-benzyl-cyclohexene (28)

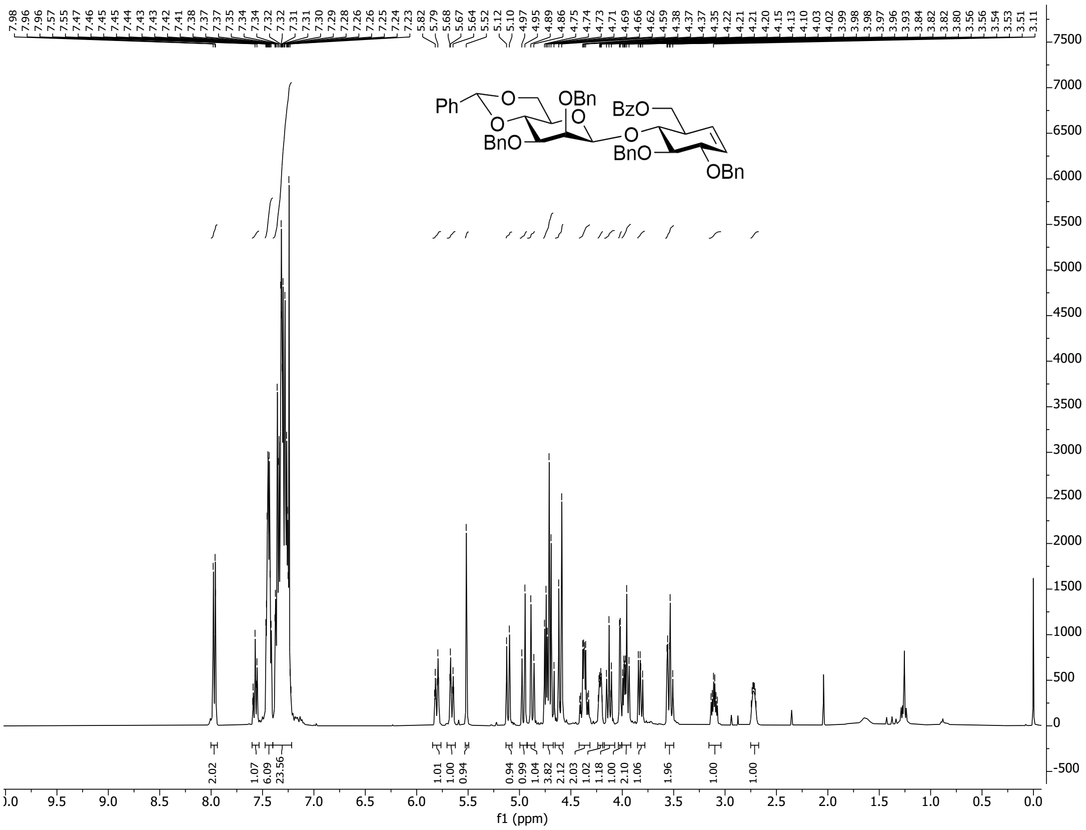

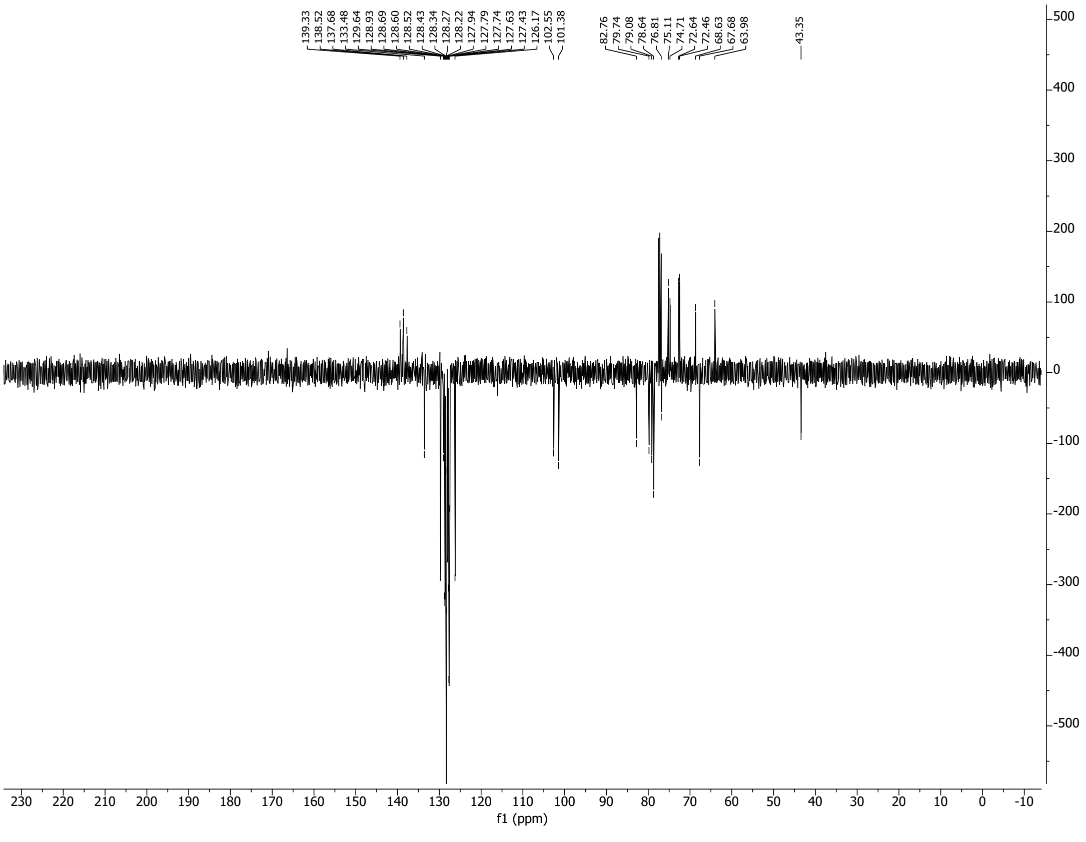

2,3-di-*O*-benzyl-4,6-*O*-benzylidene-(1-4)-β-D-mannopyranosyl-2,3-di-*O*-benzyl-cyclohexene (29)

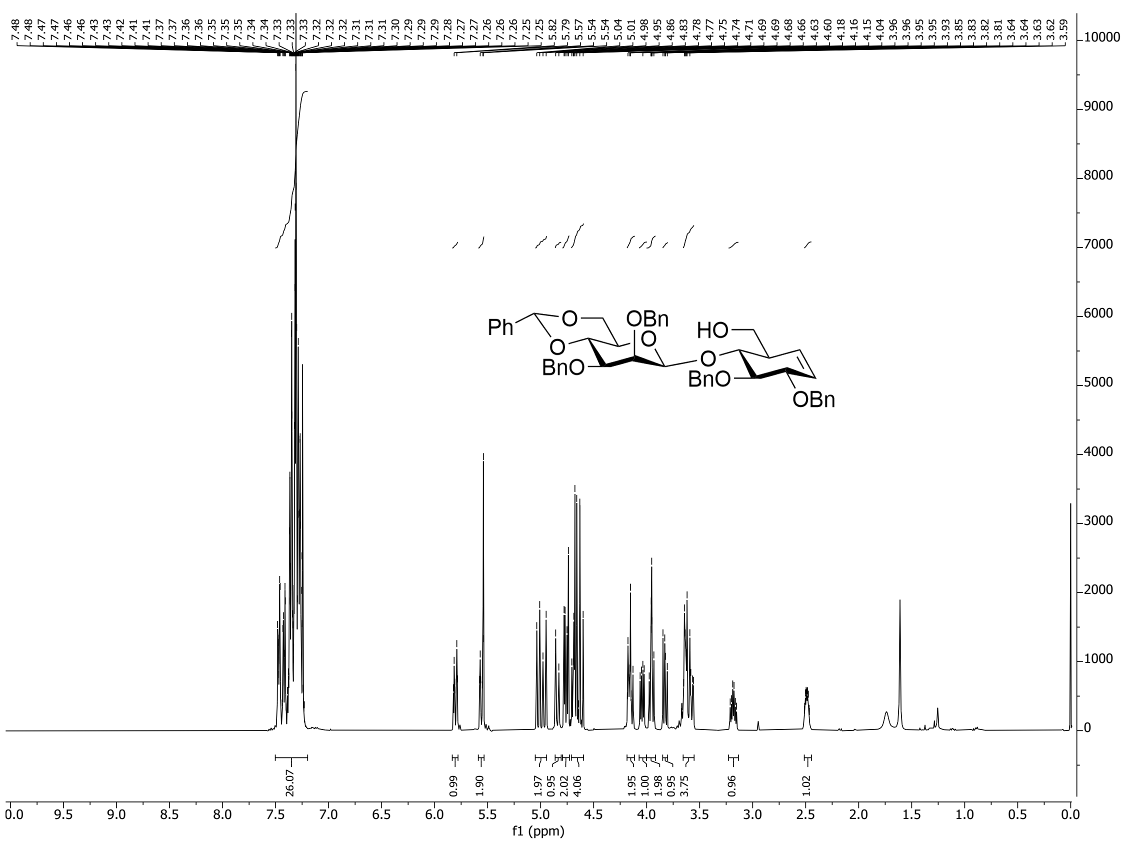

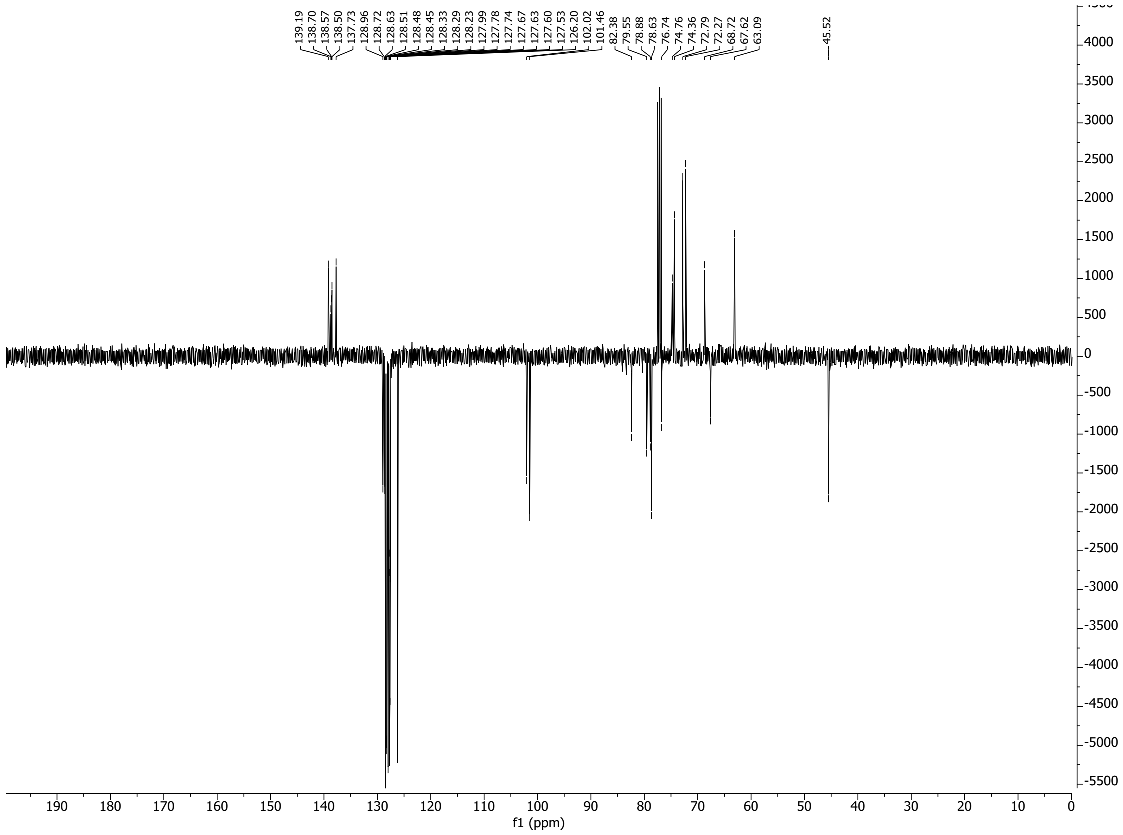

2,3-di-*O*-benzyl-4,6-*O*-benzylidene-(1-4)-β-D-mannopyranosyl-2,3-di-*O*-benzyl-cyclophellitol (30)

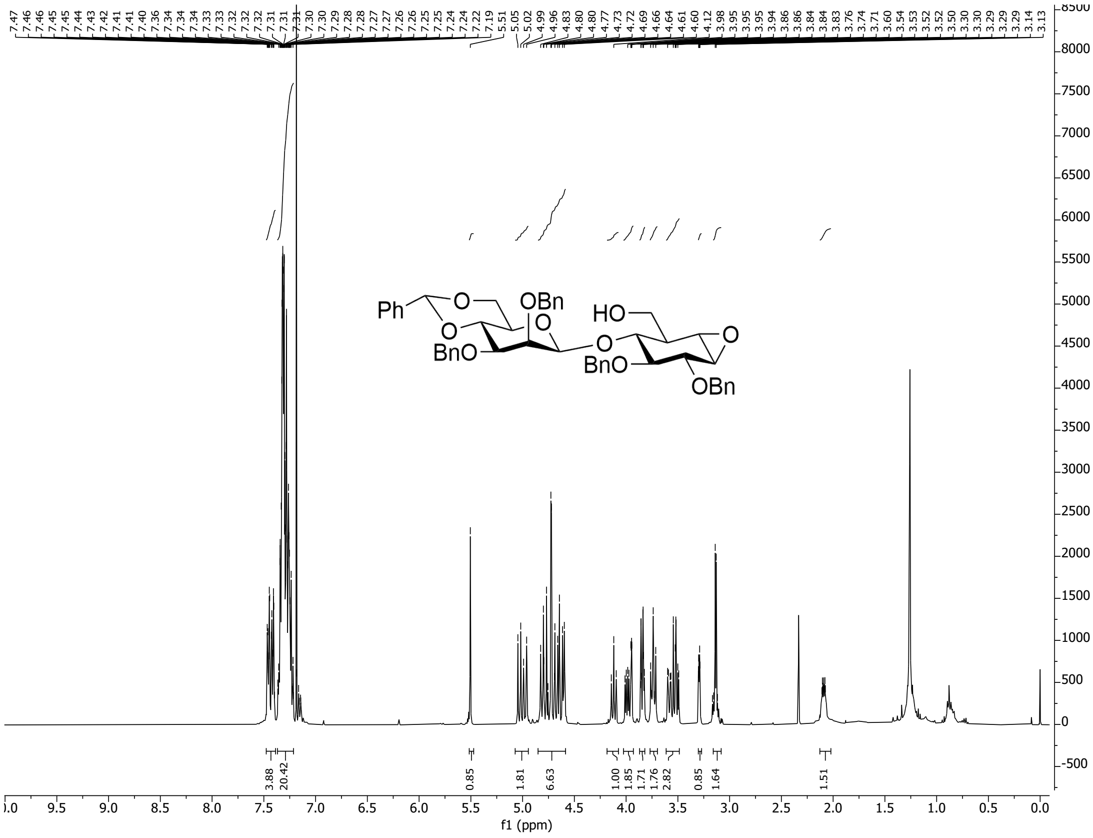

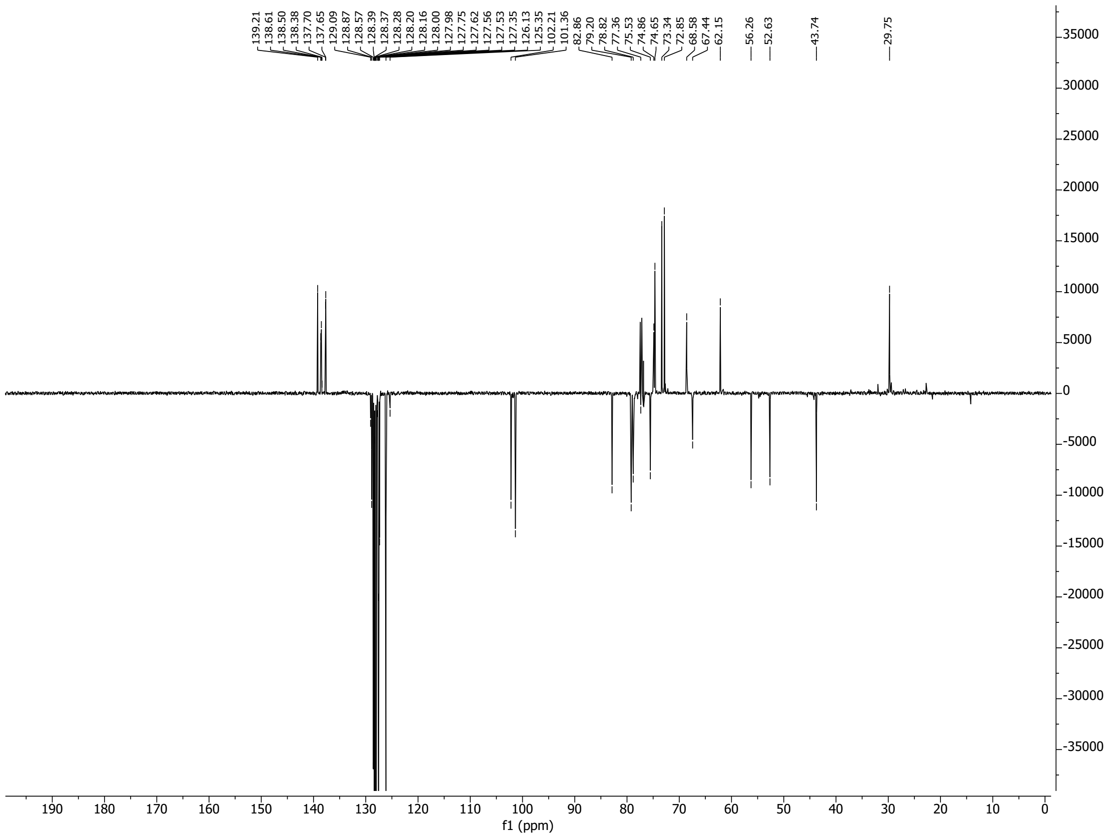

β-D-Mannose-(1-4)-cyclophellitol (17)

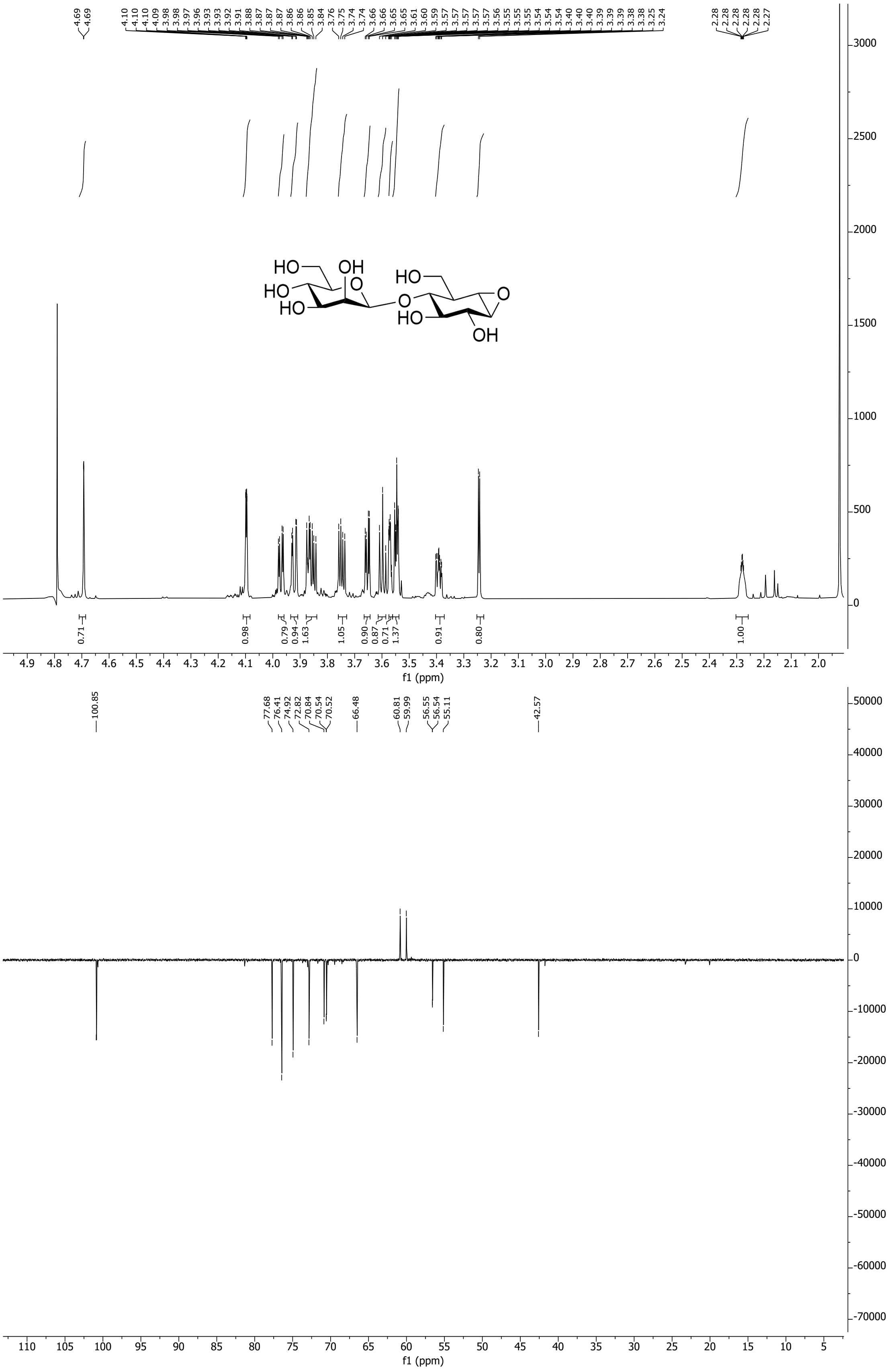

2,3-di-*O*-benzyl-4,6-*O*-benzylidene-(1-4)-β-D-mannopyranosyl-2,3-di-*O*-benzyl-6-*O*-naphthyl-gluco-cyclohexene (31)

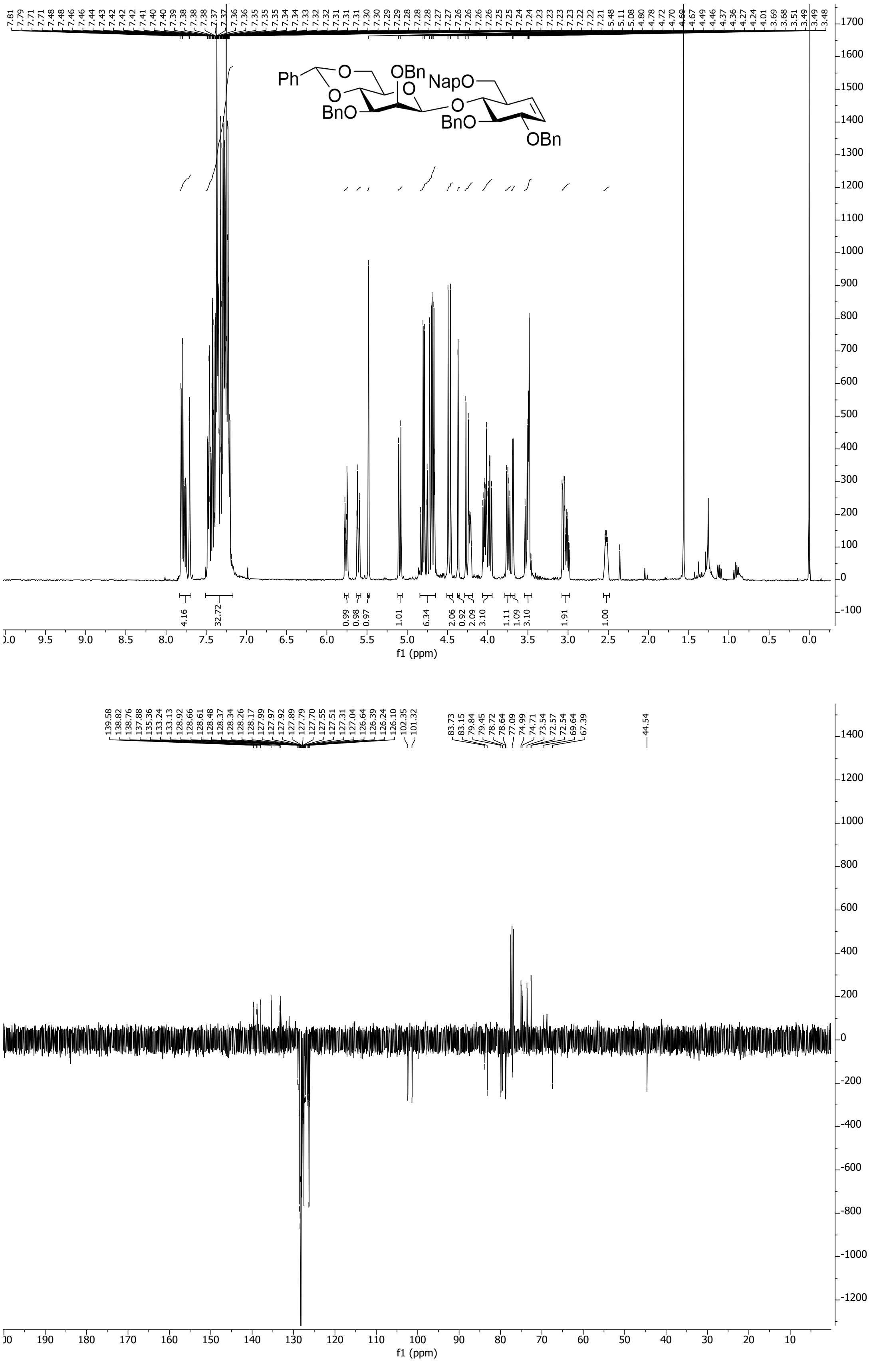

2,3,6-di-*O*-benzyl-(1-4)-β-D-mannopyranosyl-2,3-di-*O*-benzyl-6-*O*-naphthyl-gluco-cyclohexene (32)

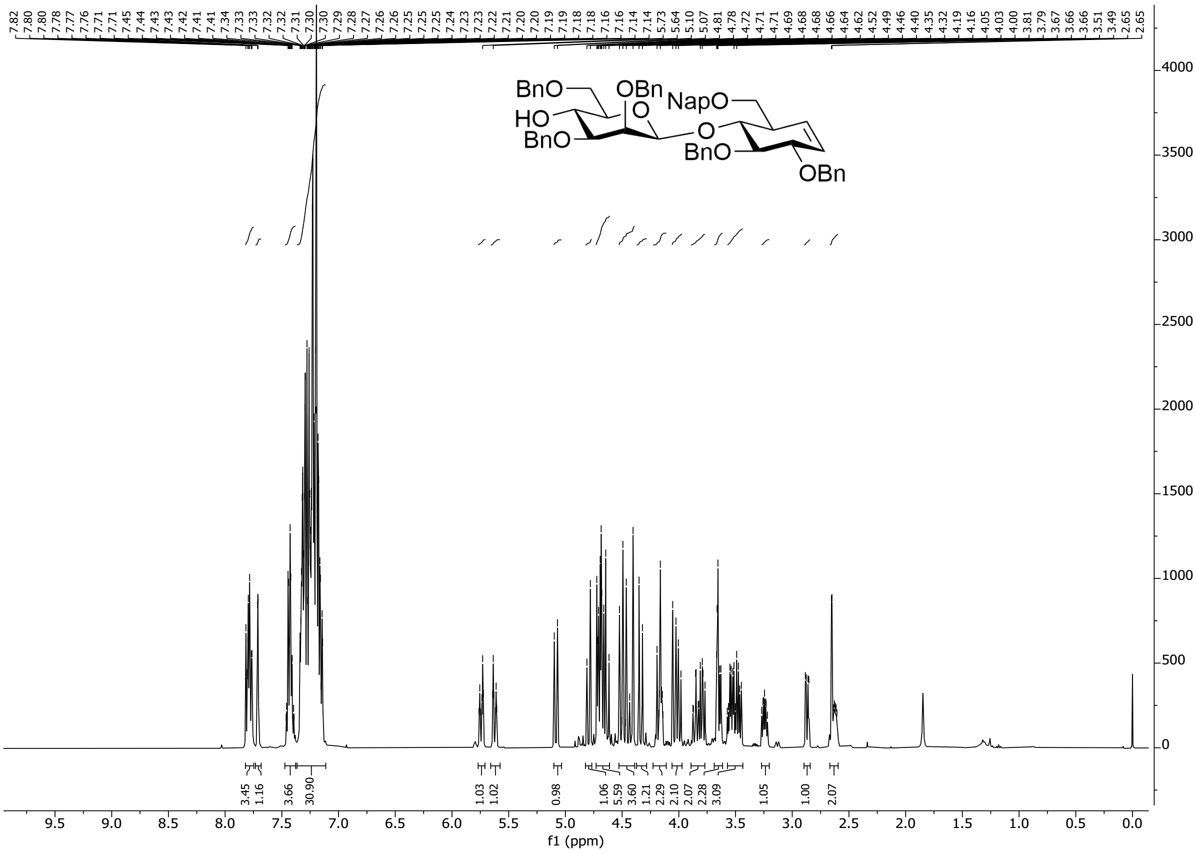

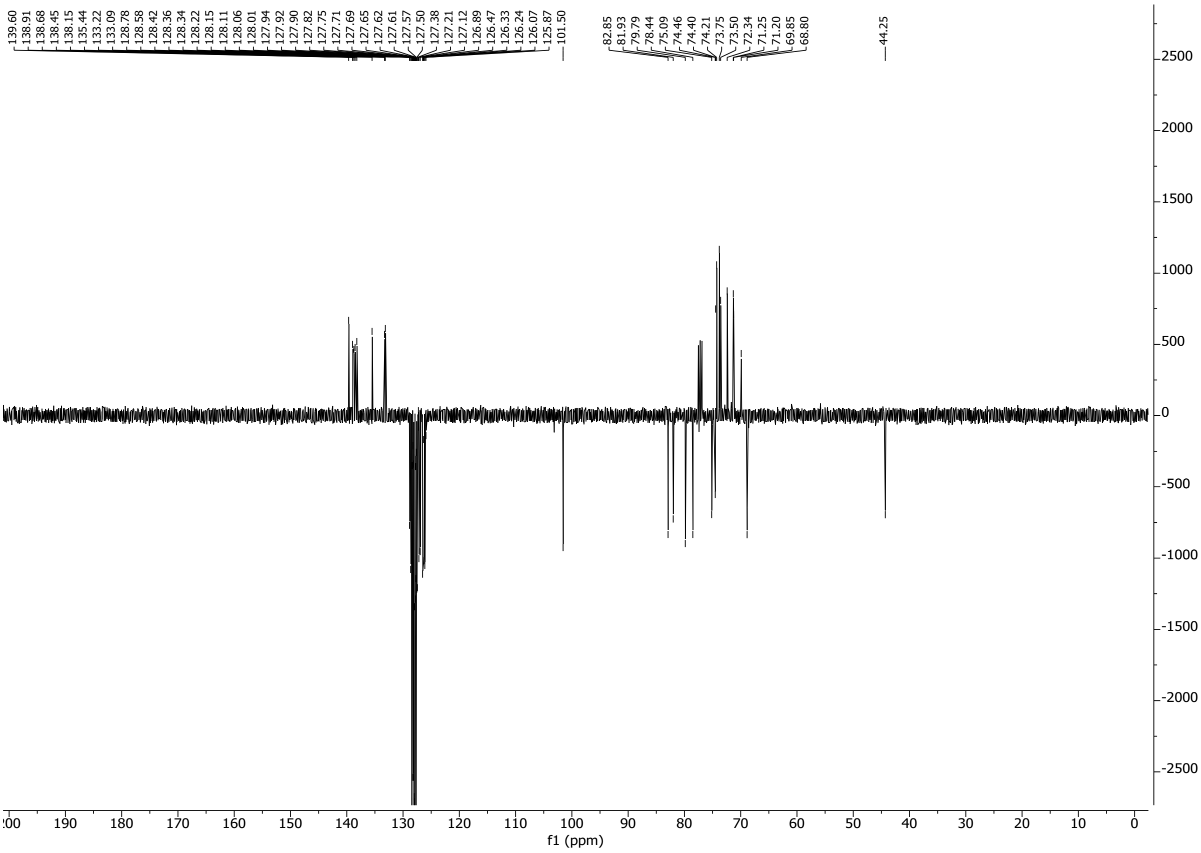

4-*O*-(8-azidooctyl)-2,3,6-tri-*O*-benzyl-(1-4)-β-D-mannopyranosyl-2,3-di-*O*-benzyl-6-*O*-naphthyl-gluco-cyclohexene (33)

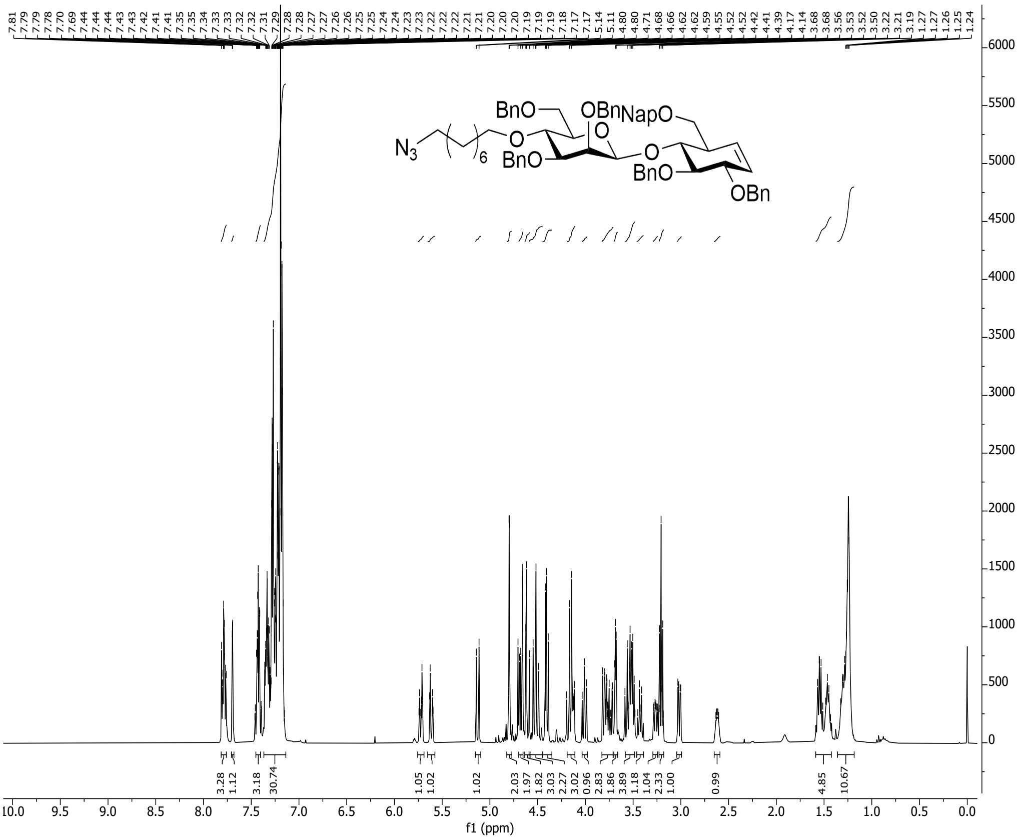

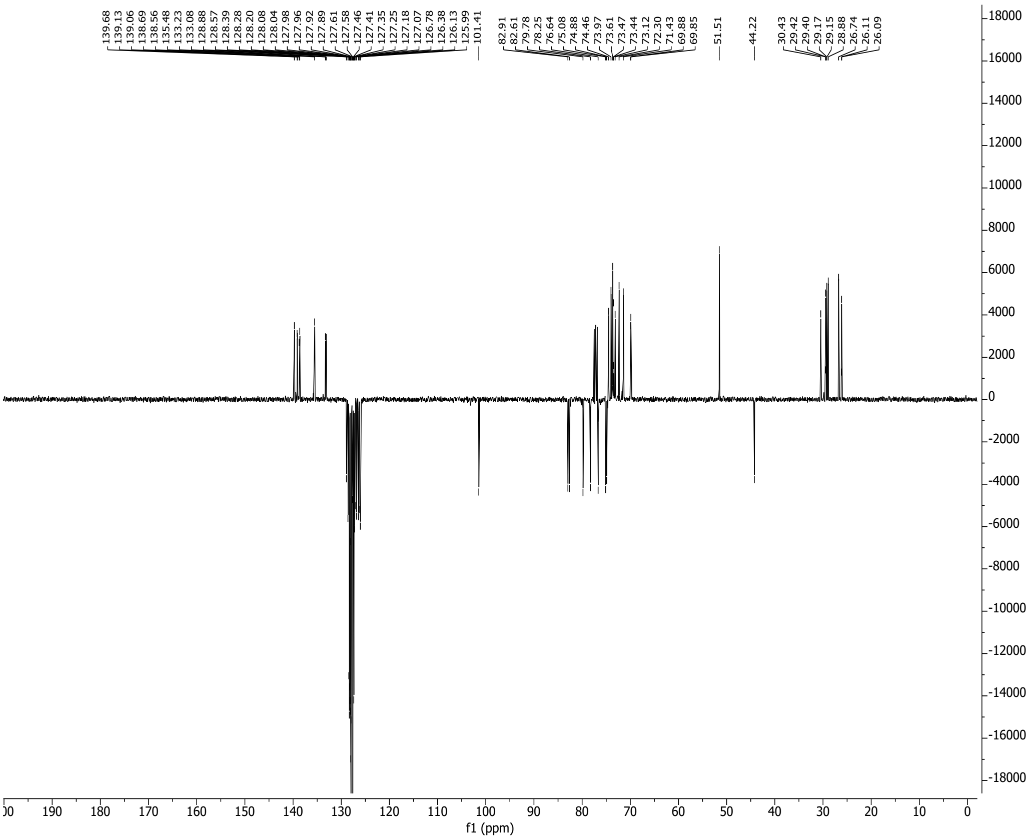

4-*O*-(8-azidooctyl)-2,3,6-tri-*O*-benzyl-(1-4)-β-D-mannopyranosyl-2,3-di-*O*-benzyl-gluco-cyclohexene (34)

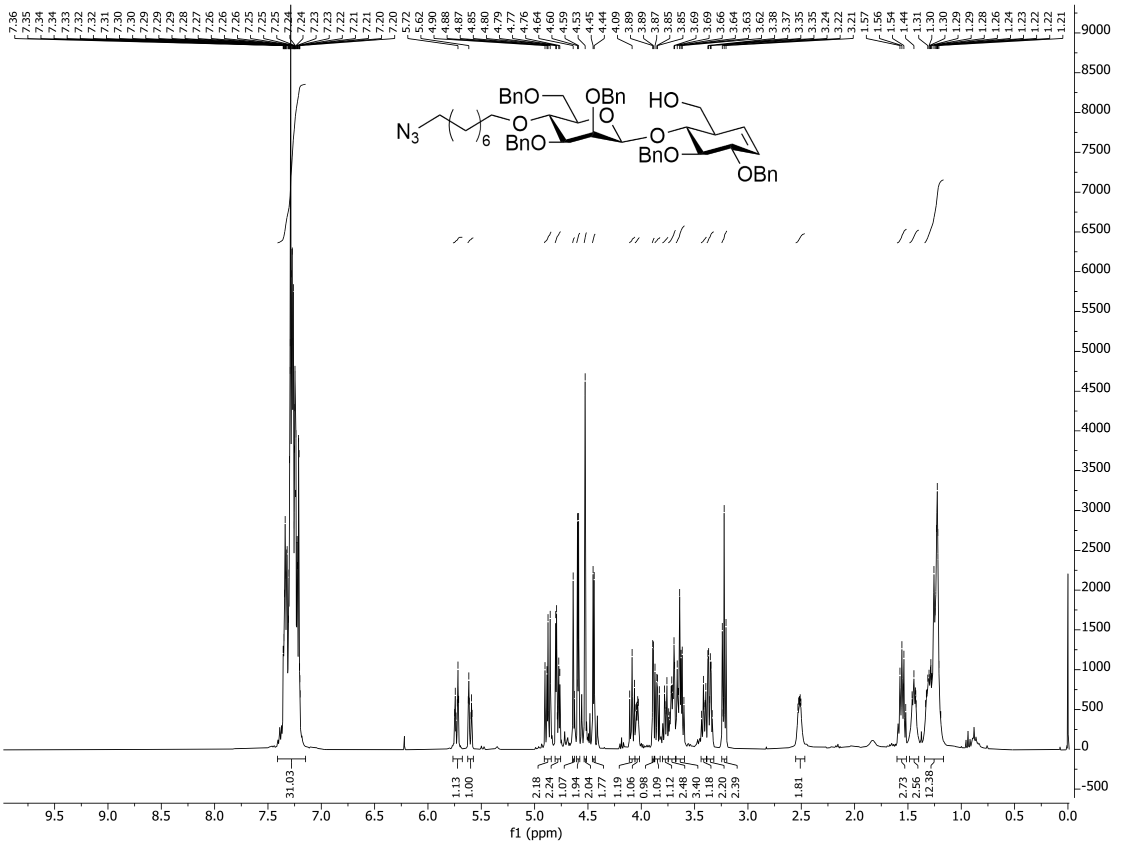

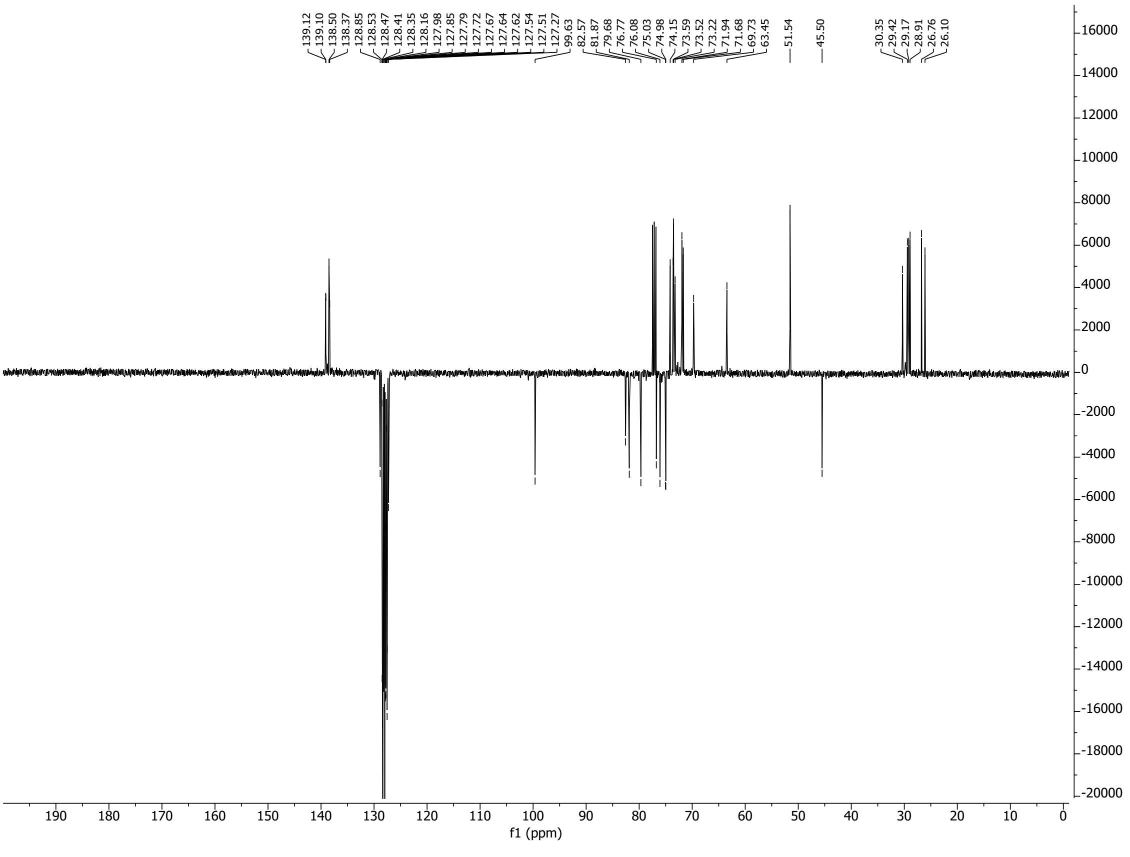

4-*O*-(8-azidooctyl)-2,3,6-tri-*O*-benzyl-(1-4)-β-D-mannopyranosyl-2,3-di-*O*-benzyl-cyclophellitol (35)

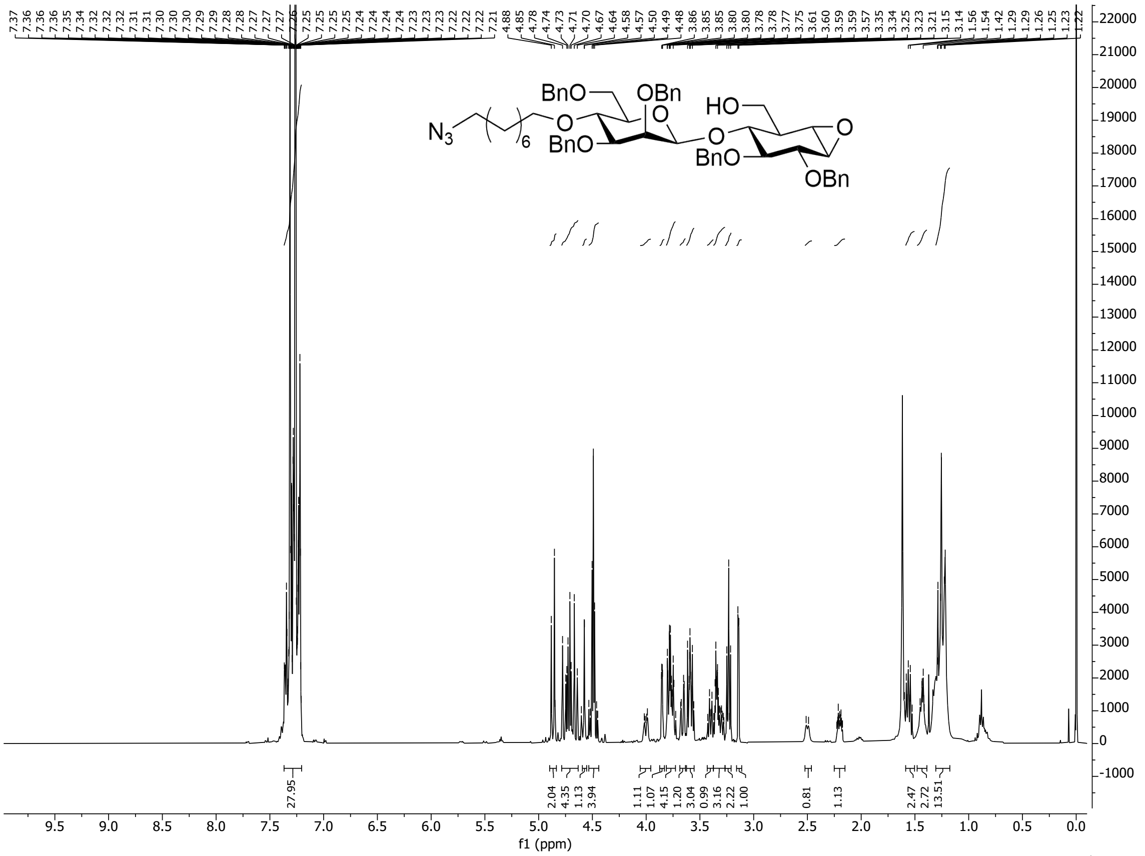

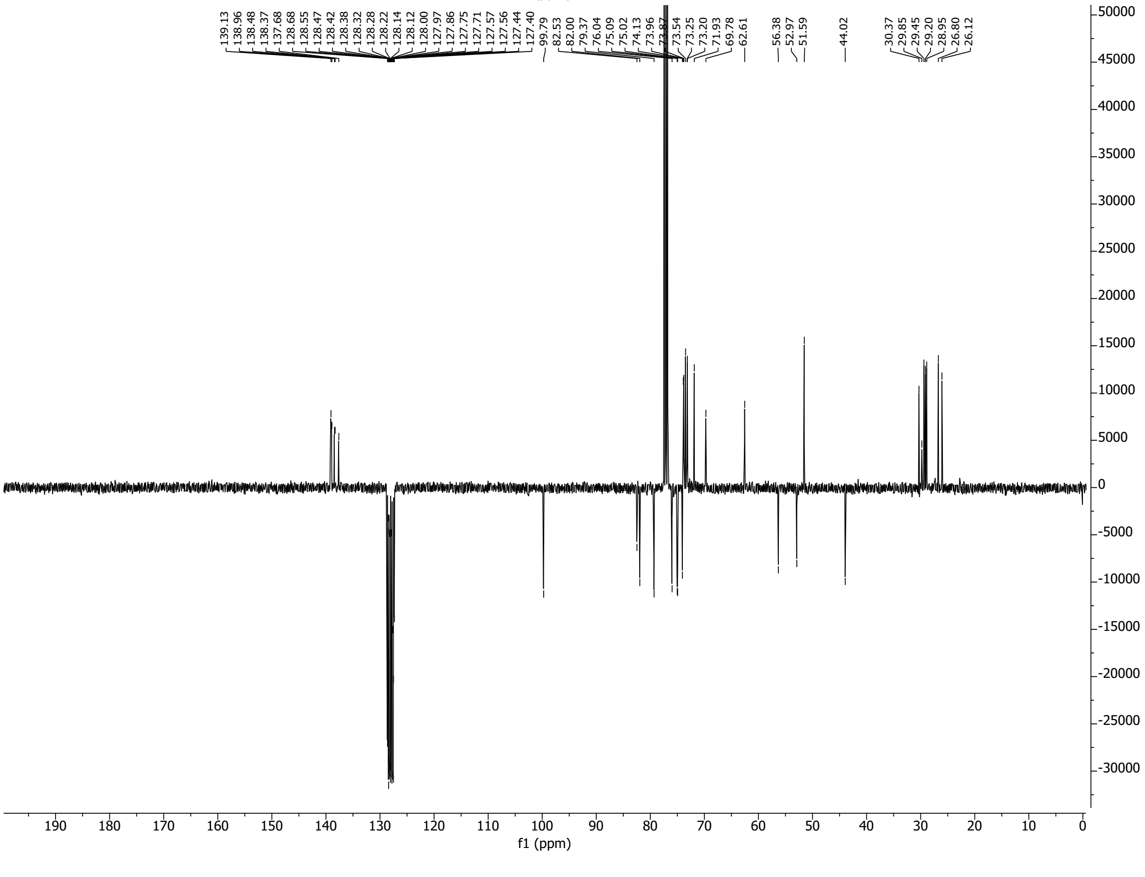

4-*O*-(8-Cy5-octyl)-β-D-mannose-(1-4)-cyclophellitol (14)

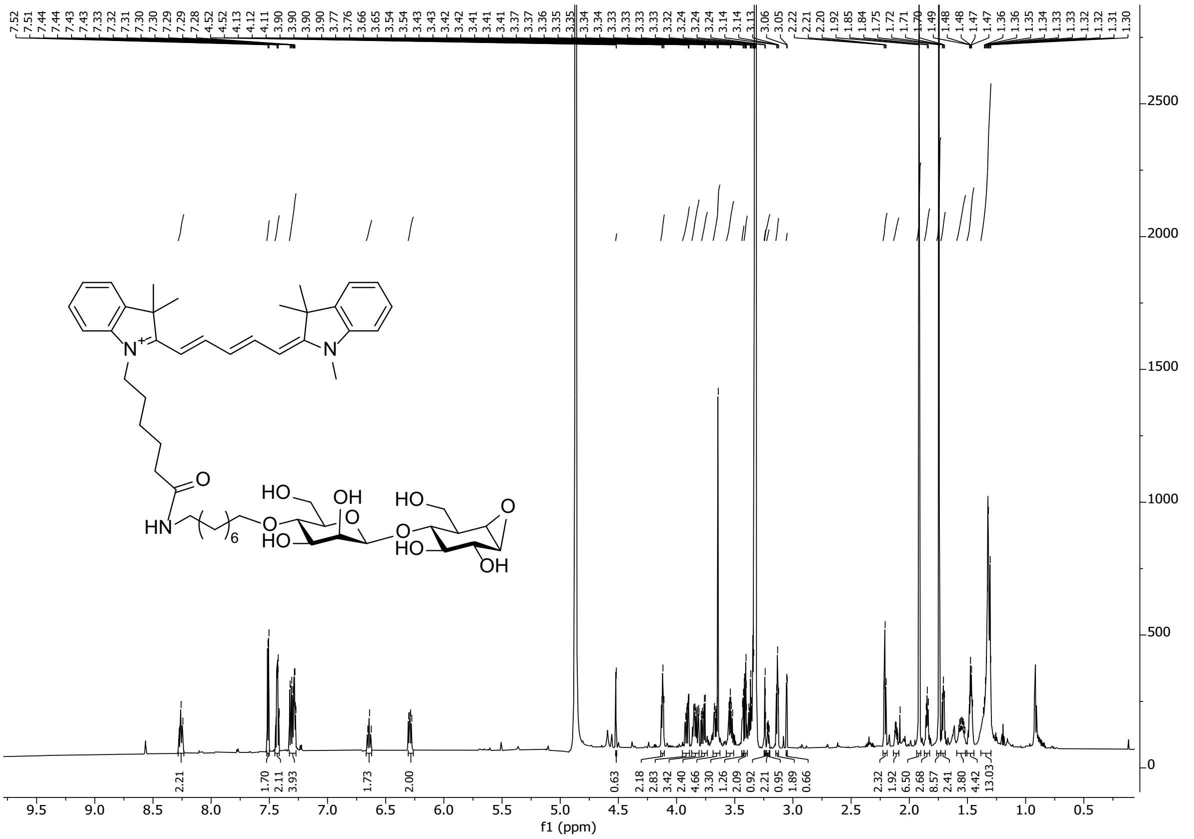

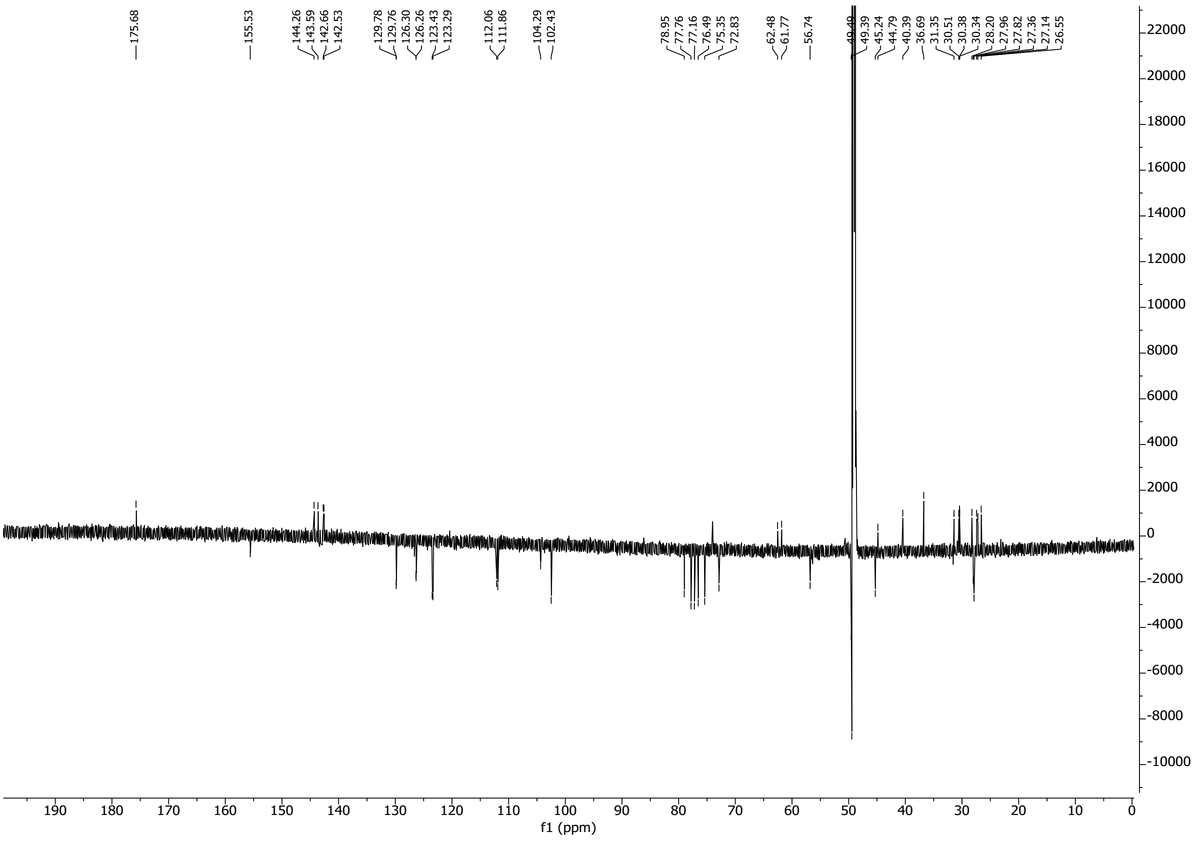

References

1 Z. Armstrong, C.-L. Kuo, D. Lahav, B. Liu, R. Johnson, T. J. M. Beenakker, C. de Boer, C.-S. Wong, E. R. van Rijssel, M. F. Debets, B. I. Florea, C. Hissink, R. G. Boot, P. P. Geurink, H. Ovaa, M. van der Stelt, G. A. van der Marel, J. D. C. Codée, J. M. F. G. Aerts, L. Wu, H. S. Overkleeft and G. J. Davies, *J. Am. Chem. Soc.*, 2020, **142**, 13021-13029.

2 K.-Y. Li, J. Jiang, M. D. Witte, W. W. Kallemeijn, H. van den Elst, C.-S. Wong, S. D. Chander, S. Hoogendoorn, T. J. M. Beenakker, J. D. C. Codée, J. M. F. G. Aerts, G. A. van der Marel and H. S. Overkleeft, *Eur. J. Org. Chem.*, 2014, 6030-6043.

3 M. Arentshorst, P. Kooloth Valappil, L. Mózsik, T. J. G. Regensburg-Tuïnk, S. J. Seekles, G. Tjallinks, M. W. Fraaije, J. Visser and A. F. J. Ram, *FEBS J*., 2023, **290**, 5127-5140.

4 M. Arentshorst, A. F. J. Ram and V. Meyer, *Methods Mol. Biol*., 2012, **835**, 133-150.

5 E. Alazi, J. Niu, J. E. Kowalczyk, M. Peng, M. V. Aguilar Pontes, J. A. L. van Kan, J. Visser, R. P. de Vries and A. F. J. Ram, *FEBS Lett.*, 2016, **590**, 1804-1815.

6 N. G. S. McGregor, C. de Boer, Q. P. O. Foucart, T. Beenakker, W. A. Offen, J. D. C. Codée, L. I. Willems, H. S. Overkleeft and G. J. Davies, *ACS Cent. Sci*., 2023, **9**, 2306-2314.

7 R. J. Williams, J. Iglesias-Fernández, J. Stepper, A. Jackson, A. J. Thompson, E. C. Lowe, J. M. White, H. J. Gilbert, C. Rovira, G. J. Davies and S. J. Williams, *Angew. Chem. Int. Ed*., 2013, **53**, 1087-1091.

8 V. M.-A. Ducros, D. L. Zechel, G. N. Murhudov, H. J. Gilbert, L. Szabó, D. Stoll, S. G. Withers and G. J. Davies, *Angew. Ed. Int. Ed.*, 2022, **41**, 2824-2827.

9 F. W. Studier, *Protein Expr. Purif*., 2005, **41**, 207-234.

10 The UniProt Consortium, *Nucleic Acids Res.*, 2025, **53**, D609-D167.

11 D. A. Polasky, D. J. Geiszler, F. Yu, K. Li, G. C. Teo and A. I. Nesvizhskii, *Mol. Cell. Prot.*, 2023, **22**, 100538.

12 F. Yu, S. E. Haynes, G. C. Teo, D. M. Avtonomov, D. A. Polasky, and A. I. Nesvizhskii, *Mol. Cell. Prot.*, 2020, **19**, 1575-1585.

13 A. M. Frankenfield, J. Ni, M. Ahmed, and L. Hao, *J. Proteome Res.*, 2022, **21**, 2104-2113.

14 V. Borlandelli, W. Offen, O. Moroz, A. Nin-Hill, N. McGregor, L. Binkhorst, A. Ishiwata, Z. Armstrong, M. Artola, C. Rovira, G. J. Davies and H. S. Overkleeft, *ACS Chem. Biol*., 2023, **18**, 2564-2573.

15 G. Winter, *J. Appl. Cryst.*, 2009, **43**, 186-190.

16 J. Jumper, R. Evans, A. Pritzel, T. Green, M. Figurnov, O. Ronneberger, K. Tunyasuvunakool, R. Bates, A. Žídek, A. Potapenko, A. Bridgland, C. Meyer, S. Kohl, A. Ballard, A. Cowie, B. Romera-Paredes, S. Nikolov, R. Jain, J. Adler, T. Back, S. Petersen, D. Reiman, E. Clancy, M. Zielinski, M. Steinegger, M. Pacholska, T. Berghammer, S. Bodenstein, D. Silver, O. Vinyals, A. Senior, K. Kavukcuoglu, P. Kohli and D. Hassabis, *Nature*, 2021, **596**, 583–589

17 A. J. McCoy, R. W. Grosse-Kunstleve, P. D. Adams, M. D. Winn, L. C. Storoni, and R. J. Read, *J. Appl. Cryst.*, 2007 **40**, 658-674.

18 P. Emsley, B. Lohkamp, W. G. Scott and K. Cowtan, *Acta Cryst.*, 2010, **66**, 486-501.

19 G. N. Murshudov, P. Skubák, A. A. Lebedev, N. S. Pannu, R. A. Steiner, R. A. Nicholls, M. D. Winn, F. Long and A. A. Vagin, *Acta Crystallogr. D Struct. Biol*., 2011, **67**, 355-367.

20 F. Long, R. A. Nicholls, P. Emsley, S. Grazulis, A. Merkys, A. Vaitkus and G. N. Murshudov, *Acta Cryst.*, 2017, **73**, 112-122.

21 A. Cartmell, E. Topakas, V. M. A. Ducros, M. D. L. Suits, G. J. Davies and H. J. Gilbert, *J. Biol. Chem.*, 2008, **283**, 34403-34413.

22 W. Kabsch, *Acta Crystallogr. D Biol. Crystallogr*., 2010, **66**, 1251132.

23 G. Winter, D. G. Waterman, J. M. Parkhurst, A. S. Brewster, R. J. Gildea, M. Gerstel, L. Fuentes-Montero, M. Vollmar, T. Michels-Clark, I. D. Young, N. K. Sauter and G. Evans, *Acta Crystallogr. D Struct. Biol*., 2018, **74**, 85-97.

24 R. J. Gildea, J. Beilsten-Edmands, D. Axford, S. Horrell, P. Aller, J. Sandy, J. Sanchez-Weatherby, C. David Owen, P. Lukacik, C. Strain-Damerell, R. L. Owen, M. A. Walsh and G. Winter, *Acta Crystallogr. D Struct. Biol*., 2022, **78**, 752-769.

25 J. Agirre, M. Atanasova, H. Bagdonas, C. B. Ballard, A. Baslé, J. Beilsten-Edmands, R. J. Borges, D. G. Brown, J. J. Burgos-Mármol, J. M. Berrisford, P. S. Bond, I. Caballero, L. Catapano, G. Chojnowski, A. G. Cook, K. D. Cowtan, T. I. Croll, J. Debreczeni, N. E. Devenish, E. J. Dodson, T. R. Drevon, P. Emsley, G. Evans, P. R. Evans, M. Fando, J. Foadi, L. Fuentes-Montero, E. F. Garman, M. Gerstel, R. J. Gildea, K. Hatti, M. L. Hekkelman, P. Heuser, S. W. Hoh, M. A. Hough, H. T. Jenkins, E. Jiménez, R. P. Joosten, R. M. Keegan, N. Keep, E. B. Krissinel, P. Kolenko, O. Kovalevskiy, V. S. Lamzin, D. M. Lawson, A. A. Lebedev, A. G. W. Leslie, B. Lohkamp, F. Long, M. Malý, A. J. McCoy, S. J. McNicholas, A. Medina, C. Millán, J. W. Murray, G. N. Murshudov, R. A. Nicholls, M. E. M. Noble, R. Oeffner, N. S. Pannu, J. M. Parkhurst, N. Pearce, J. Pereira, A. Perrakis, H. R. Powell, R. J. Read, D. J. Rigden, W. Rochira, M. Sammito, F. S. Rodríguez, G. M. Sheldrick, K. L. Shelley, F. Simkovic, A. J. Simpkin, P. Skubak, E. Sobolev, R. A. Steiner, K. Stevenson, I. Tews, J. M. H. Thomas, A. Thorn, J. T. Valls, V. Uski, I. Usón, A. Vagin, S. Velankar, M. Vollmar, H. Walden, D. Waterman, K. S. Wilson, M. D. Winn, G. Winter, M. Wojdyr and K. Yamashita, *Acta Crystallogr. D Struct. Biol*., 2023, **79**, 449-461.

26 A. Vagin and A. Teplyakov, *Acta Crystallogr. D Struct. Biol*., 2010, **66**, 22-25.

27 A. Casañal, B. Lohkamp and P. Emsley, *Protein Sci.*, 2020, **29**, 1055-1064.

28 A. A. Lebedev, P. Young, M. N. Isupov, O. V. Moroz, A. A. Vagin and G. N. Murshudov, *Acta Crystallogr. D Struct. Biol.*, 2012, **68**, 431-440.

29 R. Car and M. Parrinello, *Phys. Rev. Lett.*, 1985, 55, 2471-2474.

30 N. Troullier and J. L. Martins, *Phys. Rev. B*, 1991, **43**, 1993-2006.

31 J. P. Perdew, K. Burke and M. Ernzerhof, *Phys. Rev. Lett.*, 1996, **77**, 3865-3868.

32 V. Borlandelli, Z. Armstrong, A. Nin-Hill, J. D. C. Codée, L. Raich, M. Artola, C. Rovira, G. J. Davies and H. S. Overkleeft, *ChemMedChem*, 2023, **18**, e202200580.

33 M. Artola, L. Wu, M. J. Ferraz, C. L. Kuo, L. Raich, I. Z. Breen, W. A. Offen, J. D. C. Codée, G. A. Van Der Marel, C. Rovira, J. M. F. G. Aerts, G. J. Davies and H. S. Overkleeft, *ACS Cent. Sci.*, 2017, **3**, 784-793.

34 M. Petricevic, L. F. Sobala, P. Z. Fernandes, L. Raich, A. J. Thompson, G. Bernardo-Seisdedos, O. Millet, S. Zhu, M. Sollogoub, J. Jiménez-Barbero, C. Rovira, G. J. Davies and S. J. Williams, *J. Am. Chem. Soc.,* 2017, **139**, 1089-1097.

35 X. Biarnés, A. Ardèvol, A. Planas, C. Rovira, A. Laio and M. Parrinello, *J. Am. Chem. Soc.*, 2007, **129**, 10686-10693.

36 A. Barducci, M. Bonomi and M. Parrinello, *Wiley Interdiscip. Rev. Comput. Mol. Sci.*, 2011, **1**, 826-843.

37 A. Laio and M. Parrinello, *Proc. Natl. Acad. Sci. U. S. A.,* 2002, **99**, 12562-12566.

38 D. Cremer and J. A. Pople, *J. Am. Chem. Soc.,* 2002, **97**, 1354-1358.
